# A monograph on the genus *Tetraserica* from the Indochinese region (Coleoptera, Scarabaeidae, Sericini)

**DOI:** 10.3897/zookeys.837.32057

**Published:** 2019-04-09

**Authors:** Silvia Fabrizi, Vivian Dalstein, Dirk Ahrens

**Affiliations:** 1 Centre for Taxonomy and Evolutionary Research; Zoologisches Forschungsmuseum Alexander Koenig Bonn, Adenauerallee 160, 53113 Bonn, Germany Centre for Taxonomy and Evolutionary Research; Zoologisches Forschungsmuseum Alexander Koenig Bonn Bonn Germany

**Keywords:** Beetles, chafers, Indochina, new species, *
Tetraserica
*

## Abstract

In this monograph on the Indochinese species of *Tetraserica* Ahrens, 2004 all species distributed in Thailand, Laos, Vietnam, Cambodia, Myanmar, and mainland Malaysia are covered as well as those of the Indian province Mizoram. From this revision, the following new combinations result: *Tetrasericagressitti* (Frey, 1972), **comb. n.**, *T.laotica* (Frey, 1972), **comb. n.**, *T.satura* (Brenske, 1898), **comb. n.**, *T.sejugata* (Brenske, 1898), **comb. n.**, *T.siantarensis* (Moser, 1922), **comb. n.**, *T.spinicrus* (Frey, 1972), **comb. n.**, *T.vietnamensis* (Frey, 1969), **comb. n.**, and *T.wapiensis* (Frey, 1972), **comb. n.** Two new synonyms were found: *Tetrasericamidoriae* Kobayashi, 2017 (**syn. n**.) = *T.laotica* (Frey, 1972); *T.graciliforceps* Liu et al. 2014 (**syn. n.**) = *T.satura* (Brenske, 1898). The lectotypes of *Tetrasericagestroi* (Brenske, 1898), *T.miniatula* (Moser, 1915), and *T.siantarensis* (Moser, 1922) are designated. 116 *Tetraserica* species were recorded from Indochina, among which 88 new species are described: *Tetrasericaallochangshouensis***sp. n.**, *T.allomengeana***sp. n.**, *T.allosejugata***sp. n.**, *T.angkorthomensis***sp. n.**, *T.angkorwatensis***sp. n.**, *T.appendiculata***sp. n.**, *T.auriculata***sp. n.**, *T.bachmaensis***sp. n.**, *T.banhuaipoensis***sp. n.**, *T.bansanpakiana***sp. n.**, *T.bolavensensis***sp. n.**, *T.breviforceps***sp. n.**, *T.cattienensis***sp. n.**, *T.champassakana***sp. n.**, *T.constanti***sp. n.**, *T.cucphongensis***sp. n.**, *T.curviforceps***sp. n.**, *T.desalvazzai***sp. n.**, *T.doiphukhaensis***sp. n.**, *T.doipuiensis***sp. n.**, *T.doisuthepensis***sp. n.**, *T.dongnaiensis***sp. n.**, *T.falciforceps***sp. n.**, *T.falciformis***sp. n.**, *T.feresiantarensis***sp. n.**, *T.filiforceps***sp. n.**, *T.fulleri***sp. n.**, *T.phukradungensis***sp. n.**, *T.geiserae***sp. n.**, *T.giulianae***sp. n.**, *T.infida***sp. n.**, *T.jakli***sp. n.**, *T.khaosoidaoensis***sp. n.**, *T.kiriromensis***sp. n.**, *T.koi***sp. n.**, *T.kollae***sp. n.**, *T.konchurangensis***sp. n.**, *T.kontumensis***sp. n.**, *T.loeiensis***sp. n.**, *T.lucai***sp. n.**, *T.microfurcata***sp. n.**, *T.microspinosa***sp. n.**, *T.multiangulata***sp. n.**, *T.nahaeoensis***sp. n.**, *T.nakaiensis***sp. n.**, *T.namnaoensis***sp. n.**, *T.neouncinata***sp. n.**, *T.nonglomensis***sp. n.**, *T.nussi***sp. n.**, *T.olegi***sp. n.**, *T.pahinngamensis***sp. n.**, *T.pailinensis***sp. n.**, *T.parasetuliforceps***sp. n.**, *T.paratonkinensis***sp. n.**, *T.petrpacholatkoi***sp. n.**, *T.phatoensis***sp. n.**, *T.phoupaneensis***sp. n.**, *T.pluriuncinata***sp. n.**, *T.pseudoliangheensis***sp. n.**, *T.pseudoruiliensis***sp. n.**, *T.pseudouncinata***sp. n.**, *T.quadriforceps***sp. n.**, *T.quadrifurcata***sp. n.**, *T.rihai***sp. n.**, *T.romae***sp. n.**, *T.rubrithorax***sp. n.**, *T.sapana***sp. n.**, *T.semidamadiensis***sp. n.**, *T.semipingjiangensis***sp. n.**, *T.semiruiliensis***sp. n.**, *T.semishanensis***sp. n.**, *T.setuliforceps***sp. n.**, *T.shanensis***sp. n.**, *T.smetsi***sp. n.**, *T.margheritae***sp. n.**, *T.soppongana***sp. n.**, *T.spanglerorum***sp. n.**, *T.spinotibialis***sp. n.**, *T.subrotundata***sp. n.**, *T.tanahrataensis***sp. n.**, *T.thainguyensis***sp. n.**, *T.trilobiforceps***sp. n.**, *T.ululalatensis***sp. n.**, *T.umphangensis***sp. n.**, *T.vari***sp. n.**, *T.veliformis***sp. n.**, *T.vientianeensis***sp. n.**, and *T.xiengkhouangensis***sp. n.** A key to the Indochinese *Tetraserica* species is given and distributions as well as the habitus and male genitalia of all species are illustrated.

## Introduction

While the genus *Tetraserica* Ahrens, 2004 included earlier only species from Indian subcontinent and Myanmar, Liu et al. (2014) added a significant number of species from China and recently additional species were described by Ahrens and Fabrizi (2016) from northeastern India and by Kobayashi (2017, 2018) from Thailand. Several molecular phylogenetic analyses confirmed the monophyly of *Tetraserica* (Ahrens & Vogler, 2008; Liu et al. 2015; Eberle et al. 2016). Based on a thorough examination of all type material of Asian mainland Sericini and an assessment of DNA-informed species boundaries (Dalstein et al. 2019) we revise here the taxonomy of the *Tetraserica* species so far known from Indochina. We found 88 new species, which are described herein. A key to the Indochinese *Tetraserica* species is given, and their distributions, as well as habitus and male genitalia, are illustrated.

## Materials and methods

The terminology and methods used for measurements, specimen dissection, and genital preparation follow Ahrens (2004). Data from specimens examined are cited in the text with original label contents given in quotation marks, multiple labels are separated by a “/”. Male genitalia were glued to a small pointed card attached to the specimen. Descriptions and illustrations of new taxa are based on the holotype or lectotype specimen, while the variation of other specimens is given separately. All descriptions and measurements were made under an Olympus SZX 12 microscope, and all genital and habitus illustrations were made with a digital camera (AxioCam HRc) attached to a stereo microscope (Zeiss Stereo Discovery V20) and Axio Version 4.8 software. The distribution maps were generated using Q-GIS 2.0.1 and Inscape software.

Type specimens and other examined material are deposited in the following institutions or collections:

**BPBM**Bernice P. Bishop Museum, Honolulu (Hawai), USA;

**BYU**Brigham Young University, Monte L. Bean Life Science Museum Utah, Provo, USA;

**CASH** coll. A Skale, Hof, Germany;

**CF** coll. G Frey (at the NHMB), Switzerland;

**CNAR** coll. A Napolov, Riga, Latvia;

**CPPB** coll. P Pacholátko, Brno, Czech Republic;

**HNHM**Hungarian Natural History Museum, Budapest, Hungary;

**HWML**Museum Nebraska University, Lincoln, Nebraska, USA;

**ISEA** Russian Academy of Sciences, Institute for Systematics and Ecology of Animals, Novosibirsk, Russia;

**ISNB**Institut Royal des Sciences naturelles de Belgique, Bruxelles, Belgium;

**MNHN**Museum national d’Histoire naturelle, Paris, France;

**MSNG**Museo Civico di Storia Naturale G Doria, Genova, Italy;

**MSNM**Museo Civico di Storia Naturale, Milano, Italy;

**MZUF**Museo Zoologico „La Specola”, Università di Firenze, Italy;

**NBAIR** National Bureau of Agricultural Insect Resources, Bangalore (India);

**NHMB**Naturhistorisches Museum, Basel, Switzerland;

**NHMUK**Natural History Museum, London, UK;

**NHMW**Natural History Museum Vienna, Austria;

**NME**Naturkundemuseum Erfurt, Germany;

**NMPC**National Museum Prague (Natural History), Czech Republic;

**QSBG**Queen Sirikit Botanic Garden, Chang Mai, Thailand;

**RMNH**Naturalis Biodiversity Centre Leiden, (Netherlands);

**SMTD**Senckenberg Museum für Tierkunde, Dresden, Germany;

**UMRM**Columbia, University of Missouri, WR Enns Entomology Museum, USA;

**UMIC**University of Mississippi, USA;

**VNMN**Vietnam National Museum of Nature, Hanoi (Vietnam);

**USNM**National Museum of Natural History, Washington D.C., U.S.A.;

**ZFMK**Zoologisches Forschungsmuseum A Koenig, Bonn, Germany;

**ZIN**Russian Academy of Sciences, Zoological Institute, St Petersburg, Russia;

**ZMAN**Universiteit van Amsterdam, Zoologisch Museum Amsterdam (now: Naturalis Leiden), Netherlands;

**ZMHB**Museum für Naturkunde, Berlin, Germany;

**ZSM**Zoologische Staatssammlung, München, Germany.

## Taxonomy

### Tetraserica

Taxon classificationAnimaliaColeopteraMelolonthidae

Ahrens, 2004

Tetraserica Ahrens, 2004: 168 (type species by original designation: Neosericagestroi Brenske, 1898); Liu et al. 2014: 83; Ahrens and Fabrizi 2016: 122; Kobayashi 2017: 33, 2018: 57.

#### Diagnosis.

Body moderately large to large (6–12 mm), mostly dark brown; ventral surface reddish brown; dorsal surface dull and glabrous.

Labroclypeus subtrapezoidal, wider than long, widest at base, lateral margins moderately convex and convergent to strongly rounded anterior angles, anterior margin weakly sinuate medially, margins moderately reflexed; surface weakly convex, moderately shiny, finely and densely punctate; frontoclypeal suture indistinctly incised, flat and weakly curved medially; ocular canthus short and triangular, impunctate, with a single terminal seta. Frons dull, with sparse, fine punctures, with single erect setae beside each eye. Antenna yellowish, with ten antennomeres; club composed of four antennomeres in male, straight, rarely longer than 1.5 times as the remaining antennomeres combined; club in female composed of three antennomeres, as long as the remaining antennomeres combined. Mentum elevated and slightly flattened anteriorly.

Pronotum moderately wide and strongly convex, lateral margins evenly convex, more strongly narrowed anteriorly towards sharp and slightly produced anterior angles. Anterior margin of pronotum slightly convex, with fine complete marginal line. Posterior angles blunt or strongly rounded. Surface finely and densely punctate, except minute setae glabrous, lateral and lateral anterior margins sparsely setose. Hypomeron not carinate. Scutellum triangular, finely and densely punctate.

Elytra oblong, widest just behind middle, striae distinctly impressed, finely and moderately densely punctate, intervals distinctly convex, with coarse and dense punctures concentrated along striae, with very minute setae in punctures; epipleural edge robust, ending at weakly curved and slightly blunt external apical angle of elytra, epipleura densely setose, apical border with a broad fringe of microtrichomes (100×).

Ventral surface weakly shiny, finely and densely punctate, metasternum sparsely covered with fine, short, or very minute setae, metacoxa glabrous, with a few single setae laterally; abdominal sternites finely and densely punctuate, with a transverse row of coarse punctures, each bearing a robust seta. Mesosternum between mesocoxae as wide as mesofemur. Pygidium weakly convex and dull, densely punctate, without smooth midline, almost glabrous, but with a few longer setae along apical margin; pygidium without strong sexual dimorphism.

Legs moderately wide; femora finely and sparsely punctate; metafemur wide and moderately shiny or dull, anterior margin acute, posterior margin smooth ventrally and only weakly widened in apical half, posterior margin smooth dorsally, with a few short setae basally. Posterior margin of metafemur generally straight or slightly convex. Metatibia moderately wide to wide and moderately long, widest at half of metatibial length, dorsal margin sharply carinate, with two groups of spines; lateral face finely and sparsely punctate; ventral edge finely serrated, with four robust equidistant setae, medial face smooth, apex interiorly near tarsal articulation with a shallow sinuation. Tarsomeres with fine, very dense setae ventrally on distal half, neither laterally nor dorsally carinate, dorsally smooth; metatarsomeres with a strongly serrated ridge ventrally and glabrous; first metatarsomere slightly shorter than two following tarsomeres combined, one third of its length longer than dorsal tibial spine. Protibia short, bidentate; anterior claws symmetrical, basal tooth of both claws bluntly truncate at apex.

Aedeagus: Phallobase with a more or less long median ventral extension (median phallobasal lamina).

#### Remarks.

*Tetraserica* differs from closely related genera, *Microserica* Brenske, 1894 and *Trioserica* Moser, 1922, by lacking the ventral carina on hypomeron. From *Microserica* it also differs by lacking the sexual dimorphism of the pygidium, from *Trioserica* by the bidentate protibia. In contrast to the *Microserica*, species of *Tetraserica* are active at night and are attracted by light.

#### Distribution.

The genus is distributed almost in the entire Oriental region except the southern Indian subcontinent; we know that additional species described by various authors are so far assigned to “*Neoserica*” from Philippines, Sumatra, and Borneo but that awaits a formal revision (Ahrens 2004).

##### Key to Indochinese species of *Tetraserica* Ahrens, 2004 (based on males):

**Table:** 

1	Posterior margin of metafemur straight or slightly convex	**19**
–	Posterior margin of metafemur with blunt tooth or sharp hook	**2**
2	Posterior margin of metafemur with blunt tooth	**3**
–	Posterior margin of metafemur with sharp hook	**12**
3	Left paramere long and narrow	**4**
–	Left paramere short and stout	**8**
4	Median lamina of phallobase as long or nearly as long as phallobase	**5**
–	Median lamina of phallobase less than ¼ as long as phallobase. Left paramere long and filiform	**7**
5	Left paramere as long as phallobase or longer, straight and not filiform	**6**
–	Left paramere nearly half as long as phallobase, curved externally, filiform	*** T. vietnamensis ***
–	Left paramere more than half as long as phallobase. Left paramere split shortly before apex into two filiform but flattened branches	***T.multiangulata* sp. n.**
6	Right paramere split in two lobes	*** T. pingjiangensis ***
–	Right paramere simple	***T.semipingjiangensis* sp. n.**
7	Right paramere shorter than phallobase, its dorsal lobe short and basally directed	*** T. takahashii ***
–	Right paramere as long as phallobase, its dorsal lobe long and distally directed	***T.phoupaneensis* sp. n.**
8	Dorsal lobe of right paramere short, not exceeding length of ventral one	*** T. maoershanensis ***
–	Dorsal lobe of right paramere longer, exceeding length of ventral one	**9**
9	Left paramere more narrow, dorsal margin weakly and evenly curved	*** T. sculptilis ***
–	Left paramere stout, dorsal margin bluntly angulate	**10**
10	Dorsal lobe of right paramere evenly curved. Apex of left paramere abruptly narrowed at apex	*** T. daqingshanica ***
–	Dorsal lobe of right paramere in basal half wide and straight, in apical half curved. Apex of left paramere gently narrowed at apex	**11**
11	Left paramere one quarter as wide as length of antennal club	***T.neouncinata* sp. n.**
–	Left paramere two thirds as wide as length of antennal club	***T.pseudouncinata* sp. n.**
12	Metafemur basally not widened, sharp hook behind basal third of metafemur	**13**
–	Metafemur basally strongly widened, sharp hook behind basal quarter of metafemur	*** T. spinicrus ***
13	Eyes smaller, ratio diameter/interocular distance ≤ 0.59	**14**
–	Eyes larger, ratio diameter/interocular distance ≥0.63. Dorsal lobe of right paramere very small and bent basally	**17**
14	Left paramere with two branches	**15**
–	Left paramere simple. Dorsal lobe of right paramere longer than the ventral one	**16**
15	Left paramere split into a dorsal hook and a basal filiform branch. Dorsal lobe of right paramere much shorter than the ventral one	***T.kontumensis* sp. n.**
–	Left paramere split into two similar branches (lateral view). Dorsal lobe of right paramere longer than the ventral one	***T.lucai* sp. n.**
16	Dorsal lobe of right paramere strongly curved, large and directed distally, exceeding ventral lobe by far, at apex with a small additional hook	*** T. liangheensis ***
–	Dorsal lobe of right paramere weakly curved, weakly exceeding ventral lobe, at apex without additional hook	***T.doisuthepensis* sp. n.**
17	Median lamina of phallobase evenly narrowed from base to apex, sharply pointed at apex. Basal portion of right paramere (lateral view) much wider than apical part	*** T. wandingensis ***
–	Median lamina of phallobase wide, abruptly narrowed and rounded at apex. Basal portion of right paramere (lateral view) not wider than apical part	**18**
18	Right paramere strongly bent at middle (lateral view)	*** T. wiangpapaoana ***
–	Right paramere weakly bent at middle (lateral view)	*** T. masumotoi ***
19	Median lamina of phallobase short, distinctly shorter than phallobase	**20**
–	Median lamina of phallobase long, at least subequal to length of phallobase	**82**
20	Median lamina of phallobase medium in length, approx. three quarter of phallobase length	**21**
–	Median lamina of phallobase short, subequal to at maximum half of length of phallobase	**37**
21	Right paramere at base without spines	**22**
–	Right paramere at base with a comb of short spines	***T.quadriforceps* sp. n.**
22	Right paramere simple	**23**
–	Right paramere with a dorsal and a ventral lobe. Metatibia robust, ratio length/width ca. 1/3.0	**35**
23	Left paramere simple	**24**
–	Left paramere split into two long and narrow filiform lobes	**27**
24	Right paramere as long as phallobase. Metatibia slender, ratio length/width ca. 1/ 3.4. Median lamina of phallobase evenly wide and rounded at apex	*** T. shunbiensis ***
–	Right paramere one third as long as phallobase. Metatibia stouter, ratio length/width ca. 1/ 3.1	**26**
–	Right paramere half as long as phallobase	**25**
25	Left paramere before apex with a blunt lateral tooth. Right paramere slightly shorter than medial phallobasal lamina	***T.champassakana* sp. n.**
–	Left paramere before apex without blunt lateral tooth. Right paramere as long as medial phallobasal lamina	***T.doipuiensis* sp. n.**
26	Left paramere nearly straight. Median lamina of phallobase narrowed distally, curved dorsally and with a dorsal tooth at apex	***T.nahaeoensis* sp. n.**
–	Left paramere strongly and evenly curved ventrally. Median lamina of phallobase narrowed distally, curved dorsally and with a dorsal tooth at apex	***T.pahinngamensis* sp. n.**
27	Dorsal lobe of left paramere shorter than ventral lobe	**28**
–	Dorsal lobe of left paramere distinctly longer than the ventral one. Apex of median phallobasal lamina moderately pointed	**32**
28	Dorsal lobe of left paramere slightly less than half as long as ventral lobe	**29**
–	Dorsal lobe of left paramere slightly more than half as long as ventral lobe	*** T. wapiensis ***
29	Right paramere distinctly shorter than median lamina of phallobase	**30**
–	Right paramere approx. as long as median lamina of phallobase	**31**
30	Apex of median phallobasal lamina blunt and curved abruptly dorsally. Right paramere bent only once	***T.kiriromensis* sp. n.**
–	Apex of median phallobasal lamina sharply pointed. Right paramere bent twice	***T.banhuaipoensis* sp. n.**
31	Right paramere bent twice, slightly narrower in lateral view. Apex of median phallobasal lamina sharply pointed	*** T. matsumotoi ***
–	Right paramere bent only once, slightly wider in lateral view. Apex of median phallobasal lamina strongly rounded	***T.subrotundata* sp. n.**
32	Dorsal lobe of left paramere a quarter of its length longer than the ventral one. Right paramere narrower (in lateral view) and half as long as median lamina of phallobase	**33**
–	Dorsal lobe of left paramere twice as long as the ventral one	**34**
33	Dorsal lobe of left paramere only little wider than the ventral one, nearly straight in dorsal view. Right paramere bent in an obtuse angle at middle	***T.loeiensis* sp. n.**
–	Dorsal lobe of left paramere distinctly wider than the ventral one, slightly curved (dorsal view). Right paramere bent in a nearly sharp angle at middle	***T.spanglerorum* sp. n.**
34	Dorsal lobe of left paramere distinctly exceeding the median lamina of phallobase. Right paramere wider (in lateral view) and little shorter than median lamina of phallobase	***T.pailinensis* sp. n.**
–	Dorsal lobe of left paramere distinctly shorter than the median lamina of phallobase. Right paramere narrower (in lateral view) and distinctly shorter than median lamina of phallobase	***T.auriculata* sp. n.**
35	Left paramere split into two long and narrow filiform lobes. Ventral lobe of right paramere longer, dorsal lobe weakly curved	*** T. doisangensis ***
–	Left paramere simple. Ventral lobe of right paramere short, dorsal lobe strongly curved	**36**
36	Left paramere moderately long and wide, not exceeding the median ventral lamina of phallobase; convex at apex	*** T. sigulianshanica ***
–	Left paramere long and filiform, widely exceeding the median ventral lamina of phallobase; bluntly widened at apex	***T.curviforceps* sp. n.**
37	Eyes of medium size, ratio diameter/interocular distance ≥ 0.59	**61**
–	Eyes small, ratio diameter/interocular distance ≤ 0.56	**38**
38	Dorsal surface of body unicoloured	**39**
–	Body blackish brown, pronotum reddish	***T.rubrithorax* sp. n.**
39	Left paramere simple	**44**
–	Left paramere divided into two lobes at base	**40**
40	Medial phallobasal lamina at least 1/4 of phallobasal length	**41**
–	Medial phallobasal lamina at maximum 1/10 of phallobasal length	***T.thainguyensis* sp. n.**
41	Left paramere distinctly shorter than the median ventral lamina of phallobase	***T.khaosoidaoensis* sp. n.**
–	Left paramere (dorsal lobe) at least as long as the median ventral lamina of phallobase	**42**
42	Dorsal lobe of left paramere as long as the median ventral lamina of phallobase	**43**
–	Dorsal lobe of left paramere twice as long as the median ventral lamina of phallobase	***T.rihai* sp. n.**
43	Dorsal lobe of left paramere massive and robust. Median phallobasal lamina shortly curved dorsally	***T.nonglomensis* sp. n.**
–	Dorsal lobe of left paramere filiform. Median phallobasal lamina straight and rounded at apex	***T.nussi* sp. n.**
44	Phallobase in dorsal view only slightly asymmetric. Left and right parameres simple, without two lobes	**45**
–	Phallobase in dorsal view strongly asymmetric	**47**
45	Left paramere nearly twice as long as narrow median phallobasal lamina	**46**
–	Left paramere little longer than large median phallobasal lamina	*** T. laotica ***
46	Left paramere distinctly longer than phallobase. Right paramere strongly curved ventrally at apex	*** T. satura ***
–	Left paramere slightly shorter than phallobase. Right paramere nearly straight (lateral view)	***T.filiforceps* sp. n.**
47	Median lamina of phallobase very short, only one quarter of phallobase length	**50**
–	Median lamina of phallobase approx. half of phallobase length	**48**
48	Robust spines on ventral margin of metatibia subequal in length. Phallobase only little produced at right side	***T.cattienensis* sp. n.**
–	Robust spine at middle of ventral margin of metatibia extremely prolonged and s-shaped, exceeding distal margin of metatibia. Phallobase strongly produced at right side	**49**
49	Median phallobasal lamina wide and blunt at apex, with a dorsal blunt tooth. Left paramere subequal in length with median phallobasal lamina	***T.bansanpakiana* sp. n.**
–	Median phallobasal lamina narrow and sharply pointed at apex, without dorsal tooth. Left paramere distinctly shorter than median phallobasal lamina	***T.margheritae* sp. n.**
50	Both parameres long and filiform, curved ventrally at apex	***T.microspinosa* sp. n.**
–	Left paramere long and filiform, weakly curved; right paramere short and slim, strongly bent at middle	***T.microfurcata* sp. n.**
–	Parameres of other shape	**51**
51	Left paramere short and stout	**52**
–	Left paramere long and narrow, more or less straight	**54**
52	Dorsal lobe of right paramere after basal third strongly curved (dorsal view). Body evenly reddish to dark brown, dorsally only slightly darker	**53**
–	Right paramere without dorsal lobe but simple	***T.doiphukhaensis* sp. n.**
53	Dorsal lobe of right paramere at apex simply pointed (dorsal view)	***T.falciformis* sp. n.**
–	Dorsal lobe of right paramere at apex with a sharp hook (dorsal view)	***T.pseudoliangheensis* sp. n.**
54	Right paramere more or less straight (ventral view). Dorsal surface blackish, ventral surface reddish brown	**55**
–	Right paramere strongly u-shaped curved. Body evenly reddish to dark brown	*** T. senohi ***
55	Right paramere at base not split into two lobes, without spines	**56**
–	Right paramere at base split into two short but widely separated lobes, without spines	**58**
–	Right paramere at base with divergent spines and a distal separated lobe	***T.phatoensis* sp. n.**
56	Right paramere simply pointed. Left paramere narrow, with blunt ventral lobe	**57**
–	Right paramere hammer-shaped, with a sharply pointed apical double spine, one directed dorsally, and a larger one ventrally. Left paramere wider, with fine dorsal basal lobe	***T.romae* sp. n.**
57	Apical lobe of left paramere narrow. Right paramere inserted only little more basally than the left paramere	*** T. longzhouensis ***
–	Apical lobe of left paramere wider. Right paramere inserted much more basally than the left paramere	***T.olegi* sp. n.**
58	Ventral metatibial spine normal, distinctly shorter than first metatarsomere. Left paramere moderately long and moderately narrow. Apex of dorsal lobe of right paramere without short setae	**59**
–	Ventral metatibial spine extremely elongated, distinctly exceeding first metatarsomere. Left paramere long and narrow, without dorsal subbasal lobe. Apex of dorsal lobe of right paramere with dense short setae	***T.bachmaensis* sp. n.**
59	Left paramere with a short dorsal subbasal lobe. Dorsal lobe of right paramere short	***T.cucphongensis* sp. n.**
–	Left paramere without dorsal subbasal lobe	**60**
60	Dorsal lobe of right paramere long	***T.falciforceps* sp. n.**
–	Dorsal lobe of right paramere short	***T.geiserae* sp. n.**
61	Both parameres with dorsal and ventral lobe	**62**
–	One or both parameres simple, without two lobes	**66**
62	Dorsal lobe of right paramere simple, not composed of fine spines	**63**
–	Dorsal lobe of right paramere composed of fine spines	**65**
63	Dorsal lobe of right paramere directed distally	**64**
–	Dorsal lobe of right paramere directed basally	***T.ululalatensis* sp. n.**
64	Dorsal lobe of left paramere as long as ventral one	*** T. fikaceki ***
–	Dorsal lobe of left paramere much shorter than ventral one	*** T. univestris ***
–	Dorsal lobe of left paramere much longer than ventral one	*** T. miniatula ***
65	Dorsal lobe of right paramere shorter, less than 1/3 as long as phallobase	*** T. chiangdaoensis ***
–	Dorsal lobe of right paramere longer, half as long as phallobase	***T.kollae* sp. n.**
66	Both parameres simple, without two lobes	**67**
–	One of parameres complex, with two lobes	**72**
67	Right paramere distinctly wider (dorsal view) than the left one	**68**
–	Right paramere as wide as (dorsal view) the left one	**69**
68	Dorsolateral tooth at basal quarter of right paramere, right paramere narrower (lateral view)	*** T. damaidiensis ***
–	Dorsolateral tooth at basal third of right paramere, right paramere wider (lateral view)	***T.semidamadiensis* sp. n.**
69	Left paramere one third as long as phallobase. Median lamina of phallophase not widened at apex	***T.xiengkhouangensis* sp. n.**
–	Left paramere longer	**70**
70	Left paramere as long as phallobase	**71**
–	Left paramere half as long as phallobase	***T.petrpacholatkoi* sp. n.**
71	Median lamina of phallophase strongly widened and truncate at apex	***T.constanti* sp. n.**
–	Median lamina of phallophase not widened at apex	***T.vientianeensis* sp. n.**
72	Right paramere simple, left one with two lobes	**73**
–	Left paramere simple, right one with two lobes	**78**
73	Right paramere basiventrally strongly widened towards apex, at base with a narrow long basal lobe	*** T. yaoquensis ***
–	Right paramere evenly narrowed towards apex	**74**
74	Dorsal surface dark brown	**76**
–	Dorsal surface yellowish brown	**75**
75	Ventral lobe of left paramere wider, stronger curved ventrally. Dorsal lobe of left paramere bluntly widened before apex	***T.shanensis* sp. n.**
–	Ventral lobe of left paramere narrower, nearly straight (lateral view). Dorsal lobe of left paramere nearly evenly narrowed towards apex	***T.semishanensis* sp. n.**
76	Left paramere with a dorsal lobe distant from base	**77**
–	Left paramere with dorsal lobe at base, lobe robust (nearly half as long as paramere) and at apex convex	***T.breviforceps* sp. n.**
77	Left paramere narrower, with a short fine dorsal lobe before the middle	***T.trilobiforceps* sp. n.**
–	Left paramere shorter and stouter, with a short fine dorsal lobe behind the middle	***T.phukradungensis* sp. n.**
78	Left paramere long, slender	**79**
–	Left paramere shorter, stouter, almost straight	*** T. wangtongensis ***
79	Left paramere bent externally. Distal lobe of right paramere short	**80**
–	Left paramere bent straight, split shortly before apex. Distal lobe of right paramere long, more than half as long as phallobase	***T.jakli* sp. n.**
80	Basal lobe of right paramere short, without spines. Insertion of right parameres lateral	*** T. changjiangensis ***
–	Basal lobe of right paramere moderately long, with trichome-like spines. Insertion of right parameres displaced dorsally	**81**
81	Left paramere half as long as phallobase	***T.setuliforceps* sp. n.**
–	Left paramere as long as phallobase	***T.parasetuliforceps* sp. n.**
82	Right paramere basally with brush of robust trichome-like spines	**83**
–	Right paramere without brush of spines	**102**
83	Left paramere composed of two lobes	**84**
–	Left paramere simple	**88**
84	Left paramere long, as long as the ventral distal phallobasal process	**85**
–	Left paramere short and trifid, 1/3 as long as the ventral distal phallobasal process	**87**
85	Ventral lobe of right paramere abruptly and strongly widened at apex, dorsal lobe of right paramere obsolete but with dense trichomes	**86**
–	Ventral lobe of right paramere not widened at apex, dorsal lobe of right paramere moderately long and narrow, composed of fine spines	***T.pluriuncinata* sp. n.**
86	Ventroapical lobe of right paramere narrower, ventral lobe of left paramere little shorter than the dorsal one	***T.allochangshouensis* sp. n.**
–	Ventroapical lobe of right paramere wider, ventral lobe of left paramere one third as long as the dorsal one	*** T. changshouensis ***
87	Median lamina of phallobase with a small dorsal preapical tooth. Dorsal lobe of left parameres and median lamina of phallobase distinctly longer than phallobase	***T.siantarensis*** (Sumatra)
–	Median lamina of phallobase without dorsal preapical tooth. Dorsal lobe of left parameres and median lamina of phallobase as long as phallobase	***T.feresiantarensis* sp. n.**
88	Left paramere with small lateral basal tooth	*** T. linaoshanica ***
–	Left paramere without small lateral basal tooth	**89**
89	Left paramere split in two filiform branches behind anterior third	**90**
–	Left paramere simple	**94**
90	Median phallobasal lamina convex or simply pointed at apex	**91**
–	Median phallobasal lamina bifurcate at apex	***T.quadrifurcata* sp. n.**
91	Filiform branches of left paramere long, at least approx. one third of paramere length	**92**
–	Filiform branches of left paramere very short, much less than one length of paramere length	**93**
92	Filiform branches of left paramere approx. one third of paramere length. Median phallobasal lamina most of its distal part narrow	*** T. mengeana ***
–	Filiform branches of left paramere approx. two third of paramere length. Median phallobasal lamina evenly narrowed towards apex	***T.allomengeana* sp. n.**
93	Distal lobe of right paramere long and robust, strongly curved upward (lateral view), with a strong subapical ventral tooth	***T.finociliata* sp. n.**
–	Distal lobe of right paramere short, weakly curved (lateral view), without ventral tooth	***T.appendiculata* sp. n.**
94	Left paramere distinctly longer than phallobase	**95**
–	Left paramere as long as phallobase, evenly curved	***T.sapana* sp. n.**
95	Left paramere bent twice or entirely curved	**100**
–	Left paramere in basal half straight	**96**
96	Left paramere before apex with tiny tooth or tooth-like process	**97**
–	Left paramere before apex without tiny tooth	***T.konchurangensis* sp. n.**
97	Median lamina of phallobase straight and strongly narrowed after basal the third	**98**
–	Median lamina of phallobase distinctly curved and evenly narrowed from base to apex	***T.namnaoensis* sp. n.**
98	Median lamina of phallobase distinctly shorter than left paramere, simply pointed, slightly rounded at apex	**99**
–	Median lamina of phallobase approx. as long as left paramere, slightly bifurcate at apex. Preapical tooth of left paramere dorsally and duplicate	***T.smetsi* sp. n.**
99	Preapical tooth of left paramere laterally. Left paramere strongly curved	*** T. shangsiensis ***
–	Preapical tooth of left paramere dorsally, transformed into a filiform process that is rounded at apex. Left paramere nearly straight	*** T. gressitti ***
100	Dorsal lobe of right paramere very small	**101**
–	Dorsal lobe of right paramere large, nearly as long as ventral lobe	*** T. tonkinensis ***
101	Left paramere bent twice. Median lamina of phallobase in apical half distinctly narrowed	*** T. xichouensis ***
–	Left paramere nearly uniformly curved. Median lamina of phallobase in apical half not narrowed	***T.paratonkinensis* sp. n.**
102	Right paramere simple, not composed of two lobes	**103**
–	Right paramere composed of two lobes	**121**
103	Left paramere at base simple	**109**
–	Left paramere at base composed of two lobes	**104**
104	Right paramere at least subequal in length to phallobase, without apical hook ventrally	**105**
–	Right paramere approx. one third as long as phallobase, or shorter	**107**
–	Right paramere half as long as phallobase. Left paramere longer than phallobase	***T.koi* sp. n.**
105	Ventral distal spine of metatibia inserted at apex of metatibia and much shorter than the metatarsomere 1. Basal lobe of left paramere ¼ as long as ventral lobe	*** T. longipenis ***
–	Ventral distal spine of metatibia inserted shortly behind middle of ventral margin of metatibia and longer than the long metatarsomere 1	**106**
106	Basal lobe of left paramere half as long as ventral lobe	*** T. angkhangensis ***
–	Basal lobe of left paramere as long as ventral lobe	***T.giulianae* sp. n.**
107	Phallobase at right side not strongly produced. Right paramere with a small apical hook ventrally	***T.tanahrataensis* sp. n.**
–	Phallobase at right side strongly produced	**108**
108	Right paramere without small apical hook. Dorsal lobe of left paramere small	*** T. maerimensis ***
–	Right paramere with a small apical hook. Dorsal lobe of left paramere half as long as ventral lobe	***T.angkorthomensis* sp. n.**
109	Left paramere over its entire length not split, but simple	**112**
–	Left paramere split behind base into two long filiform branches	**110**
110	Left paramere split behind basal third. Right paramere short and curved	***T.dongnaiensis* sp. n.**
–	Left paramere split in apical half. Right paramere long and straight	**111**
111	Right paramere split into two narrow branches at apical quarter	*** T. sejugata ***
–	Right paramere split into two narrow branches at basal third	***T.allosejugata* sp. n.**
112	Left paramere more or less narrow, at middle distinctly narrower than apex of phallobase (lateral view)	**113**
–	Left paramere wide over its entire length, at middle nearly as wide as apex of phallobase (lateral view)	***T.fulleri* sp. n.**
113	Left paramere strongly bent at base	**117**
–	Left paramere straight at base	**114**
114	Left paramere straight in basal half distinctly wider than in apical half. Right paramere half as long as phallobase	***T.desalvazzai* sp. n.**
–	Left paramere straight in basal half only little wider than in apical half. Right paramere at maximum one third as long as phallobase	**115**
115	Median lamina of phallobase at apex curved dorsally. Basis of left paramere not extended ventrally	***T.angkorwatensis* sp. n.**
–	Median lamina of phallobase at apex straight. Basis of left paramere strongly extended ventrally	**116**
116	Right paramere much less than one third as long as left paramere	***T.pseudoruiliensis* sp. n.**
–	Right paramere one third as long as left paramere	*** T. gestroi ***
117	Left paramere bent downward basally. Median phallobasal lamina simply pointed	**118**
–	Left paramere bent upward basally. Median phallobasal lamina widened and sharply truncate at apex	***T.vari* sp. n.**
118	Median lamina of phallobase straight. Phallobase at right side not strongly produced	**119**
–	Median lamina of phallobase evenly curved ventrally. Phallobase at right side strongly produced	***T.infida* sp. n.**
119	Ventral terminal spine of metatibia slightly shorter than first metatarsomere	**120**
–	Ventral terminal spine of metatibia distinctly longer than first metatarsomere. Right paramere strongly bent at middle (lateral view)	***T.spinotibialis* sp. n.**
120	Right paramere moderately bent at middle (lateral view)	*** T. ruiliensis ***
–	Right paramere nearly straight (lateral view)	***T.semiruiliensis* sp. n.**
121	Left paramere simple	**123**
–	Left paramere composed of two lobes	**122**
122	Right side of phallobase strongly produced. Dorsal lobe of left paramere as long as ventral one	***T.soppongana* sp. n.**
–	Right side of phallobase not strongly produced. Dorsal lobe of left paramere short, distinctly shorter than ventral one	**129**
123	Dorsal lobe of right paramere wide, with sickle-shaped, large apical hook	*** T. tianchiensis ***
–	Dorsal lobe of right paramere narrow, evenly curved or straight, and sharply pointed	**124**
124	Left paramere filiform. Right paramere much less than half as long as left paramere	*** T. jinghongensis ***
–	Left paramere elongate and robust	**125**
125	Left paramere with a large triangular dorsomedian extension	**126**
–	Left paramere without large dorsomedian extension	**127**
126	Right paramere slightly more than half as long as left paramere. Split between lobes of right parameres deep, dorsal lobe as long as ventral lobe	***T.veliformis* sp. n.**
–	Right paramere half as long as left paramere. Dorsal lobe of right paramere distinctly shorter than ventral lobe	***T.umphangensis* sp. n.**
127	Right paramere a quarter as long as left paramere. Split between lobes of right parameres little deep, dorsal lobe reduced in size	*** T. latefemorata ***
–	Right paramere half as long as left paramere. Split between lobes of right parameres deep, dorsal lobe not reduced in size	**128**
128	Left paramere at middle narrow (lateral view), only little longer than median phallobasal lamina	***T.bolavensensis* sp. n.**
–	Left paramere at middle distinctly widened, distinctly longer than median phallobasal lamina	***T.nakaiensis* sp. n.**
129	Dorsal lobe of right paramere triangular and short, sharply pointed	*** T. menglongensis ***
–	Dorsal lobe of right paramere convexly widened and elongate	*** T. ruiliana ***

##### Descriptions

### Tetraserica finociliata sp. n.

Taxon classificationAnimaliaColeopteraMelolonthidae

http://zoobank.org/FD9F606D-0301-4790-B3A6-43FA7E59E440

Figures 1 , 47

#### Type material examined.

Holotype: ♂ “Myanmar (Burma) 21 km E Putao, H-550 m Nan S Bon vill., 1-5.5.98 leg S. Murzin & V. Sinaev/ Coll. D. Ahrens/ 215 Sericini Asia spec.” (ZFMK). Paratypes: 1 ♂ “Myanmar (Burma) 25 km E Putao, H-800 m Nan Sa Bon vill., 6-9.5.98 leg S. Murzin & V. Sinaev/ Coll. D. Ahrens” (ZFMK), 1 ♂ “Myanmar (Burma) 21 km S Putao, H-550 m Nan Sa Bon vill., 1-5.5.98 leg S. Murzin & V. Sinaev/ Coll. D. Ahrens” (ZFMK).

#### Description.

Length of body: 8.9 mm; length of elytra: 6.8 mm; maximum width: 5.4 mm. Surface of labroclypeus and disc of frons glabrous. Smooth area anterior to eye twice as wide as long. Eyes moderately large, ratio of diameter/interocular width: 0.67. Ratio of length of metepisternum/metacoxa: 1/1.53. Metatibia moderately long and wide, ratio width/length: 1/3.77; basal group of dorsal spines of metatibia at first third of metatibial length.

**Figure 1.**
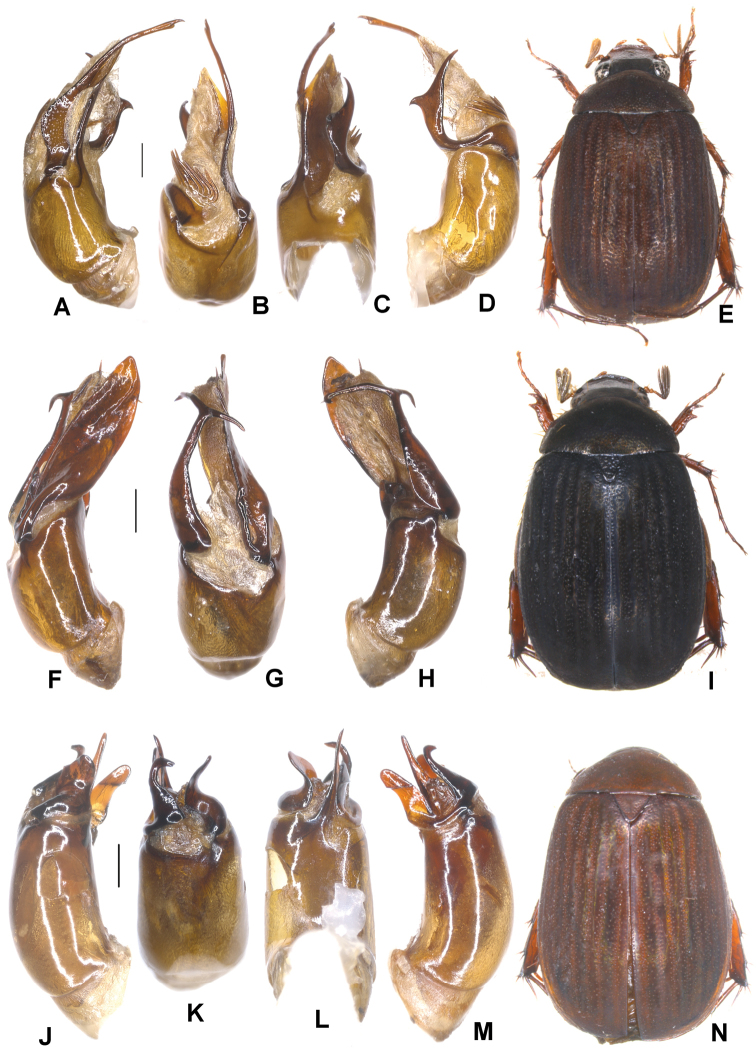
**A–E***Tetrasericafinociliata* sp. n. (holotype) **F–I***T.romae* sp. n. (holotype) **J–N***T.neouncinata* sp. n. (holotype) **A, F, J** aedeagus, left side lateral view **D, H, M** aedeagus, right side lateral view **B, G, K** parameres, dorsal view **C, L** parameres, ventral view **E, I, N** habitus. Scale bars: 0.5 mm. Habitus not to scale.

Aedeagus: Fig. 1A–D. Habitus: Fig. 1E.

Female unknown.

#### Variation.

Length of body: 8.9–10.2 mm; length of elytra: 6.8–7.1 mm; maximum width: 5.4–5.8 mm.

#### Diagnosis.

*Tetrasericafinociliata* sp. n. differs from all other *Tetraserica* species by the long and robust distal lobe of right paramere being strongly curved upwards (lateral view), and having a strong subapical ventral tooth.

#### Etymology.

The species name (adjective in the nominative singular) is derived from the combined Latin words *fino*- (fine) and *ciliatus* (ciliate), with reference to the fine comp of trichomes at the base of the right paramere.

### Tetraserica romae sp. n.

Taxon classificationAnimaliaColeopteraMelolonthidae

http://zoobank.org/39DC2D2E-F504-4252-8659-2DCF8231C784

Figures 1 , 51

#### Type material examined.

Holotype: ♂ “Vietnam-N (Na Hang) 160 km NW Ha Noi, NE env. of Na Hang 26.5-6.6.1996 150–200 m lg. A. Napolov & I. Roma/ CNa Riga” (ZFMK). Paratype: 1 ♂ “X-DA4501 labcode: VD 023 Vietnam: Tuyn Quang pr. SE-E env. of Na Hang, 200–700 m 22°17'30"–22'30"N, 105°26'–28'E, 1-12.v.2010 L. Dembický leg. (VN2/2010 MZM EXPEDITION) Tetraserica sp VI_V1” (ZFMK), 2 ♂♂ “Vietnam-N Tuyen quang pr. SE-E env. of Na Hang 22°17'30"–22'30"N, 105°26'–28'E, 200–700 m L. Dembicky leg., 1.-12.V.2010” (ZFMK).

#### Description.

Length of body: 7.3 mm; length of elytra: 5.3 mm; maximum width: 4.6 mm. Dorsal surface blackish, ventral surface reddish brown. Surface of labroclypeus and disc of frons glabrous. Smooth area anterior to eye twice as wide as long. Eyes small, ratio of diameter/interocular width: 0.46. Ratio of length of metepisternum/metacoxa: 1/1.65. Metatibia moderately long and wide, ratio width/length: 1/3.81; basal group of dorsal spines of metatibia at first third of metatibial length.

Aedeagus: Fig. 1F–H. Habitus: Fig. 1I.

Female unknown.

#### Variation.

Length of body: 7.3–8.1 mm; length of elytra: 5.3–5.8 mm; maximum width: 4.6–5.0 mm.

#### Diagnosis.

*Tetrasericaromae* sp. n. differs from all other *Tetraserica* species by the right hammer-shaped paramere, which has a sharply pointed double spine at apex, one directed dorsally, and a larger one ventrally.

#### Etymology.

The new species is named after one of its collectors, Ilona Roma (noun in genitive singular).

### Tetraserica neouncinata sp. n.

Taxon classificationAnimaliaColeopteraMelolonthidae

http://zoobank.org/98E20F14-4F65-430F-98F8-26589E6AC496

Figures 1 , 50

#### Type material examined.

Holotype: ♂ “NE-Laos: Houa Phan prov.; Ban Saleui, Phou Pan (Mt.)- 20°12'N, 104°01'E, 11.iv.-15.v. 2012, 1300–1900 m leg. C. Holzschuh - ZFMK Ankauf 2012” (ZFMK). Paratypes: 1 ♂ “X-DA4582/ X-DA4582 labcode VD049 Laos: Houa Phan prov.; Ban Saleui, Phou Pan (Mt.)- 20°12'N, 104°01'E, 15-30.iv.2014 leg. C. Holzschuh Ankauf 2014/ Tetraserica spLA_V22/ sp-LA-V22” (ZFMK), 1 ♂ “Laos-NE, Houa Phan prov., 20°12–13.5'N, 103°59.5’-104°01'E, Ban Saluei- Phou Pane Mt., 1340–1870 m, 10.v.-16.vi.2009, M. Brancucci & local coll. leg./ NHMB Basel, NMPC Prague Laos 2009 Expedition: M. Brancucci, M. Geiser, Z. Kraus, D. Hauck, V. Kubáň” (NHMB), 2 ♂♂ “NE-Laos: Houa Phan prov., 20°13'09–19"N, 103°59'54"–104°00'03"E, 1480–1510 m, Phou Pane Mt., 22.iv.- 14.v.2008, Vít Kubán leg.” (ZFMK), 1 ♂ “Myanmar, Mandalay prov. Kyaukpadanng 10.6.2009 From Li Jingke” (ZFMK).

#### Description.

Length of body: 8.8 mm; length of elytra: 7.9 mm; maximum width: 6.1 mm. Surface of labroclypeus and disc of frons glabrous. Smooth area anterior to eye twice as wide as long. Eyes small, ratio of diameter/interocular width: 0.55. Ratio of length of metepisternum/metacoxa: 1/1.76. Posterior margin of metafemur with blunt tooth. Metatibia moderately long and wide, ratio width/length: 1/3.31; basal group of dorsal spines of metatibia at first third of metatibial length.

Aedeagus: Fig. 1J–M. Habitus: 1N.

Female unknown.

#### Variation.

Length of body: 8.8–9.8 mm; length of elytra: 7.1–7.9 mm; maximum width: 5.8–6.1 mm.

#### Diagnosis.

The new species differs from the similar *T.daqingshanica* by the dorsal lobe of the right paramere being wide and straight in the basal half and curved in the apical half; the apex of left paramere gently narrows towards apex in *T.neouncinata*.

#### Etymology.

The species name (adjective in the nominative singular) is derived from the combined Greek word *neo*- (new) and Latin word *uncinatus*, with reference to the uncinated shape of the right paramere.

### Tetraserica pseudouncinata sp. n.

Taxon classificationAnimaliaColeopteraMelolonthidae

http://zoobank.org/83311389-FED9-4ECF-BAB2-A07C820F9644

Figures 2 , 50

#### Type material examined.

Holotype: ♂ “X-DA4693 labcode: VD086 China, Guangxi A.R., Schiwandashan National Forest Park (forested river valley; at light), 290–360 m, 21°54.4'N, 107°54.2'E, 5-9.iv.2013, M. Fikáček, J. Hájek, J. Růžička leg. Tetraserica spCH_V47/ sp-CH-V47/ X-DA4693“ (ZFMK).

#### Description.

Length of body: 9.3 mm; length of elytra: 6.9 mm; maximum width: 5.9 mm. Surface of labroclypeus and disc of frons glabrous. Smooth area anterior to eye twice as wide as long. Eyes moderately large, ratio of diameter/interocular width: 0.58. Ratio of length of metepisternum/metacoxa: 1/1.7. Posterior margin of metafemur with blunt tooth. Metatibia moderately long and wide, ratio width/length: 1/3.31; basal group of dorsal spines of metatibia at first third of metatibial length.

**Figure 2.**
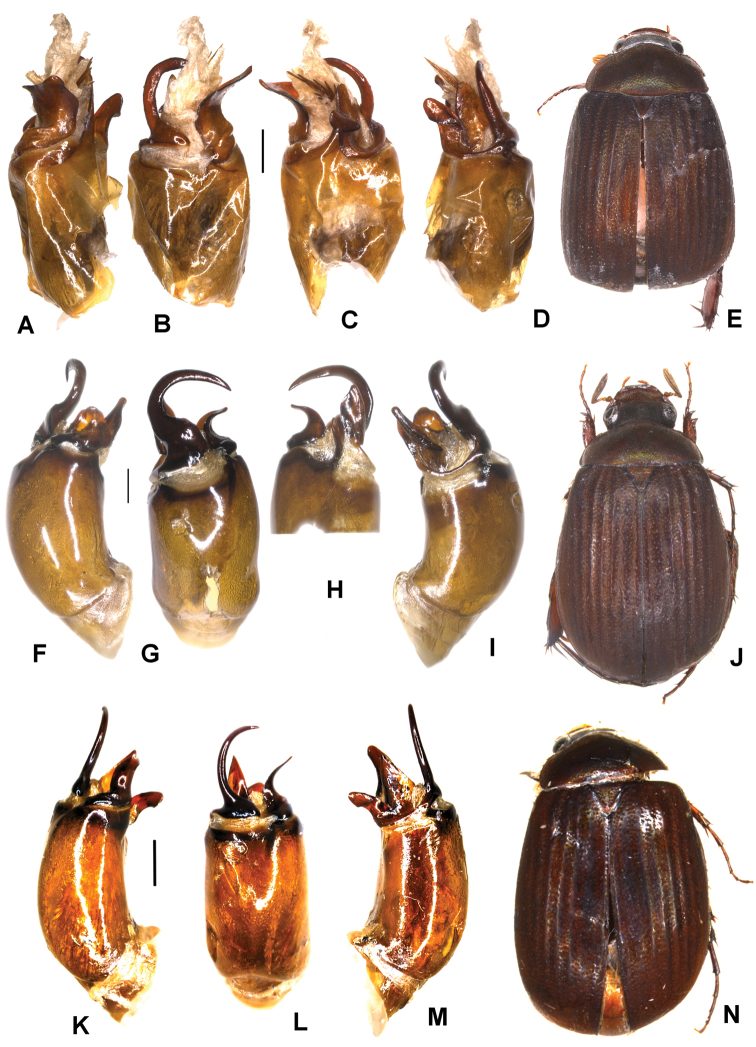
**A–E***Tetrasericapseudouncinata* sp. n. (holotype) **F–J***T.falciformis* sp. n. (holotype) **K–N***T.sculptilis* Liu et al., 2014 (holotype). **A, F, K** aedeagus, left side lateral view **D, I, M** aedeagus, right side lateral view **B, G, L** parameres, dorsal view **C, H** parameres, ventral view **E, J, N** habitus. Scale bars: 0.5 mm. Habitus not to scale.

Aedeagus: Fig. 2A–D. Habitus: Fig. 2E.

Female unknown.

#### Diagnosis.

*Tetrasericapseudouncinata* sp. n. differs from *T.neouncinata* by the left paramere being two thirds as wide as length of the antennal club (rather than one quarter as wide as in *T.neouncinata*).

#### Etymology.

The species name (adjective in the nominative singular) is derived from the combined Greek word *pseudo*- (false) and Latin word *uncinatus*, with reference to the uncinated shape of the right paramere.

### Tetraserica falciformis sp. n.

Taxon classificationAnimaliaColeopteraMelolonthidae

http://zoobank.org/4BC31299-C3A5-4981-8735-E4D6A2757300

Figures 2 , 48

#### Type material examined.

Holotype: ♂ “Laos, Champassak Prov. Dong Hua Xao NBCA 2 km S of Ban NongLuang, bank of Touay-Guai stream/ 15°4'N, 106°13'E, 800 m, at light, No. 24 1-5.IV.1998, leg. O. Merkl & G. Csorba/ 599 Sericini Asia spec.” (HNHM). Paratypes: 2 ♂♂ “Laos, Champassak Prov. Dong Hua Xao NBCA 2 km S of Ban NongLuang, bank of Touay-Guai stream/ 15°4'N, 106°13'E, 800 m, at light, No. 24 1-5.IV.1998, leg. O. Merkl & G. Csorba” (HNHM, ZFMK), 3 ♂♂ “Laos, Attapeau prov.; Annam Highlands Mts Dong Amphan; NBCA, ca. 1160 m NONG FA (crater lake) env.; 15°05.9'N, 107°25.6'E, St. Jakl lgt, 30.4.-6.5.2010” (NMPC, ZFMK), 1 ♂ “X-DA4499 labcode: VD022 Laos, Attapeu prov. Annam Highlands Mts. Dong Amphan NBCA, ca.1160 m NONG FA (crater lake) env. 15°05.9'N, 107°25.6'E, 30.iv-6.v.2010, Jiři Hájek leg. Tetraserica spLA_Annam1/ X-DA4499/ sp-LA-Annam1” (ZFMK), 1 ♂ “X-DA4498 labcode: VD021 Laos, Attapeu prov. Annam Highlands Mts. Dong Amphan NBCA, ca.1160 m NONG FA (crater lake) env. 15°05.9'N, 107°25.6'E, 30.iv-6.v.2010, Jiři Hájek leg. Tetraserica spLA_Annam1/ X-DA4498” (ZFMK).

#### Description.

Length of body: 11.8 mm; length of elytra: 7.5 mm; maximum width: 7 mm. Surface of labroclypeus and disc of frons glabrous. Smooth area anterior to eye twice as wide as long. Eyes small, ratio of diameter/interocular width: 0.53. Ratio of length of metepisternum/metacoxa: 1/1.58. Metatibia short and wide, ratio width/length: 1/3.21; basal group of dorsal spines of metatibia at first third of metatibial length.

Aedeagus: Fig. 2F–I. Habitus: Fig. 2J.

Female unknown.

#### Variation.

Length of body: 11.8–12.5 mm; length of elytra: 7.5–9.2 mm; maximum width: 6.4–7.0 mm.

#### Diagnosis.

*Tetrasericafalciformis* sp. n. differs in male genital shape from the similar *T.sculptilis* by having the posterior margin of metafemur straight rather than being blunt or with a tooth; furthermore, the dorsal lobe of the right paramere is markedly curved and strongly widened at base.

#### Etymology.

The species name (adjective in the nominative singular) is derived from the combined Latin words *falcis*- (sickle) and *formis* (of shape), with reference to the sickle-shaped right paramere.

### Tetraserica sculptilis

Taxon classificationAnimaliaColeopteraMelolonthidae

Liu, Fabrizi, Bai, Yang & Ahrens, 2014

Figures 2 , 56

Tetraserica sculptilis Liu, Fabrizi, Bai, Yang & Ahrens, 2014: 89, fig. 1E–H.

#### Material examined.

**Vietnam**: 1 ♂ “Vietnam: Vinh Phú: Tam Doa W. Edge of the town. 930 m 20–31 May 1996 B. Hubley R. Bain. ROM 961033/ UV light; scrub growth at 1° forest edge 21°27'N, 105°39'E” (HWML), 1 ♂ “Vietnam, Vinh Phú: Tam Dao Hill Stn. Telecommunications tower, Jan-May 1996 per B. Hubley, ROM 961024” (HWML).

Aedeagus: Fig. 2K–M. Habitus: Fig. 2N.

#### Remarks.

The species was known only from China (Hubei and Yunnan provinces); it is now recorded for first time from northern Vietnam.

### Tetraserica pseudoliangheensis sp. n.

Taxon classificationAnimaliaColeopteraMelolonthidae

http://zoobank.org/3DA098CC-2732-4296-A73A-B4F8F0604C23

Figures 3 , 50

#### Type material examined.

Holotype: ♂ “Laos centr. Khammouan prov. NAKAI env. 4-8.5.1998, Route No 8, alt 560±20 m, N17°42.8, E 105°08.9 (GPS) E. Jendek & O. Šausa leg./ Coll. P. Pacholátko/ 167 Sericini Asia spec.” (CPPB).

#### Description.

Length of body: 10.1 mm; length of elytra: 7.5 mm; maximum width: 6.3 mm. Surface of labroclypeus and disc of frons glabrous. Smooth area anterior to eye twice as wide as long. Eyes moderately large, ratio of diameter/interocular width: 0.57. Ratio of length of metepisternum/metacoxa: 1/1.58. Metatibia short and wide, ratio width/length: 1/2.89; basal group of dorsal spines of metatibia at first third of metatibial length.

**Figure 3.**
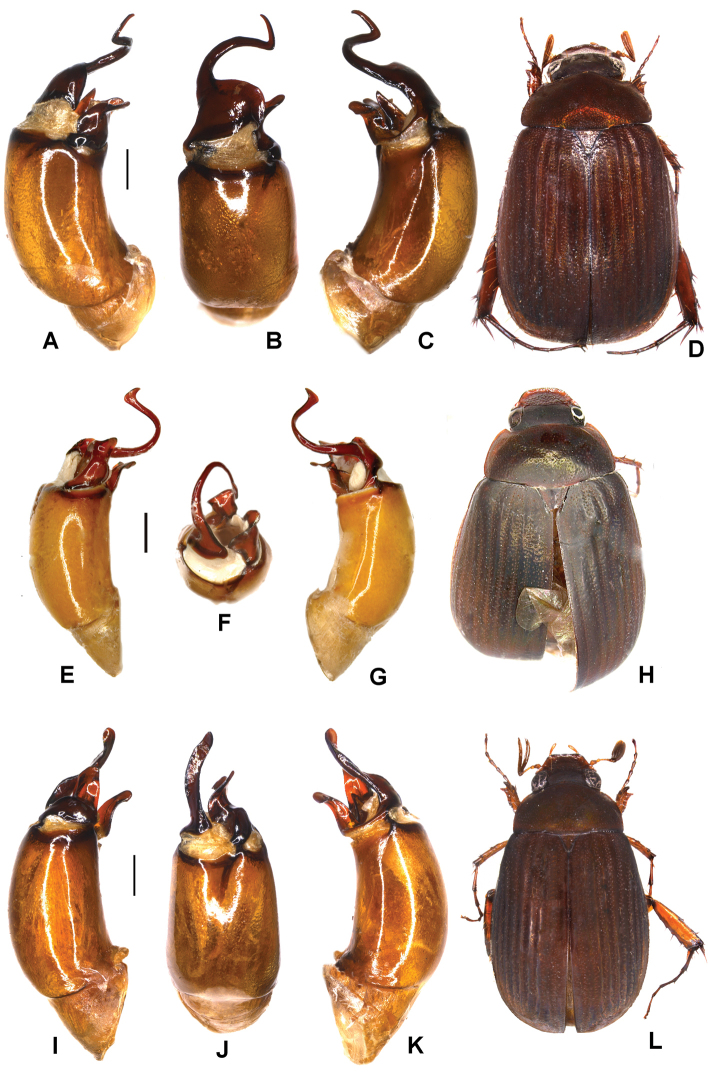
**A–D***Tetrasericapseudoliangheensis* sp. n. (holotype) **E–H***T.liangheensis* Liu et al., 2014 (holotype) **I–L**T.*T.doisuthepensis* sp. n. (holotype) **A, E, I** aedeagus, left side lateral view **C, G, K** aedeagus, right side lateral view **B, F, J** parameres, dorsal view **D, H, L** habitus. Scale bars: 0.5 mm. Habitus not to scale.

Aedeagus: Fig. 3A–C. Habitus: Fig. 3D.

Female unknown.

#### Diagnosis.

*Tetrasericapseudoliangheensis* sp. n. differs from *T.falciformis* by the dorsal lobe of right paramere having a sharp hook at its apex (dorsal view). The species is also rather similar to *T.liangheensis* Liu et al., 2014; however, in the new species the posterior margin of metafemur does not possess a tooth and the dorsal lobe of the right paramere is much wider at the base.

#### Etymology.

The species name (adjective in the nominative singular) is derived from the combined Latin words *pseudo*- (nearly) and the species name *liangheensis*, with reference to the similarity to *T.liangheensis* Liu et al., 2014.

### Tetraserica liangheensis

Taxon classificationAnimaliaColeopteraMelolonthidae

Liu, Fabrizi, Bai, Yang & Ahrens, 2014

Figures 3 , 51

Tetraserica liangheensis Liu, Fabrizi, Bai, Yang & Ahrens, 2014: 103, fig. 6A–D.

#### Material examined.

**Laos**: 13 ♂♂ “NE-Laos: Houa Phan prov.; Ban Saleui, Phou Pan (Mt.)- 20°12'N, 104°01'E, 11.iv.-15.v.2012, 1300–1900 m, leg. C. Holzschuh ZFMK Ankauf 2012” (ZFMK), 4 ♂♂ “NE-Laos: Houa Phan prov., 20°13'09–19"N, 103°59'54"–104°00'03"E, 1480–1510 m, Phou Pane Mt., 22.IV.- 14.V.2008, Vít Kubáň leg.” (ZFMK), 1 ♂ “NE-Laos: Houa Phan prov., 20°13'09–19"N, 103°59'54"–104°00'03"E, Phu Pane Mt., 1480–1510 m, 17.5.-3.6.2007, Vit Kubáň leg.” (ZFMK), 1 ♂ “Laos -NE: Houa Phan prov. 20°12.138'N, 104°00621'E, PHU PHAN Mt ~1750 m 17.5- 3.6.2007 Vit. Kubáň leg.” (ZFMK), 1 ♂ “Laos, 21°09'N, 101°19'E, Louangnamtha pr. Namtha-Muang Sing, 5-31.v.1997, 900–1200 m Vit. Kubáň leg/ coll. P. Pacholátko” (CPPB), 1 ♂ “X-DA3439a labcode VD006 Laos, Xieng Khouang, Phonsavan (30 km NE), Phou Sane Mt. 1400–1600 m 19°38'20"N, 103°20'20"E, 10-30.5.2009 Vit. Kubáň Tetraserica spLA1/ sp-LA-1” (ZFMK), 1 ♂ “Laos-NE, Houa Phan prov., 20°12–13.5'N, 103°59.5’-104°01'E, Ban Saluei- Phou Pane Mt., 1340–1870 m, 10.v.-16.vi.2009, M. Brancucci & local coll. leg./ NHMB Basel, NMPC Prague Laos 2009 Expedition: M. Brancucci, M. Geiser, Z. Kraus, D. Hauck, V. Kubáň” (NHMB), 9 ♂♂ “LAOS-NE, Xieng Khouang prov., 19°37–8'N, 103°20–1'E, 30 km NE Phonsavan Ban Na Lam -> Phou Sane Mt., 1300–1700 m, 10.-30.v.2009, M. Geiser leg./ NHMB Basel, NMPC Prague, Laos 2009, Expedition: M. Brancucci, M. Geiser, Z. Kraus, D. Hauck, V. Kubáň/ 914 Sericini Asia spec.” (NHMB), 7 ♂♂ “LAOS-NE, Xieng Khouang prov., 19°37–8'N, 103°20–1'E, Phonsavan (30 km NE) Phou Sane Mt., 1400–1700 m, 10.-30.v.2009, D. Hauck leg./ NHMB Basel, NMPC Prague, Laos 2009, Expedition: M. Brancucci, M. Geiser, Z. Kraus, D. Hauck, V. Kubáň/ 914 Sericini Asia spec.” (NHMB), 7 ♂♂ “LAOS-NE, Xieng Khouang prov., 19°37–8'N, 103°20'E, Phonsavan (30 km NE) Phou Sane Mt., 1420 m, 10-30.v.2009, Z. Kraus leg./ NHMB Basel, NMPC Prague, Laos 2009, Expedition: M. Brancucci, M. Geiser, Z. Kraus, D. Hauck, V. Kubáň/ 914 Sericini Asia spec.” (NHMB), 5 ♂♂ “LAOS-NE, Xieng Khouang prov., 19°37–8'N, 103°20'E, 30 km NE Phonsavan Ban Na Lam- > Phou Sane Mt., 1300–1500 m, 10.-30.v.2009, M. Brancucci leg./ NHMB Basel, NMPC Prague, Laos 2009, Expedition: M. Brancucci, M. Geiser, Z. Kraus, D. Hauck, V. Kubáň/ 914 Sericini Asia spec.” (NHMB), 1 ♂ “Laos-NE, Houa Phan prov., 20°12–13.5'N, 103°59.5’-104°01'E, Ban Saluei- Phou Pane Mt., 1340–1870 m, 10.v.-16.vi.2009, M. Brancucci & local coll. leg./ NHMB Basel, NMPC Prague Laos 2009 Expedition: M. Brancucci, M. Geiser, Z. Kraus, D. Hauck, V. Kubáň” (NHMB), 1 ♂ “Laos-NE, Houa Phan prov., 20°12–13.5'N, 103°59.5’-104°01'E, Ban Saleui- Phou Pane Mt. 1340–1870 m, 15.iv.-15.v.2008; Lao collectors leg./ 911 Sericini Asia spec.” (NHMB), 1 ♂ “X-DA3439b labcode VD007 Laos, Xieng Khouang, Phonsavan (30 km NE), Phou Sane Mt. 1400–1600 m 19°38'20"N, 103°20'20"E, 10-30.5.2009 Vit. Kubáň Tetraserica spLA1” (ZFMK), 1 ♂ “X-DA2443b labcode VD009 Laos, Xieng Khouang, Phonsavan (30 km NE), Phou Sane Mt. 1400–1600 m 19°38'20"N, 103°20'20"E, 10-30.5.2009 Vit. Kubáň Tetraserica spLA1” (ZFMK), 1 ♂ “X-DA2443a labcode VD008 Laos, Xieng Khouang, Phonsavan (30 km NE), Phou Sane Mt. 1400–1600 m 19°38'20"N, 103°20'20"E, 10-30.5.2009 Vit. Kubáň Tetraserica spLA1” (ZFMK), 1 ♂ “X-DA2409 labcode VD005 Laos, Xieng Khouang, Phonsavan (30 km NE), Phou Sane Mt. 1400–1600 m 19°38'20"N, 103°20'20"E, 10-30.5.2009 Vit. Kubáň Tetraserica spLA1” (ZFMK), 1 ♂ “X-DA4505 labcode VD027 Laos, Chiang Tung (Stupa) GH 5 km SE Muang Sing, 750 m, 26.03.-5.04.10 S. Murzin leg. Tetrasericaliangheensis” (ZFMK), 1 ♂ “X-DA4504 labcode VD026 Laos, Chiang Tung (Stupa) GH 5 km SE Muang Sing, 750 m, 26.03.-5.04.10 S. Murzin leg. Tetrasericaliangheensis” (ZFMK), 1 ♂ “X-DA4508 labcode VD029 Laos, Chiang Tung (Stupa) GH 5 km SE Muang Sing, 750 m, 26.03.-5.04.10 S. Murzin leg. Tetrasericaliangheensis” (ZFMK), 1 ♂ “Haut Mekong Muong Sing 18.IV.1918 R.V. de Salvaza.” (NHMUK), 1 ♂ “X-DA4509 labcode VD030 Laos, Chiang Tung (Stupa) GH 5 km SE Muang Sing, 750 m, 26.03.-5.04.10 S. Murzin leg. Tetrasericaliangheensis” (ZFMK), 1 ♂ “X-DA4513 labcode: VD033 LAOS Hua Phan prov.; Ban Saleui, Phou Pan (Mt) 20°12'N, 104°01'E, 3-5.iv.2013 leg. C. Holzschuh Tetraserica spLA_V6/ X-DA4513” (ZFMK), 1 ♂ “X-DA4562 labcode: VD042 LAOS Hua Phan prov.; Ban Saleui, Phou Pan (Mt) 20°12'N, 104°01'E, 3-5.iv.2013 leg. C. Holzschuh – ZFMK Ankauf 2014 Tetrasericaliangheensis/ X-DA45623” (ZFMK), 1 ♂ “X-DA4812 labcode VD101 Laos, Stupa GH, 5 km W Muang Sing, 750 m, 21.1482N 101.1711E, M. Murzin O. Shulga leg. Tetraserica spLA_V60” (ZFMK). **Thailand**: 1 ♂ “Thai N, Nan prov. 19°13'N, 101°7'E, Doi Phukha N.P. Headq. 22-26.iv.1999, ca 1500 m”, D. Hauck leg./ coll. Pacholátko” (CPPB), 1 ♂ “Thai N, Nan prov., Doi Phu Kha N.P Headq., 19°13'N, 101°7'E, 28.iv.-1.v.1999, M. Riha leg/ coll. P. Pacholátko/ 126 Sericini Asia spec.” (CPPB). **China**: 1 ♂ “China: S-Yunnan (Xishuangbanna) 37km NW Jinghong Guo Men Shan (NNNR)/ N22°17.91 E100°38.85 1080 m 26.V.2008 leg. A. Weigel LF” (NME).

Aedeagus: Fig. 3E–G. Habitus: Fig. 3H.

#### Remarks.

The species was known only from China (Yunnan province); it is now recorded for first time from Laos and Thailand.

### Tetraserica doisuthepensis sp. n.

Taxon classificationAnimaliaColeopteraMelolonthidae

http://zoobank.org/F86FCA75-863E-4B29-8EF4-9B3741AA2F94

Figures 3 , 48

#### Type material examined.

Holotype: ♂ “Thai 24-29-iv.1993 Doi Suthep Pacholátko & Dembický leg./ coll. P. Pacholátko/ 141 Sericini Asia spec.” (CPPB). Paratype: 1 ♂ “Laos, La Oudomxay 16.6.2005, ex coll. Sabatinelli” (ZFMK).

#### Description.

Length of body: 10.4 mm; length of elytra: 7.6 mm; maximum width: 6.6 mm. Surface of labroclypeus and disc of frons glabrous. Smooth area anterior to eye twice as wide as long. Eyes moderately large, ratio of diameter/interocular width: 0.56. Ratio of length of metepisternum/metacoxa: 1/1.63. Posterior margin of metafemur with sharp hook. Metatibia moderately long and wide, ratio width/length: 1/4.14; basal group of dorsal spines of metatibia at first third of metatibial length.

Aedeagus: Fig. 3I–K. Habitus: Fig. 3L.

Female unknown.

#### Variation.

Length of body: 10.1–10.4 mm; length of elytra: 7.4–7.6 mm; maximum width: 5.5–6.6 mm.

#### Diagnosis.

*Tetrasericadoisuthepensis* sp. n. differs from *T.liangheensis* Liu et al., 2014 by the dorsal lobe of right paramere being weakly curved, barely exceeding the ventral lobe of right paramere, and in having no additional hook at the apex. The new species differs from *T.sculptilis* by the stouter left paramere having a bluntly angulate dorsal margin.

#### Etymology.

The new species is named after the type locality, Doi Suthep (adjective in the nominative singular).

### Tetraserica trilobiforceps sp. n.

Taxon classificationAnimaliaColeopteraMelolonthidae

http://zoobank.org/8B06FEB0-DBD9-440E-8416-94C1F3588FD2

Figures 4 , 51

#### Type material examined.

Holotype: ♂ “Laos, 21°09'N, 101°19'E, Louangnamtha pr. Namtha-MuangSing 5-31.v.1997. 900–1200 m, Vit Kubáň leg/ coll. Pacholátko/ 232 Sericini Asia spec.” (CPPB). Paratypes: 1 ♂ “Laos, 21°09'N, 101°19'E, Louangnamtha pr. Namtha-MuangSing 5-31.v.1997. 900–1200 m, Vit Kubáň leg/ coll. Pacholátko/ 232 Sericini Asia spec.” (CPPB), 1 ♂ “LAOS north, 5-11.v.1997, 20 km NW Louang Namtha, N21°09.2 E 101°18.7, alt. 900±100 m, M. Štrba & R. Hergovits leg./ coll. P. Pacholátko/ 186 Sericini Asia spec.” (ZFMK).

#### Description.

Length of body: 8.3 mm; length of elytra: 5.8 mm; maximum width: 4.5 mm. Surface of labroclypeus and disc of frons glabrous. Smooth area anterior to eye twice as wide as long. Eyes moderately large, ratio of diameter/interocular width: 0.63. Ratio of length of metepisternum/metacoxa: 1/1.64. Metatibia moderately long and wide, ratio width/length: 1/3.31; basal group of dorsal spines of metatibia at first third of metatibial length.

**Figure 4.**
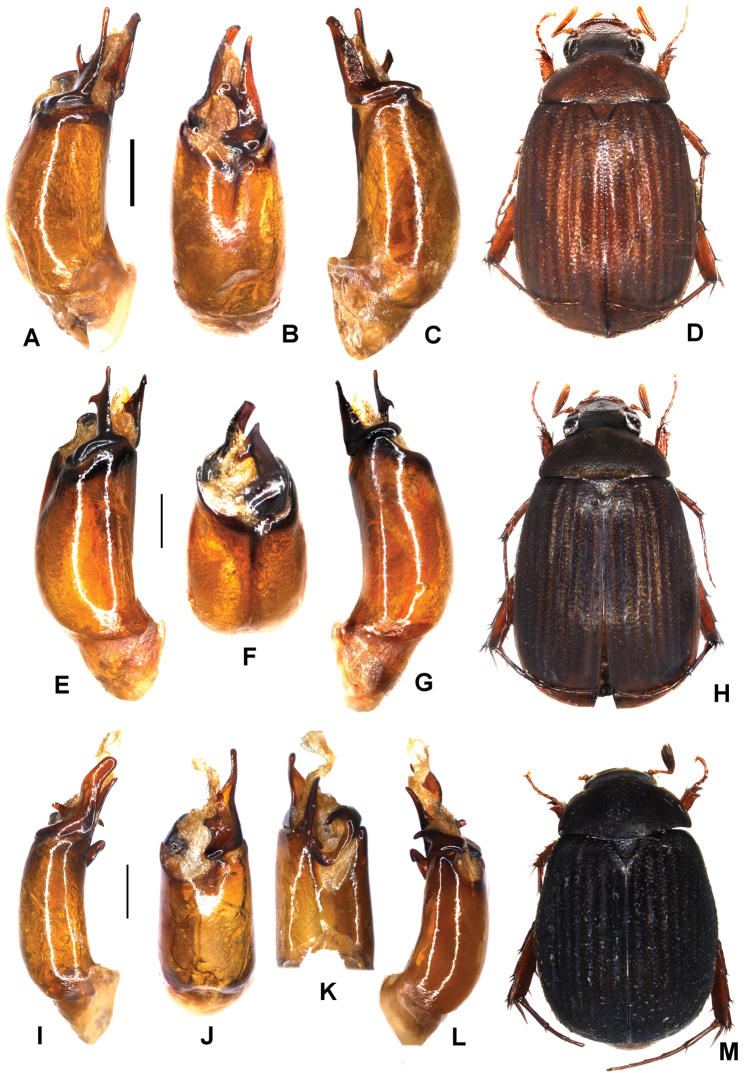
**A–D***Tetrasericatrilobiforceps* sp. n. (holotype) **E–H***T.phukradungensis* sp. n. (holotype) **I–M***T.cucphongensis* sp. n. (holotype) **A, E, I** aedeagus, left side lateral view **C, G, L** aedeagus, right side lateral view **B, F, J** parameres, dorsal view **K** parameres, ventral view **D, H, M** habitus. Scale bars: 0.5 mm. Habitus not to scale.

Aedeagus: Fig. 4A–C. Habitus: 4D.

Female unknown.

#### Variation.

Length of body: 8.3–8.9 mm; length of elytra: 5.8–6.2 mm; maximum width: 4.5–5.2 mm.

#### Diagnosis.

*Tetrasericatrilobiforceps* sp. n. differs from all other *Tetraserica* species by the left paramere having a dorsal lobe distant from base.

#### Etymology.

The species name (noun in apposition) is derived from the combined Latin words *tri*- (three times), *lobatus* (lobed), and *forceps*, with reference to the shape of the left paramere.

### Tetraserica phukradungensis sp. n.

Taxon classificationAnimaliaColeopteraMelolonthidae

http://zoobank.org/C481250B-84B9-4834-B7E0-B60CF6D03011

Figures 4 , 51

#### Type material examined.

Holotype: ♂ „THAI-NE, Loei prov., Phu Kradung N.P., 16°52'N, 101°49‘E, 16-18.v.1999, 1000 m, D. Hauck leg./ coll. P. Pacholátko” (CPPB).

#### Description.

Length of body: 9.1 mm; length of elytra: 6.9 mm; maximum width: 5.6 mm. Surface of labroclypeus and disc of frons glabrous. Smooth area anterior to eye twice as wide as long. Eyes moderately large, ratio of diameter/interocular width: 0.68. Ratio of length of metepisternum/metacoxa: 1/1.63. Metatibia moderately long and wide, ratio width/length: 1/3.36; basal group of dorsal spines of metatibia at first third of metatibial length.

Aedeagus: Fig. 4E–G. Habitus: Fig. 4H.

Female unknown.

#### Diagnosis.

*Tetrasericaphukradungensis* sp. n. differs from *T.trilobiforceps* sp. n. species by the shorter and stouter left paramere which has a short fine dorsal lobe behind the middle (rather than before the middle as in *T.trilobiforceps*).

#### Etymology.

The new species is named after the type locality, Phu Kradung (adjective in the nominative singular).

### Tetraserica cucphongensis sp. n.

Taxon classificationAnimaliaColeopteraMelolonthidae

http://zoobank.org/A25F69F4-F4AD-492B-97D2-0B43C981E028

Figures 4 , 46

#### Type material examined.

Holotype: ♂ “N-Vietnam, Cuc Phuong Nat. Park. 21-22.v.1996 Pacholátko & Dembický leg./ coll. P. Pacholátko/ 937 Sericini Asia spec.” (CPPB).

#### Description.

Length of body: 7.3 mm; length of elytra: 5.5 mm; maximum width: 4.9 mm. Dorsal surface blackish, ventral surface reddish brown. Surface of labroclypeus and disc of frons glabrous. Smooth area anterior to eye twice as wide as long. Eyes small, ratio of diameter/interocular width: 0.48. Ratio of length of metepisternum/metacoxa: 1/1.67. Metatibia moderately long and wide, ratio width/length: 1/3.33; basal group of dorsal spines of metatibia at first third of metatibial length.

Aedeagus: Fig. 4I–L. Habitus: Fig. 4M.

Female unknown.

#### Diagnosis.

This species differs from all other *Tetraserica* species in having a simple left paramere, by the strongly asymmetric phallobase (dorsal view), the very short median lamina of phallobase, and by having the right paramere more or less straight (ventral view).

#### Etymology.

The new species is named after the type locality, Cuc Phuong (adjective in the nominative singular).

### Tetraserica geiserae sp. n.

Taxon classificationAnimaliaColeopteraMelolonthidae

http://zoobank.org/7429EB1F-D04A-4AC9-9722-9CA7A5A83B5D

Figures 5 , 47

#### Type material examined.

Holotype: ♂ “Tonkin occ. Rég. de Hoa Binh R.P.A. de Cooman 1918/ 1006 Asia Sericini spec.” (MNHN). Paratypes: 3 ♂♂ “Tonkin occ. Rég. de Hoa Binh R.P.A. de Cooman 1918” (MNHN, ZFMK).

#### Description.

Length of body: 7 mm; length of elytra: 4.9 mm; maximum width: 4.3 mm. Dorsal surface blackish, ventral surface reddish brown. Surface of labroclypeus and disc of frons both glabrous. Smooth area anterior to eye twice as wide as long. Eyes small, ratio of diameter/interocular width: 0.46. Ratio of length of metepisternum/metacoxa: 1/1.81. Metatibia short and wide, ratio width/length: 1/3.27; basal group of dorsal spines of metatibia at first third of metatibial length.

**Figure 5.**
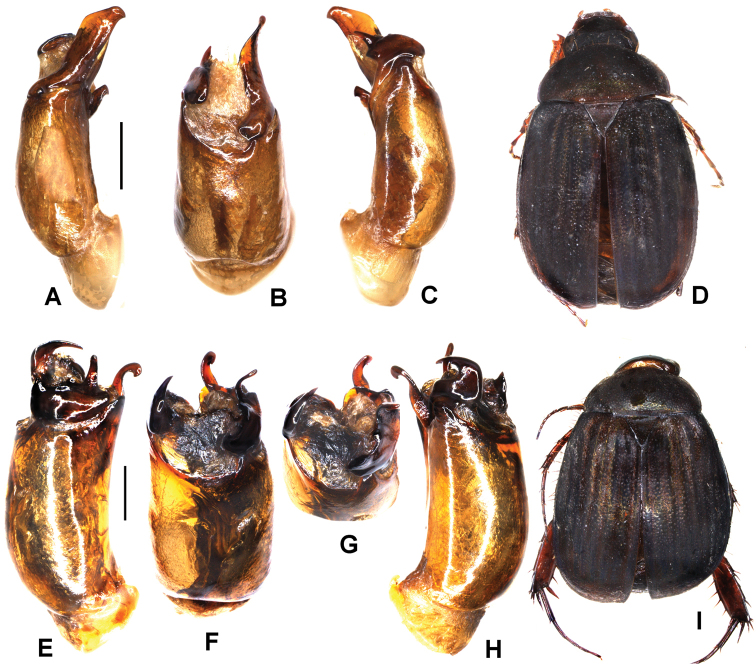
**A–D***Tetrasericageiserae* sp. n. (holotype) **E–I***T.thainguyensis* sp. n. (holotype) **A, E** aedeagus, left side lateral view **C, H** aedeagus, right side lateral view **B, F** parameres, dorsal view **G** parameres distal view **D, I** habitus. Scale bars: 0.5 mm. Habitus not to scale.

Aedeagus: Fig. 5A–C. Habitus: Fig. 5D.

Female unknown.

#### Variation.

Length of body: 7.0–7.1 mm; length of elytra: 4.9–6.2 mm; maximum width: 4.3–5.2 mm.

#### Diagnosis.

*Tetrasericageiserae* sp. n. differs from *T.cucphongensis* in lacking the dorsal subbasal lobe of the left paramere.

#### Etymology.

The new species is named in honour of Dr Geiser, Silvia’s oncologist, for all her efforts and care (noun in genitive singular).

### Tetraserica thainguyensis sp. n.

Taxon classificationAnimaliaColeopteraMelolonthidae

http://zoobank.org/05FB20E2-38C8-477D-A1EC-163DA2209D30

Figures 5 , 53

#### Type material examined.

Holotype: ♂ “N. Vietnam: 40 km NE Thainguyen, 300 m, 8.V.1963, leg. O. Kabakov/ 1009 Asia Sericini spec.” (ZIN).

#### Description.

Length of body: 7.6 mm; length of elytra: 7.1 mm; maximum width: 6.3 mm. Surface of labroclypeus and disc of frons glabrous. Smooth area anterior to eye twice as wide as long. Eyes moderately large, ratio of diameter/interocular width: 0.56. Ratio of length of metepisternum/metacoxa: 1/1.74. Metatibia short and wide, ratio width/length: 1/2.32; basal group of dorsal spines of metatibia at first third of metatibial length.

Aedeagus: Fig. 5E–H. Habitus: Fig. 5I.

Female unknown.

#### Diagnosis.

*Tetrasericathainguyensis* sp. n. differs from the similar *T.geiserae* by having the left paramere very short and split into two lobes.

#### Etymology.

The new species is named with reference to its occurrence close to Thainguyen (adjective in the nominative singular).

### Tetraserica xiengkhouangensis sp. n.

Taxon classificationAnimaliaColeopteraMelolonthidae

http://zoobank.org/CF5C626E-3D40-4FEA-8B3D-98CBD006C399

Figures 6 , 51

#### Type material examined.

Holotype: ♂ “LAOS-NE, Xieng Khouang prov., 19°37–8'N, 103°20'E, 30km NE Phonsavan Ban Na Lam- > Phou Sane Mt., 1300–1500 m, 10.-30.v.2009, M. Brancucci leg./ NHMB Basel, NMPC Prague, Laos 2009, Expedition: M. Brancucci, M. Geiser, Z. Kraus, D. Hauck, V. Kubáň/ 914 Sericini Asia spec.” (NHMB). Paratypes: 1 ♂ “LAOS-NE, Xieng Khouang prov., 19°37–8'N, 103°20'E, Phonsavan (30km NE) Phou Sane Mt., 1420 m, 10-30.v.2009, Z. Kraus leg./ NHMB Basel, NMPC Prague, Laos 2009, Expedition: M. Brancucci, M. Geiser, Z. Kraus, D. Hauck, V. Kubáň/ 914 Sericini Asia spec.” (ZFMK).

#### Description.

Length of body: 8.6 mm; length of elytra: 6.5 mm; maximum width: 5.3 mm. Surface of labroclypeus and disc of frons glabrous. Smooth area anterior to eye twice as wide as long. Eyes moderately large, ratio of diameter/interocular width: 0.68. Ratio of length of metepisternum/metacoxa: 1/1.73. Metatibia moderately long and wide, ratio width/length: 1/3.62; basal group of dorsal spines of metatibia at first third of metatibial length.

**Figure 6.**
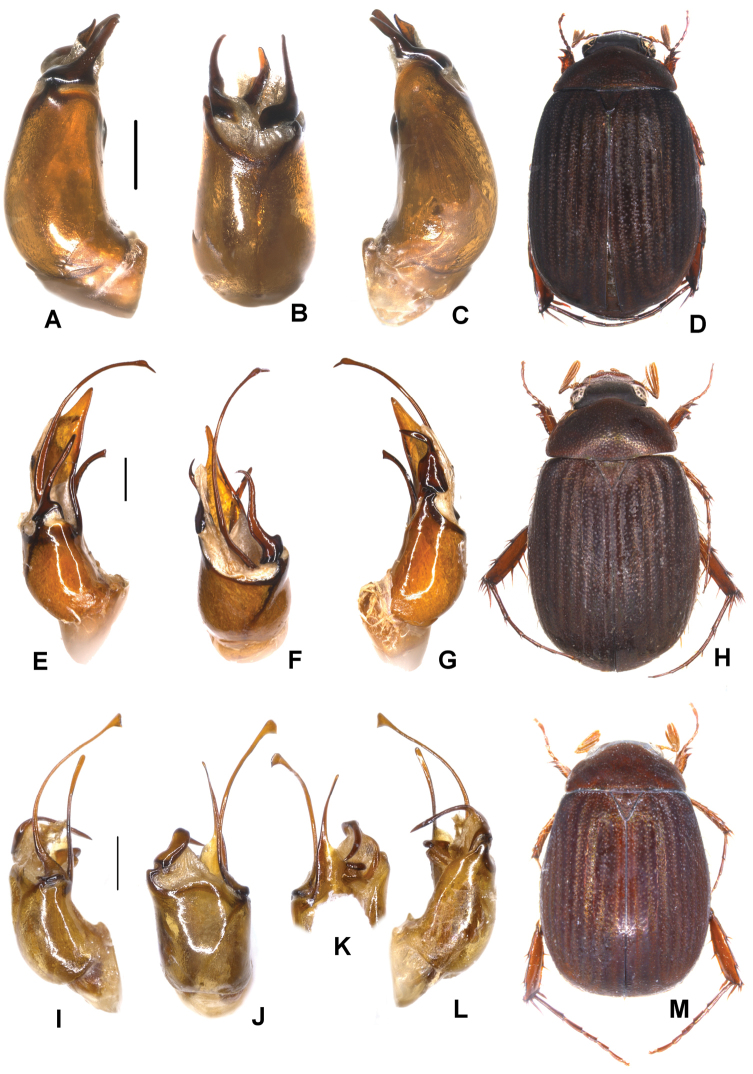
**A–D***Tetrasericaxiengkhouangensis* sp. n. (holotype) **E–H***T.doisangensis* Kobayashi, 2017 (Thailand: Doi Inthanon) **I–M***T.curviforceps* sp. n. (holotype) **A, E, I** aedeagus, left side lateral view **C, G, L** aedeagus, right side lateral view **B, F, J** parameres, dorsal view **K** parameres, ventral view **D, H, M** habitus. Scale bars: 0.5 mm. Habitus not to scale.

Aedeagus: Fig. 6A–C. Habitus: Fig. 6D.

Female unknown.

#### Variation.

Length of body: 8.6–8.7 mm; length of elytra: 6.3–6.5 mm; maximum width: 5.2–5.3 mm.

#### Diagnosis.

*Tetrasericaxiengkhouangensis* sp. n. differs from the similar *T.damaidiensis* by having the right paramere distinctly not wider (dorsal view) than the left one, and the left paramere short, one third as long as phallobase.

#### Etymology.

The new species is named with reference to its occurrence in the Xieng Khouang province (adjective in the nominative singular).

### Tetraserica doisangensis

Taxon classificationAnimaliaColeopteraMelolonthidae

Kobayashi, 2017

Figures 6 , 48

Tetraserica doisangensis Kobayashi, 2017: 38, figs 4, 13.

#### Material examined.

1 ♂ “Ban Angkhai alt. 750 m Samoeng Distr. Chiangmai pref. Thailand 15-20-V-1998 K. Masumoto leg.” (ZFMK), 1 ♂ “X-DA 4639 labcode VD079 THAILAND, Chiang Mai Doi Inthanon NP Vachirathan Fall 700 m Malaise Trap, 18°32.31'N, 98°36.048'E, 9-16.iii.2007, Y. Areeluck leg. T1808, Tetraserica spTH_V39/ X-DA4639/ sp-TH-V39” (QSBG), 1 ♂ “NW Thailand 9-16.V. 1991 MAE HONG SON Ban Huai Po 1600 m leg. P. Pacholátko/ coll. P. Pacholátko/ TS67/ 157 Sericini Asia spec.” (CPPB), 1 ♂ “N THAILAND: Angkhai village, Samoeng Dist., Chiang Mai Prov., 9-11.v.1999 K. Masumoto leg.” (ZFMK), 1 ♂ “X-DA 4640 labcode VD073 THAILAND, Chiang Mai Doi Inthanon NP Vachirathan Fall 700 m Malaise Trap, 18°32.31'N, 98°36.048'E, 9-16.iii.2007, Y. Areeluck leg. T1808, Tetraserica spTH_V39/ X-DA4639” (ZFMK), 1 ♂ “N-Thailand Ban Mai [Huai] Po 9.-16.5.1991 L. Horak lgt./ Coll. Milan Nikodym Praha” (ZFMK), 1 ♂ “Chiang Mai N. Thailand V 1985 N. Koyama leg./ Ex coll. Takeshi Matsumoto (formerly Itoh) via coll. D. Ahrens” (ZFMK), 1 ♂ “NW Thailand, 19.19N, 97.59E, Mae Hong Son, 1991 Ban Huai Po, 1600–2000 m 17.-23.5., L. Dembický leg.” (NHMW).

Aedeagus: Fig. 6E–G. Habitus: Fig. 6H.

### Tetraserica curviforceps sp. n.

Taxon classificationAnimaliaColeopteraMelolonthidae

http://zoobank.org/4E654C98-F2C6-44C0-AE04-9757BBE26D5A

Figures 6 , 47

#### Type material examined.

Holotype: ♂ “NE-Laos: Houa Phan prov., 20°13'09–19"N, 103°59'54"–104°00'03"E, 1480–1510 m, Phou Pane Mt., 22.iv.- 14.v.2008, Vít Kubán leg./ sp51” (ZFMK). Paratypes: 6 ♂♂ “NE-Laos: Houa Phan prov., 20°13'09–19"N, 103°59'54"–104°00'03"E, 1480–1510 m, Phou Pane Mt., 22.iv.- 14.v.2008, Vít Kubáň leg.” (ZFMK), 4 ♂♂, 3 ♀♀ “NE-Laos: Houa Phan prov., 20°13'09–19"N, 103°59'54"–104°00'03"E, 1480–1510 m, Phou Pane Mt., 22.IV.- 14.V.2008, Vít Kubáň leg.” (ZFMK), 7 ♂♂ “NE-Laos: Houa Phan prov., 20°13'09–19"N, 103°59'54"–104°00'03"E, 1480–1510 m, Phou Pane Mt., 22.4.- 14.5.2008, Vít Kubáň leg.” (ZFMK), 1 ♂ “NE-Laos: Houa Phan prov.; Ban Saleui, Phou Pan (Mt.)- 20°12'N, 104°01'E, 11.iv.-15.v.2012, 1300–1900 m, leg. C. Holzschuh ZFMK Ankauf 2012/13” (ZFMK), 1 ♂ “X-DA4511 labcode: VD031 LAOS: Houa Phan prov.; Ban Saleui, Phou Pan (Mt.) 20°12'N, 104°01'E, 3-5.v.2013, leg. C. Holzschuh Tetraserica spLA_V17/ X-DA4511” (ZFMK), 1 ♂ “X-DA4566 labcode: VD044 LAOS: Houa Phan prov.; Ban Saleui, Phou Pan (Mt.) 20°12'N, 104°01'E, 15-30.iv.2014, leg. C. Holzschuh - ZFMK Ankauf 2014 Tetraserica spLA_V17/ X-DA4566” (ZFMK), 1 ♂ “X-DA4570 labcode: VD045 LAOS: Houa Phan prov.; Ban Saleui, Phou Pan (Mt.) 20°12'N, 104°01'E, 15-30.iv.2014, leg. C. Holzschuh - ZFMK Ankauf 2014 Tetraserica spLA_V17/ X-DA4570/ sp-LA-V17” (ZFMK), 1 ♂ “X-DA4577 labcode: VD047 LAOS: Houa Phan prov.; Ban Saleui, Phou Pan (Mt.) 20°12'N, 104°01'E, 15-30.iv.2014, leg. C. Holzschuh - ZFMK Ankauf 2014 Tetraserica spLA_V17/ X-DA4577/ sp-LA-V17” (ZFMK), 1 ♂ “X-DA4583 labcode: VD050 LAOS: Houa Phan prov.; Ban Saleui, Phou Pan (Mt.) 20°12'N, 104°01'E, 15-30.iv.2014, leg. C. Holzschuh - ZFMK Ankauf 2014 Tetraserica spLA_V17/ X-DA4583” (ZFMK), 1 ♂ “X-DA4588 labcode: VD052 LAOS: Houa Phan prov.; Ban Saleui, Phou Pan (Mt.) 20°12'N, 104°01'E, 1.-2.v.2014, leg. C. Holzschuh - ZFMK Ankauf 2014 Tetraserica spLA_V17/ X-DA4588” (ZFMK), 1 ♂ “X-DA4593 labcode: VD055 LAOS: Houa Phan prov.; Ban Saleui, Phou Pan (Mt.) 20°12'N, 104°01'E, 6-14.iv.2014, leg. C. Holzschuh - ZFMK Ankauf 2014 Tetraserica spLA_V17/ X-DA4570/ sp-LA-V17” (ZFMK), 5 ♂♂ “Laos-NE, Houa Phan prov., 20°12–13.5'N, 103°59.5’-104°01'E, Ban Saleui-> Phou Pane Mt. 1340–1870 m, 15.iv.-15.v.2008; Lao collectors leg.” (NHMB), 1 ♂ “Laos-NE, Houa Phan prov., 20°12–13.5'N, 103°59.5'–104°01'E, Ban Saluei- Phou Pane Mt., 1340–1870 m, 10.v.-16.vi.2009, M. Brancucci & local coll. leg./ NHMB Basel, NMPC Prague Laos 2009 Expedition: M. Brancucci, M. Geiser, Z. Kraus, D. Hauck, V. Kubáň” (NHMB).

#### Description.

Length of body: 6.5 mm; length of elytra: 5.4 mm; maximum width: 4.4 mm. Surface of labroclypeus and disc of frons glabrous. Smooth area anterior to eye twice as wide as long. Eyes moderately large, ratio of diameter/interocular width: 0.56. Ratio of length of metepisternum/metacoxa: 1/1.52. Metatibia moderately long and wide, ratio width/length: 1/3.45; basal group of dorsal spines of metatibia at first third of metatibial length.

Aedeagus: Fig. 6I–L. Habitus: Fig. 6M.

Female unknown.

#### Variation.

Length of body: 6.5–7.8 mm; length of elytra: 5.4–6.0 mm; maximum width: 4.4–4.8 mm.

#### Diagnosis.

*Tetrasericacurviforceps* sp. n. differs from *T.sigulianshanica* Liu et al., 2014 by the distinctly finer and longer parameres. From *T.doisangensis* Kobayashi, 2017 it differs by the narrow median phallobasal apophysis and the strongly curved dorsal lobe of the right paramere.

#### Etymology.

The species name (noun in apposition) is derived from the combined Latin words *curvus* (curved) and *forceps*, with reference to the shape of the curved right paramere.

### Tetraserica miniatula

Taxon classificationAnimaliaColeopteraMelolonthidae

(Moser, 1915)

Figures 7 , 56

Neoserica miniatula Moser, 1915: 171.
Tetraserica miniatula : Ahrens and Fabrizi 2016: 125.

#### Type material examined.

Lectotype (here designated): ♂ “Pegu India/ Neosericaminiatula Type ♂ Moser” (ZMHB). Paralectotype: 1 ♀ “Pegu India/ Neosericaminiatula Type ♀ Moser” (ZMHB).

**Redescription.** Length of body: 8.0 mm; length of elytra: 5.8 mm; maximum width: 4.8 mm. Surface of labroclypeus and disc of frons glabrous. Smooth area anterior to eye twice as wide as long. Eyes large, ratio of diameter/interocular width: 0.72. Ratio of length of metepisternum/metacoxa: 1/1.62. Metatibia moderately long and wide, ratio width/length: 1/3.07; basal group of dorsal spines of metatibia at first third of metatibial length.

Female: Antennal club with three antennomeres, as long as remaining antennomeres combined. Eyes smaller than in male, ratio of diameter/interocular width: 0.64. Pygidium flat.

**Figure 7.**
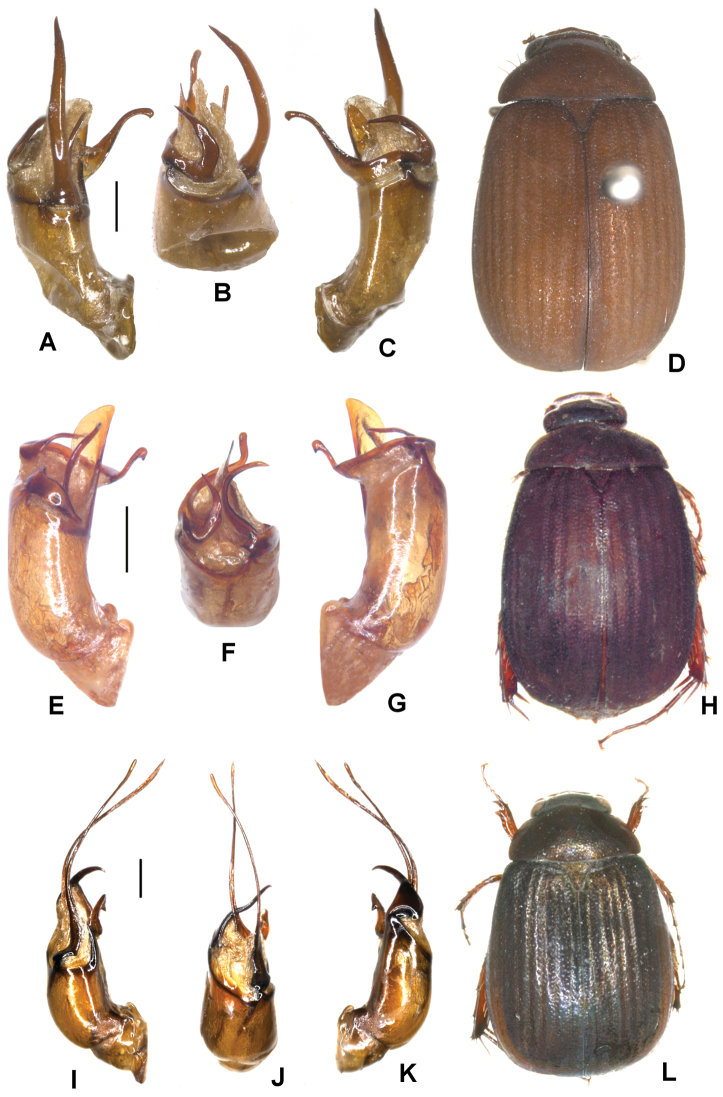
**A–D***Tetrasericaminiatula* (Moser, 1915) (syntype) **E–H***T.univestris* Ahrens & Fabrizi, 2016 sp. n. (holotype) **I–L***T.jinghongensis* Liu et al., 2014 (holotype) **A, E, I** aedeagus, left side lateral view **C, G, K** aedeagus, right side lateral view **B, F, J** parameres, dorsal view **D, H, L** habitus. Scale bars: 0.5 mm. Habitus not to scale.

Aedeagus: Fig. 7A–C. Habitus: Fig. 7D.

### Tetraserica univestris

Taxon classificationAnimaliaColeopteraMelolonthidae

Ahrens & Fabrizi, 2016

Figures 7 , 51

Tetraserica univestris Ahrens & Fabrizi, 2016: 128, figs 10Q–S, 31G.

#### Material examined.

1 ♂ “INDIA: Kolasib, Mizoram; 24°13'N, 92°40'E, K. Sreedevi

25.iv.2014” (NBAIR).

#### Remarks.

The species was described from Assam (India). This is the first record from Mizoram state (India).

Aedeagus: Fig. 7E–G. Habitus: Fig. 7H.

### Tetraserica jinghongensis

Taxon classificationAnimaliaColeopteraMelolonthidae

Liu, Fabrizi, Bai, Yang & Ahrens, 2014

Figures 7 , 50

Tetraserica jinghongensis Liu, Fabrizi, Bai, Yang & Ahrens, 2014: 100, fig. 5A–D.

#### Material examined.

4 ex. “China: S-Yunnan (Xishuangbanna) 37 km NW Jinghong Guo Men Shan (NNNR)/ N22°17.91 E100°38.85 1080 m 26.V.2008 leg. A. Weigel LF” (NME).

Aedeagus: Fig. 7I–K. Habitus: Fig. 7L.

#### Remarks.

This species has been recorded only from China (Yunnan Province) and Thailand (Kobayashi 2017).

### Tetraserica bachmaensis sp. n.

Taxon classificationAnimaliaColeopteraMelolonthidae

http://zoobank.org/4A17C685-256C-45E3-8B12-25BFBF093903

Figures 8 , 46

#### Type material examined.

Holotype: ♂ “X-DA4699 labcode: VD088, VIETNAM Tua Thien Hue Prov. Bach Ma Nat. Park, surr. Hotel Morin (1350–1400 m), 23-28.V.2014, legit L. Bartolozzi, G. Chelazzi, A. Bandinelli, S. Banbi, F. Fabiano, (n° Magazz. 2978) Tetraserica sp VI_V49/ sp VI-V49/ 935 Sericini Asia spec.” (VNMN).

#### Description.

Length of body: 10.1 mm; length of elytra: 7.6 mm; maximum width: 6.9 mm. Dorsal surface blackish, ventral surface reddish brown. Surface of labroclypeus and disc of frons both glabrous. Smooth area anterior to eye twice as wide as long. Eyes moderately large, ratio of diameter/interocular width: 0.56. Ratio of length of metepisternum/metacoxa: 1/1.56. Metatibia short and wide, ratio width/length: 1/2.68; basal group of dorsal spines of metatibia at first third of metatibial length. Ventral metatibial spine extremely elongated, distinctly exceeding first metatarsomere.

**Figure 8.**
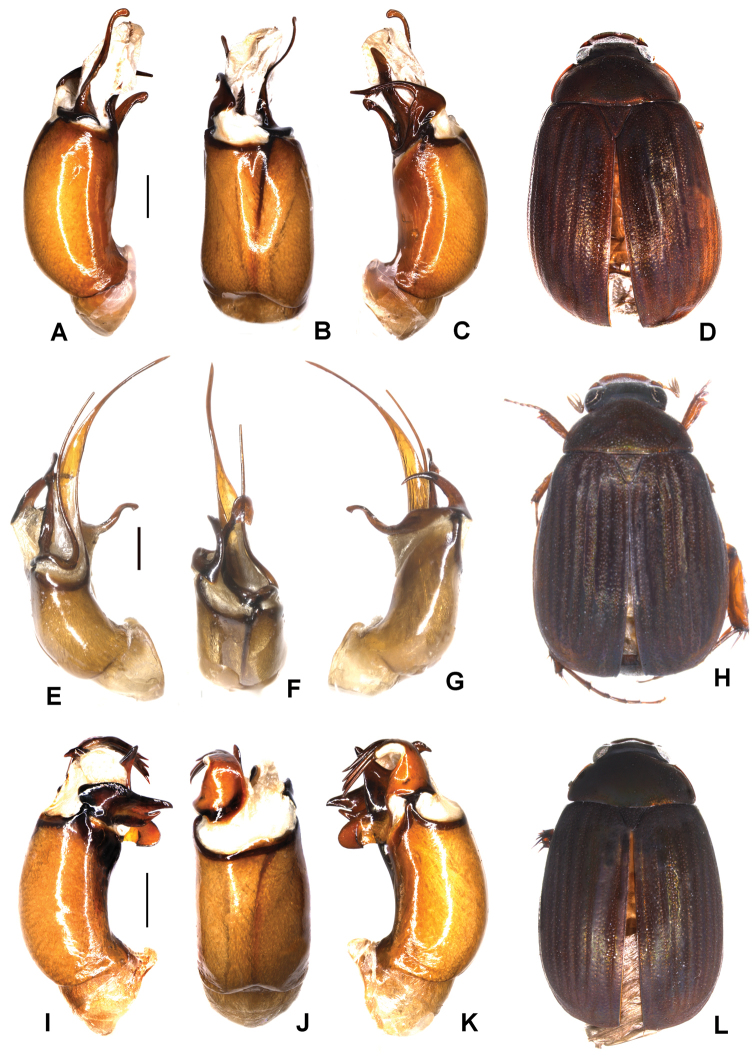
**A–D***Tetrasericabachmaensis* sp. n. (holotype) **E–H***T.pluriuncinata* sp. n. (holotype) **I–L***T.chiangdaoensis* Kobayashi, 2017 (Thailand, Chiang Dao) **A, E, I** aedeagus, left side lateral view **C, G, K** aedeagus, right side lateral view **B, F, J** parameres, dorsal view **D, H, L** habitus. Scale bars: 0.5 mm. Habitus not to scale.

Aedeagus: Fig. 8A–C. Habitus: Fig. 8D.

Female unknown.

#### Diagnosis.

*Tetrasericabachmaensis* sp. n. differs from the similar *T.sigulianshanica* Liu et al., 2014 and *T.curviforceps* sp. n. by its shorter median phallobasal lamina.

#### Etymology.

The new species is named after the type locality, Bach Ma National Park (adjective in the nominative singular).

### Tetraserica pluriuncinata sp. n.

Taxon classificationAnimaliaColeopteraMelolonthidae

http://zoobank.org/24567129-EB28-4AE2-A241-270AD4E68C85

Figures 8 , 53

#### Type material examined.

Holotype: ♂ “NE Thailand 1-15.[v.]1991. Mae Hong Son, Ban Huai Po, 800–1600 m, S. Bily leg.” (ZFMK). Paratypes: 1 ♂ “NW Thailand, Mae Hong Son, 9-16.V.1991 Ban Huai Po 1600 m leg. P. Pacholátko/ coll. P. Pacholátko/ TS135/ 146 Sericini Asia spec.” (CPPB), 7 ♂♂ “NW Thailand, 19.19N, 97.59E, Mae Hong Son, 1991 Ban Huai Po, 1600–2000 m 17.-23.5., L. Dembický leg.” (NHMW, ZFMK).

#### Description.

Length of body: 7.6 mm; length of elytra: 5.9 mm; maximum width: 5 mm. Surface of labroclypeus and disc of frons glabrous. Smooth area anterior to eye twice as wide as long. Eyes moderately large, ratio of diameter/interocular width: 0.77. Ratio of length of metepisternum/metacoxa: 1/1.52. Metatibia moderately long and wide, ratio width/length: 1/3.38; basal group of dorsal spines of metatibia at first third of metatibial length.

Aedeagus: Fig. 8E–G. Habitus: Fig. 8H.

Female unknown.

#### Variation.

Length of body: 7.6 mm; length of elytra: 5.8–5.9 mm; maximum width: 4.8–5 mm.

#### Diagnosis.

*Tetrasericapluriuncinata* sp. n. differs from *T.bachmaensis* by the left paramere being split into two long branches (or lobes) and by having much longer median phallobasal apophysis.

#### Etymology.

The species name (adjective in the nominative singular) is derived from the combined Greek word *pluri*s (higher) and Latin word *uncinatus* (hooked), with reference to the pluri-hooked shape of the right paramere.

### Tetraserica chiangdaoensis

Taxon classificationAnimaliaColeopteraMelolonthidae

Kobayashi, 2017

Figures 8 , 50

Tetraserica chiangdaoensis Kobayashi, 2017: 41, figs 8, 17.

#### Material examined.

1 ♂ “X-DA4842 labcode VD102 Thailand, Chiang Dao Hill Resort (100 km N of Chiang Mai) 600 m, 1-5.vi.2009, S. Murzin leg. Tetraserica spTH_V53/ X-DA 4842/ 925 Sericini Asia spec.” (ZFMK), 1 ♂ “DA2072/ 835092/ 835092 X-DA2072 Thailand Chiang Mai Pr. 114 km N Chiang Mai Highway station, 700 m, 5-7.vi.2008 leg. S. Murzin” (ZFMK), 1 ♂ “X-DA4543 labcode VD041 Thailand, Chiang Dao Hill Resort (100 km N of Chiang Mai) 600 m, 28-31.v.2009, S. Murzin leg., Tetraserica spTH_V15/ X-DA4543” (ZFMK), 1 ♂ “X-DA4741 labcode VD093 Thailand, Chiang Dao Hill Resort 600 m, 19.55779N 99.0766E, 28.iv.-5.v.2011, M. Murzin, O. Shulga leg., Tetraserica spTH_V53/ X-DA4714” (ZFMK), 1 ♂ “X-DA4770 labcode: VD097, LAOS, Stupa GH, 5km W Muang Sing, 750 m, 21.1482N 101.1711E, 9.v-2.vi.2011, M. Murzin, O. Shulga leg. Tetraserica spLA_V56” (ZFMK), 1 ♂ “X-DA4846 labcode VD103 Thailand, Chiang Dao Hill Resort (100 km N of Chiang Mai) 600 m, 1-5.vi.2009, S. Murzin leg. Tetraserica spTH_V53/ X-DA 4846” (ZFMK), 1 ♂ “X-DA4863 labcode VD104 Thailand, Chiang Dao Hill Resort (100 km N of Chiang Mai) 600 m, 1-5.vi.2009, S. Murzin leg. Tetraserica spTH_V53/ X-DA 4863” (ZFMK), 1 ♂ “X-DA4864 labcode VD105 Thailand, Chiang Dao Hill Resort (100 km N of Chiang Mai) 600 m, 1-5.vi.2009, S. Murzin leg. Tetraserica spTH_V53/ X-DA 4864” (ZFMK), 1 ♂ “X-DA4884 Thailand N: Chiang Dao Hill Resort (100 km N of Chiang Mai) 600 m 28.v.-8.vi. 2009 S. Murzin leg.” (ZFMK), 1 ♂ “X-DA4887 Thailand N: Chiang Dao Hill Resort (100 km N of Chiang Mai) 600m 28.v.-8.vi. 2009 S. Murzin leg.” (ZFMK), 1 ♂ “X-DA4916 Thailand N: Chiang Dao Hill Resort (100 km N of Chiang Mai) 600 m 28.v.-8.vi. 2009 S. Murzin leg.” (ZFMK), 1 ♂ “X-DA4919 Thailand N: Chiang Dao Hill Resort (100 km N of Chiang Mai) 600 m 28.v.-8.vi. 2009 S. Murzin leg.” (ZFMK), 1 ♂ “X-DA4930 Thailand N: Chiang Dao Hill Resort (100 km N of Chiang Mai) 600 m 28.v.-8.vi. 2009 S. Murzin leg.” (ZFMK), 1 ♂ “X-DA4959 Thailand N: Chiang Dao Hill Resort (100 km N of Chiang Mai) 600 m 28.v.-8.vi. 2009 S. Murzin leg.” (ZFMK), 1 ♂ “X-DA4936 Thailand N: Chiang Dao Hill Resort (100 km N of Chiang Mai) 600 m 28.v.-8.vi. 2009 S. Murzin leg.” (ZFMK), 1 ♂ “X-DA4937 Thailand N: Chiang Dao Hill Resort (100 km N of Chiang Mai) 600 m 28.v.-8.vi. 2009 S. Murzin leg.” (ZFMK), 1 ♂ “THAI-NE, Loei prov., Phu Kradung N.P., 16°52'N, 101°49'E, 16-18.v.1999, 1000m, D. Hauck leg./ coll. P. Pacholátko/ 128 Sericini Asia spec.” (CPPB), 1 ♂ “Thailand, Nam Nao, Phetchabua; 16°5'N, 101°40'E; 19.V.1999; leg. K. Masumoto” (ZFMK).

Aedeagus: Fig. 8I–K. Habitus: Fig. 8L.

### Tetraserica sapana sp. n.

Taxon classificationAnimaliaColeopteraMelolonthidae

http://zoobank.org/839700D2-1AB4-40DC-82A5-2A9900923CD7

Figures 9 , 51

#### Type material examined.

Holotype: ♂ “Vietnam bor. Pr. Hoang lien son SA PA V.1990, J Picka lgt./ 908 Sericini Asia spec.” (NHMB).

#### Description.

Length of body: 8.8 mm; length of elytra: 7.1 mm; maximum width: 5.5 mm. Surface of labroclypeus and disc of frons glabrous. Smooth area anterior to eye twice as wide as long. Eyes moderately large, ratio of diameter/interocular width: 0.6. Ratio of length of metepisternum/metacoxa: 1/1.31. Metatibia moderately long and wide, ratio width/length: 1/4.91; basal group of dorsal spines of metatibia at first third of metatibial length.

**Figure 9. A–E:**
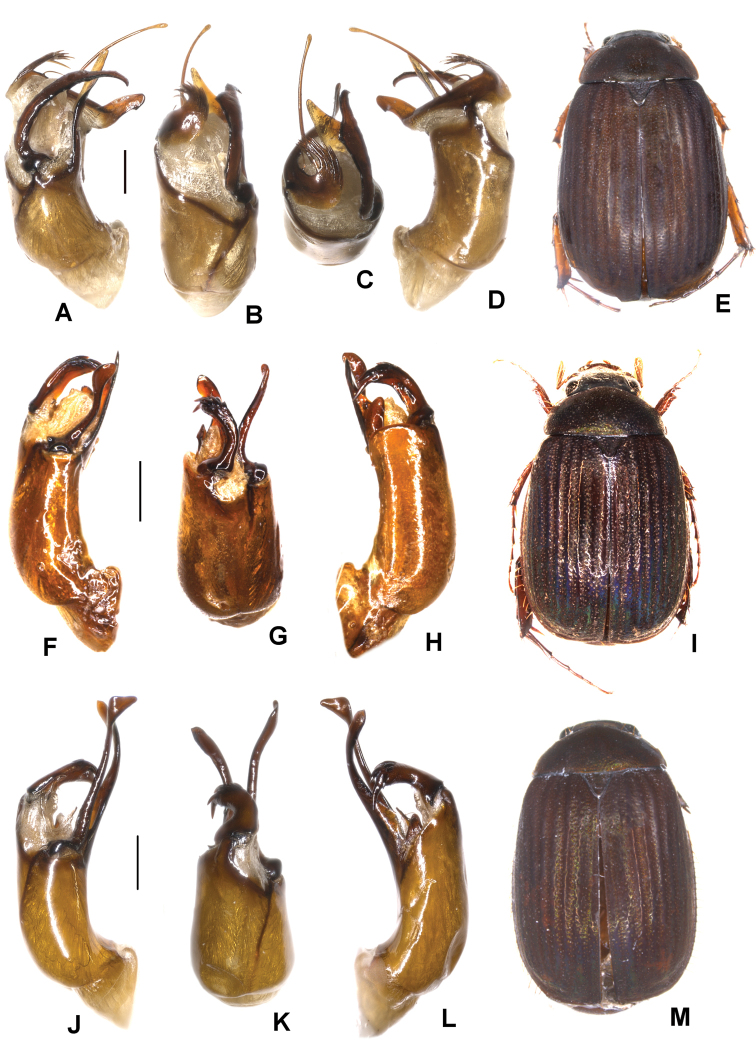
*Tetrasericasapana* sp. n. (holotype) **F–I***T.setuliforceps* sp. n. (holotype) **J–M***T.parasetuliforceps* sp. n. (holotype) **A, F, J** aedeagus, left side lateral view **D, H, L** aedeagus, right side lateral view **B, G, K** parameres, dorsal view **C** parameres, distal view **E, I, M** habitus. Scale bars: 0.5 mm. Habitus not to scale.

Aedeagus: Fig. 9A–D. Habitus: 9E.

Female unknown.

#### Diagnosis.

*Tetrasericasapana* sp. n. is similar to *T.pluriuncinata* sp. n. in shape of male genitalia but differs from the latter by the shorter and simpler left paramere and the presence of trichome-like spines on the dorsal lobe of the right paramere.

#### Etymology.

The new species is named after the type locality, Sa Pa (adjective in the nominative singular).

### Tetraserica setuliforceps sp. n.

Taxon classificationAnimaliaColeopteraMelolonthidae

http://zoobank.org/73A2B365-28C8-4D42-9A0D-C548B98D9D28

Figures 9 , 56

#### Type material examined.

Holotype: ♂ “NW Thailand, 19.19N, 97.59E, Mae Hong Son, 1991 Ban Huai Po, 1600–2000 m 9–16.5., L. Dembický leg./ 130 Sericini Asia spec.” (NHMW). Paratypes: 1 ♂ “NW Thailand, 19.19N, 97.59E, Mae Hong Son, 1991 Ban Huai Po, 1600–2000 m 17.-23.5., L. Dembický leg.” (NHMW), 1 ♂ “NW Thailand: 7-12.V., Mae Hong Son distr. 1996 Soppong-pai; 19°27’, 98°20’, J. Horák lgt.; 1500 m leg./ coll. Pacholátko” (CPPB), 1 ♂ “NW Thailand 9.-16.V. Mae Hong Son 1991, Ban Huai Po, 1600 m, leg. P. Pacholátko/ coll. P. Pacholátko” (CPPB), 1 ♂ “NW THAI 7-14.V. DOI SUTHEP PUI 1300–1500 m 1992 leg. P. Pacholátko/ coll. P. Pacholátko” (CPPB), 1 ♂ “THAI 24-29.lV.1993, DOI SUTHEP Pacholátko & Dembicky leg/ coll. P. Pacholátko” (ZFMK).

#### Description.

Length of body: 7.5 mm; length of elytra: 5.9 mm; maximum width: 4.6 mm. Surface of labroclypeus and disc of frons glabrous. Smooth area anterior to eye twice as wide as long. Eyes moderately large, ratio of diameter/interocular width: 0.59. Ratio of length of metepisternum/metacoxa: 1/1.68. Metatibia moderately long and wide, ratio width/length: 1/3.33; basal group of dorsal spines of metatibia at first third of metatibial length.

Aedeagus: Fig. 9F–H. Habitus: Fig. 9I.

Female unknown.

#### Variation.

Length of body: 6.7–8.5 mm; length of elytra: 4.9–6.0 mm; maximum width: 4.5–5.0 mm.

#### Diagnosis.

*Tetrasericasetuliforceps* sp. n. resembles *T.changjiangensis* Liu et al., 2014 in the shape of aedeagus but it differs by the distinctly longer dorsal lobe of right paramere, which also bears trichome-like spines, and by the nearly straight left paramere.

#### Etymology.

The species name (noun in apposition) is derived from the combined Latin words *setulus* (with small setae) and *forceps*, with reference to the setae present on the right paramere.

### Tetraserica parasetuliforceps sp. n.

Taxon classificationAnimaliaColeopteraMelolonthidae

http://zoobank.org/CD33C3EF-ECE2-4B03-BDA7-FDECC262B58E

Figures 9 , 49

#### Type material examined.

Holotype: ♂ “THAILAND, Prov. Tak, Doi Mussoi, 800 m a.V. 2010, leg. T. Ihle 16°45, 309'N, 98°55, 404'E/ 901 Sericini Asia spec.” (NME).

#### Description.

Length of body: 7.6 mm; length of elytra: 6 mm; maximum width: 4.9 mm. Surface of labroclypeus and disc of frons glabrous. Smooth area anterior to eye twice as wide as long. Eyes moderately large, ratio of diameter/interocular width: 0.63. Ratio of length of metepisternum/metacoxa: 1/1.73. Metatibia short and wide, ratio width/length: 1/3; basal group of dorsal spines of metatibia at first third of metatibial length.

Aedeagus: Fig. 9J–L. Habitus: Fig. 9M.

Female unknown.

#### Diagnosis.

*Tetrasericaparasetuliforceps* sp. n. differs from *T.setuliforceps* sp. n. by the longer parameres, with the left paramere being as long as phallobase.

#### Etymology.

The species name (noun in apposition) is derived from the combined Greek prefix *para*- (near) and the species name *setuliforceps*, with reference to the similarity to the previous species.

### Tetraserica kollae sp. n.

Taxon classificationAnimaliaColeopteraMelolonthidae

http://zoobank.org/44B57F67-EEEA-40A2-84AC-88416F684C8B

Figures 10 , 48

#### Type material examined.

Holotype: ♂ “INDIA: Kolasib, Mizoram; 24°13'N, 92°40'E; K. Sreedevi 25.iv.2014/ 942 Sericini Asia spec.” (NBAIR). Paratype: 1 ♂ “INDIA: Kolasib, Mizoram; 24°13'N, 92°40'E; K. Sreedevi 25.iv.2014” (ZFMK).

#### Description.

Length of body: 7.5 mm; length of elytra: 5.6 mm; maximum width: 4.8 mm. Surface of labroclypeus and disc of frons glabrous. Smooth area anterior to eye twice as wide as long. Eyes moderately large, ratio of diameter/interocular width: 0.67. Ratio of length of metepisternum/metacoxa: 1/1.48. Metatibia short and wide, ratio width/length: 1/3.21; basal group of dorsal spines of metatibia at first third of metatibial length.

**Figure 10. A–E:**
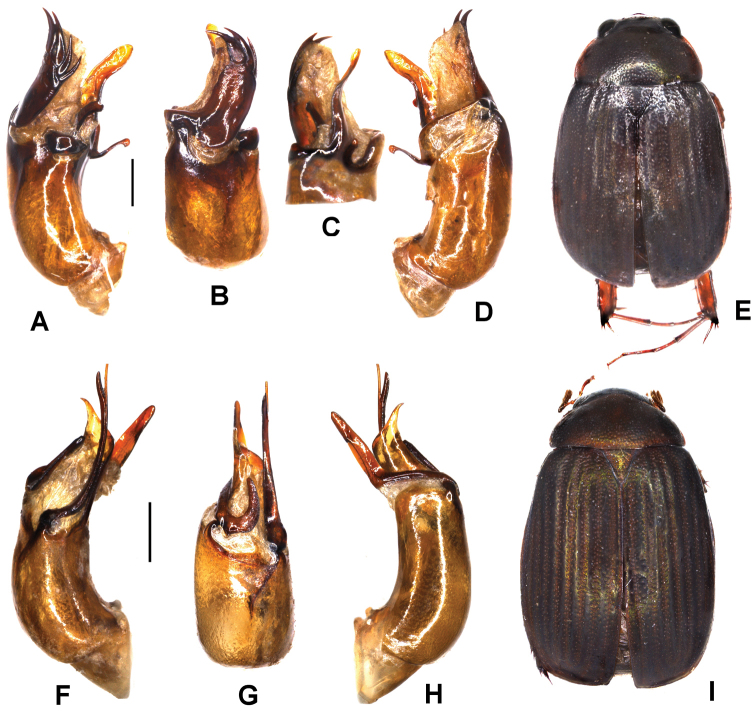
*Tetrasericakollae* sp. n. (holotype) **F–I***T.jakli* sp. n. (holotype) **A, F** aedeagus, left side lateral view **D, H** aedeagus, right side lateral view **B, G** parameres, dorsal view **C** parameres, ventral view **E, I** habitus. Scale bars: 0.5 mm. Habitus not to scale.

Aedeagus: Fig. 10A–D. Habitus: Fig. 10E.

Female unknown.

#### Variation.

Length of body: 7.5–7.8 mm; length of elytra: 5.6–5.8 mm; maximum width: 4.8–4.9 mm.

#### Diagnosis.

*Tetrasericakollae* sp. n. is very similar to *T.brahmaputrae* Ahrens, 2004 in the shape of the genitalia; it differs from it by having a wider dorsal lobe of the right paramere, which is inserted more laterally on the right side, as well as the more strongly curved distal lobe of the right paramere.

#### Etymology.

The new species is named after Dr Kolla Sreedevi, collector of the new species (noun in the genitive singular).

### Tetraserica jakli sp. n.

Taxon classificationAnimaliaColeopteraMelolonthidae

http://zoobank.org/ EC8E05B1-BB73-49CA-8F4D-667A4DBBF269

Figures 10 , 49

#### Type material examined.

Holotype: ♂ “Laos, Attapeau prov.; Annam Highlands Mts Dong Amphan; NBCA, ca. 1160 m NONG FA (crater lake) env.; 15°05.9'N, 107°25.6'E; St. Jakl lgt, 30.4.-6.5.2010/ 1012 Asia Sericini sp. “ (ZFMK).

#### Description.

Length of body: 7.3 mm; length of elytra: 5.6 mm; maximum width: 4.8 mm. Surface of labroclypeus and disc of frons glabrous. Smooth area anterior to eye twice as wide as long. Eyes small, ratio of diameter/interocular width: 0.5. Ratio of length of metepisternum/metacoxa: 1/1.78. Metatibia short and wide, ratio width/length: 1/2.86; basal group of dorsal spines of metatibia at first third of metatibial length.

Aedeagus: Fig. 10F–H. Habitus: Fig. 10I.

Female unknown.

#### Diagnosis.

The shape of aedeagus of *T.jakli* sp. n. is somewhat similar to that of *T.ferrugata* (Blanchard, 1850) from the Himalayas; the left paramere of the new species is, however, distinctly longer and distinctly split into two short filiform branches at apex.

#### Etymology.

The new species is named after its collector, St. Jakl (noun in genitive singular).

### Tetraserica appendiculata sp. n.

Taxon classificationAnimaliaColeopteraMelolonthidae

http://zoobank.org/C694F1B9-EA18-4A1D-BA35-5C9D29021ADE

Figures 11 , 46

#### Type material examined.

Holotype: ♂ “X-DA4795 labcode: VD100, LAOS, Stupa GH, 5 km W Muang Sing, 750 m, 21.1482N 101.1711E, 9.v-2.vi.2011, M. Murzin, O. Shulga leg. Tetraserica spLA_V59/ sp-LA-v59/ X-DA4795” (ZFMK). Paratype: 1 ♂ “Laos, 21°09'N, 101°19'E, Louangnamtha pr. Namtha->Muang Sing, 5-31.v.1997, 900-Vit. Kubáň leg. -1200 m/ Coll. P. Pacholátko/ LS57” (CPPB).

#### Description.

Length of body: 8.8 mm; length of elytra: 6.5 mm; maximum width: 5.3 mm. Surface of labroclypeus and disc of frons glabrous. Smooth area anterior to eye twice as wide as long. Eyes moderately large, ratio of diameter/interocular width: 0.65. Ratio of length of metepisternum/metacoxa: 1/1.52. Metatibia short and wide, ratio width/length: 1/3.21; basal group of dorsal spines of metatibia at first third of metatibial length.

**Figure 11. A–E:**
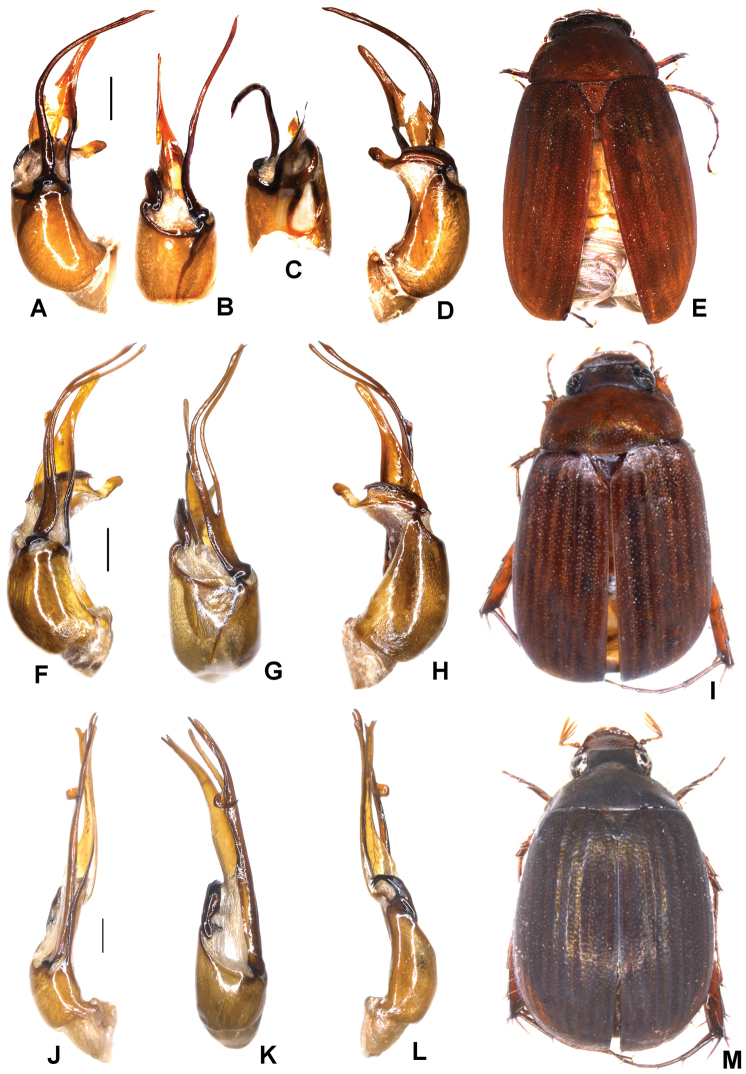
*Tetrasericaappendiculata* sp. n. (holotype) **F–I***T.allomengeana* sp. n. (holotype) **J–M***T.quadrifurcata* sp. n. (holotype) **A, F, J** aedeagus, left side lateral view **D, H, L** aedeagus, right side lateral view **B, G, K** parameres, dorsal view **C** parameres, ventral view **E, I, M** habitus. Scale bars: 0.5 mm. Habitus not to scale.

Aedeagus: Fig. 11A–D. Habitus: Fig. 11E.

Female unknown.

#### Variation.

Length of body: 7.8–8.8 mm; length of elytra: 5.9–6.5 mm; maximum width: 5.0–5.3 mm.

#### Diagnosis.

The shape of aedeagus of *T.appendiculata* sp. n. is very similar to that of *T.mengeana* Liu et al., 2014; the new species differs in having one filiform sub-branch of the left paramere reduced, being much shorter than the width of principal branch at apical third.

#### Etymology.

The species name (adjective in the nominative singular) is derived from the Latin word *appendiculus* (small appendage), with reference to the small appendix of the paramere.

### Tetraserica allomengeana sp. n.

Taxon classificationAnimaliaColeopteraMelolonthidae

http://zoobank.org/1EFA30B9-2618-4324-A6E0-2EB5318CBFFF

Figures 11 , 46

#### Type material examined.

Holotype: ♂ “NE-Laos: Houa Phan prov.; Ban Saleui, Phou Pan (Mt.)- 20°12'N, 104°01'E, 11.iv.-15.v.2012, 1300–1900 m leg. C. Holzschuh - ZFMK Ankauf 2012/ sp 34 Silvia” (ZFMK). Paratypes: 4 ♂♂ “NE-Laos: Houa Phan prov.; Ban Saleui, Phou Pan (Mt.)- 20°12'N, 104°01'E, 11.iv.-15.v.2012, 1300–1900 m leg. C. Holzschuh - ZFMK Ankauf 2012” (ZFMK), 1 ♂ “NE-Laos: Houa Phan prov.; Ban Saleui, Phou Pan (Mt.)- 20°12'N, 104°01'E, 11.iv.-15.v.2012, 1300–1900 m, leg. C. Holzschuh ZFMK Ankauf 2012/13” (ZFMK), 1 ♂ “NE-Laos: Houa Phan prov.; Ban Saleui, Phou Pan (Mt.) 20°12'N, 104°01'E, 11.iv.-15.v.2012, 1300–1900 m, leg. C. Holzschuh - ZFMK Ankauf 2012/2013/ sp 88 Silvia” (ZFMK), 6 ♂♂ “NE-Laos: Houa Phan prov., 20°13'09–19"N, 103°59'54"–104°00'03"E, 1480–1510 m, Phou Pan (Mt.) 22.IV.- 14.V. 2008, Vit Kubáň leg.” (ZFMK), 2 ♂♂ “NE-Laos: Houa Phan prov., 20°13'09–19"N, 103°59'54"–104°00'03"E, Phu Pane Mt., 1480–1510 m, 22.4.- 14.5.2008, Vit Kubáň leg.” (ZFMK), 1 ♂ “Laos-NE, Houa Phan prov., 20°12–13.5'N, 103°59.5'–104°01'E, Ban Saleui- Phou Pane Mt. 1340–1870 m, 15.iv.-15.v.2008; Lao collectors leg./ 911 Sericini Asia spec. “ (NHMB), 1 ♂ “Laos, 24-30.iv.1999, Louangphrabang pr., 20°33–4'N, 102°14'E, Ban Song Cha (5km W), 1200m, Vít Kubáň leg./ 202 Sericini Asia spec.” (CPPB), 1 ♂ “Laos-NE, Houa Phan prov., 20°13'09–19"N, 103°59'54’’ – 104°00'03"E, 1480–1550 m, Phou Pane Mt., 1.-16.vi.2009, Zdeněk Kraus leg./ NHMB Basel, NMPC Prague Laos 2009 Expedition: M. Brancucci, M. Geiser, Z. Kraus, D. Hauck, V. Kubán” (NHMB).

#### Description.

Length of body: 8.9 mm; length of elytra: 6.6 mm; maximum width: 5.0 mm. Surface of labroclypeus and disc of frons glabrous. Smooth area anterior to eye twice as wide as long. Eyes large, ratio of diameter/interocular width: 0.7. Ratio of length of metepisternum/metacoxa: 1/1.65. Metatibia moderately long and wide, ratio width/length: 1/3.36; basal group of dorsal spines of metatibia at first third of metatibial length.

Aedeagus: Fig. 11F–H. Habitus: Fig. 11I.

Female unknown.

#### Variation.

Length of body: 8.1–9.1 mm; length of elytra: 5.6–6.9 mm; maximum width: 4.8–5.3 mm.

#### Diagnosis.

The shape of aedeagus of *T.allomengeana* sp. n. is very similar to that of *T.mengeana* Liu et al., 2014; the new species differs by the filiform branches of left paramere being approximately two thirds of the paramere length, and by having the median phallobasal lamina evenly narrowed towards apex, rather than being narrow for most of its distal part as in *T.mengeana*.

#### Etymology.

The species name (adjective in the nominative singular) is derived from the combined Greek word *allo*- (other) and the species name *mengeana*, with reference to the similarity to *T.mengeana*.

### Tetraserica quadrifurcata sp. n.

Taxon classificationAnimaliaColeopteraMelolonthidae

http://zoobank.org/16DE05DE-9B95-4091-AA27-6E973A907E0E

Figures 11 , 50

#### Type material examined.

Holotype: ♂ “Cambodia: Kirirom Nat. Park, h = 650 m, 11°18'6"N, 104°5'40"E, 9-16.XII.1999 leg. M. & S. Murzin/ coll. D. Ahrens/ 117 Sericini Asia spec.” (ZFMK). Paratype: 1 ♂, 1 ♀ “Thailand, Khao Yai; at light, 15.VII.1991; leg. M. Tanida” (ZFMK).

#### Description.

Length of body: 8.9 mm; length of elytra: 6.8 mm; maximum width: 5.6 mm. Surface of labroclypeus and disc of frons glabrous. Smooth area anterior to eye twice as wide as long. Eyes moderately large, ratio of diameter/interocular width: 0.62. Ratio of length of metepisternum/metacoxa: 1/1.67. Metatibia moderately long and wide, ratio width/length: 1/3.33; basal group of dorsal spines of metatibia at first third of metatibial length.

Aedeagus: Fig. 11J–L. Habitus: 11M.

#### Variation.

Length of body: 8.9–9.8 mm; length of elytra: 6.8–7.0 mm; maximum width: 5.5–5.6 mm. Female: Eyes as large as in male; antennal club composed of four antennomeres, basal joint of club one quarter as long as club.

#### Diagnosis.

*Tetrasericaquadrifurcata* sp. n. differs from all other *Tetraserica* species by its long median phallobasal apophysis having the median phallobasal lamina bifurcate at apex.

#### Etymology.

The species name (adjective in the nominative singular) is derived from the combined Latin prefix *quadri* (four) and *furcata* (forked), with reference to the shape of doubly bifurcate parameres.

### Tetraserica siantarensis

Taxon classificationAnimaliaColeopteraMelolonthidae

(Moser, 1922) comb. n.

Figures 12 , 50

Neoserica siantarensis Moser, 1922: 104.

#### Type material examined.

Lectotype (here designated): ♂ “Fort de Kock (Sumatra) 920 m Januari 1922 leg. E. Jacobson/ Neosericasiantarensis Mos. [handwritten by Moser] det. J. Moser 1926 [handwritten by curator?]” (ZMAN). Paralectotype: 1 ♀ “Fort de Kock (Sumatra) 920 m Januari 1922 leg. E. Jacobson/ Neosericasiantarensis Mos.[handwritten by Moser]/ det. J. Moser 1926 [handwritten by curator?]” (ZMAN).

#### Additional material examined.

2 ♂♂, 1 ♀ “Malaysia W, Kelantan 40 km N of Gua Musang Gunung Berangkat Kampong Riek; 1100 m, 15.v.-8.vi.2017 Petr Cechovsky lgt.” (NME), 1 ♂, 1 ♀ “W-Sumatra Payakumbuh Sarilamak env. 15.-16.2.91 Jezevec lgt.” (ZFMK), 1 ♂ “Indonesia W, Sumatra, Harau Valley, Payakumbuh near Bukit Tinggi, XI.1993-IV.1994, Sarimudanas leg.” (ZFMK), 1 ♂, 1 ♀ “Indonesia Sumatra Utara Sungei Kopas II, ca 60 km E Pematangsiantar ca. 300 m, Sekundärwald 25.03.1997, leg.C U. P. Zorn” (ZFMK), 1 ♂ “I-W. Sumatra 600 m Paykumbuh 6-10.1. Harau vill. env. St. Jakl lgt.1991” (ZFMK), 1 ♂, 1 ♀ “Sumatra Utara Aek Bum b. Rantanprabat 11/12.2.96 lg. Zorn” (ZFMK), 2 ♂♂ “Malaysia 10-16.iv.1999 Kelantan prov. Kampong Raja env. Lgt. Mir. Janalik” (ZFMK), 1 ♂ “Indonesien Sumatra Aceh-Prov. Umg. Kotacane Ketambe 15.-22. Januar 2016 leg. Horst Rudolph” (Coll. H. Rudolph), 1 ♂ “Indonesien/Sumatra Sindar Raya LF Simaraopa 350 m NN 24. Februar 1993 leg. Horst Rudolph” (Coll. H. Rudolph).

#### Diagnostic description.

Body length: 8.8 mm, length of elytra: 7.2 mm, width: 6.0 mm.

Surface of labroclypeus and disc of frons glabrous. Smooth area anterior to eye three times as wide as long. Antennal club 1.1 times as long as remaining antennomeres combined. Eyes moderately large; ratio of diameter/interocular width: 0.69. Ratio of length of metepisternum/metacoxa: 1/1.59. Metafemur dull, anterior margin acute, without submarginal serrated line; anterior row of setae-bearing punctures absent; posterior margin with a blunt tooth. Metatibia short and wide, ratio width/length: 1/2.7; basal group of dorsal spines of metatibia at first third of metatibial length.

**Figure 12.**
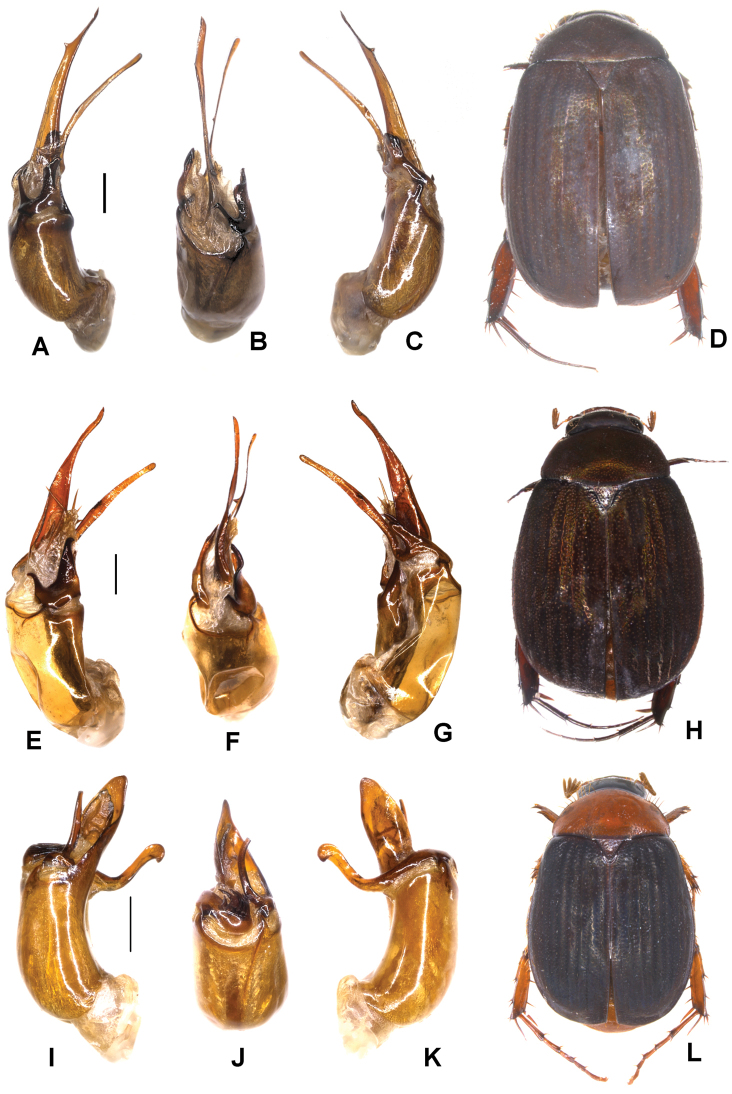
**A–D***Tetrasericasiantarensis* (Moser, 1922) (lectotype) **E–H***Tetrasericaferesiantarensis* sp. n. (holotype) **I–L***T.rubrithorax* sp. n. (holotype) **A, E, I** aedeagus, left side lateral view **C, G, K** aedeagus, right side lateral view **B, F, J** parameres, dorsal view **D, H, L** habitus. Scale bars: 0.5 mm. Habitus not to scale.

Aedeagus: Fig. 12A–C. Habitus: Fig. 12D.

### Tetraserica feresiantarensis sp. n.

Taxon classificationAnimaliaColeopteraMelolonthidae

http://zoobank.org/C6D00E7D-2957-468E-B664-5D8D33209B22

Figures 12 , 47

#### Type material examined.

Holotype: ♂ “Malaysia: Hulu Perak, Bangunan Camp c/o Kampung Semelor (E shore Lake Tasek-Temengor), 230 m a.s.l., 10-19/VII/2007 L. Bartolozzi legit/ Museo Zoologico “La Specola” num. Mag. 2814/ 916 Sericini Asia spec.” (MZUF). Paratypes: 2 ♂♂ “Malaysia: Hulu Perak, Bangunan Camp c/o Kampung Semelor (E shore Lake Tasek-Temengor), 230 m a.s.l., 10-19/VII/2007 L. Bartolozzi legit/ Museo Zoologico “La Specola” num. Mag. 2814” (MZUF, ZFMK), 1 ♂ “Malaysia: Hulu Perak, Bangunan Camp c/o Kampung Semelor (E schore Lake Tasek-Temengor), 101°26'16"E, 5°30'18"N, 230 m 29.VI-4.VII.2008/ L. Bartolozzi, G. Mazza, F. Cianferoni, F. Fabiano (num. Mag. 2847)” (MZUF), 1 ♂ “S Thailand: Betong Gunung Cang dun vill. Yala dist., J. Horák leg./ 159 Sericini Asia spec.” (CPPB), 1 ♂ “S. Thailand 25.4.1992 Betong S. Bily leg.” (NHMB).

#### Description.

Length of body: 8.9 mm; length of elytra: 6.3 mm; maximum width: 5.9 mm. Surface of labroclypeus and disc of frons glabrous. Smooth area anterior to eye twice as wide as long. Eyes moderately large, ratio of diameter/interocular width: 0.63. Ratio of length of metepisternum/metacoxa: 1/1.71. Metatibia short and wide, ratio width/length: 1/2.68; basal group of dorsal spines of metatibia at first third of metatibial length.

Aedeagus: Fig. 12E–G. Habitus: Fig. 12H.

Female unknown.

#### Variation.

Length of body: 8.9–9.5 mm; length of elytra: 6.3–7.2 mm; maximum width: 5.8–5.9 mm.

#### Diagnosis.

*Tetrasericaferesiantarensis* sp. n. differs from the very similar *T.siantarensis* Moser, 1915 by the median lamina of phallobase having no dorsal preapical tooth and the dorsal lobe of left parameres and median lamina of phallobase being as long as phallobase, rather than being distinctly longer as in *T.siantarensis*.

#### Etymology.

The species name (adjective in the nominative singular) is derived from the combined Latin words *fere*- (nearly) and the species name *siantarensis*, with reference to the similarity to *T.siantarensis* (Moser).

### Tetraserica rubrithorax sp. n.

Taxon classificationAnimaliaColeopteraMelolonthidae

http://zoobank.org/FF5D1E31-EAE0-4EB8-A6E2-1C013B9603CC

Figures 12 , 51

#### Type material examined.

Holotype: ♂ “Myanmar N (Burma), H-550 m, 21 km E Putao, Nan Sa Bon vill., leg S. Murzin & V. Sinaev 1-5.5.98/ coll D. Ahrens/ 427 Sericini Asia spec.” (ZFMK).

#### Description.

Length of body: 6.9 mm; length of elytra: 5 mm; maximum width: 3.9 mm. Body blackish brown, pronotum reddish. Surface of labroclypeus and disc of frons glabrous. Smooth area anterior to eye twice as wide as long. Eyes small, ratio of diameter/interocular width: 0.43. Ratio of length of metepisternum/metacoxa: 1/1.42. Metatibia short and wide, ratio width/length: 1/3.08; basal group of dorsal spines of metatibia at first third of metatibial length.

Aedeagus: Fig. 12I–K. Habitus: Fig. 12L.

Female unknown.

#### Diagnosis.

*Tetrasericarubrithorax* sp. n. differs from all other known *Tetraserica* species by having the body blackish brown and pronotum reddish.

#### Etymology.

The species name (noun in apposition) is derived from the combined Latin words *rubus* (red) and *thorax*, with reference to the red pronotum of the species.

### Tetraserica falciforceps sp. n.

Taxon classificationAnimaliaColeopteraMelolonthidae

http://zoobank.org/9DDFDF2A-A586-4A0B-A7C1-EE9AA5098F2D

Figures 13 , 48

#### Type material examined.

Holotype: ♂ “NE-Laos: Houa Phan prov.; Ban Saleui, Phou Pan (Mt.)- 20°12'N, 104°01'E, 11.iv.-15.v. 2012, 1300–1900 m leg. C. Holzschuh - ZFMK Ankauf 2012/ sp 37 Silvia Indochina” (ZFMK). Paratypes: 2 ♂♂, 2 ♀♀ “NE-Laos: Houa Phan prov.; Ban Saleui, Phou Pan (Mt.)- 20°12'N, 104°01'E, 11.iv.-15.v. 2012, 1300–1900 m leg. C. Holzschuh - ZFMK Ankauf 2012 “ (ZFMK), 1 ♂ “NE-Laos: Houa Phan prov.; Ban Saleui, Phou Pan (Mt.)- 20°12'N, 104°01'E, 1300–1900 m 01.-31.5.2011, leg. C. Holzschuh - ZFMK Ankauf 2012” (ZFMK), 4 ♂♂ “C. Vietnam: Thanhhoa prov., Langchan, 300 m, 17.-18.IV.1963, leg. O. Kabakov” (ZIN, ZFMK), 1 ♂ “N Vietnam 1986 prov. Ha son binh Hoa Binh 5.-7.7. V. Shliva lgt.” (NHMB).

#### Description.

Length of body: 6.9 mm; length of elytra: 5.1 mm; maximum width: 4.5 mm. Dorsal surface blackish, ventral surface reddish brown. Surface of labroclypeus and disc of frons glabrous. Smooth area anterior to eye twice as wide as long. Eyes small, ratio of diameter/interocular width: 0.44. Ratio of length of metepisternum/metacoxa: 1/1.44. Metatibia moderately long and wide, ratio width/length: 1/3.8; basal group of dorsal spines of metatibia at first third of metatibial length.

**Figure 13.**
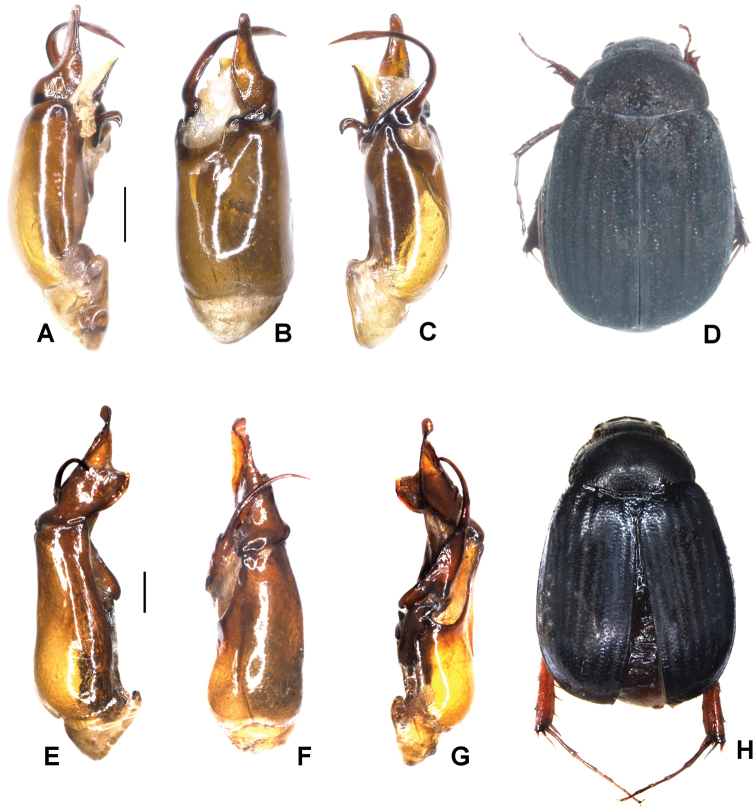
**A–D***Tetrasericafalciforceps* sp. n. (holotype) **E–H***T.olegi* sp. n. (holotype) **A, E** aedeagus, left side lateral view **C, G** aedeagus, right side lateral view **B, F** parameres, dorsal view **D, H** habitus. Scale bars: 0.5 mm. Habitus not to scale.

Aedeagus: Fig. 13A–C. Habitus: Fig. 13D.

#### Variation.

Length of body: 6.9–7.5 mm; length of elytra: 5.1–5.9 mm; maximum width: 4.5–5.2 mm. Female: Eyes as large as in male; antennal club composed of three joints, short, as long as remaining antennomeres combined.

#### Diagnosis.

*Tetrasericafalciforceps* sp. n. is rather similar to *T.sigulianshanica* Liu et al., 2014 in shape of the aedeagus; the new species differs by the straight left paramere not being widened at apex and by the longer and widely arched dorsal lobe of the right paramere.

#### Etymology.

The species name (noun in apposition) is derived from the combined Latin words *falcis* (sickle) and *forceps*, with reference to setae present to the sickle-shaped right paramere.

### Tetraserica olegi sp. n.

Taxon classificationAnimaliaColeopteraMelolonthidae

http://zoobank.org/628E8513-AA4F-48E2-813F-621927C4E6B1

Figures 13 , 50

#### Type material examined.

Holotype: ♂ “N. Vietnam: 40 km NE Thainguyen, 300 m, 14.V.1963, leg. O Kabakov” (ZIN).

#### Description.

Length of body: 6.8 mm; length of elytra: 5.6 mm; maximum width: 0.1 mm. Dorsal surface blackish, ventral surface reddish brown. Surface of labroclypeus and disc of frons glabrous. Smooth area anterior to eye twice as wide as long. Eyes small, ratio of diameter/interocular width: 0.42. Ratio of length of metepisternum/metacoxa: 1/1.56. Metatibia short and wide, ratio width/length: 1/3.14; basal group of dorsal spines of metatibia at first third of metatibial length.

Aedeagus: Fig. 13E–G. Habitus: Fig. 13H.

Female unknown.

#### Diagnosis.

The new species is very similar to the Chinese species *T.longzhouensis* Liu et al., 2014, but differs by the wider apical lobe of its left paramere.

#### Etymology.

The new species is named after Oleg N Kabakov (1928–2009).

### Tetraserica smetsi sp. n.

Taxon classificationAnimaliaColeopteraMelolonthidae

http://zoobank.org/D76D8280-422B-45F9-B26D-3E59E5051088

Figures 14 , 51

#### Type material examined.

Holotype: ♂ “Coll. I. R. Sc. N. B., Cambodia, Pursat prov., Phnom Samkos W.S. forest edge, light trap 14.iv.2005 Leg. K. Smets & I. Var” (ISNB). Paratypes: 2 ♂♂ “Coll. I. R. Sc. N. B. Cambodia (Pursat Prov.) Phnom Samkos wild life Sanctuary, light trap Forest Edge & Primary 14-IV-2005 Leg. K. Smets & I. Var” (ISNB), 7 ♂♂ “Coll. I. R. Sc. N. B. Cambodia, Pursat prov. Phnom Samkos W.S. Pramaoy Forest Edge 15-IV-2005, Light Trap Leg. K. Smets & I. Var” (ISNB, ZFMK).

#### Description.

Length of body: 9.4 mm; length of elytra: 6.9 mm; maximum width: 5.8 mm. Surface of labroclypeus and disc of frons glabrous. Smooth area anterior to eye twice as wide as long. Eyes moderately large, ratio of diameter/interocular width: 0.65. Ratio of length of metepisternum/metacoxa: 1/1.79. Metatibia short and wide, ratio width/length: 1/2.78; basal group of dorsal spines of metatibia at first third of metatibial length.

**Figure 14.**
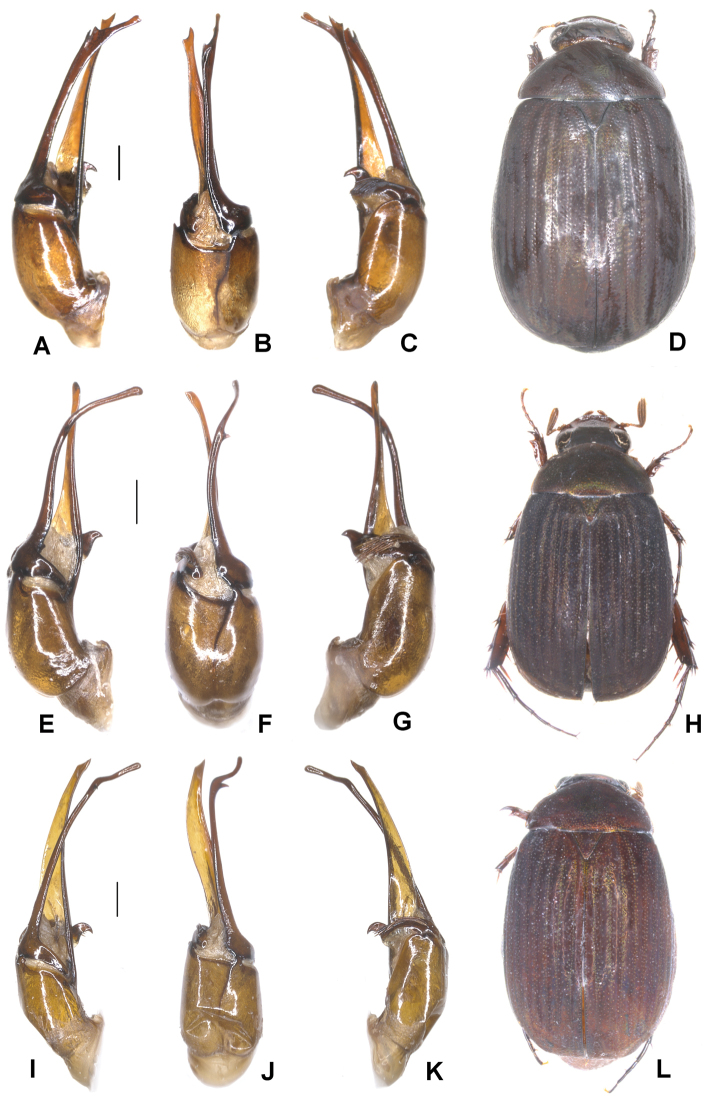
**A–D***Tetrasericasmetsi* sp. n. (holotype) **E–H***T.shangsiensis* Liu et al., 2014 (Vietnam: Tay Yen Tu Nat. Res.) **I–L***T.namnaoensis* sp. n. (holotype) **A, E, I** aedeagus, left side lateral view **C, G, K** aedeagus, right side lateral view **B, F, J** parameres, dorsal view **D, H, L** habitus. Scale bars: 0.5 mm. Habitus not to scale.

Aedeagus: Fig. 14A–C. Habitus: Fig. 14D.

Female unknown.

#### Variation.

Length of body: 9.3–9.4 mm; length of elytra: 6.6–6.9 mm; maximum width: 5.7–5.8 mm.

#### Diagnosis.

*Tetrasericasmetsi* sp. n. is very similar to *T.shangsiensis* Liu et al., 2014 in shape of the aedeagus. The new species differs from the latter by the median lamina of phallobase being approximately as long as left paramere and slightly bifurcate at apex, as well as the preapical tooth of the left paramere being dorsally positioned and duplicate. In *T.shangsiensis* the median lamina of phallobase is distinctly shorter than left paramere, simply pointed, and slightly rounded at apex.

#### Etymology.

The new species is named after one of its collectors, K Smets (noun in genitive singular).

### Tetraserica shangsiensis

Taxon classificationAnimaliaColeopteraMelolonthidae

Liu, Fabrizi, Bai, Yang & Ahrens, 2014

Figures 14 , 51

Tetraserica shangsiensis Liu, Fabrizi, Bai, Yang & Ahrens, 2014: 112, fig. 8I–L.

#### Material examined.

**Vietnam**: 1 ♂ “N-Vietnam Tam Dao Vinh Phu Prov. 21°27'18"N, 105°38'58"E, 1050–1200 m 2.-6.VI.1999 leg. Fabrizi, Jäger, Ahrens/ coll. D. Ahrens” (ZFMK), 1 ♂ “N-Vietnam, Thal Nguyen Prov., vic. Ngoc Thanh, Me Linh (IEBR station), 12.V.2012, 21°23'3"N, 105°42'44"E, 60–80 m, leg. A. Skale” (CASH), 2 ♂♂ “N-Vietnam, Bac Giang Prov., Tay Yen Tu Nat. Res., Thanh So’n, N21°12.812’ E106°45.846’, 86 m 18.5-21.5.2016 leg. A. Skale” (CASH, ZFMK), 1 ♂ “C-Vietnam: Kon Tum Province, surroundings Kon Plong, 1120 m 14°37, 350'N, 108° 17, 651E/ leg. L. Bartolozzi, S. Bambi, A. Badinelli, V. Sbordoni- at light 4-7.V.2016 (Mag. 3078)” (MZUF), 2 ♂”C Vietnam: Thua Thien-Hue Prov., A Luoi District, Sao La Nature Reserve (600–650 m) 16.077°N 107.488°E/ 20-27.V.2017 L. Bartolozzi, E. Orbach, V. Sbordoni, S. Banbi, A. Bandinelli leg. at light (numero Mag. 3089)” (MZUF, ZFMK), 4 ♂♂ “Vietnam, N, Ninh Binh Pr., 90 km SW Hanoi Cuc Phuong NP, primat rescue centre, 25.IV./ 2012, 190 m, 20°14'24"N, 105°42'53"E, leg. A. Weigel, light trap/ collection NATURKUNDE MUSEUM ERFURT” (NME), 1 ♂ “N-Vietnam, Tonkin /Tam Dao/ Vinh Phu Prov. 2-11.6.1985, Vít Kubáň leg./ Coll. P. Pacholátko/ VS38” (CPPB). **Laos**: 1 ♂ “Laos centr., Khammouan prov. NAKAI env., 4-8.5.1998, Route No 8, alt. 560±20 m N17°42.8, E 105°08.9 (GPS), E. Jendek & O. Šausa leg.” (CPPB), 3 ♂♂ “Laos, Sekong prov., ca. 12 km S. Sekong TAD FAEK waterfalls (at light) 15°14.7'N, 106°45.1'E, 118 m, Jiři Hájek leg. 8+12.v.2010” (NMPC), 1 ♂ “Khouang 1944/ Indo Chine Coll. Dussault” (NHMB).

Aedeagus: Fig. 14E–H. Habitus: Fig. 14H.

#### Remarks.

The species was recorded from China only, and it is here reported from Vietnam and Laos.

### Tetraserica namnaoensis sp. n.

Taxon classificationAnimaliaColeopteraMelolonthidae

http://zoobank.org/C8564AA1-5820-42DC-B064-2DBB7D0531D7

Figures 14 , 51

#### Type material examined.

Holotype: ♂ “Thailand, Nam Nao, Phetchabua; 16°5'N, 101°40'E; 19.V.1999; leg. K. Masumoto” (ZFMK). Paratypes: 1 ♂ “Thailand, Nam Nao, Phetchabua; 16°5'N, 101°40'E; 19.V.1999; leg. K. Masumoto” (ZFMK), 1 ♂ “Laos Bolikhamxai pr., 18°16'N, 103°11'E, 70 km NEE Vientiane, 27-30.iv.1997, 150 m, Vít Kubáň leg./ Coll. P. Pacholátko/ LS52/ 190 Sericini Asia spec.” (CPPB), 1 ♂ “X-DA4881 labcode VD107 Thailand, Chiang Dao Hill Resort (100 km N of Chiang Mai) 600 m, 28.v- 8.vi.2009, S. Murzin leg, / Tetraserica spTH_63/ sp THV63” (ZFMK), 4 ♂♂, 1 ♀ “Laos P.D.R.: Xieng Khowang 14-20.May. 1994, K. Miura leg./ coll D. Ahrens” (ZFMK), 1 ♂ “Thai 17-24/5.91 Chiang Dao mts. 19.25'N, 98.52E lgt. D. Kral 1000 m” (NMPC), 1 ♂ “Laos. Xieng Khouang 29.IV.1919. R.V. de Salvazza” (NHMUK).

#### Description.

Length of body: 9.3 mm; length of elytra: 7.3 mm; maximum width: 5.5 mm. Surface of labroclypeus and disc of frons glabrous. Smooth area anterior to eye twice as wide as long. Eyes moderately large, ratio of diameter/interocular width: 0.77. Ratio of length of metepisternum/metacoxa: 1/1.83. Metatibia moderately long and wide, ratio width/length: 1/3.67; basal group of dorsal spines of metatibia at first third of metatibial length.

Aedeagus: Fig. 14I–K. Habitus: Fig. 14L.

#### Variation.

Length of body: 9.3–11.0 mm; length of elytra: 7.3–8.2 mm; maximum width: 5.5–6.8 mm. Female: Eyes as large as in male; antennal club composed of three joints, short, as long as remaining antennomeres combined.

#### Diagnosis.

*Tetrasericanamnaoensis* sp. n. is very similar to *T.shangsiensis* Liu et al., 2014 and *T.smetsi* sp. n. in shape of the aedeagus. The new species differs from these two species by the median lamina of its phallobase being distinctly curved and evenly narrowed from the base to apex, rather than being straight and strongly narrowed after the basal third.

#### Etymology.

The new species is named after the type locality, Nam Nao (adjective in the nominative singular).

### Tetraserica konchurangensis sp. n.

Taxon classificationAnimaliaColeopteraMelolonthidae

http://zoobank.org/4D3221F5-B681-476B-B016-D56CA4828FC6

Figures 15 , 48

#### Type material examined.

Holotype: ♂ “C-Vietnam Gia Lai Province, Kon Chu Rang Nature Reserve, surroundings HQ, about 900 m 14°28, 450'N, 108°32, 401'E/ leg. L. Bartolozzi, S. Bambi, A. Badinelli, V. Sbordoni- at light 8-12.V.2016 (n° Mag. 3078)/ 238 Sericini Asia spec.” (VNMN). Paratypes: 5 ♂♂ “C-Vietnam Gia Lai Province, Kon Chu Rang Nature Reserve, surroundings HQ, about 900 m 14°28, 450'N, 108°32, 401'E/ leg. L. Bartolozzi, S. Bambi, A. Badinelli, V. Sbordoni- at light 8-12.V.2016 (n° Mag. 3078) (MZUF, ZFMK), 1 ♂ “S-Vietnam, 14.10N 108.30E 40 Km NW of An Khe Buon Luoi, 620–750 m 28.3-12.4.1995 Pacholátko & Dembický leg./ (coll.Pacholátko)” (CPPB)

#### Description.

Length of body: 9.8 mm; length of elytra: 7.5 mm; maximum width: 6.1 mm. Surface of labroclypeus and disc of frons glabrous. Smooth area anterior to eye twice as wide as long. Eyes moderately large, ratio of diameter/interocular width: 0.66. Ratio of length of metepisternum/metacoxa: 1/1.78. Metatibia short and wide, ratio width/length: 1/3.18; basal group of dorsal spines of metatibia at first third of metatibial length.

**Figure 15.**
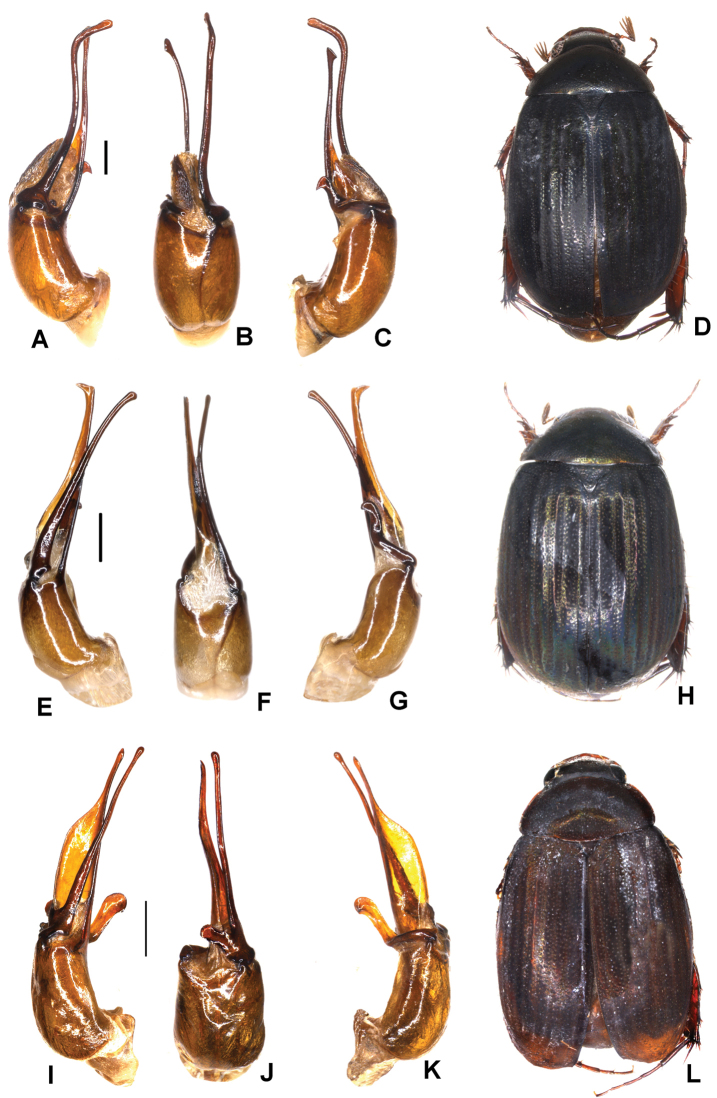
**A–D***Tetrasericakonchurangensis* sp. n. (holotype) **E–H***T.angkorwatensis* sp. n. (holotype) **I–L***T.koi* sp. n. (holotype) **A, E, I** aedeagus, left side lateral view **C, G, K** aedeagus, right side lateral view **B, F, J** parameres, dorsal view **D, H, L** habitus. Scale bars: 0.5 mm. Habitus not to scale.

Aedeagus: Fig. 15A–C. Habitus: Fig. 15D.

Female unknown.

#### Variation.

Length of body: 8.6–9.8 mm; length of elytra: 6.5–7.5 mm; maximum width: 5.5–6.1 mm.

#### Diagnosis.

*Tetrasericakonchurangensis* sp. n. is very similar to *T.shangsiensis* Liu et al., 2014, *T.namnaoensis* sp. n., and *T.smetsi* sp. n. in shape of the aedeagus. *Tetrasericakonchurangensis* sp. n. differs from all three by the left paramere lacking a tiny tooth before the apex.

#### Etymology.

The new species is named with reference to its occurrence in the Kon Chu Rang Nature Reserve (adjective in the nominative singular).

### Tetraserica angkorwatensis sp. n.

Taxon classificationAnimaliaColeopteraMelolonthidae

http://zoobank.org/B830AF1C-2AD5-4B33-ADFF-BDE63DF04610

Figures 15 , 47

#### Type material examined.

Holotype: ♂ “Coll. I. R. Sc. N. B. Cambodia: Siem Reap prov. Forest S of Angkor Wat 26-IV-2005, Light Trap, Leg K. Smets & I. Var/ 904 Sericini Asia spec.” (ISNB). Paratypes: 4 ♂♂ “Coll. I. R. Sc. N. B. Cambodia: Siem Reap prov. Forest S of Angkor Wat 26-IV-2005, Light Trap, Leg K. Smets & I. Var” (ISNB, ZFMK).

#### Description.

Length of body: 7.5 mm; length of elytra: 5.9 mm; maximum width: 4.9 mm. Surface of labroclypeus and disc of frons glabrous. Smooth area anterior to eye twice as wide as long. Eyes moderately large, ratio of diameter/interocular width: 0.68. Ratio of length of metepisternum/metacoxa: 1/1.79. Metatibia short and wide, ratio width/length: 1/3.07; basal group of dorsal spines of metatibia at first third of metatibial length.

Aedeagus: Fig. 15E–G. Habitus: Fig. 15H.

Female unknown.

#### Variation.

Length of body: 7.5–7.8 mm; length of elytra: 5.8–5.9 mm; maximum width: 4.9–5.0 mm.

#### Diagnosis.

*Tetrasericaangkorwatensis* sp. n. is rather similar to *T.gestroi* (Brenske, 1898) in shape of aedeagus; the new species differs by having the median lamina of the phallobase curved at its apex dorsally, while the base of the left paramere is not extended ventrally.

#### Etymology.

The new species is named after the type locality, Angkor Wat (adjective in the nominative singular).

### Tetraserica koi sp. n.

Taxon classificationAnimaliaColeopteraMelolonthidae

http://zoobank.org/CB4B777E-146A-440B-B689-03CA74A504FC

Figures 15 , 48

#### Type material examined.

Holotype: ♂ “Thailand (S) Prov. Chumphon, ca 5 km S von Phato 06.VII.2008 leg. M Langer/ 1000 Asia Sericini spec.” (ZFMK). Paratypes: 1 ♂, 1 ♀ “Thailand (S) Prov. Chumphon, ca 5 km S von Phato 06.VII.2008 leg. M Langer” (ZFMK).

#### Description.

Length of body: 7 mm; length of elytra: 5.4 mm; maximum width: 4.5 mm. Surface of labroclypeus and disc of frons glabrous. Smooth area anterior to eye twice as wide as long. Eyes small, ratio of diameter/interocular width: 0.48. Ratio of length of metepisternum/metacoxa: 1/1.39. Metatibia short and wide, ratio width/length: 1/3.25; basal group of dorsal spines of metatibia at first third of metatibial length.

Aedeagus: Fig. 15I–K. Habitus: Fig. 15L.

#### Variation.

Length of body: 7.0–8.4 mm; length of elytra: 5.4–6.1 mm; maximum width: 4.5–4.8 mm. Female: Eyes as large as in male; antennal club composed of three antennomeres, as long as remaining antennomeres combined.

#### Diagnosis.

*Tetrasericakoi* sp. n. differs from all other species with the left paramere composed of two lobes at its base, by the right paramere being half as long as phallobase, and the left paramere longer than phallobase.

#### Etymology.

The new species is named in honour of Prof Dr Ko, Silvia’s oncologist and director of the Johanniter Hospital in Bonn, for all his efforts and care (noun in genitive singular).

### Tetraserica fulleri sp. n.

Taxon classificationAnimaliaColeopteraMelolonthidae

http://zoobank.org/1EE52ADD-0250-4428-82C4-DF0268E6B7C4

Figures 16 , 47

#### Type material examined.

Holotype: ♂ “Thailand. Nakhon ratchasima: Khao Yai NP. 700–800 m; 21 Apr 1990 U.V. light EF♀90020E E. Fuller/ 1014 Asia Sericini spec.” (UMIC). Paratypes: 1 ♂ “Thailand. Nakhon ratchasima: Khao Yai NP. 700–800 m; 21 Apr 1990; UV light EF♀90020E E. Fuller” (ZFMK), 3 ♂♂ “Thailand: Nahon Nayok Khao Yai N.P. Lum Ta Kong 14°25.565'N, 101°23.442'E, 726 m; malaise; 19-26.iv.07 Wirat Sukho leg. T2127” (QSBG), 2 ♂♂, 1 ♀ “Thailand: Nahon Nayok Khao Yai N.P. Lum Ta Kong View Point 14°25.565'N, 101°23.442'E, 726 m; Malaise trap; 12-19.iv.07; Wirat Sukho leg. T2124” (QSBG).

#### Description.

Length of body: 9.1 mm; length of elytra: 7.1 mm; maximum width: 5.5 mm. Surface of labroclypeus and disc of frons glabrous. Smooth area anterior to eye twice as wide as long. Eyes small, ratio of diameter/interocular width: 0.53. Ratio of length of metepisternum/metacoxa: 1/1.73. Metatibia short and wide, ratio width/length: 1/3.25; basal group of dorsal spines of metatibia at first third of metatibial length.

**Figure 16. A–E:**
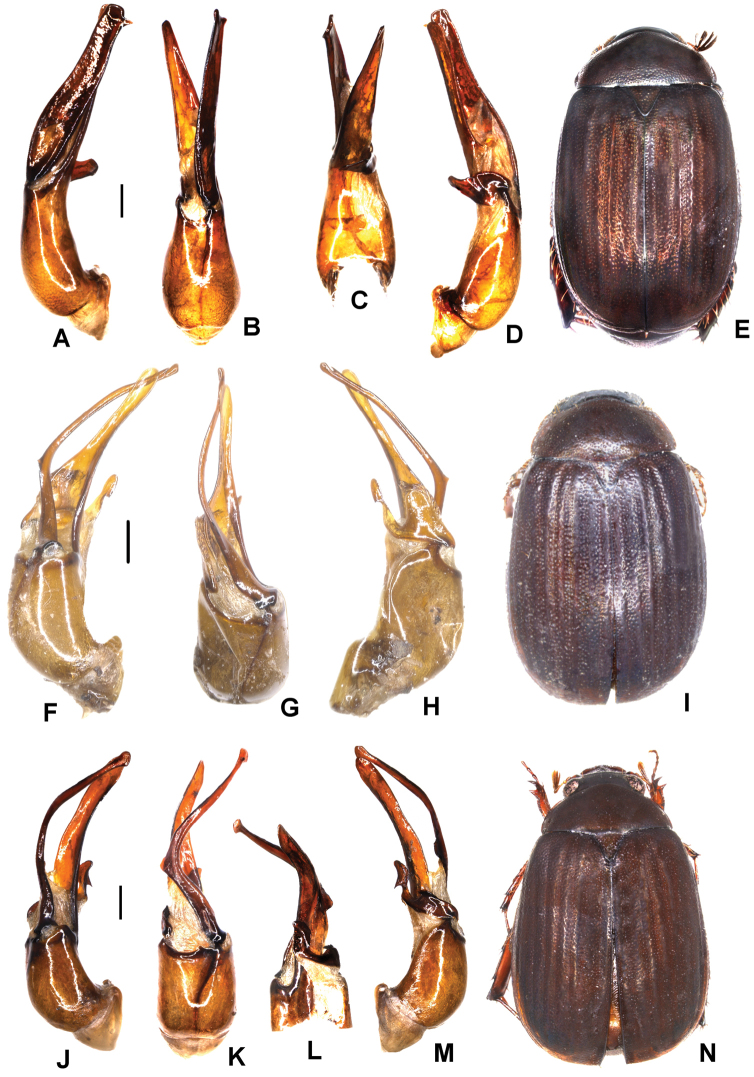
*Tetrasericafulleri* sp. n. (holotype) **F–I***T.tonkinensis* (Moser) (lectotype) **J–N***T.paratonkinensis* sp. n. (holotype) **A, F, J** aedeagus, left side lateral view **D, H, M** aedeagus, right side lateral view **B, G, K** parameres, dorsal view **C, L** parameres, ventral view **E, I, N** habitus. Scale bars: 0.5 mm. Habitus not to scale.

Aedeagus: Fig. 16A–D. Habitus: Fig. 16E.

#### Variation.

Length of body: 8.4–9.8 mm; length of elytra: 6.5–7.3 mm; maximum width: 5.2–5.8 mm. Female: Eyes as large as in male; antennal club composed of three antennomeres, as long as remaining antennomeres combined.

#### Diagnosis.

*Tetrasericafulleri* sp. n. differs from all other *Tetraserica* species in having long parameres and a long median phallobasal lamina, and by the simple left paramere being wide for its entire length (at middle nearly as wide as apex of phallobase; lateral view).

#### Etymology.

The new species is named after its collector, E Fuller (noun in genitive singular).

### Tetraserica paratonkinensis sp. n.

Taxon classificationAnimaliaColeopteraMelolonthidae

http://zoobank.org/B7A8135E-8B4A-45E2-90B1-2B951780FC46

Figures 16 , 50

#### Type material examined.

Holotype: ♂ “Coll. I. R. Sc. N. B. Vietnam, Hoang Lien N.P. 22°21'00"N, 103°46'20"E, 1-5.vii.2013 night collecting Leg. J. Constant & J. Bresseel, I.G.: 32.454/ 234 Sericini Asia spec.” (ISNB). Paratype: 1 ♂ “N Vietnam, (Tonkin) pr. Vinh Phu 1990 Tam Dao, 6-9.v. P. Pacholátko leg./ coll. P. Pacholátko/ VS80” (CPPB), 1 ♂ “Vietnam N (Sa Pa) Lao Cai Prov., 250 km from Hanoi bearin 31°, Sa Pa vill. Env. Hoang Lien Son Nat. Res., 27.5.-3.6.1998 1250 m leg. A. Naplolov” (CNAR).

#### Description.

Length of body: 9.5 mm; length of elytra: 7.4 mm; maximum width: 6.3 mm. Surface of labroclypeus and disc of frons glabrous. Smooth area anterior to eye twice as wide as long. Eyes moderately large, ratio of diameter/interocular width: 0.63. Ratio of length of metepisternum/metacoxa: 1/1.47. Metatibia moderately long and wide, ratio width/length: 1/3.69; basal group of dorsal spines of metatibia at first third of metatibial length.

Aedeagus: Fig. 16J–M. Habitus: Fig. 16N.

Female unknown.

#### Variation.

Length of body: 9.5–10.2 mm; length of elytra: 7.1–7.4 mm; maximum width: 5.8–6.3 mm.

#### Diagnosis.

*Tetrasericaparatonkinensis* sp. n. is very similar to *T.xichouensis* Liu et al., 2014 in shape of aedeagus, but differs by the left paramere being nearly uniformly curved and the median lamina of phallobase that is not narrowed in its apical half.

#### Etymology.

The species name (adjective in the nominative singular) is derived from the combined Greek word *para*- (close to) and the species name *tonkinensis*, with reference to the similarity with *T.tonkinensis* (Moser).

### Tetraserica tonkinensis

Taxon classificationAnimaliaColeopteraMelolonthidae

(Moser, 1908)

Figures 16 , 51

Neoserica tonkinensis Moser, 1908: 328.
Tetraserica tonkinensis : Liu, Fabrizi, Bai, Yang & Ahrens, 2014: 109, fig. 7E–H.

#### Material examined.

3 ♂♂ “Tonkin Montes Mauson April, Mai 2-3000’ H. Frustorfer” (MNHN), 2 ♂♂ “Tonkin Montes Mauson April, Mai 2-3000’ H Frustorfer” (CF, ZMHB).

#### Remarks.

The species was revised and redescribed in Liu et al. (2014).

Aedeagus: Fig. 16F–H. Habitus: Fig. 16I.

### Tetraserica quadriforceps sp. n.

Taxon classificationAnimaliaColeopteraMelolonthidae

http://zoobank.org/E9DDE586-65D3-4FDB-9AB3-2FAE5A00332A

Figures 17 , 51

#### Type material examined.

Holotype: ♂ “Vietnam N 1989 Tam Dao 12-24.5. Vinh Phu prov. Stranad Jan leg.” (ZFMK). Paratypes: 1 ♂ “Coll. I. R. Sc. N. B. C Vietnam, Tam Dao N.P. 21°31'N 105°33'E, 25-30.vii.2011, Malaise trap, Leg. J: Costant & J. Bresseel, I.G.: 31.933, GTI project” (ISNB), 1 ♂ “N-Vietnam (Tonkin) pr. Vinh Phu, Tam Dao. 6-9.V.1990 Vít. Kubáň leg.”/ Coll. Milan Nikodým, Praha/ 726 Sericini Asia spec.” (ZFMK), 1 ♂ “N. Vietnam, 900 m Tam Dao 13-24.5 1989 A. Olexa/ Fereiwilliger Museumsverain (Basel) 1989” (NHMB), 1 ♂ “N-Vietnam, (Tonkin) Tamdao 12-24.5.1989 PACHOLÁTKO leg./ coll. P. Pacholátko/ VS21/ 242 Sericini Asia spec.” (CPPB), 1 ♂ “Vietnam, Tam dao 27.5.-2.6.1986 Vinh phu prov. Jan Horák lgt.” (NHMB), 2 ♂♂ “N-Vietnam 5-10.VI.1989 Tam Dao Brantlová lgt.” (NHMB), 1 ♂ “N. Vietnam: Vinh Phu prov., Tam Dao 900 m, 14.V.1962 leg. O. Kabakov” (ZIN).

#### Description.

Length of body: 9.9 mm; length of elytra: 7 mm; maximum width: 6.1 mm. Surface of labroclypeus and disc of frons glabrous. Smooth area anterior to eye twice as wide as long. Eyes moderately large, ratio of diameter/interocular width: 0.65. Ratio of length of metepisternum/metacoxa: 1/1.26. Metatibia short and wide, ratio width/length: 1/2.6; basal group of dorsal spines of metatibia at first third of metatibial length.

**Figure 17. A–E:**
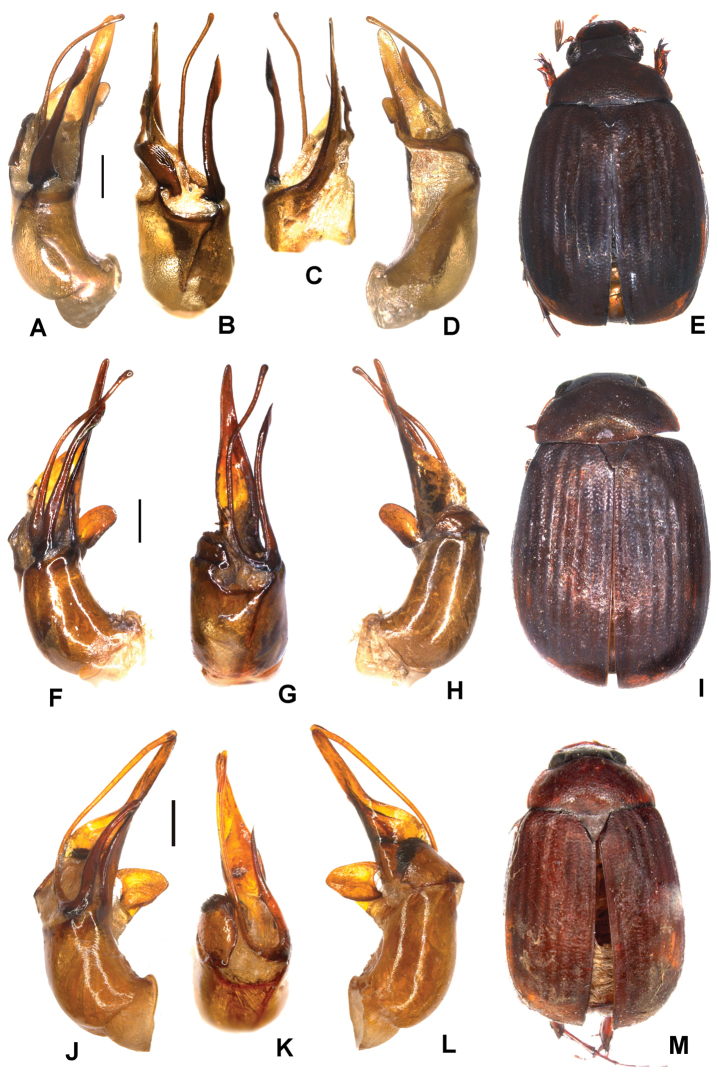
*Tetrasericaquadriforceps* sp. n. (holotype) **F–I***T.allochangshouensis* sp. n. (holotype) **J–M***T.changshouensis* Liu et al., 2014 (holotype) **A, F, J** aedeagus, left side lateral view **D, H, L** aedeagus, right side lateral view **B, G, K** parameres, dorsal view **C** parameres, ventral view **E, I, M** habitus. Scale bars: 0.5 mm. Habitus not to scale.

Aedeagus: Fig. 17A–D. Habitus: Fig. 17E.

Female unknown.

#### Variation.

Length of body: 8.0–9.9 mm; length of elytra: 6.2–7.0 mm; maximum width: 5.0–6.1 mm.

#### Diagnosis.

*Tetrasericaquadriforceps* sp. n. differs from all other *Tetraserica* species with a median lamina of the phallobase that is longer than the half-length of phallobase (but not exceeding the length of it), and by the right paramere having a comb of short spines at base. The shape of aedeagus resembles that of *T.changshouensis* Liu et al., 2014, but the medial phallobasal lamina is much shorter and the apex of the right paramere less strongly widened.

#### Etymology.

The species name (noun in apposition) is derived from the combined Latin prefix *quadri* (four) and *forceps*, with reference to the four branches of the parameres.

### Tetraserica allochangshouensis sp. n.

Taxon classificationAnimaliaColeopteraMelolonthidae

http://zoobank.org/A1F52B84-2605-4B04-894E-652F3438CFF5

Figures 17 , 46

#### Type material examined.

Holotype: ♂ “Tonkin Montes Mauson April, Mai 2-3000’ H. Frustorfer/ 1005 Asia Sericini spec.” (MNHN). Paratype: 1 ♂ “Tonkin Montes Mauson April, Mai 2-3000’ H. Frustorfer” (ZFMK).

#### Description.

Length of body: 8.8 mm; length of elytra: 6.6 mm; maximum width: 5.6 mm. Surface of labroclypeus and disc of frons glabrous. Smooth area anterior to eye twice as wide as long. Eyes moderately large, ratio of diameter/interocular width: 0.56. Ratio of length of metepisternum/metacoxa: 1/1.5. Metatibia short and wide, ratio width/length: 1/3.27; basal group of dorsal spines of metatibia at first third of metatibial length.

Aedeagus: Fig. 17F–H. Habitus: Fig. 17I.

Female unknown.

#### Diagnosis.

The new species is close to the Chinese species *T.changshouensis* Liu et al., 2014, but differs by the narrower ventroapical lobe of right paramere and the longer ventral lobe of left paramere.

#### Variation.

Length of body: 8.2–8.8 mm; length of elytra: 6.5–6.6 mm; maximum width: 5.0–5.6 mm.

#### Etymology.

The species name (adjective in the nominative singular) is derived from the combined Greek prefix *allo*- (other) and the species name *changshouensis*, with reference to the similarity to *T.changshouensis*.

### Tetraserica changshouensis

Taxon classificationAnimaliaColeopteraMelolonthidae

Liu, Fabrizi, Bai, Yang & Ahrens, 2014

Figures 17 , 47

Tetraserica changshouensis Liu, Fabrizi, Bai, Yang & Ahrens, 2014: 112, fig. 8E–H.

#### Material examined.

1 ♂ “China, S- Guizhou, 30.V.-11.VI. Yaorenshan For. Park 25°55'N, 107°58'E, 1100 m Jaroslav Turna leg., 2001” (ZFMK).

Aedeagus: Fig. 17J–L. Habitus: Fig. 17M.

### Tetraserica dongnaiensis sp. n.

Taxon classificationAnimaliaColeopteraMelolonthidae

http://zoobank.org/D8CAB95A-F929-4317-8171-EF9B29CBF6D7

Figures 18 , 47

#### Type material examined.

Holotype: ♂ “Museum Leiden Viet Nam (Dong Nai Prov.) Cát Tiên N.P.: along Dong trail, 9.iv-19.v.2007. Leg. C. van Achterberg, R. de Vries, E. Gassó, Miracle & Nguyen Than Mahn/ humid lowland forest; in malaise traps; 250 m. 11°26'20.6"N, 107°25'42"E/ 960 Sericini Asia spec.” (RMNH). Paratypes: 4 ♂♂ “Museum Leiden Viet Nam (Dong Nai Prov.) Cát Tiên N.P.: along Dong trail, 9.iv-19.v.2007. Leg. C. van Achterberg, R. de Vries, E. Gassó, Miracle & Nguyen Than Mahn/ humid lowland forest; in malaise traps; 250 m. 11°26'20.6"N, 107°25'42"E” (RMNH, ZFMK), 2 ♂♂ “Museum Leiden Viet Nam (Dong Nai Prov.) Cát Tiên N.P.: along Dong trail, 1.iv-13.v.2007. Leg. May Phu Quy, Nguyen Than Mahn/ humid lowland forest; in malaise traps; 250 m, 11°26'20.6"N, 107°25'42"E” (RMNH).

#### Description.

Length of body: 10 mm; length of elytra: 7.5 mm; maximum width: 6.4 mm. Surface of labroclypeus and disc of frons glabrous. Smooth area anterior to eye twice as wide as long. Eyes moderately large, ratio of diameter/interocular width: 0.62. Ratio of length of metepisternum/metacoxa: 1/1.61. Metatibia short and wide, ratio width/length: 1/2.36; basal group of dorsal spines of metatibia at first third of metatibial length.

**Figure 18.**
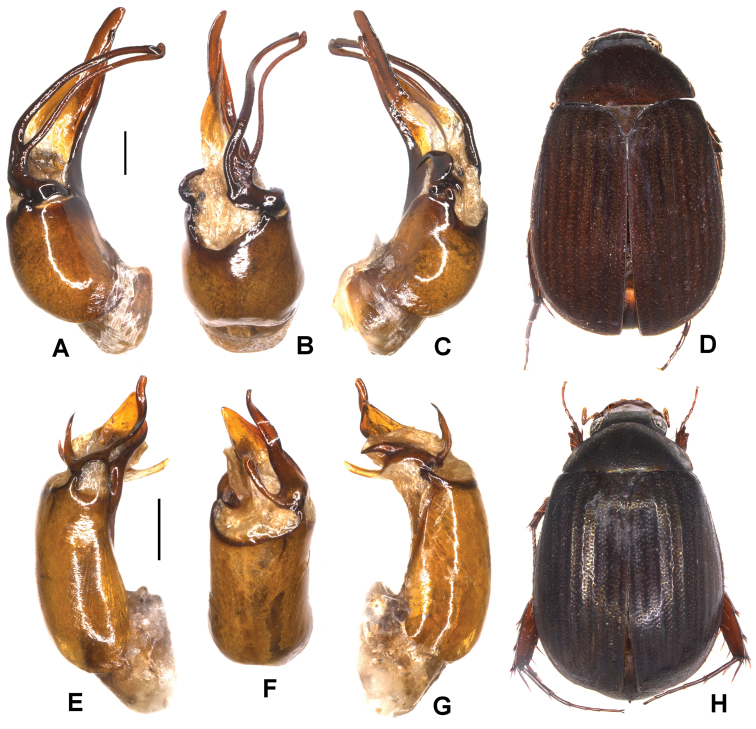
**A–D***Tetrasericadongnaiensis* sp. n. (holotype) **E–H***T.phatoensis* sp. n. (holotype) **A, E** aedeagus, left side lateral view **C, G** aedeagus, right side lateral view **B, F** parameres, dorsal view **D, H** habitus. Scale bars: 0.5 mm. Habitus not to scale.

Aedeagus: Fig. 18A–C Habitus: Fig. 18D.

Female unknown.

#### Variation.

Length of body: 9.1–10.5 mm; length of elytra: 6.9–7.9 mm; maximum width: 5.8–6.8 mm.

#### Diagnosis.

*Tetrasericadongnaiensis* sp. n. differs from all other *Tetraserica* species that have long median phallobasal lamina with simple left and simple right parameres at base (without brush of spines), by the left paramere being split behind basal third into two filiform branches, and the right paramere being short and curved.

#### Etymology.

The new species is named with reference to its occurrence in the Dong Nai province (adjective in the nominative singular).

### Tetraserica phatoensis sp. n.

Taxon classificationAnimaliaColeopteraMelolonthidae

http://zoobank.org/410729C5-9321-4536-8CF1-8113E4B0B9DE

Figures 18 , 49

#### Type material examined.

Holotype: ♂ “Thailand, Chumphon prov., 27.iii-14.iv.1996 Pha To env. 9°48’ 98”47, P. Průdek leg./ coll. P. Pacholátko/ 150 Sericini Asia spec.” (CPPB).

#### Description.

Length of body: 8.3 mm; length of elytra: 6.1 mm; maximum width: 5.3 mm. Dorsal surface blackish, ventral surface reddish brown. Surface of labroclypeus and disc of frons glabrous. Smooth area anterior to eye twice as wide as long. Eyes small, ratio of diameter/interocular width: 0.43. Ratio of length of metepisternum/metacoxa: 1/0.04. Metatibia short and wide, ratio width/length: 1/3.07; basal group of dorsal spines of metatibia at first third of metatibial length.

Aedeagus: Fig. 18E–G. Habitus: Fig. 18H.

Female unknown.

#### Diagnosis.

*Tetrasericaphatoensis* sp. n. differs from all other *Tetraserica* species with very short median lamina of the phallobase (one quarter of phallobase length) and a long and narrow, more or less straight, left paramere, and by the right paramere having divergent spines at the base and a separate lobe distally.

#### Etymology.

The new species is named after the type locality, Pha To (adjective in the nominative singular).

### Tetraserica masumotoi

Taxon classificationAnimaliaColeopteraMelolonthidae

Kobayashi, 2017

Figures 19 , 50

Tetraserica masumotoi Kobayashi, 2017: 40, figs 6, 15.

#### Material examined.

1 ♂ “NW Thailand, 19.19N, 97.59E, Mae Hong Son, 1991 Ban Huai Po, 1600–2000 m, 17-23.5., L. Dembický leg./ 600 Sericini Asia spec.” (NHMW), 1 ♂ “N-Thailand Ban Mai [Huai] Po 9.-16.5.1991 L. Horak lgt./ Coll. Milan Nikodym Praha” (ZFMK), 1 ♂ “Thailand 9.-16.5.1991 Mae Hong Son Ban Huai Po 1600–2000 m J. Horak lgt./ coll. Milan Nikodym, Praha” (ZFMK).

**Redescription.** Length of body: 8.5 mm; length of elytra: 6 mm; maximum width: 5.3 mm. Surface of labroclypeus and disc of frons glabrous. Smooth area anterior to eye twice as wide as long. Eyes moderately large, ratio of diameter/interocular width: 0.63. Ratio of length of metepisternum/metacoxa: 1/1.56. Posterior margin of metafemur with sharp hook. Metatibia short and wide, ratio width/length: 1/3.29; basal group of dorsal spines of metatibia at first third of metatibial length.

**Figure 19.**
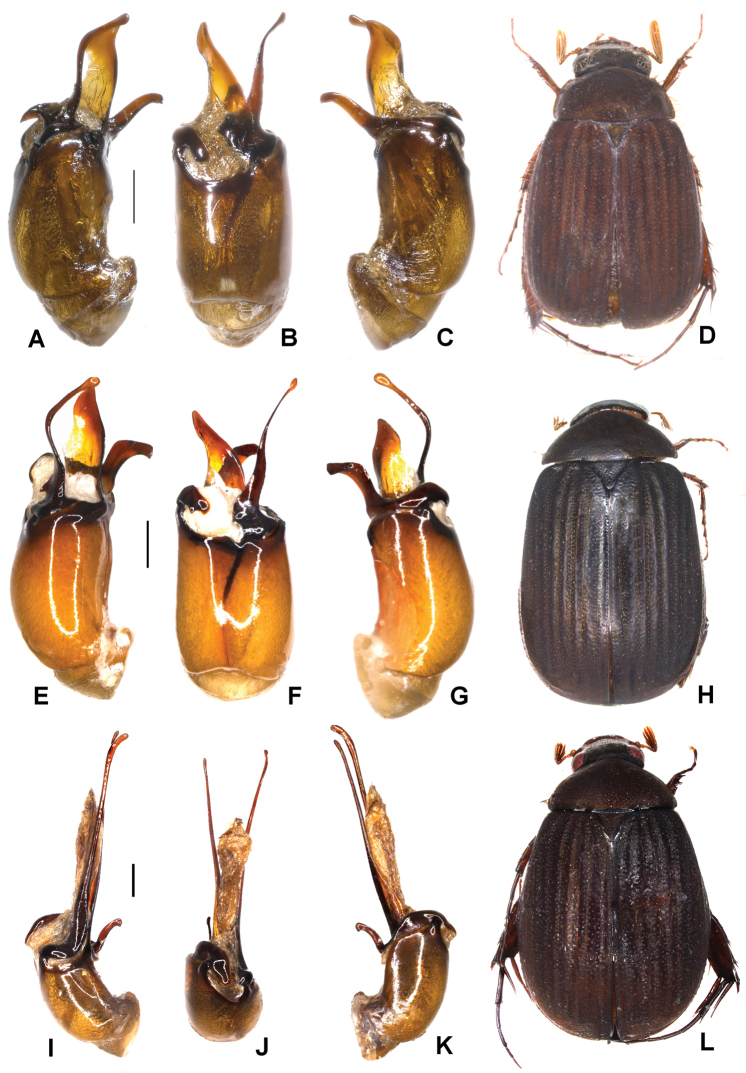
**A–D***Tetrasericamasumotoi* Kobayashi, 2017 (Thailand, Mae Hong Son) **E–H***T.wiangpapaoana* Kobayashi, 2017 (Thailand, Doi Inthanon) **I–L***T.spinotibialis* sp. n. (holotype) **A, E, I** aedeagus, left side lateral view **C, G, K** aedeagus, right side lateral view **B, F, J** parameres, dorsal view **D, H, L** habitus. Scale bars: 0.5 mm. Habitus not to scale.

Aedeagus: Fig. 19A–C. Habitus: Fig. 19D.

### Tetraserica wiangpapaoana

Taxon classificationAnimaliaColeopteraMelolonthidae

Kobayashi, 2017

Figures 19 , 55

Tetraserica wiangpapaoana Kobayashi, 2017: 41, figs 7, 16.

#### Material examined.

1 ♂ “X-DA2306 labcode: VD002 Thailand N.: Doi Inthanon Nat. Res., 1250m 22-31.v.2008, S. Murzin leg. Tetraserica spTHAIx1/ X-DA2306/ sp-THAIx1V” (ZFMK), 1 ♂ “Thai 24-29.IV.1993, DOI SUTHEP Pacholátko & Dembický leg.”/ coll. P. Pacholátko/ 142 Sericini Asia spec.” (CPPB), 1 ♂ “NW Thai, 7-14.V.1992, DOI SUTHEP PUI 1300–1500 m leg. P. Pacholátko/ coll. P. Pacholátko/ 137 Sericini Asia spec.” (CPPB), 1 ♂ “Thailand: 29.5.-5.6.1989 Dai [Doi] Inthanon, Lichtfalle, Bang Khun Klang 1200 m 98°32'E, 18°32'N, Chantaramongkol & Malicky leg.” (ZSM), 3 ♂♂ “N-Thailand V.1990 Doi Inthanon lg. Malicky” (ZSM), 1 ♂ “836048 Thailand N. Thailand: Doi Inthanon Nat. Res., 1250 m, 22-31.v.2008, S. Murzin leg. Tetraserica spTHAIx1” (ZFMK), 1 ♂ “836046 Thailand N. Thailand: Doi Inthanon Nat. Res., 1250m 22-31.v.2008, S. Murzin leg. Tetraserica spTHAIx1” (ZFMK).

**Redescription.** Length of body: 9.5 mm; length of elytra: 7.6 mm; maximum width: 5.6 mm. Surface of labroclypeus and disc of frons glabrous. Smooth area anterior to eye twice as wide as long. Eyes moderately large, ratio of diameter/interocular width: 0.67. Ratio of length of metepisternum/metacoxa: 1/1.42. Posterior margin of metafemur with sharp hook. Metatibia moderately long and wide, ratio width/length: 1/4.23; basal group of dorsal spines at first third of metatibial length.

Aedeagus: Fig. 19E–G. Habitus: Fig. 19H.

### Tetraserica spinotibialis sp. n.

Taxon classificationAnimaliaColeopteraMelolonthidae

http://zoobank.org/7E06B2BC-5645-4FF7-B660-1D4A9B0444A4

Figures 19 , 56

#### Type material examined.

Holotype: ♂ “NW Thailand, 9-16.V. MAE HONG SON. 1991 Ban Huai Po 1600 m leg. P. Pacholátko/ coll. P. Pacholátko/ 918 Sericini Asia spec.” (CPPB). Paratypes: 1 ♂ “NW Thailand, 1-8.V. MAE HONG SON 1992 BAN SI LANG 1200 m J. HORAK LEG./ coll. P. Pacholátko” (ZFMK), 4 ♂♂ “NW Thailand, 19.19N, 97.59E Mae Hong Song, 1991 Ban Huai Po, 1600–2000 m 17.-23.5., L. Dembicky leg.” (NHMW, ZFMK), 2 ♂♂ “NW Thailand, 19.19N, 97.59E Mae Hong Song, 1991 Ban Si Lang, 1200 m 23.-31.5., L. Dembicky leg.” (NHMW), 1 ♂ “N-Thailand Ban Mai [Huai] Po 9.-16.5.1991 L. Horak lgt./ Coll. Milan Nikodym Praha” (ZFMK), 3 ♂♂, 1 ♀ “Birmania Lashio VI-53 Bentoglio” (MSNM).

#### Description.

Length of body: 9 mm; length of elytra: 5.8 mm; maximum width: 5.8 mm. Surface of labroclypeus and disc of frons glabrous. Smooth area anterior to eye twice as wide as long. Eyes moderately large, ratio of diameter/interocular width: 0.71. Ratio of length of metepisternum/metacoxa: 1/1.72. Metatibia short and wide, ratio width/length: 1/2.05; basal group of dorsal spines of metatibia at first third of metatibial length. Ventral terminal spine of metatibia distinctly longer than first metatarsomere.

Aedeagus: Fig. 19I–K. Habitus: Fig. 19L.

#### Variation.

Length of body: 9.0–9.9 mm; length of elytra: 5.8–7.0 mm; maximum width: 5.5–5.8 mm. Female: Eyes as large as in male; antennal club composed of three antennomeres, as long as remaining antennomeres combined.

#### Diagnosis.

Aedeagus of *Tetrasericaspinotibialis* sp. n. resembles that of *T.ruiliensis* Liu et al., 2014 in shape. The new species differs by the ventral terminal spine of metatibia being distinctly longer than first metatarsomere, as well as by the right paramere being more strongly bent in the middle in lateral view.

#### Etymology.

The species name (adjective in the nominative singular) is derived from the combined Latin words *spina* and *tibialis*, with reference to the strongly developed spine of the mesotibia.

### Tetraserica vari sp. n.

Taxon classificationAnimaliaColeopteraMelolonthidae

http://zoobank.org/F2C63CEC-6502-4999-B3C9-C0F5CEBF53BB

Figures 20 , 53

#### Type material examined.

Holotype: ♂ “Coll. I. R. Sc. N. B. Cambodia, Pursat Prov., Phnom Samkos W.S., forest edge, light trap 14.iv.2005 Leg. K. Smets & I. Var” (ISNB). Paratypes: 1 ♂ “Coll. I. R. Sc. N. B., Cambodia, Pursat Prov., Phnom Samkos, W.S. Pramaoy, edge forest, Light trapping/ 16-XI-2005 Leg. K. Smets & I. Var” (ISNB), 1 ♂ “Coll. I. R. Sc. N. B. Cambodia Kirirom N. P. Light Trap Pine forest, 21 IV 2005 Leg. Smets & I. Var” (ISNB), 2 ♂♂ “Coll. I. R. Sc. N. B., Cambodia, Pursat Prov., Phnom Samkos Wildlife Pramaoy 16.XI.2005 forest edge, Light trapping, Leg. K. Smets & I. Var” (ISNB), 3 ♂♂ “Coll. I. R. Sc. N. B., Cambodia, Pursat prov. Phnom Samkos W.S., forest edge, light trap 14.iv.2005 Leg. K. Smets & I. Var” (ISNB, ZFMK), 1 ♂ “Coll. I. R. Sc. N. B. Cambodia (Pursat Prov.) Phnom Samkos wild life Sanctuary, light trap Forest Edge & Primary 14-IV-2005 Leg. K. Smets & I. Var” (ZFMK), 1 ♂ “Coll. I. R. Sc. N. B. Cambodia, Pursat Prov Phnom Samkos W.S., Pramaoy Forest Edge 15-IV-2005, Light Trap Leg. K. Smets & I. Var” (ISNB), 1 ♂ “E Thailand, 5-13.5. Chanthaburi Dist. Khao Soi Dao, 1998, M. Knížek lgt./ coll. P. Pacholátko” (CPPB), 1 ♂ “Cambodia, Cardamom Mts., near Pramuoy village, 12°16'N, 103°01'E, 270 m, Dry Dipterocarp and Gallery Forest, 24-25.ii.2000, leg. M. Nuss/ Staatl. Museum für Tierkunde Dresden/ 118 Sericini Asia spec.” (SMTD).

#### Description.

Length of body: 10.4 mm; length of elytra: 8 mm; maximum width: 6.6 mm. Surface of labroclypeus and disc of frons glabrous. Smooth area anterior to eye twice as wide as long. Eyes moderately large, ratio of diameter/interocular width: 0.66. Ratio of length of metepisternum/metacoxa: 1/1.69. Metatibia short and wide, ratio width/length: 1/2.62; basal group of dorsal spines of metatibia at first third of metatibial length.

**Figure 20.**
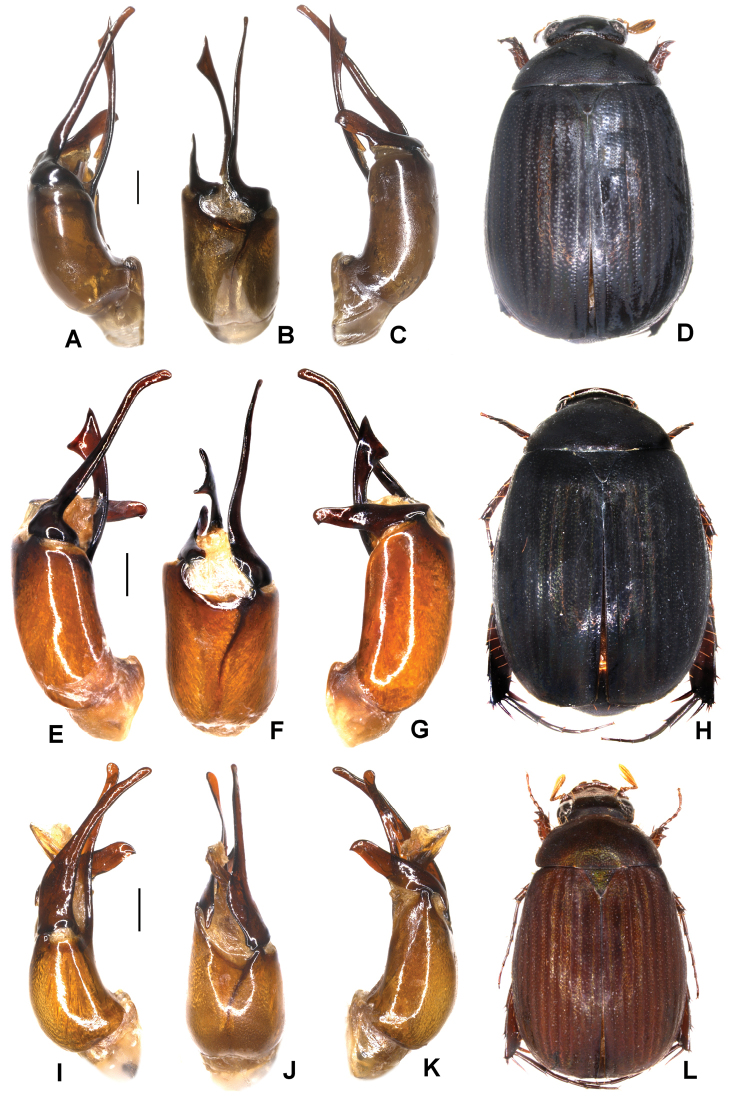
**A–D***Tetrasericavari* sp. n. (holotype) **E–H***T.constanti* sp. n. (holotype) **I–L***T.vientianeensis* sp. n. (holotype) **A, E, I** aedeagus, left side lateral view **C, G, K** aedeagus, right side lateral view **B, F, J** parameres, dorsal view **D, H, L** habitus. Scale bars: 0.5 mm. Habitus not to scale.

Aedeagus: Fig. 20A–C. Habitus: Fig. 20D.

Female unknown.

#### Variation.

Length of body: 10.4–11.2 mm; length of elytra: 8.0–8.1 mm; maximum width: 6.6–6.9 mm.

#### Diagnosis.

*Tetrasericavari* sp. n. differs from all other *Tetraserica* species in having a long median phallobasal lamina, a basally bent left paramere, and the median phallobasal lamina widened and sharply truncated at its apex.

#### Etymology.

The new species is named after one of its collectors, I Var (noun in genitive singular).

### Tetraserica constanti sp. n.

Taxon classificationAnimaliaColeopteraMelolonthidae

http://zoobank.org/5A39A989-5418-424B-8599-0464458530C1

Figures 20 , 48

#### Type material examined.

Holotype: ♂ “Coll. I. R. Sc. N. B. Cambodia - 8 km north of Sre Noi (road to Along Vaeng)/ Light trap 29.V.2003 Leg. J. Constant & K. Smets/ 926 Sericini Asia spec.” (ISNB). Paratype: 1 ♂ “Coll. I. R. Sc. N. B. Cambodia (Siem Road prov) Kbal Spean, Light Trap 28 V 2005 Leg Var & Grootaert” (ZFMK).

#### Description.

Length of body: 9.8 mm; length of elytra: 7.5 mm; maximum width: 6.5 mm. Surface of labroclypeus and disc of frons glabrous. Smooth area anterior to eye twice as wide as long. Eyes moderately large, ratio of diameter/interocular width: 0.63. Ratio of length of metepisternum/metacoxa: 1/1.79. Metatibia short and wide, ratio width/length: 1/2.57; basal group of dorsal spines of metatibia at first third of metatibial length.

Aedeagus: Fig. 20E–G. Habitus: Fig. 20H.

Female unknown.

#### Variation.

Length of body: 9.8–10.2 mm; length of elytra: 7.3–7.5 mm; maximum width: 6.2–6.5 mm.

#### Diagnosis.

*Tetrasericaconstanti* sp. n. is very similar to *T.vari* sp. n. in the shape of aedeagus but differs by the distinctly shorter median phallobasal lamina and parameres.

#### Etymology.

The new species is named after one of its collectors, J Constant (noun in genitive singular).

### Tetraserica vientianeensis sp. n.

Taxon classificationAnimaliaColeopteraMelolonthidae

http://zoobank.org/D7FA5448-3EDC-4924-AF48-C298C86BF208

Figures 20 , 54

#### Type material examined.

Holotype: ♂ “Laos Bolikhamxai pr., 18°16'N, 103°11'E, 70 km NEE Vientiane 27-30.iv.1997, 150 m, Vít Kubáň leg./ coll. P. Pacholátko/ 197 Sericini Asia spec.” (CPPB).

#### Description.

Length of body: 8.3 mm; length of elytra: 6 mm; maximum width: 4.9 mm. Surface of labroclypeus and disc of frons glabrous. Smooth area anterior to eye twice as wide as long. Eyes moderately large, ratio of diameter/interocular width: 0.64. Ratio of length of metepisternum/metacoxa: 1/1.65. Metatibia short and wide, ratio width/length: 1/2.93; basal group of dorsal spines of metatibia at first third of metatibial length.

Aedeagus: Fig. 20I–K. Habitus: Fig. 20L.

Female unknown.

#### Diagnosis.

*Tetrasericavientianeensis* sp. n. differs from the similar *T.constanti* sp. n. by the basally straight and unbent left paramere, and by the median lamina of phallophase which is not widened at apex.

#### Etymology.

The new species is named with reference to its close occurrence to the city of Vientiane (adjective in the nominative singular).

### Tetraserica breviforceps sp. n.

Taxon classificationAnimaliaColeopteraMelolonthidae

http://zoobank.org/F5BB7D81-329D-435F-8F0D-CE381D5D25B6

Figures 21 , 47

#### Type material examined.

Holotype: ♂ “Laos, Sekong Prov. ca. 12 km S Sekong TAD FAEK Waterfalls (at light) 15°14, 7'N, 106° 45, 1'E, 118m, Jiři Hájek leg., 8+12.v.2010/ 207 Sericini Asia spec.” (NMPC). Paratype: 1 ♂ “Laos centr., Khammouan prov. NAKAI env. 4-8.5.1998, Route No 8, alt. 560 ± 20 m, N17°42.8, E 105°08.9 (GPS), E Jendek & O Šauša leg./ coll. P Pacholátko” (CPPB), 1 ♂ “Laos, Sekong Prov. ca. 12 km S Sekong TAD FAEK Waterfalls (at light) 15°14, 7'N, 106° 45, 1'E, 118 m, Jiři Hájek leg. 8+12.v.2010/ 207 Sericini Asia spec.” (ZFMK).

#### Description.

Length of body: 8 mm; length of elytra: 6 mm; maximum width: 5.6 mm. Surface of labroclypeus and disc of frons glabrous. Smooth area anterior to eye twice as wide as long. Eyes moderately large, ratio of diameter/interocular width: 0.74. Ratio of length of metepisternum/metacoxa: 1/2.08. Metatibia short and wide, ratio width/length: 1/2.44; basal group of dorsal spines of metatibia at first third of metatibial length.

**Figure 21.**
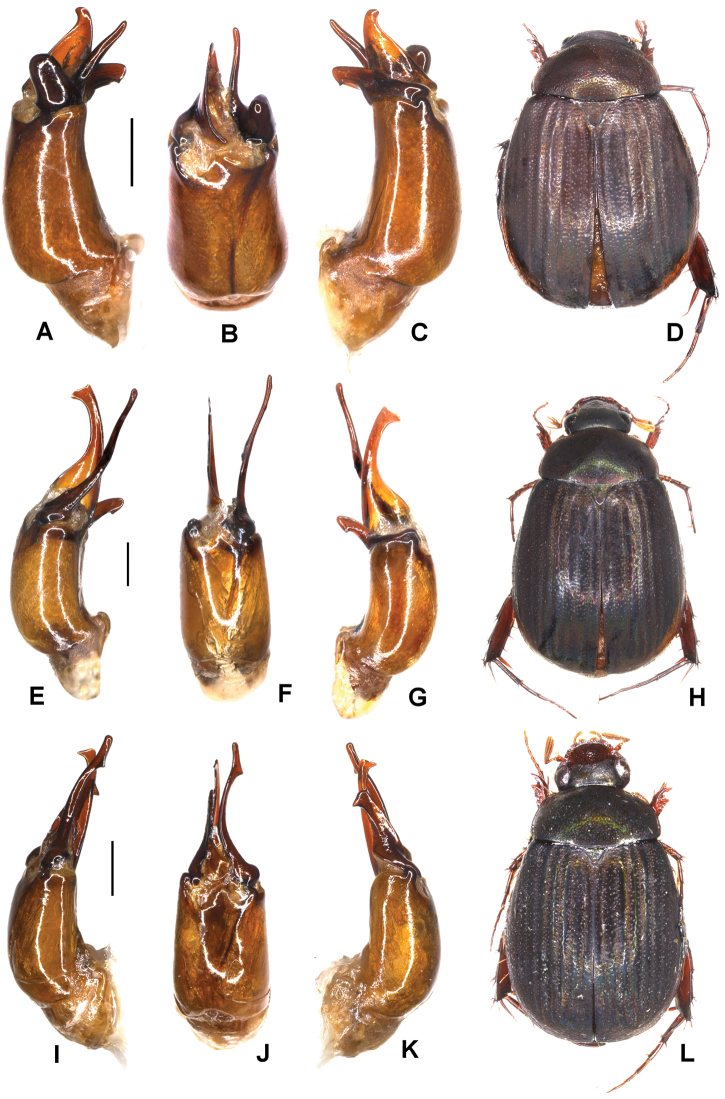
**A–D***Tetrasericabreviforceps* sp. n. (holotype) **E–H***T.nahaeoensis* sp. n. (holotype) **I–L***T.champassakana* sp. n. (holotype) **A, E, I** aedeagus, left side lateral view **C, G, K** aedeagus, right side lateral view **B, F, J** parameres, dorsal view **D, H, L** habitus. Scale bars: 0.5 mm. Habitus not to scale.

Aedeagus: Fig. 21A–C. Habitus: Fig. 21D.

Female unknown.

#### Variation.

Length of body: 8.0–9.2 mm; length of elytra: 6.0–6.5 mm; maximum width: 5.5–5.6 mm.

#### Diagnosis.

*Tetrasericabreviforceps* sp. n. differs from all other *Tetraserica* species with its short parameres and very short median phallobasal lamina, and by the left paramere having a robust (nearly half as long as paramere), subsphaerical, and apically convex dorsal lobe at its base.

#### Etymology.

The name of the new species (noun in apposition) is derived from the combined Latin words *brevis* (short) and *forceps*, with reference to the short parameres.

### Tetraserica nahaeoensis sp. n.

Taxon classificationAnimaliaColeopteraMelolonthidae

http://zoobank.org/CA0A4BE2-C4D9-43BA-BDE2-89EC261A1AB2

Figures 21 , 49

#### Type material examined.

Holotype: ♂ “Coll. I. R. Sc. N. B. THAILAND (loei), Na-Haeo (field res stat) 15-19.V.2003 Light trap Leg. J. Constant, K. Smets & P. Grootaert/ 143 Sericini Asia spec.” (ISNB). Paratypes: 1 ♂ “Coll. I. R. Sc. N. B. THAILAND (loei), Na-Haeo (field res stat) 15-19.V.2003 Light trap Leg. J. Constant, K. Smets & P. Grootaert” (ISNB), 1 ♂ “Coll. I. R. Sc. N. B. THAILAND (Loei Prov.) Na-Haeo (Field Res. St.) 15-19.V.2003 Light trap Leg Constant & K. Smets” (ZFMK), 1 ♂ “Coll. I. R. Sc. N. B. THAILAND (Loei) Na-Haeo (edge pond) Light trap 17.V.2003 Leg. J. Constant, K. Smets” (ISNB), 1 ♂ “Coll. I. R. Sc. N. B. Thailand, Loei Na Haeo 22/V/2000 Station Leg. P. Grootaert” (ZFMK), 1 ♂ “Coll. I. R. Sc. N. B. Thailand (Loei) Na Haeo 23/05/1998 Leg. P. Grootaert” (ISNB), 1 ♂ “Thai 21-26.V.1993, Namuang Pacholátko & Dembicky leg./ coll. P. Pacholátko/ TS92” (CPPB).

#### Description.

Length of body: 9.6 mm; length of elytra: 6.9 mm; maximum width: 6 mm. Surface of labroclypeus and disc of frons glabrous. Smooth area anterior to eye twice as wide as long. Eyes small, ratio of diameter/interocular width: 0.55. Ratio of length of metepisternum/metacoxa: 1/1.77. Metatibia short and wide, ratio width/length: 1/3.12; basal group of dorsal spines of metatibia at first third of metatibial length.

Aedeagus: Fig. 21E–G. Habitus: Fig. 21.

Female unknown.

#### Variation.

Length of body: 8.4–9.6 mm; length of elytra: 6.5–6.9 mm; maximum width: 5.9–6.0 mm.

#### Diagnosis.

*Tetrasericanahaeoensis* sp. n. differs from all other *Tetraserica* species in having a moderately long median phallobasal lamina being narrowed distally, curved dorsally, and having a dorsal tooth at apex; simple left parameres, the shorter right paramere (being one third as long as phallobase), the nearly straight left paramere.

#### Etymology.

The new species is named after the type locality, Na-Haeo (adjective in the nominative singular).

### Tetraserica champassakana sp. n.

Taxon classificationAnimaliaColeopteraMelolonthidae

http://zoobank.org/29A7B159-942A-4762-A24C-394BCE2F9DA8

Figures 21 , 46

#### Type material examined.

Holotype: ♂ “Laos, Champassak Prov., Dong Hua Xao NBCA bank of Nam Phak river, 15°59'N, 105°55'E/ 280 m, at light, No. 14, 28-29.III.1998 leg. O Merkl & G Csorba” (HNHM). Paratype: 1 ♂ “Laos, Champassak Prov., Dong Hua Xao NBCA bank of Nam Phak river, 15°59'N, 105°55'E/ 280 m, at light, No. 14, 28-29.III.1998 leg. O Merkl & G Csorba” (ZFMK).

#### Description.

Length of body: 8.8 mm; length of elytra: 5.9 mm; maximum width: 5.3 mm. Surface of labroclypeus and disc of frons glabrous. Smooth area anterior to eye twice as wide as long. Eyes small, ratio of diameter/interocular width: 0.5. Ratio of length of metepisternum/metacoxa: 1/1.73. Metatibia short and wide, ratio width/length: 1/2.93; basal group of dorsal spines of metatibia at first third of metatibial length.

Aedeagus: Fig. 21I–K. Habitus: Fig. 21L.

Female unknown.

#### Variation.

Length of body: 7.2–8.8 mm; length of elytra: 5.4–5.9 mm; maximum width: 4.5–5.3 mm.

#### Diagnosis.

*Tetrasericachampassakana* sp. n. is rather similar to *T.nahaeoensis* sp. n. in the shape of the aedeagus but differs by the longer right paramere being half as long as phallobase.

#### Etymology.

The new species is named with reference to its occurrence in Champassak province (adjective in the nominative singular).

### Tetraserica cattienensis sp. n.

Taxon classificationAnimaliaColeopteraMelolonthidae

http://zoobank.org/244038AC-E7EE-4531-AA94-7439E68B0D81

Figures 22 , 46

#### Type material examined.

Holotype: ♂ “Museum Leiden Viet Nam (Dong Nai Prov.) Cát Tiên NP: Ficus trail. 9-26.iv.2007. leg. May Phu Quy, Nguyen Than Mahn/ humid lowland forest; in malaise traps; 250 m 11°26'20, 6"N, 107°25'42"E” (RMNH). Paratypes: 1 ♂ “Museum Leiden Viet Nam (Dong Nai Prov.) Cát Tiên N.P.: Ficus trail. 9-26.iv.2007. leg. May Phu Quy, Nguyen Than Mahn/ humid lowland forest; in malaise traps; 250 m 11°26'20, 6"N, 107°25'42"E” (ZFMK), 1 ♂ “Museum Leiden Viet Nam (Dong Nai Prov.) Cát Tiên N.P.: Ficus trail. 10.iv-15.v.2007. Leg. May Phu Quy, Nguyen Than Mahn/ humid lowland forest; in malaise traps; 250 m. 11°26'20, 6"N, 107°25'42"E” (RMNH), 1 ♂ “S Vietnam, 1-5.5.1994 Nam Cat Tien Nat. park P. Pacholátko & L. Dembický leg./ coll. P. Pacholátko/VS 118” (CPPB).

#### Description.

Length of body: 8.4 mm; length of elytra: 6 mm; maximum width: 5 mm. Surface of labroclypeus and disc of frons glabrous. Smooth area anterior to eye twice as wide as long. Eyes moderately large, ratio of diameter/interocular width: 0.67. Ratio of length of metepisternum/metacoxa: 1/1.61. Metatibia short and wide, ratio width/length: 1/2.35; basal group of dorsal spines of metatibia at first third of metatibial length.

**Figure 22.**
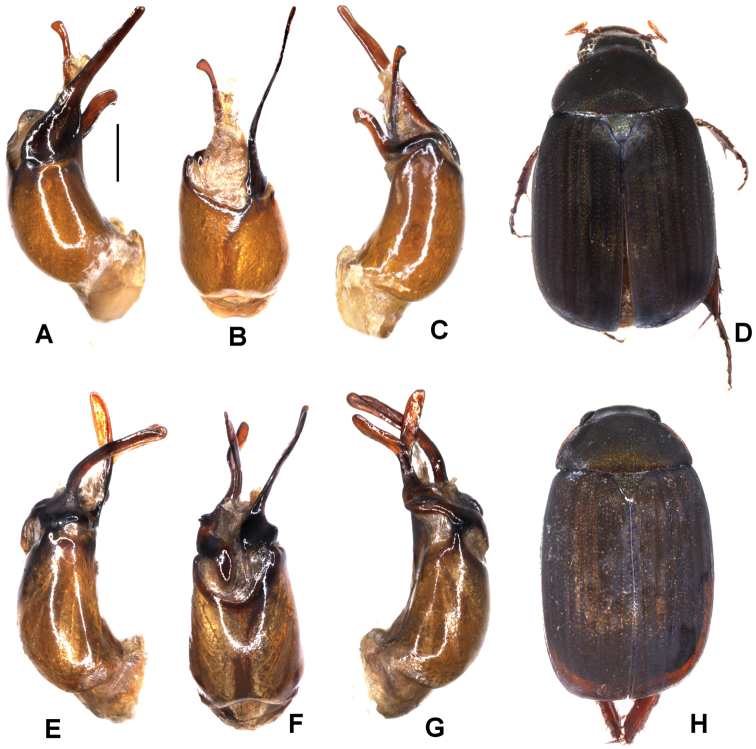
**A–D***Tetrasericacattienensis* sp. n. (holotype) **E–H***T.multiangulata* sp. n. (holotype) **A, E** aedeagus, left side lateral view **C, G** aedeagus, right side lateral view **B, F** parameres, dorsal view **D, H** habitus. Scale bars: 0.5 mm. Habitus not to scale.

Aedeagus: Fig. 22A–C. Habitus: Fig. 22D.

Female unknown.

#### Variation.

Length of body: 7.9–8.4 mm; length of elytra: 5.9–6 mm; maximum width: 4.9–5.0 mm.

#### Diagnosis.

*Tetrasericacattienensis* sp. n. differs from all other *Tetraserica* species in having a straight or slightly convex posterior margin of the metafemur, small eyes, and the short median phallobasal lamina being half as long as phallobase. From the similar *T.bansanpakiana* sp. n. and *T.margheritae* sp. n., *T.cattienensis* sp. n. differs by the robust spines on the ventral margin of metatibia being subequal in length.

#### Etymology.

The new species is named after the type locality, Cát Tiên NP (adjective in the nominative singular).

### Tetraserica multiangulata sp. n.

Taxon classificationAnimaliaColeopteraMelolonthidae

http://zoobank.org/39A606B9-CB46-4D7B-BED6-CCBCA31DBC11

Figures 22 , 49

#### Type material examined.

Holotype: ♂ “N. Vietnam: 40 km NE Thainguyen, 300 m, 14.V.1963, leg. O. Kabakov” (ZIN).

#### Description.

Length of body: 7.6 mm; length of elytra: 6 mm; maximum width: 4.5 mm. Surface of labroclypeus and disc of frons glabrous. Smooth area anterior to eye twice as wide as long. Eyes small, ratio of diameter/interocular width: 0.53. Ratio of length of metepisternum/metacoxa: 1/1.5. Posterior margin of metafemur with blunt tooth. Metatibia moderately long and wide, ratio width/length: 1/3.31; basal group of dorsal spines of metatibia at first third of metatibial length.

Aedeagus: Fig. 22E–G. Habitus: Fig. 22H.

Female unknown.

#### Diagnosis.

*Tetrasericamultiangulata* sp. n. differs by all other *Tetraserica* species by the blunt tooth at the posterior margin of metafemur, by the long and narrow left paramere, by the median lamina of phallobase being as long or nearly as long as phallobase, and by the left paramere being more than half as long as phallobase; furthermore, the left paramere is split shortly before the apex into two filiform but flattened branches.

#### Etymology.

The name of the new species (adjective in the nominative singular) is derived from the combined Latin words *multi* (numerous) and *angulatus* (angled), with reference to the parameres being bent numerous times.

### Tetraserica tanahrataensis sp. n.

Taxon classificationAnimaliaColeopteraMelolonthidae

http://zoobank.org/C8530975-DCD8-41D8-AE52-845DBBDF0990

Figures 23 , 51

#### Type material examined.

Holotype: ♂ “Malaysia-W, Pahang, 30 km E of IPOH, 1500 m Camerons Highlands, TANAHRATA, 7-9.i.1999, P. Čechovský leg./ 928 Sericini Asia spec.” (ZFMK). Paratype: 1 ♂ “X-DA0347 labcode: VD1, Malaysia-W, Kelantan Road between Kampong Raja and Gua Musang, 04°63'N, 101°, 45'E/ 04°88'N, 101°95'E, 1-28.iv.2006, P. Cechovsky leg. Tetraserica sp?1/ X-DA0347/ sp-MA1” (ZFMK), 1 ♂ “X-DA3135 Malaysia: Pahang, Tanah Rata (at light), 4°28'20"N, 101°22'42"E, 8.iii.2010, leg. P. Sipek H. Sipkova” (ZFMK), 1 ♂ “Malaysia, Pahang Cameron Highlands, 2 km S Tanah Rata on Tapah road,/ montane rainforest at light No 93 29.III.1995 O. Merkl & I. Szikossy” (HNHM).

#### Description.

Length of body: 9.6 mm; length of elytra: 7.4 mm; maximum width: 6.1 mm. Surface of labroclypeus and disc of frons glabrous. Smooth area anterior to eye twice as wide as long. Eyes small, ratio of diameter/interocular width: 0.53. Ratio of length of metepisternum/metacoxa: 1/1.48. Metatibia short and wide, ratio width/length: 1/3.25; basal group of dorsal spines of metatibia at first third of metatibial length.

**Figure 23.**
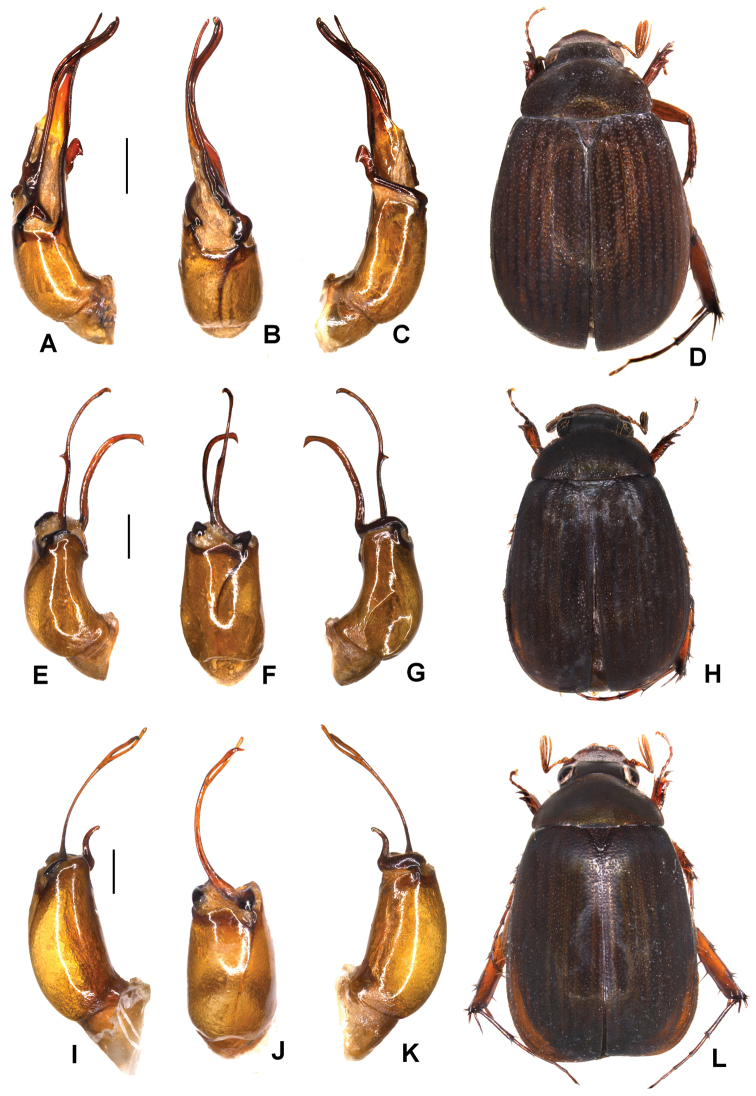
**A–D***Tetrasericatanahrataensis* sp. n. (holotype) **E–H***T.microspinosa* sp. n. (holotype) **I–L***T.microfurcata* sp. n. (holotype) **A, E, I** aedeagus, left side lateral view **C, G, K** aedeagus, right side lateral view **B, F, J** parameres, dorsal view **D, H, L** habitus. Scale bars: 0.5 mm. Habitus not to scale.

Aedeagus: Fig. 23A–C. Habitus: Fig. 23D.

Female unknown.

#### Variation.

Length of body: 9.6–9.9 mm; length of elytra: 7.4–7.5 mm; maximum width: 6.0–6.1 mm.

#### Diagnosis.

*Tetrasericatanahrataensis* sp. n. is rather similar to *T.mengeana* Liu et al., 2014 in the general shape of the aedeagus; the new species differs by lacking trichome-like spines at the base of the right paramere, and the less strongly curved left paramere, which is nearly straight in the basal two thirds.

#### Etymology.

The new species is named after the type locality, Tanahrata (adjective in the nominative singular).

### Tetraserica microspinosa sp. n.

Taxon classificationAnimaliaColeopteraMelolonthidae

http://zoobank.org/F762B902-3FCE-4090-BAFE-96FBF7027015

Figures 23 , 50

#### Type material examined.

Holotype: ♂ “N Vietnam - Lao Cai province, Van Ban district: Van Ban Nature Reserve- (~1000 m)- 23-26.V.2011/ L. Bartolozzi, S. Bambi, F. Fabiano, E. Orbach leg./ 934 Sericini Asia spec.” (VNMN).

#### Description.

Length of body: 7.5 mm; length of elytra: 5.8 mm; maximum width: 5 mm. Surface of labroclypeus and disc of frons glabrous. Smooth area anterior to eye twice as wide as long. Eyes small, ratio of diameter/interocular width: 0.46. Ratio of length of metepisternum/metacoxa: 1/1.61. Metatibia moderately long and wide, ratio width/length: 1/3.58; basal group of dorsal spines of metatibia at first third of metatibial length.

Aedeagus: Fig. 23E–G. Habitus: Fig. 23H.

Female unknown.

#### Diagnosis.

*Tetrasericamicrospinosa* sp. n. differs from all *Tetraserica* species in having a very short median phallobasal lamina and by having both parameres long and filiform.

#### Etymology.

The name of the new species (adjective in nominative singular) is derived from the combined Greek and Latin words *micros* (small) and *spinosus* (with spines), with reference to a small spine-like tooth on the left paramere.

### Tetraserica microfurcata sp. n.

Taxon classificationAnimaliaColeopteraMelolonthidae

http://zoobank.org/E448A3AD-3288-4365-A4C0-1E951690C9E4

Figures 23 , 49

#### Type material examined.

Holotype: ♂ “Laos, 1-16.V.1999, Louangpharabang pr., 23°33–4'N, E102°14'E, Ban Song Cha (5km W), 1200 m, Vít Kubáň leg./ coll. P. Pacholátko” (CPPB). Paratypes: 1 ♂ “Vietnam: Cuc phong, Ninh binh, 3-10.V.1966 Exp. Gy. Topál / Nr. 268 beaten from bushes near creek” (ZFMK), 1 ♀ “Vietnam: Cuc phong, Ninh binh, 3-10.V.1966 Exp. Gy. Topál / Nr. 252 singled material” (HNHM), 1 ♂ “Vietnam: Cuc phong, Ninh binh, 6-18.V.1966 Topál/ Nr. 387 from trap in soil” (HNHM).

#### Description.

Length of body: 8.6 mm; length of elytra: 6.5 mm; maximum width: 5.3 mm. Surface of labroclypeus and disc of frons glabrous. Smooth area anterior to eye twice as wide as long. Eyes moderately large, ratio of diameter/interocular width: 0.57. Ratio of length of metepisternum/metacoxa: 1/1.38. Metatibia moderately long and wide, ratio width/length: 1/3.43; basal group of dorsal spines of metatibia at first third of metatibial length.

Aedeagus: Fig. 23I–K. Habitus: Fig. 23L.

Female unknown.

#### Variation.

Length of body: 7.6–8.6 mm; length of elytra: 5.5–6.5 mm; maximum width: 4.5–5.3 mm.

#### Diagnosis.

*Tetrasericamicrofurcata* sp. n. differs from the similar *T.microspinosa* sp. n. by the weakly curved, long and filiform left paramere, which is split in apical quarter into two fine branches, as well as the short and slim right paramere being strongly bent at middle.

#### Etymology.

The name of the new species (adjective in nominative singular) is derived from the combined Greek and Latin words *micros* (small) and *furcatus* (forked), with reference to the superficial split of the left paramere into two filiform branches.

### Tetraserica takahashii

Taxon classificationAnimaliaColeopteraMelolonthidae

Kobayashi, 2017

Figures 24 , 54

Tetraserica takahashii Kobayashi, 2017: 36, figs 3, 12.

#### Material examined.

1 ♂ “X-DA4503 labcode: VD025/ Vietnam, Quang Binh prov. 1 km N of Cha Lo, 400 m Vietnam-Laos border area 17°41'22"N, 105°45'45"E, 11.-24.iv.2010 L. Dembický leg. (VN1/ 2010 MZM EXPEDDITION) Tetraserica spVI_V3/ X-DA4503/ sp-VI-V2” (ZFMK), 1 ♂ “X-DA4502 labcode: VD024/ Vietnam, Quang Binh prov. 1km N of Cha Lo, 400 m Vietnam-Laos border area 17°41'22"N, 105°45'45"E, 11-24.iv.2010 L. Dembický leg. (VN1/ 2010 MZM EXPEDDITION) Tetraserica spVI_V3/ X-DA4502” (ZFMK), 3 ♂♂ “Vietnam, Quang Binh prov. 1 km N of Cha Lo, 400 m Vietnam-Laos border area 17°41'22"N, 105°45'45"E, L Dembický leg. 11-24.iv.2010” (ZFMK).

#### Description.

Length of body: 9 mm; length of elytra: 6.8 mm; maximum width: 6.3 mm. Surface of labroclypeus and disc of frons glabrous. Smooth area anterior to eye twice as wide as long. Eyes moderately large, ratio of diameter/interocular width: 0.58. Ratio of length of metepisternum/metacoxa: 1/1.55. Metatibia short and wide, ratio width/length: 1/3.17; basal group of dorsal spines of metatibia at first third of metatibial length.

**Figure 24.**
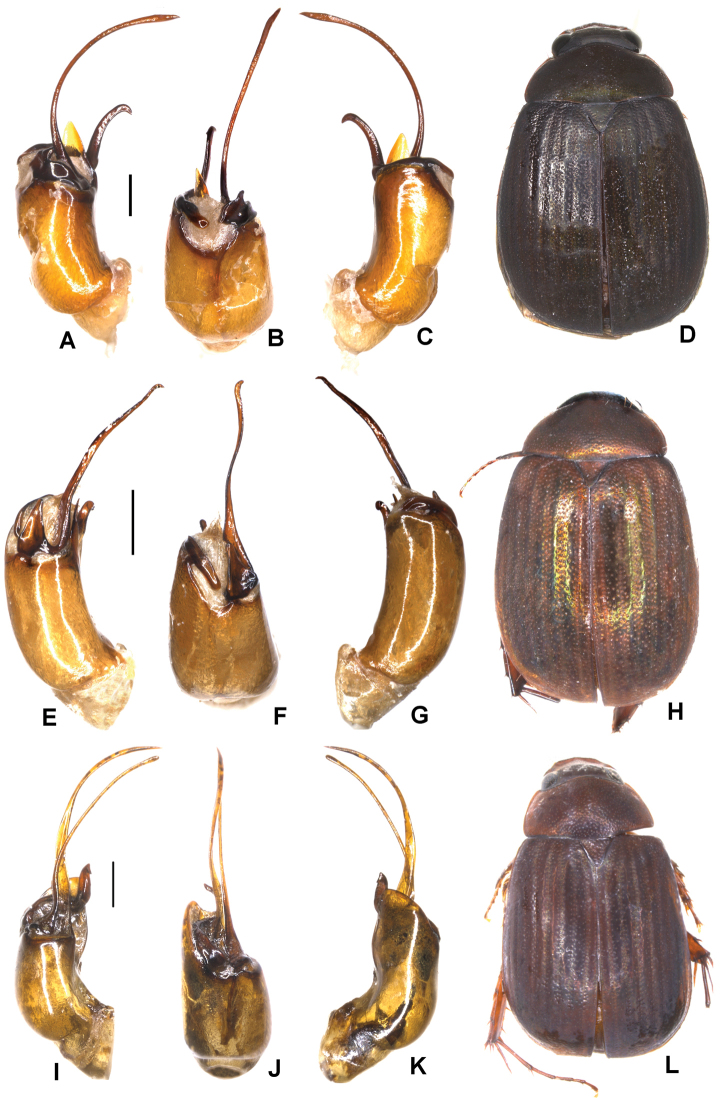
**A–D***Tetrasericatakahashii* Kobayashi, 2017 (Vietnam: Cha Lo) **E–H***T.ululalatensis* (holotype) **I–L***T.infida* sp. n. (holotype) **A, E, I** aedeagus, left side lateral view **C, G, K** aedeagus, right side lateral view **B, F, J** parameres, dorsal view **D, H, L** habitus. Scale bars: 0.5 mm. Habitus not to scale.

Aedeagus: Fig. 24A–C. Habitus: Fig. 24D.

### Tetraserica ululalatensis sp. n.

Taxon classificationAnimaliaColeopteraMelolonthidae

http://zoobank.org/4FFC6D8B-5555-47A8-8EF6-D30F8C0F9477

Figures 24 , 51

#### Type material examined.

Holotype: ♂ “Malaysia W, Kelantan 30 km NW of Gua Musang Ulu Lalat Mt. 800–1000 m Kampong Sungai om 27.v.-19.vi.2011 Petr Cechovsky lgt.” (ZFMK).

#### Description.

Length of body: 7.6 mm; length of elytra: 6.0 mm; maximum width: 4.7 mm. Surface of labroclypeus and disc of frons glabrous. Smooth area anterior to eye twice as wide as long. Eyes large, ratio of diameter/interocular width: 0.72. Ratio of length of metepisternum/metacoxa: 1/1.62. Metatibia moderately long and wide, ratio width/length: 1/3.1; basal group of dorsal spines of metatibia at first third of metatibial length.

Aedeagus: Fig. 24E–G. Habitus: Fig. 24H.

Female unknown.

#### Diagnosis.

*Tetrasericaululalatensis* sp. n. differs from similar *T.fikaceki* Liu et al., 2014 by the dorsal lobe of right paramere being directed basally, and the ventral lobe of left paramere being extremely long rather than short.

#### Etymology.

The new species is named after the type locality, Ulu Lalat Mt (adjective in the nominative singular).

### Tetraserica infida sp. n.

Taxon classificationAnimaliaColeopteraMelolonthidae

http://zoobank.org/1657B2A3-FC91-4577-BC86-314FF439CF24

Figures 24 , 47

#### Type material examined.

Holotype: ♂ “Birmania Ruby M/ Doherty” (NHMUK). Paratype: 1 ♀ “Birmania Ruby M^es^/ Doherty/ Fry Coll. 1900.100.” (NHMUK).

#### Description.

Length of body: 7.3 mm; length of elytra: 5.4 mm; maximum width: 4.6 mm. Surface of labroclypeus and disc of frons glabrous. Smooth area anterior to eye twice as wide as long. Eyes moderately large, ratio of diameter/interocular width: 0.56. Ratio of length of metepisternum/metacoxa: 1/1.52. Metatibia short and wide, ratio width/length: 1/2.85; basal group of dorsal spines of metatibia at first third of metatibial length.

Aedeagus: Fig. 24I–K. Habitus: Fig. 24L.

#### Variation.

Length of body: 7.3–8.1 mm; length of elytra: 5.4–6.6 mm; maximum width: 4.6–5.0 mm. Female: Antennal club with three antennomeres, as long as remaining antennomeres combined; eyes as large as in male.

#### Diagnosis.

*Tetrasericainfida* sp. n. strongly resembles *T.maerimensis* Kobayashi, 2018; the new species differs from the latter by the simple left paramere not being divided into dorsal and ventral lobes.

#### Etymology.

The name of the new species (adjective in nominative singular) is derived from the combined Latin prefix *in*- (non-) and adjective *fidus* (split), with reference to its unsplit left paramere.

### Tetraserica bansanpakiana sp. n.

Taxon classificationAnimaliaColeopteraMelolonthidae

http://zoobank.org/2BA8B100-260C-4481-A964-9298E108A3CA

Figures 25 , 47

#### Type material examined.

Holotype: ♂ “NW Thailand, 25.iv-7.v.1996, Chiang Mai prov., BAN SAN PAKIA Sv. Bílý leg., 1700 m/ coll. P. Pacholátko/ 131 Sericini Asia spec.” (CPPB). Paratypes: 1 ♂ “Thai-N, 1.-19.5.1998, Chiang Mai prov., BAN SAN PAKIA, Bednařik leg., 1400 m/ coll. P. Pacholátko” (ZFMK), 1 ♂ “Thai-N 1-15.v.1998, Chiang Mai prov., 19°19'N, 98°50'E, SAN PAKIA, 1400 m, Vít Kubáň leg./ coll. P. Pacholátko/ 125 Sericini Asia spec.” (CPPB), 2 ♂♂ “Thai, 17.-24.V.1991, Chiang Dao, 1000 m, 19°25'N, 98°52'E, Vit Kubaň leg./ Thailand “Thanon Thong Chai” D. Král & V. Kubáň” (ZFMK), 3 ♂♂, 3 ♀♀ “Thailand 17.-24.V.1991 Chiang Dao 1000m 98°52'E, 19°25'N, V. Kuban lg.” (ZFMK), 1 ♂, 2 ♀♀ “Thailand 10.-16.V.1991 Chiang Dao 600 m 19°24'N, 98°55'E, V. Kuban lg.” (ZFMK), 3 ♂♂, 5 ♀♀ “Thailand 9.-14.V.1991 Chiang Dao 350 m 19°22'N, 98°57'E, V. Kuban lg.” (ZFMK), 3 ♂♂, 1 ♀ “Thailand bor. Chiang Dao env. 21.5.-4.6.1995, lgt. Snizek M.” (ZFMK), 1 ♀ “Thailand bor. Chiang Mai, 56 km NW, 99°25', 19°05', 7-14.6.1995, lgt. Snizek M.” (ZFMK), 3 ♂♂ “Thai 17-24/5.91 Chiang Dao mts. 19.25'N, 98.52E, lgt. D. Kral, 1000 m” (NMPC), 1 ♂ “839467 Tetraserica spTHAI_DE09_1 Thailand L. Dembicky 9.-13.5.2009 Doi Chiang Dao env. Chiang Mai Prov. 19°24'45"N, 98°51'30"E, 1200 m” (ZFMK).

#### Description.

Length of body: 6.9 mm; length of elytra: 4.6 mm; maximum width: 4.1 mm. Surface of labroclypeus and disc of frons glabrous. Smooth area anterior to eye twice as wide as long. Eyes small, ratio of diameter/interocular width: 0.41. Ratio of length of metepisternum/metacoxa: 1/1.6. Metatibia short and wide, ratio width/length: 1/3.2; basal group of dorsal spines of metatibia at first third of metatibial length. Robust spine at middle of ventral margin extremely prolonged and s-shaped, exceeding distal margin of metatibia.

**Figure 25.**
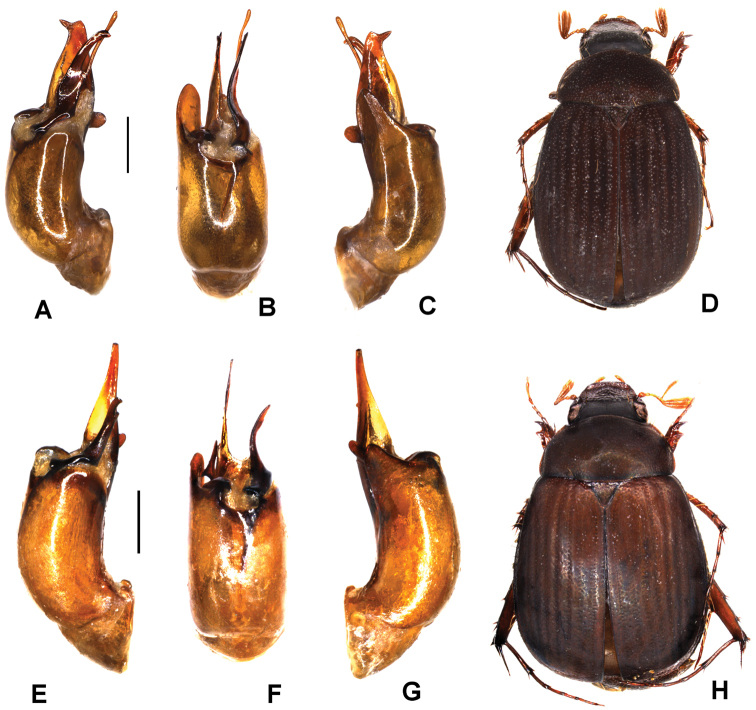
**A–D***Tetrasericabansanpakiana* sp. n. (holotype) **E–H***T.margheritae* sp. n. (holotype) **A, E** aedeagus, left side lateral view **C, G** aedeagus, right side lateral view **B, F** parameres, dorsal view **D, H** habitus. Scale bars: 0.5 mm. Habitus not to scale.

Aedeagus: Fig. 25A–C. Habitus: Fig. 25D.

#### Variation.

Length of body: 6.9–8.5 mm; length of elytra: 4.6–5.9 mm; maximum width: 4.1–5.1 mm. Colour may vary from dark brown to nearly entirely yellowish brown with dark head. Female: Antennal club with three antennomeres, as long as remaining antennomeres combined; eyes as large as in male; pygidium flat; robust spines on ventral margin of metatibia subequal in length.

#### Diagnosis.

The new species differs from all other *Tetraserica* species by the robust metatibial spine in middle of ventral margin being extremely elongate and s-shaped, exceeding distal margin of metatibia.

#### Etymology.

The new species is named after the type locality, Ban San Pakia (adjective in the nominative singular).

### Tetraserica margheritae sp. n.

Taxon classificationAnimaliaColeopteraMelolonthidae

http://zoobank.org/7DE10DB1-020C-48F0-80C1-0C0B9757430A

Figures 25 , 51

#### Type material examined.

Holotype: ♂ “Thailand bor. Chiang Mai, 56 km NW 99°25'E, 19°05'N, 7-14.6.1995 lgt. Snizek M./ 943 Sercini Asia spec.” (ZFMK). Paratype: 1 ♂ “Thailand bor. Chiang Mai, 56km NW 99°25'E, 19°05'N, 7-14.6.1995 lgt. Snizek M./ 943 Sercini Asia spec.” (ZFMK).

#### Description.

Length of body: 7.3 mm; length of elytra: 6.1 mm; maximum width: 4.6 mm. Surface of labroclypeus and disc of frons glabrous. Smooth area anterior to eye twice as wide as long. Eyes small, ratio of diameter/interocular width: 0.44. Ratio of length of metepisternum/metacoxa: 1/1.62. Metatibia short and wide, ratio width/length: 1/2.83; basal group of dorsal spines of metatibia at first third of metatibial length. Robust spine at middle of ventral margin extremely prolonged and s-shaped, exceeding distal margin of metatibia.

Aedeagus: Fig. 25E–G. Habitus: Fig. 25H.

Female unknown.

#### Variation.

Length of body: 7.1–7.3 mm; length of elytra: 4.8–6.1 mm; maximum width: 4.5–4.6 mm.

#### Diagnosis.

*Tetrasericamargheritae* sp. n. differs from the externally similar *T.bansanpakiana* sp. n. by the median phallobasal lamina being narrower and sharply pointed at apex, also by having a dorsal tooth and the left paramere being distinctly shorter than median phallobasal lamina.

#### Etymology.

The new species is named after Silvia’s mother, Margherita (noun in genitive singular).

### Tetraserica maerimensis

Taxon classificationAnimaliaColeopteraMelolonthidae

Kobayashi, 2018

Figures 26 , 51

Tetraserica maerimensis Kobayashi, 2018: 57.

#### Material examined.

1 ♂ “N Thailand 12-14.V.1990 Doi Inthanon lg Malicky / Zoologische Staatssammlung München/ 113 Sericini Asia spec.” (ZSM), 16 ♂♂, 5 ♀♀ “Thailand: 29.5.-5.6.1989 Dai [Doi] Inthanon, Lichtfalle, Bang Khun Klang 1200 m 98°32'E, 18°32'N, Chantaramongkol & Malicky leg.” (ZSM), 16 ♂♂, 11 ♀♀ “N-Thailand 25.-29.5.1990 Doi Inthanon leg. Malicky” (ZSM), 25 ♂♂, 6 ♀♀ “N-Thailand V.1990 Doi Inthanon lg. Malicky” (ZSM), 1 ♂ “N-Thailand 1.-17.VII.1990 Doi Inthanon leg. Malicky” (ZSM), 34 ♂♂, 6 ♀♀ “N-Thailand 12.-14.V.1990 Doi Inthanon leg. Malicky” (ZSM), 1 ♂ “NW Thailand, 19.19N, 97.59E Mae Hong Song, 1991 Ban Huai Po, 1600–2000 m 17.-23.5., L. Dembicky leg.” (NHMW), 1 ♂ “X-DA2309/ X-DA2309 labcode: VD003 Thailand N.: Doi Inthanon Nat. Res. 1250 m 22-31.v.2008 S. Murzin Tetraserica spThaix2/ sp-ThaiX2” (ZFMK), 1 ♀ “X-DA2322/ X-DA2322 labcode: VD004 Thailand N.: Doi Inthanon Nat. Res. 1250 m 22-31.v.2008 S. Murzin Tetraserica spThaix2” (ZFMK), 1 ♂ “THAI 28-31/5 1995 19.27N 98.20E Soppong 1500 m Vít Kubáň leg./ coll. P. Pacholátko/ TS29/ 140 Sericini Asia spec.” (CPPB), 4 ♂♂ “THAILAND, CHIANG MAI Chiang Mai, Doi Sutheppui, Natn. Park (1300 m) 1-2.6.1984 Matti Hämäiäinen leg./ MUSEUM LEIDEN Ex. Coll. M. Hämäiäinen acq. 2002” (RMNH), 2 ♂♂ “Thailand 7.-12.5.1996 Mae Hong Son prov. Soppong 1500 m 19°27'N; 98°20'E, lgt. S. Becvar” (ZFMK), 2 ♂♂, 2 ♀♀ “N. Thailand Mae Hong Son Pref. Soppong Pai Dist. Alt. 1290 m 20-21.V.1998 K. Masumoto leg.” (ZFMK), 1 ♂ “N-Thailand Ban Mai [Huai] Po 9.-16.5.1991 L. Horak lgt./ Coll. Milan Nikodym Praha” (ZFMK), 1 ♂ “836053/ 836053 Thailand N. Doi Inthanon Nat. Res. 1250 m 22-31.v.2008 leg. S. Murzin Tetraserica spTHAIx2” (ZFMK).

**Figure 26.**
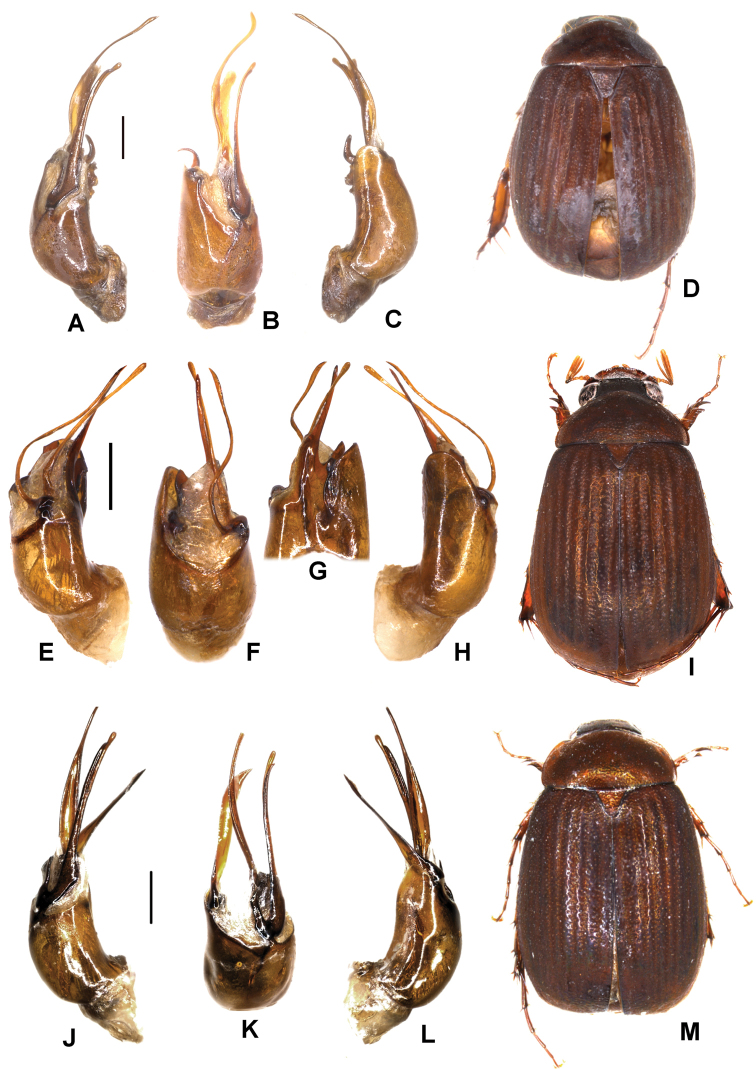
**A–D***Tetrasericamaerimensis* Kobayashi, 2018 (Thailand, Doi Inthanon) **E–I***T.soppongana* sp. n. (holotype) **J–M***T.longipenis* Liu et al., 2014 (holotype) **A, E, J** aedeagus, left side lateral view **C, H, L** aedeagus, right side lateral view **B, F, K** parameres, dorsal view **G** parameres, ventral view **D, I, M** habitus. Scale bars: 0.5 mm. Habitus not to scale.

Aedeagus: Fig. 26A–C. Habitus: Fig. 26D.

### Tetraserica soppongana sp. n.

Taxon classificationAnimaliaColeopteraMelolonthidae

http://zoobank.org/17358D8D-981B-4CB4-863B-E15CF4F97588

Figures 26 , 53

#### Type material examined.

Holotype: ♂ “THAI 1-8.V.1993 SOPPONG PAI 1800 m Pacholátko & Dembický leg./ Coll. P. Pacholátko/ 160 Sericini Asia spec.” (CPPB). Paratype: 1 ♂ “THAI 28-31/5.1995 19.27N 98.20E SOPPONG 1500m Vít Kubáň leg./ Coll. P. Pacholátko/ TS49/ 472 Sericini Asia spec.” (ZFMK).

#### Description.

Length of body: 6.9 mm; length of elytra: 5 mm; maximum width: 4 mm. Body yellowish brown, head dark, antenna yellow. Surface of labroclypeus and disc of frons glabrous. Smooth area anterior to eye twice as wide as long. Eyes moderately large, ratio of diameter/interocular width: 0.71. Ratio of length of metepisternum/metacoxa: 1/1.55. Metatibia moderately long and wide, ratio width/length: 1/3.4; basal group of dorsal spines of metatibia at first third of metatibial length.

Aedeagus: Fig. 26E–H. Habitus: Fig. 26I.

Female unknown.

#### Variation.

No significant size variation between types.

#### Diagnosis.

*Tetrasericasoppongana* sp. n. differs from all other *Tetraserica* species in having a long median phallobasal lamina, the right paramere composed of two lobes and having no brush of spines at the base of the right paramere, by the left paramere being composed of two lobes, the right side of phallobase strongly produced, and the dorsal lobe of left paramere as long as ventral one.

#### Etymology.

The new species is named after the type locality, Soppong (adjective in the nominative singular).

### Tetraserica longipenis

Taxon classificationAnimaliaColeopteraMelolonthidae

Liu, Fabrizi, Bai, Yang & Ahrens, 2014

Figures 26 , 49

Tetraserica longipenis Liu, Fabrizi, Bai, Yang & Ahrens, 2014: 98, fig. 4I–L.

#### Material examined.

**Vietnam**: 1 ♂ “N-VIETNAM Cao Bang Prov., vic. Vin Den, Nui Pia Oac Nat. Res.06.-10.V.2013, 22°33‘53“N, 105°52‘53"E, 900–1300 m A. Skale“ (ZFMK). **Thailand**: 1 ♂ “Mt. Doi Ku Sath-an, Na Noi Nan. N. Thailand 16/V/93 S. Ohmomo leg./ coll. D. Ahrens” (ZFMK). **Laos**: 1 ♂ “LAOS north, 24-30.V.1997, 20 km NWLouang Namtha, N 21°09.2, E 101°18.7, alt. 900±100 m, E. Jendek & O. Šauša leg./ (Coll. P. Pacholátko)” (CPPB), 1 ♂ “LAOS, N 21°09.2, 101°19 E, Louangnamtha pr., Namtha-Muang Sing, 5-31.v.1997, 900–1200 m Vít Kubáň leg./ Coll. P. Pacholátko” (CPPB), 1 ♂ “ X-DA4512 labcode: VD032 LAOS Hua Phan prov.; Ban Saleui, Phou Pan (Mt) 20°12'N, 104°01'E, 3-5.iv.2013 leg., C. Holzschuh Tetraserica spLA_V5/ X-DA4512” (ZFMK).

Aedeagus: Fig. 26J–L. Habitus: Fig. 26M.

#### Remarks.

This species was known from southern China and Thailand (Kobayashi 2017), and it is recorded here from Vietnam and Laos for the first time.

### Tetraserica angkhangensis

Taxon classificationAnimaliaColeopteraMelolonthidae

Kobayashi, 2017

Figures 27 , 47

Tetraserica angkhangensis Kobayashi, 2017: 42, figs 9, 18.

#### Material examined.

**Myanmar**: 1 ♂ “Burma (Myanmar) SW: Shan state Taunggyi J. Rejsek 1-18.6.1997/ coll. D Ahrens/ Sericini Asia spec. 214” (ZFMK), 8 ♂♂ “Burma (Myanmar) SW: Shan state Taunggyi J. Rejsek 1-18.6.1997/ coll. D Ahrens” (ZFMK), 4 ♂♂, 2 ♀♀ “Burma (Myanmar) SW: Shan state Taunggyi J. Reysek [sic!] 1-18.6.1997/ coll. D Ahrens” (ZFMK).

**Figure 27.**
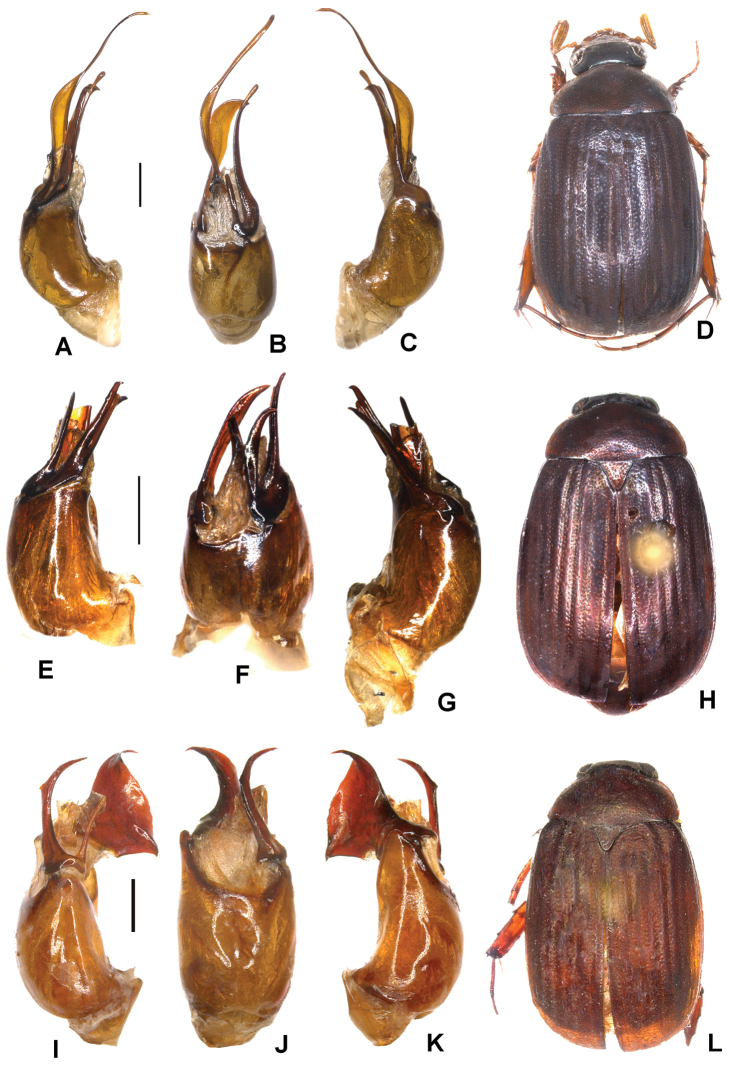
**A–D***Tetrasericaangkhangensis* Kobayashi, 2017 (Myanmar, Taunggyi) **E–H***T.giulianae* sp. n. (holotype) **I–L***T.yaoquensis* Liu et al., 2014 (holotype) **A, E, I** aedeagus, left side lateral view **C, G, K** aedeagus, right side lateral view **B, F, J** parameres, dorsal view **D, H, L** habitus. Scale bars: 0.5 mm. Habitus not to scale.

Aedeagus: Fig. 27A–C. Habitus: Fig. 27D.

#### Remarks.

This species was described from Thailand, and is recorded from Myanmar for the first time.

### Tetraserica giulianae sp. n.

Taxon classificationAnimaliaColeopteraMelolonthidae

http://zoobank.org/A8E0B987-9B05-4DA9-9AC8-9FF05EAE1E91

Figures 27 , 56

#### Type material examined.

Holotype: ♂ “Shan States Mandets/ 1001 Asia Sericini spec.” (MNHN).

#### Description.

Length of body: 7.8 mm; length of elytra: 5.9 mm; maximum width: 4.8 mm. Surface of labroclypeus and disc of frons glabrous. Smooth area anterior to eye twice as wide as long. Eyes moderately large, ratio of diameter/interocular width: 0.56. Ratio of length of metepisternum/metacoxa: 1/1.64. Metatibia short and wide, ratio width/length: 1/3.08; basal group of dorsal spines of metatibia at first third of metatibial length. Ventral distal spine of metatibia inserted shortly behind middle of ventral margin of metatibia and longer than the long metatarsomere 1.

Aedeagus: Fig. 27E–G. Habitus: Fig. 27H.

Female unknown.

#### Diagnosis.

*Tetrasericagiulianae* sp. n. is similar to *T.angkhangensis* Kobayashi, 2017 in shape of the aedeagus, but the new species differs by the basal lobe of left paramere being as long as the ventral lobe, rather than half its length.

#### Etymology.

The new species is named in honour of our friend Giuliana Caturegli (Rome) (noun in genitive singular).

### Tetraserica yaoquensis

Taxon classificationAnimaliaColeopteraMelolonthidae

Liu, Fabrizi, Bai, Yang & Ahrens, 2014

Figures 27 , 55

Tetraserica yaoquensis Liu, Fabrizi, Bai, Yang & Ahrens, 2014: 98, fig. 4E–H.

#### Material examined.

**Laos**: 1 ♂ “Laos, 21°09'N, 101°19'E, Louangnamtha pr. Namtha-Muang Sing, 5-31.v.1997, 900–1200 m Vit. Kubáň leg/ coll. P. Pacholátko/ 198 Sericini Asia spec.” (CPPB), 1 ♂ “X-DA4627 labcode: VD066 LAOS, Stupa GH, 5 km W Muang Sing, 750 m, 21.1482N 101.1711E, 9.v-2.vi.2011, M. Murzin, O. Shulga leg. Tetraserica spLA_V35” (ZFMK).

Aedeagus: Fig. 27I–K. Habitus: Fig. 27L.

#### Remarks.

This species was described from Yunnan, China, and it is recorded from Laos for the first time.

### Tetraserica gressitti

Taxon classificationAnimaliaColeopteraMelolonthidae

(Frey, 1972) comb. n.

Figures 28 , 48

Tetraserica gressitti Frey, 1972: 198, fig. 60.

#### Type material examined.

Paratypes: 1 ♂, 1 ♀ “Viet Nam DiLinh (Dijiring) 1200 m, 22-28.IV.60/ at light/ L.W. Quate/ Paratype Neosericagressitti G. Frey 1971” (CF), 1 ♂, 1 ♀ “Viet Nam DiLinh (Dijiring) 1200 m, 27.IX-14.X.1960/ Light trap/ L.W. Quate Collector/ Paratype Neosericagressitti G. Frey 1971” (CF).

#### Additional material examined.

1 ♂ “Vietnam Djiring, 900m 24.IV.1960” (BPBM), 1 ♂ “S Vietnam: Lam Dong Prov., Cát-Tien Distr., Cát-Tien National Park, Headquarter area (120 m. a.s.l) 11-15.VI.2015 at light/ legit L. Bartolozzi, G. Chelazzi, S. Bambi, F. Fabiano, E. Orbach, V. Sbordoni (numero magazzino 3023)/ 235 Sericini Asia spec.” (MZUF), 1 ♂ “S VIETNAM 1-15.5.1994 Nam Cát Tian –Nat. park, P. Pacholátko & L. Dembický leg./ coll. P. Pacholátko” (CPPB), 2 ♂♂ “Museum Leiden Viet Nam (Dong Nai Prov.) Cát-Tian N. P.: near guesthouse. 13-v-2007. leg. E. Gassó Miracle & Nguyen Than Mahn/ secondary humid lowland forest; light trap (ML), 18-20 hrs11°26'20.6"N, 107°25'42"E” (RMNH), 1 ♂ “Museum Leiden Viet Nam (Dong Nai Prov.) Cát-Tien N.P.: Dong trail; near guesthouse 16.v.2007. leg. E. Gassó Miracle & Nguyen Than Mahn/ humid lowland forest; light trap (ML), 19-21:30 hrs; 11°26'20.6"N, 107°25'42"E” (RMNH), 2 ♂♂ “Museum Leiden Viet Nam (Dong Nai Prov.) Cát-Tien N.P.: Botanical Garden. 13-20.v.2007. leg. C. van Achterberg, R. de Vries & E. Gassó Miracle/ mixed bamboo and wood forest; in malaise traps; 250 m, 11°26'20.6"N, 107°25'42"E” (RMNH), 1 ♂ “S. Vietnam (Cat Tien) 120 km NNE Ho Chi Minh, Cat Tien Nat. Park 27.6.-10.7.1995 leg. A. Napolov” (CNAR).

#### Redescription.

Length of body: 9.3 mm; length of elytra: 6.8 mm; maximum width: 6 mm. Surface of labroclypeus and disc of frons glabrous. Smooth area anterior to eye twice as wide as long. Eyes moderately large, ratio of diameter/interocular width: 0.61. Ratio of length of metepisternum/metacoxa: 1/1.79. Metatibia short and wide, ratio width/length: 1/2.88; basal group of dorsal spines of metatibia at first third of metatibial length.

**Figure 28.**
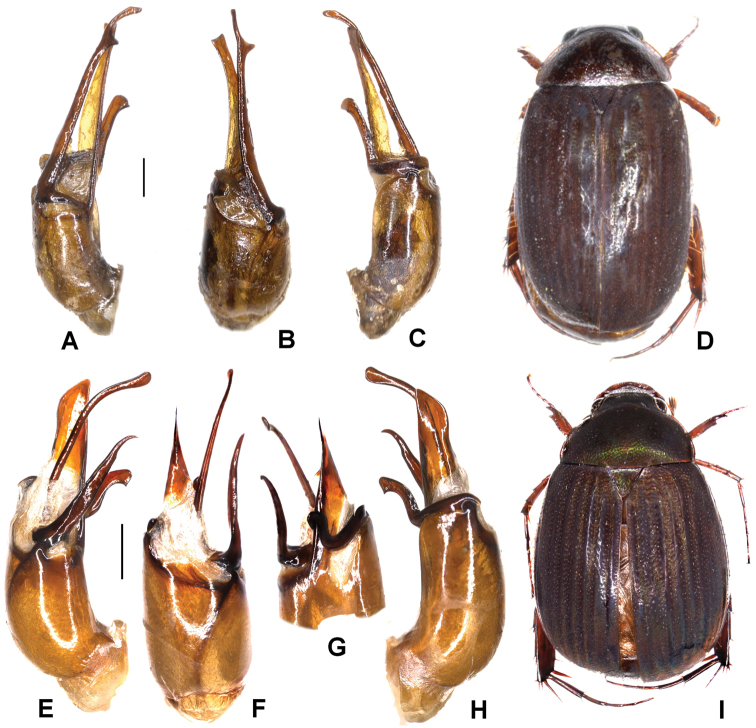
**A–D***Tetrasericagressitti* (Frey, 1972) (paratype) **E–I***T.loeiensis* sp. n. (holotype) **A, E** aedeagus, left side lateral view **C, H** aedeagus, right side lateral view **B, F** parameres, dorsal view **G** parameres, dorsal view **D, I** habitus. Scale bars: 0.5 mm. Habitus not to scale.

Aedeagus: Fig. 28A–C. Habitus: Fig. 28D.

Female unknown.

### Tetraserica loeiensis sp. n.

Taxon classificationAnimaliaColeopteraMelolonthidae

http://zoobank.org/757B29AB-A280-41E1-B6F1-6341FB332A97

Figures 28 , 48

#### Type material examined.

Holotype: ♂ “Coll. I. R. Sc. N. B., THAILAND (loei), Na-Haeo (field res stat) 15-19.V.2003 Light trap Leg. J. Constant, K. Smets & P. Grootaert/ 66V sp.” (ISNB). Paratypes: 1 ♂ “Coll. I. R. Sc. N. B., THAILAND (loei), Na-Haeo (field res stat) 15-19.V.2003 Light trap Leg. J. Constant, K. Smets & P. Grootaert” (ZFMK), 1 ♂ “X-DA4647 labcode: VD075, THAILAND: Loei Ruea NP behind checkpoint 691 m Malaise trap, 17°27.829'N, 101°21.360'E, 5-8.vi.2007, Patikhom Tumtop leg. T2514, Tetraserica spTH_V66/ X-DA4647” (QSBG).

#### Description.

Length of body: 7.5 mm; length of elytra: 5.6 mm; maximum width: 5 mm. Surface of labroclypeus and disc of frons glabrous. Smooth area anterior to eye twice as wide as long. Eyes moderately large, ratio of diameter/interocular width: 0.56. Ratio of length of metepisternum/metacoxa: 1/1.8. Metatibia moderately long and wide, ratio width/length: 1/3.91; basal group of dorsal spines of metatibia at first third of metatibial length.

Aedeagus: Fig. 28E–H. Habitus: Fig. 28I.

Female unknown.

#### Variation.

Length of body: 7.5–7.7 mm; length of elytra: 5.5–5.6 mm; maximum width: 4.7–5.0 mm.

#### Diagnosis.

*Tetrasericaloeiensis* sp. n. is rather similar to *T.matsumotoi* Kobayashi, 2017 and *T.wapiensis* (Frey, 1972) in shape of the aedeagus; the new species differs from both by the dorsal lobe of left paramere which is distinctly longer than the ventral one.

#### Etymology.

The new species is named with reference to its occurrence in Loei province (adjective in the nominative singular).

### Tetraserica subrotundata sp. n.

Taxon classificationAnimaliaColeopteraMelolonthidae

http://zoobank.org/E13B4C20-331D-4A7E-8652-9A8FA6B817E7

Figures 29 , 53

#### Type material examined.

Holotype: ♂ “Coll. I. R. Sc. N. B. Cambodia (Pursat Prov.) Phnom Samkos wild life Sanctuary, light trap Forest Edge & Primary 14-IV-2005 Leg. K Smets & I Var/ 903 Sericini Asia spec. “ (ISNB). Paratypes: 1 ♂ “Coll. I. R. Sc. N. B. Cambodia, Pursat Prov. Phnom Samkos W.S. Pramaoy, forest edge, 13-IV-2005, Light Trap Leg. K Smets & I Var/ Sericini Asia spec. 905” (ZFMK), 1 ♂ “Coll. I. R. Sc. N. B. Cambodia, Pursat Prov. Phnom Samkos W.S. Pramaoy, forest edge, 13-IV-2005, Light Trap Leg. K Smets & I Var” (ISNB).

#### Description.

Length of body: 7.1 mm; length of elytra: 5.5 mm; maximum width: 4.8 mm. Surface of labroclypeus and disc of frons glabrous. Smooth area anterior to eye twice as wide as long. Eyes moderately large, ratio of diameter/interocular width: 0.57. Ratio of length of metepisternum/metacoxa: 1/1.83. Metatibia short and wide, ratio width/length: 1/3.15; basal group of dorsal spines of metatibia at first third of metatibial length.

**Figure 29.**
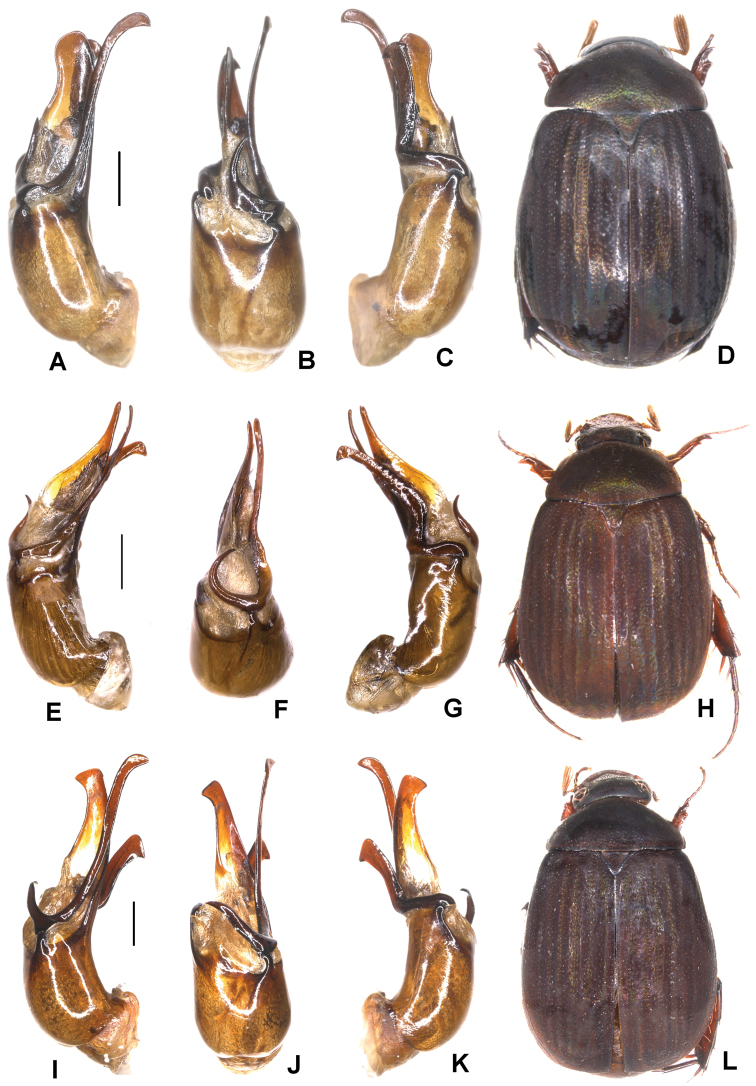
**A–D***Tetrasericasubrotundata* sp. n. (holotype) **E–H***T.matsumotoi* Kobayashi, 2017 (Thailand: Chom Thong) **I–L***T.kiriromensis* sp. n. (holotype) **A, E, I** aedeagus, left side lateral view **C, G, K** aedeagus, right side lateral view **B, F, J** parameres, dorsal view **D, H, L** habitus. Scale bars: 0.5 mm. Habitus not to scale.

Aedeagus: Fig. 29A–C. Habitus: Fig. 29D.

Female unknown.

#### Variation.

Length of body: 7.1–8.1 mm; length of elytra: 5.5–5.8 mm; maximum width: 4.7–4.8 mm.

#### Diagnosis.

*Tetrasericasubrotundata* sp. n. is rather similar to *T.matsumotoi* Kobayashi, 2017 in shape of the aedeagus; the new species differs by the right paramere being bent only once and being slightly wider in lateral view, and the apex of median phallobasal lamina strongly rounded instead of sharply pointed.

#### Etymology.

The name of the new species (adjective in nominative singular) is derived from the combined Latin prefix *sub*- (under) and adjective *rotundatus* (rounded), with reference to the shape of the medial apical phallobasal lamina of the aedeagus.

### Tetraserica matsumotoi

Taxon classificationAnimaliaColeopteraMelolonthidae

Kobayashi, 2017

Figures 29 , 51

Tetraserica matsumotoi Kobayashi, 2017: 34, figs 1, 10.

#### Material examined.

1 ♂ “Thailand occ. bor. 24-28.04.1991 Chom Thong Jan Farkač leg./ NHM Basel/ 104 Sericini Asia spec.” (NHMB), 1 ♂ “Thailand occ. bor. 24-28.04.1991 Chom Thong Jan Farkač leg.” (NHMB), 3 ♂♂ “NE Thailand. 23-27.4.1991 Chom Thong S. Bily leg.” (NHMB, ZFMK), 1 ♂ “a. 200, Klong Wang Chao, Kamphaeng P Thai., 24-27.V.2005 Takakuwa, M. leg.” (ZFMK), 1 ♂ “N- THAILAND: Angkhai village, Samoeng Dist. Chiang Mai Prov., 9-11.v.1999 K. Masumoto leg./ coll. Dirk Ahrens” (ZFMK), 1 ♂ “N. Thailand: Chiang Mai Pref., Ban Angkhai, Samoeng Dist., 750 m, 15.-20.V.1998 K. Masumoto leg.” (ZFMK), 1 ♂ “X-DA4660/ X-DA4660 labcode: VD079 THAILAND Chiang Dao Hill Resort 600 m, 19.55779N 99.0766E, 28.iv.-5.v.2011, M. Murzin, O. Shulga leg. Tetraserica spTH_V43” (ZFMK), 1 ♂ “X-DA4720/ X-DA4720 labcode: VD094 THAILAND Chiang Dao Hill Resort 600 m, 19.55779N 99.0766E, 28.iv.-5.v.2011, M. Murzin, O. Shulga leg. Tetraserica spTH_V54” (ZFMK), 1 ♂ “X-DA4722/ X-DA4722 labcode: VD095 THAILAND Chiang Dao Hill Resort 600 m, 19.55779N 99.0766E, 28.iv.-5.v.2011, M. Murzin, O. Shulga leg. Tetraserica spTH_V43” (ZFMK), 1 ♂ “X-DA4727/ X-DA4727 labcode: VD096 THAILAND Chiang Dao Hill Resort 600 m, 19.55779N 99.0766E, 28.iv.-5.v.2011, M. Murzin, O. Shulga leg. Tetraserica spTH_V43” (ZFMK), 1 ♂ “X-DA4869 labcode VD106 Thailand, Chiang Dao Hill Resort (100 km N of Chiang Mai) 600 m, 28.v.-8.vi.2009, S. Murzin leg. Tetraserica spTH_V43/ X-DA4869” (ZFMK), 1 ♂ “X-DA4956 labcode VD108 Thailand, Chiang Dao Hill Resort (100 km N of Chiang Mai) 600 m, 28.v.-8.vi.2009, S. Murzin leg. Tetraserica spTH_V43/ X-DA4956” (ZFMK), 1 ♂ “THAI, 9.-13.IV.1991 THIMONGHTA 350 m 15 02'N, 98 35'E, P. Pacholátko leg./ coll. P. Pacholátko” (CPPB), 1 ♂ “N- THAILAND III.1992 3 km W Ban Rai 170 km NW Bankok 150 m NN lg. Malicky/ Zoologische Staatssammlung München” (ZSM), 13 ♂♂ “Thailand 9.-14.V.1991 Chiang Dao 350 m 19°22'N, 98°57'E, V. Kuban lg./ coll. Milan Nikodym, Praha “ (ZFMK), 2 ♂♂ “Thailand 26.-28.V.1991 Palong 750 m 19°55'N, 99°06'E, Vit Kuban lgt./ coll. Milan Nikodym, Praha” (ZFMK), 5 ♂♂ “N-Thailand 25.-29.5.1990 Doi Inthanon leg. Malicky” (ZSM), 1 ♂ “a. 200 m, Klong Wang Chao Kamphaeng P Thai., 24-27.V.2005 Takakuwa, M. leg.” (ZFMK), 1 ♂ “W-Thailand: Pu Nam Long, Hot Spring, 100 km NW of Kanchaburi; 06-8.V.1993 leg. T. Itoh “ (ZFMK), 1 ♂ “Thai 9-14.5.1991 Chiang Dao 350 m 19°22'N, 98°57'E, D. Kral lgt.” (NMPC), 1 ♂ “Thai 26/4- 4-6/5.91 Umphang 500 m 16°04'N, 98°53'E, David Kral lgt.” (NMPC), 1 ♂ “835112/ 835112 – Thailand 100 km N Chiang Mai, Chiang Dao hill Resort 20-30.vi.2008 leg. S. Murzin – Tetraserica Thaisp5” (ZFMK), 1 ♂ “NW Thailand, 19.19N, 97.59E, Mae Hong Son, 1991, Ban Huai Po, 1600–2000 m 17.-23.5. L. Dembický leg.” (NHMB), 1 ♂ “Haut Mekong Muong Sing 18.IV.1918 R.V. de Salvaza.” (NHMUK), 3 ♂♂ “THA, Phitsanulok, 45 km E Phit-sanulok, Thung 16°51'18"N/ 100°40'19"E, 155 m, 06.05.2012 1/2012 leg. E. u. J. Hüttinger” (NME).

Aedeagus: Fig. 29E–G. Habitus: Fig. 29H.

### Tetraserica kiriromensis sp. n.

Taxon classificationAnimaliaColeopteraMelolonthidae

http://zoobank.org/9558FB03-AA41-465E-A16D-A5893FD593E1

Figures 29 , 48

#### Type material examined.

Holotype: ♂ “Coll. I. R. Sc. N. B., Cambodia, Kirirom N.P. 21.iv.2005 Pine forest, Light Trap, Leg. K Smets & I Var” (ISNB). Paratypes: 2 ♂♂ “Coll. I. R. Sc. N. B., Cambodia, Kirirom NP, 21.iv.2005, Pine forest, Light Trap, Leg. K Smets & I Var” (ISNB, ZFMK).

#### Description.

Length of body: 8.4 mm; length of elytra: 5.8 mm; maximum width: 5.4 mm. Surface of labroclypeus and disc of frons glabrous. Smooth area anterior to eye twice as wide as long. Eyes small, ratio of diameter/interocular width: 0.5. Ratio of length of metepisternum/metacoxa: 1/1.69. Metatibia short and wide, ratio width/length: 1/3.29; basal group of dorsal spines of metatibia at first third of metatibial length.

Aedeagus: Fig. 29I–K. Habitus: Fig. 29L.

Female unknown.

#### Variation.

Length of body: 7.5–8.6 mm; length of elytra: 5.8–6.4 mm; maximum width: 5.0–5.9 mm.

#### Diagnosis.

*Tetrasericakiriromensis* sp. n. is rather similar to *T.matsumotoi* Kobayashi, 2017 in shape of aedeagus; the new species differs by the right paramere being distinctly shorter than median lamina of phallobase; also the apex of median phallobasal lamina is blunt and curved abruptly dorsally (rather than being straight and sharply pointed).

#### Etymology.

The new species is named after the type locality, Kirirom National Park (adjective in the nominative singular).

### Tetraserica angkorthomensis sp. n.

Taxon classificationAnimaliaColeopteraMelolonthidae

http://zoobank.org/5EED768B-5DB7-4773-84E6-252D3FD88FCB

Figures 30 , 46

#### Type material examined.

Holotype: ♂ “Coll. I. R. Sc. N. B. CAMBODIA (Siem Riep Prov) Angkor Thom 26-V-2003 Light Trap Leg. Constant & K. Smets/ 938 Sericini Asia spec.” (ISNB).

#### Description.

Length of body: 7.9 mm; length of elytra: 5.6 mm; maximum width: 5.6 mm. Surface of labroclypeus and disc of frons glabrous. Smooth area anterior to eye twice as wide as long. Eyes small, ratio of diameter/interocular width: 0.41. Ratio of length of metepisternum/metacoxa: 1/1.79. Metatibia short and wide, ratio width/length: 1/3.08; basal group of dorsal spines of metatibia at first third of metatibial length.

**Figure 30.**
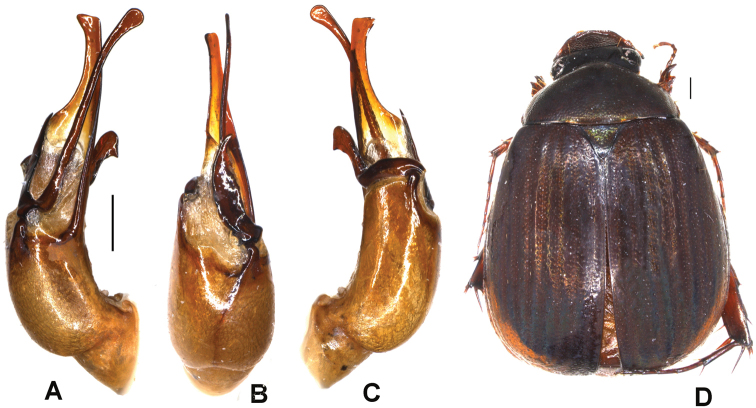
**A–D***Tetrasericaangkorthomensis* sp. n. (holotype) **A** aedeagus, left side lateral view **C** aedeagus, right side lateral view **B** parameres, dorsal view **D** habitus. Scale bars: 0.5 mm. Habitus not to scale.

Aedeagus: Fig. 30A–C. Habitus: Fig. 30D.

Female unknown.

#### Diagnosis.

*Tetrasericaangkorthomensis* sp. n. differs from the very similar *T.kiriromensis* sp. n. by the distinctly shorter right paramere and wider and longer dorsal lobe of left paramere which is directed more distally rather mesally.

#### Etymology.

The new species is named after the type locality, Angkor Thom (adjective in the nominative singular).

### Tetraserica pailinensis sp. n.

Taxon classificationAnimaliaColeopteraMelolonthidae

http://zoobank.org/D7456CED-5A84-426D-ABF8-0F2FE6E4879B

Figures 31 , 50

#### Type material examined.

Holotype: ♂ “X-DA4506 labcode: VD028 Cambodia Pailin 200 m, 11-16.v.2009, S. Murzin leg. Tetraserica spCA_V4/ X-DA4506/ sp-CA-V4” (ZFMK). Paratype: 1 ♂ “835104/ 835104 Cambodia Pailin 270 m 6-16.v.2008 S. Murzin leg. Tetraserica sp CA2sp1” (ZFMK).

#### Description.

Length of body: 8.5 mm; length of elytra: 6.5 mm; maximum width: 5 mm. Surface of labroclypeus and disc of frons glabrous. Smooth area anterior to eye twice as wide as long. Eyes small, ratio of diameter/interocular width: 0.5. Ratio of length of metepisternum/metacoxa: 1/1.88. Metatibia moderately long and wide, ratio width/length: 1/3.46; basal group of dorsal spines of metatibia at first third of metatibial length.

**Figure 31.**
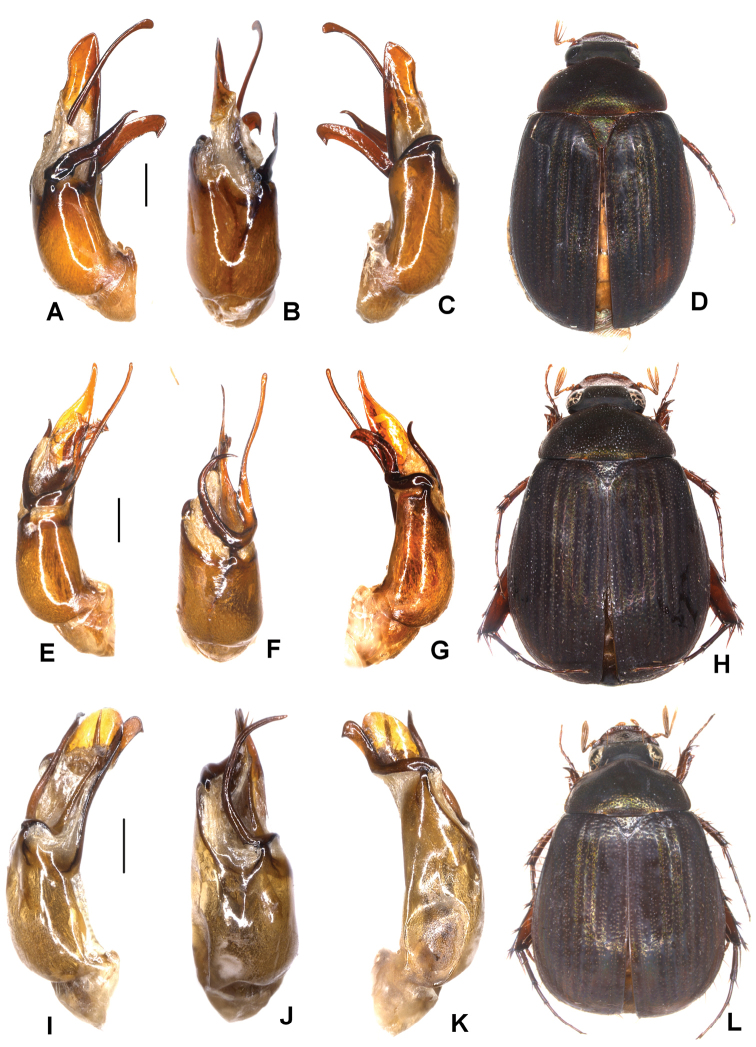
**A–D***Tetrasericapailinensis* sp. n. (holotype) **E–H***T.banhuaipoensis* sp. n. (holotype) **I–L***T.nussi* sp. n. (holotype) **A, E, I** aedeagus, left side lateral view **C, G, K** aedeagus, right side lateral view **B, F, J** parameres, dorsal view **D, H, L** habitus. Scale bars: 0.5 mm. Habitus not to scale.

Aedeagus: Fig. 31A–C. Habitus: Fig. 31D.

Female unknown.

#### Variation.

Length of body: 8.0–8.5 mm; length of elytra: 5.9–6.5 mm; maximum width: 5.0–5.4 mm.

#### Diagnosis.

*Tetrasericapailinensis* sp. n. is similar to *T.loeiensis* sp. n. in shape of aedeagus; *T.pailinensis* sp. n. differs from the latter by the dorsal lobe of the left paramere being twice as long as the ventral one.

#### Etymology.

The new species is named after the type locality, Pailin (adjective in the nominative singular).

### Tetraserica banhuaipoensis sp. n.

Taxon classificationAnimaliaColeopteraMelolonthidae

http://zoobank.org/8A9F9D74-65E9-4518-B507-321C68725FFB

Figures 31 , 47

#### Type material examined.

Holotype: ♂ “NW Thailand, 9.-16.V. MAE HONG SON 1991 Ban Huai Po 1600 m leg. P. Pacholátko/ coll. P. Pacholátko/ 151 Sericini Asia spec.” (CPPB). Paratypes: 1 ♂ “NW Thailand, 9.-16.V. MAE HONG SON 1991 Ban Huai Po 1600 m leg. P. Pacholátko/ coll. P. Pacholátko” (ZFMK), 1 ♂ “THAI: 29.IV.1993, PAI City; Pacholatko & Dembicky leg./ coll. Pacholátko/ 155 Sericini Asia spec.” (ZFMK), 1 ♂ “THAI: 29.IV.1993, PAI City; Pacholatko & Dembicky leg./ coll. Pacholátko” (CPPB).

#### Description.

Length of body: 8.5 mm; length of elytra: 6.4 mm; maximum width: 5.6 mm. Surface of labroclypeus and disc of frons glabrous. Smooth area anterior to eye twice as wide as long. Eyes moderately large, ratio of diameter/interocular width: 0.56. Ratio of length of metepisternum/metacoxa: 1/1.71. Metatibia short and wide, ratio width/length: 1/3.07; basal group of dorsal spines of metatibia at first third of metatibial length.

Aedeagus: Fig. 31E–G. Habitus: Fig. 31H.

Female unknown.

#### Variation.

Length of body: 6.0–8.5 mm; length of elytra: 4.7–6.4 mm; maximum width: 4.2–5.6 mm.

#### Diagnosis.

*Tetrasericabanhuaipoensis* sp. n. is similar to *T.matsumotoi* Kobayashi, 2017 and *T.kiriromensis* sp. n. in shape of the aedeagus. *T.banhuaipoensis* sp. n. differs from *T.matsumotoi* by having the right paramere distinctly shorter than median lamina of phallobase; from *T.kiriromensis* it differs by the sharply pointed apex of median phallobasal lamina and the right paramere being bent twice.

#### Etymology.

The new species is named after the type locality, Ban Huai Po (adjective in the nominative singular).

### Tetraserica nussi sp. n.

Taxon classificationAnimaliaColeopteraMelolonthidae

http://zoobank.org/578BA569-0089-47AE-BE8D-04DE7D42BC67

Figures 31 , 49

#### Type material examined.

Holotype: ♂ “Cambodia 3. Cardamom Mts., near Cham Kar Chhrey, 12°20'N, 103°01'E, 350 m, Dry Riverine in evergreen forest, 6.iii.2000, leg. M. Nuss/ Staatl. Museum für Tierkunde Dresden/ 119 Sericini Asia spec.” (SMTD). Paratypes: 1 ♂ “Coll. I. R. Sc. N. B. Cambodia, Pursat prov. Phnom Samkos W.S. Pramaoy, forest edge 13-IV-2005, Light Trap Leg. K. Smets & I. Var” (ZFMK), 1 ♂ “Coll. I. R. Sc. N. B. Cambodia, Pursat prov. Phnom Samkos W.S. Pramaoy Forest Edge 15-IV-2005, Light Trap Leg. K. Smets & I. Var” (ISNB), 1 ♂ “Coll. I. R. Sc. N. B. Cambodia, Pursat prov. Phnom Samkos W.S. Pramaoy, forest edge 13.iv. 2005, Light Trap Leg. K. Smets & I. Var” (ISNB).

#### Description.

Length of body: 8 mm; length of elytra: 5.8 mm; maximum width: 5.4 mm. Surface of labroclypeus and disc of frons glabrous. Smooth area anterior to eye twice as wide as long. Eyes small, ratio of diameter/interocular width: 0.54. Ratio of length of metepisternum/metacoxa: 1/1.68. Metatibia moderately long and wide, ratio width/length: 1/3.38; basal group of dorsal spines of metatibia at first third of metatibial length.

Aedeagus: Fig. 31I–K. Habitus: Fig. 31L.

Female unknown.

#### Variation.

Length of body: 8.0–8.1 mm; length of elytra: 5.7–5.8 mm; maximum width: 4.9–5.4 mm.

#### Diagnosis.

*Tetrasericanussi* sp. n. is very similar to *T.subrotundata* sp. n. in shape of aedeagus, but differs by the much shorter parameres and median phallobasal lamina; the dorsal lobe of left paramere is as long as the ventral one (and not shorter as in *T.subrotundata*) and the apex of phallobase is strongly asymmetric (dorsal view).

#### Etymology.

The new species is named after one of its collectors, M Nuss (noun in genitive singular).

### Tetraserica khaosoidaoensis sp. n.

Taxon classificationAnimaliaColeopteraMelolonthidae

http://zoobank.org/51AB518E-0306-4DC2-9C8D-3C4CFEBA1B16

Figures 32 , 48

#### Type material examined.

Holotype: ♂ “E Thailand, 5.-13.5. Chanthaburi Dist. Khao Soi Dao, 1998, J. Horák leg./ coll. P Pacholátko/ 129 Sericini Asia spec.” (CPPB). Paratype: 1 ♂ “E Thailand, 5.-13.5. Chanthaburi Dist. Khao Soi Dao, 1998, M Knížek lgt./ coll. P Pacholátko” (ZFMK).

#### Description.

Length of body: 7.4 mm; length of elytra: 5.6 mm; maximum width: 4.6 mm. Surface of labroclypeus and disc of frons glabrous. Smooth area anterior to eye twice as wide as long. Eyes small, ratio of diameter/interocular width: 0.54. Ratio of length of metepisternum/metacoxa: 1/1.68. Metatibia short and wide, ratio width/length: 1/3; basal group of dorsal spines of metatibia at first third of metatibial length.

**Figure 32.**
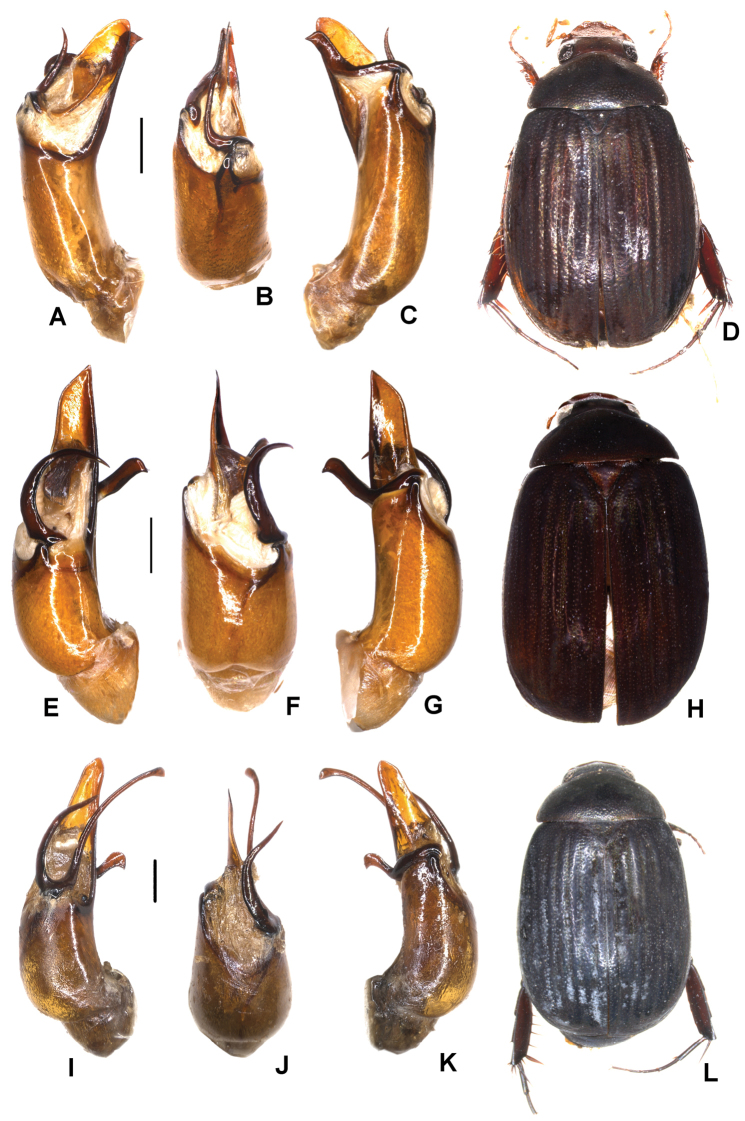
**A–D***Tetrasericakhaosoidaoensis* sp. n. (holotype) **E–H***T.pahinngamensis* sp. n. (holotype) **I–L***T.wapiensis* (Frey, 1972) (paratype) **A, E, I** aedeagus, left side lateral view **C, G, K** aedeagus, right side lateral view **B, F, J** parameres, dorsal view **D, H, L** habitus. Scale bars: 0.5 mm. Habitus not to scale.

Aedeagus: Fig. 32A–C. Habitus: Fig. 32D.

Female unknown.

#### Variation.

Length of body: 7.4–7.8 mm; length of elytra: 5.5–5.6 mm; maximum width: 4.6–4.8 mm.

#### Diagnosis.

*Tetrasericakhaosoidaoensis* sp. n. is very similar to *T.nussi* sp. n. in shape of the aedeagus; but differs by the distinctly shorter right paramere as well as the only slightly bent right paramere.

#### Etymology.

The new species is named after the type locality, Khao Soi Dao (adjective in the nominative singular).

### Tetraserica pahinngamensis sp. n.

Taxon classificationAnimaliaColeopteraMelolonthidae

http://zoobank.org/DE3FF861-BE13-4414-94B0-694795D0E5EB

Figures 32 , 49

#### Type material examined.

Holotype: ♂ “X-DA4631 labcode: VD068 THAILAND Chaiyaphum Pa Hin Ngam NP Dry evergreen forest near stream 461m Malaisie trap, 15°40, 569'N, 101°26.705'E, 13.-19.vi.2007, Katae Sa-nog & Buakaw Adnafai leg., T2479, Tetraserica spTH_V36/ X-DA4631/ sp-TH-V36” (QSBG). Paratypes: 1 ♂ “Thailand Chaiyaphum Pa Hin Ngam NP Dipterocarp forest 15°38.099'N, 101°23.921'E, 698 m Malaisie trap, 1.-7.ii.2007, Katae Sa-nog & Buakaw Adnafai T1644” (QSBG), 1 ♂ “X-DA4630 labcode: VD067 THAILAND Chaiyaphum Pa Hin Ngam NP Dry evergreen forest near stream 461m Malaisie trap, 15°40, 569'N, 101°26.705'E, 13-19.vi.2007, Katae Sa-nog & Buakaw Adnafai leg., T2479, Tetraserica spTH_V36/ X-DA4630” (ZFMK), 1 ♂ “X-DA4679 labcode: VD081 THAILAND Chaiyaphum Pa Hin Ngam NP Dry evergreen forest near stream 461m Malaisie trap, 15°40, 569'N, 101°26.705'E, 13.-19.vi.2007, Katae Sa-nog & Buakaw Adnafai leg., T2479, Tetraserica spTH_V36/ X-DA4679” (ZFMK), 1 ♂ “X-DA4680 labcode: VD082 THAILAND Chaiyaphum Pa Hin Ngam NP Dry evergreen forest near stream 461m Malaisie trap, 15°40, 569'N, 101°26.705'E, 13.-19.vi.2007, Katae Sa-nog & Buakaw Adnafai leg., T2479, Tetraserica spTH_V36/ X-DA4680” (ZFMK), 1 ♂ “X-DA4682 THAILAND Chaiyaphum Pa Hin Ngam NP Deciduous forest 357m Malaise trap 15°39.966'N, 101°27.198'E, Katae Sa-nog & Buakaw Adnafai leg. 7-13.vi.2007” (ZFMK), 1 ♂ “X-DA4683 THAILAND Chaiyaphum Pa Hin Ngam NP Deciduous forest 357m Malaise trap 15°39.966'N, 101°27.198'E, Katae Sa-nog & Buakaw Adnafai leg. 7-13.vi.2007” (ZFMK), 1 ♂ “X-DA4684 THAILAND Chaiyaphum Pa Hin Ngam NP Deciduous forest 357 m Malaise trap 15°39.966'N, 101°27.198'E, Katae Sa-nog & Buakaw Adnafai leg. 7-13.vi.2007” (ZFMK), 1 ♂ “X-DA4685 THAILAND Chaiyaphum Pa Hin Ngam NP Deciduous forest 357 m Malaise trap 15°39.966'N, 101°27.198'E, Katae Sa-nog & Buakaw Adnafai leg. 7-13.vi.2007” (ZFMK), 1 ♂ “X-DA4695 THAILAND Chaiyaphum, Pa Hin Ngam NP, Deciduous forest near stream 398 m, Malaise trap 15°40.232'N, 101°26.942'E, Katae Sa-nog & Buakaw Adnafai leg. 19.-20.vi.2007” (ZFMK), 1 ♂ “X-DA4701 THAILAND Chaiyaphum Pa Hin Ngam NP Deciduous forest 357m Malaise trap 15°39.966'N, 101°27.198'E, Katae Sa-nog & Buakaw Adnafai leg. 1.-7.vi.2007” (ZFMK), 1 ♂ “X-DA4707 THAILAND Chaiyaphum Tat Tone NP Streamside at Tat Fah waterfall 242m Malaise trap 15°56.463'N, 102°05.953'E, Tawit Jaruphan & Orawan Budsawong leg. 26.iii.-2.iv.2007” (ZFMK), 1 ♂ “Thailand, Prov. Prachin Buri, Sakaerat Ecol. Research Institute,/ No. 25 at light 1.VI.2001 E. Horaváth & Gy. Sziráki” (HNHM).

#### Description.

Length of body: 8.1 mm; length of elytra: 6.4 mm; maximum width: 5.1 mm. Surface of labroclypeus and disc of frons glabrous. Smooth area anterior to eye twice as wide as long. Eyes small, ratio of diameter/interocular width: 0.52. Ratio of length of metepisternum/metacoxa: 1/1.92. Metatibia short and wide, ratio width/length: 1/2.87; basal group of dorsal spines of metatibia at first third of metatibial length.

Aedeagus: Fig. 32E–G. Habitus: Fig. 32H.

Female unknown.

#### Variation.

Length of body: 7.6–8.9 mm; length of elytra: 5.6–6.9 mm; maximum width: 4.9–5.8 mm.

#### Diagnosis.

*Tetrasericapahinngamensis* sp. n. is very similar to *T.wapiensis* (Frey, 1972) in shape of aedeagus, differing by the left paramere being simple rather than divided into two long and narrow filiform lobes.

#### Etymology.

The new species is named with reference to its occurrence in the Pa Hin Ngam National Park (adjective in the nominative singular).

### Tetraserica wapiensis

Taxon classificationAnimaliaColeopteraMelolonthidae

(Frey, 1972) comb. n.

Figures 32 , 54

Neoserica wapiensis Frey, 1972: 203, fig. 66.

#### Type material examined.

Paratype: 1 ♂ “Süd Laos, Wapi, 1967/ Paratype Neosericawapiensis G. Frey 1971” (CF).

#### Additional material examined.

**Thailand**: 1 ♂ “Thail.: Ko Samui Coral Cove; Light 0-10 NN 23.4.1991/ leg. F. Hieß/ Coll. Dirk Ahrens” (ZFMK), 1 ♂ “836978/ 836978 Thailand Phetchabun Nam Nao NP Hill evergreen forest 838 m 9.-16.vi.2007 Leng Janteab leg. Tetraserica TIGERsp1” (QSBG), 1 ♂ “836987/ 836987 Thailand Phetchabun Nam Nao NP Hill evergreen forest 838 m 16-23.vi.2007 Noopean Hongyothi leg. Tetraserica TIGERsp1b” (QSBG), 1 ♂ “X-DA2449 labcode: VD010 THAILAND Petchabun Nam Nao NP Hill evergreen forest 838 m 16°44.387'N, 101°34.531'E, 2.-9.vi.2007, Noopean Hongyothi leg. T2433, Tetraserica TIGER sp1” (QSBG), 1 ♂ “X-DA4646 labcode: VD074 THAILAND Petchabun Nam Nao NP Hill evergreen forest 838 m Malaise trap, 16°44.387'N, 101°34.531E, 9-16.vi.2007, Leng Jantaleb leg. T2438, Tetraserica spTH_V41” (QSBG), 1 ♂ “X-DA4650 labcode: VD077 THAILAND Petchabun Nam Nao NP Hill evergreen forest 838m Malaise trap, 16°44.387'N, 101°34.531E, 9-16.vi.2007, Noopean Hongyothi leg. T 2436, Tetraserica spTH_V41/2” (QSBG), 1 ♂ “X-DA4651 labcode: VD078 THAILAND Petchabun Nam Nao NP Hill evergreen forest 838 m Malaise trap, 16°44.387'N, 101°34.531E, 9-16.vi.2007, Noopean Hongyothi leg. T2436, Tetraserica spTH_V42” (QSBG), 1 ♂ “X-DA4676 labcode: VD080 THAILAND Petchabun Nam Nao NP Hill evergreen forest 838 m Malaise trap, 16°44.387'N, 101°34.531E, 26.v.-2.vi.2007, Noopean Hongyothi leg. T2430, Tetraserica spTH_V42” (QSBG), 1 ♂ “X-DA4690 labcode: VD084 THAILAND Petchabun Nam Nao NP Hill evergreen forest 834m Malaise trap, 16°44.371'N, 101°34.549'E, 29.v.-2.vi.2007, Leng Jantaleb leg. T2429, Tetraserica spTH_V41” (QSBG), 1 ♂ “X-DA4708 labcode: VD090 THAILAND Petchabun Nam Nao NP Hill evergreen forest 883m Malaise trap, 16°44.402'N, 101°34.560'E, 26.v.-2.vi.2007, Leng Jantaleb leg. T2431, Tetraserica spTH_V41” (QSBG), 1 ♂ “X-DA4709 labcode: VD091 THAILAND Petchabun Nam Nao NP Hill evergreen forest 883 m Malaise trap, 16°44.402'N, 101°34.560'E, 26.v.-2.vi.2007, Leng Jantaleb leg. T2431, Tetraserica spTH_V42” (QSBG), 1 ♂ “X-DA4710 labcode: VD092 THAILAND Petchabun Nam Nao NP Hill evergreen forest 883 m Malaise trap, 16°44.402'N, 101°34.560'E, 26.v.-2.vi.2007, Leng Jantaleb leg. T2431, Tetraserica spTH_V42” (QSBG), 1 ♂ “X-DA4711 labcode: VD092 THAILAND Petchabun Nam Nao NP Hill evergreen forest 883 m Malaise trap, 16°44.402'N, 101°34.560'E, 26.v.-2.vi.2007, Leng Jantaleb leg. T2431, Tetraserica spTH_V42” (ZFMK). **Laos**: 1 ♂ “LAOS, SEKONG prov. Ca. 12 km S Sekong TAD FAEK waterfalls (at light) 15°14.7'N, 106°45.1'E, 118 m Jiři Hájek leg. 8.+12.v.2010/ 956 Sericini Asia spec.” (NMPC).

#### Redescription.

Length of body: 7.5 mm; length of elytra: 5.5 mm; maximum width: 4.8 mm. Surface of labroclypeus and disc of frons glabrous. Smooth area anterior to eye twice as wide as long. Eyes small, ratio of diameter/interocular width: 0.45. Ratio of length of metepisternum/metacoxa: 1/1.67. Metatibia moderately long and wide, ratio width/length: 1/3.58; basal group of dorsal spines of metatibia at first third of metatibial length.

Aedeagus: Fig. 32I–K. Habitus: Fig. 32L.

#### Remarks.

This species was originally described from Laos, and is now reported from Thailand.

### Tetraserica auriculata sp. n.

Taxon classificationAnimaliaColeopteraMelolonthidae

http://zoobank.org/F3EEC3C3-B94A-46DA-A124-D755C513DD6F

Figures 33 , 46

#### Type material examined.

Holotype: ♂ “X-DA4700 labcode: VD089 CAMBODIA Sihanouk-ville 20–40 m, 20.-30.iv.2008, S. Murzin leg. Tetraserica spCA_V50/ X-DA4700/ sp-CA-V50” (ZFMK).

#### Description.

Length of body: 8.1 mm; length of elytra: 6.6 mm; maximum width: 5.4 mm. Surface of labroclypeus and disc of frons glabrous. Smooth area anterior to eye twice as wide as long. Eyes small, ratio of diameter/interocular width: 0.5. Ratio of length of metepisternum/metacoxa: 1/1.73. Metatibia short and wide, ratio width/length: 1/3; basal group of dorsal spines of metatibia at first third of metatibial length.

**Figure 33.**
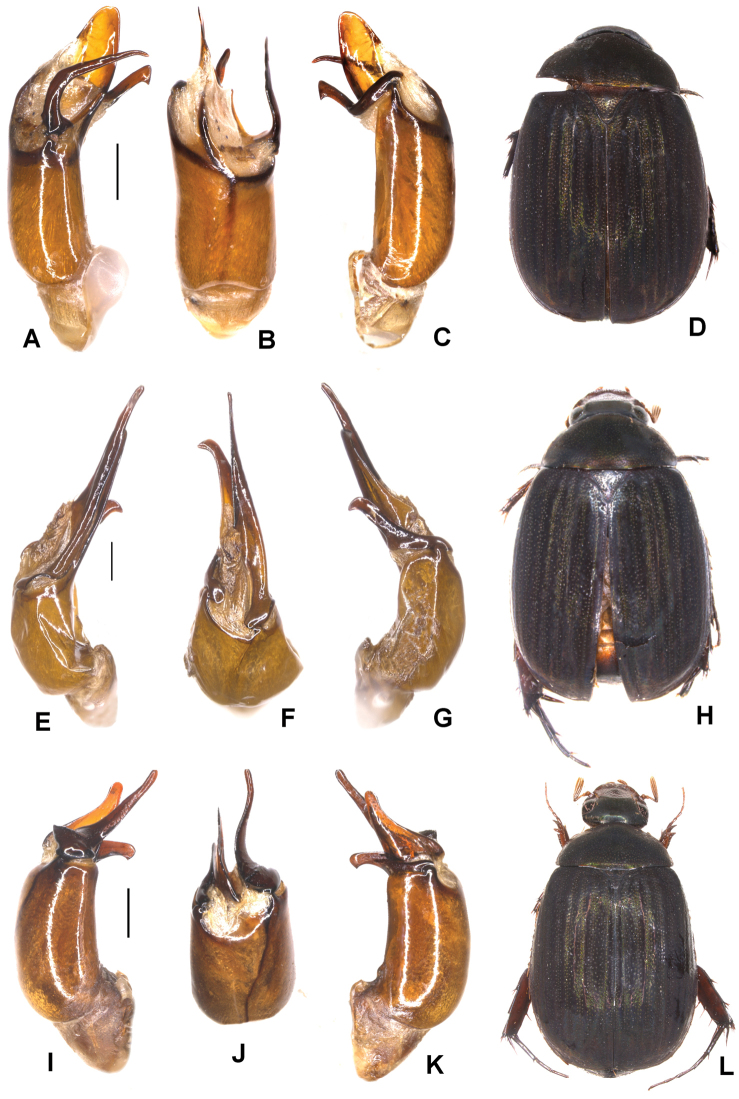
**A–D***Tetrasericaauriculata* sp. n. (holotype) **E–H***T.semipingjiangensis* sp. n. (holotype) **I–L***T.laotica* (Frey, 1972) (holotype) **A, E, I** aedeagus, left side lateral view **C, G, K** aedeagus, right side lateral view **B, F, J** parameres, dorsal view **D, H, L** habitus. Scale bars: 0.5 mm. Habitus not to scale.

Aedeagus: Fig. 33A–C. Habitus: Fig. 33D.

Female unknown.

#### Diagnosis.

*Tetrasericaauriculata* sp. n. is similar to *T.pailinensis* sp. n. in shape of aedeagus, from which *it* differs by the dorsal lobe of the left paramere being distinctly shorter than the median lamina of phallobase and the right paramere being narrower (in lateral view) and distinctly shorter than median lamina of phallobase. In *T.pailinensis* sp. n. the dorsal lobe of left paramere is distinctly exceeding the median lamina of phallobase and the right paramere is wider (in lateral view) and a little shorter than median lamina of phallobase.

#### Etymology.

The name of the new species (adjective in nominative singular) is derived from the Latin adjective *auriculatus* (ear-shaped), with reference to the ear-shaped median apical lamella of phallobase.

### Tetraserica semipingjiangensis sp. n.

Taxon classificationAnimaliaColeopteraMelolonthidae

http://zoobank.org/630CA170-72D5-434D-A209-61ECA48C954C

Figures 33 , 50

#### Type material examined.

Holotype: ♂ “N THAILAND III.1992, 3 km W Ban Rai 170 km NW Bankok 150 m NN lg. Malicky/ Zoologische Staatssammlung München” (ZSM).

#### Description.

Length of body: 9.6 mm; length of elytra: 7.1 mm; maximum width: 6.3 mm. Surface of labroclypeus and disc of frons glabrous. Smooth area anterior to eye twice as wide as long. Eyes small, ratio of diameter/interocular width: 0.5. Ratio of length of metepisternum/metacoxa: 1/1.72. Posterior margin of metafemur with blunt tooth. Metatibia short and wide, ratio width/length: 1/3.13; basal group of dorsal spines of metatibia at first third of metatibial length.

Aedeagus: Fig. 33E–G. Habitus: Fig. 33H.

Female unknown.

#### Diagnosis.

*Tetrasericasemipingjiangensis* sp. n. differs from *T.pingjiangensis* Liu et al., 2014 by the simple right paramere, which is not split in two lobes.

#### Etymology.

The species name (adjective in the nominative singular) is derived from the combined Latin words *semi* (half) and the species name *pingjiangensis*, with reference to the similarity to *T.pingjiangensis*.

### Tetraserica laotica

Taxon classificationAnimaliaColeopteraMelolonthidae

(Frey, 1972) comb. n.

Figures 33 , 49

Neoserica laotica Frey, 1972: 202, fig. 65.
Tetraserica midoriae Kobayashi, 2017: 38, figs 5, 14, **syn. n.**

#### Type material examined.

Holotype: ♂ “Laos, V 1967 Ban-Van-Eue/ Type Form1 Neosericalaotica G. Frey 1971” (CF). Paratypes: 1 ♀ “Laos, V 1967 Ban Van Eua/ Paratype Neosericalaotica G. Frey 1971” (CF), 1 ♀ “Laos: Sedone Prov. Pakse, 15.V.1965/ Paratype Neosericalaotica G. Frey 1971” (CF).

#### Additional material examined.

1 ♂ “Laos: Vientiane Prov. Ban Van Eue 31.V.1966” (BPBM), 1 ♂ “Laos: Vientiane Prov. Ban Van Eue 13-15.IV.1965” (BPBM), 1 ♂ “LAOS Bolikhamxai pr. 18°16 N, 103°11 E 70 Km NEE Vientiane 27-30.iv.1997, 150 m, Vít Kubáň leg./ coll. P Pacholátko” (CPPB), 1 ♂ “LAOS Bolikhamxai pr. 18°16 N, 103°11 E 70 Km NEE Ventiame 2-3.iv.1997, 150 m, Vít Kubáň leg./ coll. P Pacholátko” (CPPB), 2 ♂♂ “Laos, Bolikhamsay Prov. Phou Khao Kuay NBCA Tad Leuk Waterfall, 280 m/ at light No 46, 11-12.IV.1998 leg. O Merkl & G Csorba” (HNHM).

#### Redescription.

Length of body: 9.8 mm; length of elytra: 6.9 mm; maximum width: 5.6 mm. Surface of labroclypeus and disc of frons glabrous. Smooth area anterior to eye twice as wide as long. Eyes small, ratio of diameter/interocular width: 0.48. Ratio of length of metepisternum/metacoxa: 1/1.73. Metatibia short and wide, ratio width/length: 1/3.06; basal group of dorsal spines of metatibia at first third of metatibial length.

Aedeagus: Fig. 33I–K. Habitus: Fig. 33L.

#### Remarks.

The shape of genitalia of the type specimens of T.*midoriae* Kobayashi, 2017 is virtually identical with that of the holotype of *Tetrasericalaotica* (Frey, 1972). Two female paratype specimens (1 ♀ “Laos, V 1967 Ban Van Eua/ Paratype Neosericalaotica G. Frey 1971” (CF), 1 ♀ “Laos: ban Van Heue 20km E of Phou-kow-kuei, 1-15V.1965/ Paratype Neosericalaotica G. Frey 1971” (CF),) do not belong to this species, as they have both a carinate hypomeron, and are likely to be assigned to the genus *Maladera*.

### Tetraserica ruiliensis

Taxon classificationAnimaliaColeopteraMelolonthidae

Liu, Fabrizi, Bai, Yang & Ahrens, 2014

Figures 34 , 54

Tetraserica ruiliensis Liu, Fabrizi, Bai, Yang & Ahrens, 2014: 113, fig. 9A–D.

#### Material examined.

2 ♂♂ “NW Thailand, 1-7.V.1992, Mae Hong Song Ban Si Lang 1000m, S. Bily leg.” (NHMB), 1 ♂ “Thailand bor. Occ. 30.04.-14.05.1991 Mae Hong Son env. Ban Huai Po, 1800 m Jan Farkac leg.” (NHMB), 1 ♂ “Birmania N.S.S. Lashio 1939 G. Salsone/ MSN Milano” (MSNM), 1 ♂ “S Vietnam, 1-15. 5. 1994 Nam Cat Tien-Nat. park, P. Pacholátko & L. Dembický leg. / coll. P. Pacholátko” (CPPB), 3 ♂♂ “S Vietnam, (Cat Tien) 120 km NNE Ho Chi Minh, Cat Tien Nat. Park, 7.-21.6.1995 lg. A. Napolov” (CNAR, ZFMK), 1 ♂ “S. Vietnam (Cat Tien) 120 km NNE Ho Chi Minh, Cat Tien Nat. Park 8.-17.7.1995 leg. A. Napolov” (CNAR), 1 ♂ “Museum Leiden Viet nam (Dong Nai Prov.) Cat Tien N.P.: near guesthouse, 13-v-2007. Leg. E. Gassó Miracle & Nguyen Than Mahn/ secondary humid lowland forest; light trap (ML), 18–20 hrs, 11°26'20.6"N, 107°25'42"E” (RMNH), 5 ♂♂ “Museum Leiden Viet nam (Dong Nai Prov.) Cat Tien N.P.: Botanical Garden, 13-20.v.2007. Leg. C. van Achterberg, R. de Vries & E. Gassó Miracle/ mixed bamboo and wood forest; in malaise trap, 250 m, 11°26'23.8"N, 107°25'44"E” (RMNH), 3 ♂♂ “Museum Leiden Viet nam (Dong Nai Prov.) Cat Tien N.P.: Eco trail, 17-v-2007. Leg. E. Gassó Miracle & Nguyen Than Mahn/ primary lowland forest; light trap (ML), 19:30 -21:30 hrs, 11°26'54.9"N, 107°26'30.9"E” (RMNH), 1 ♂ “Museum Leiden Viet nam (Dong Nai Prov.) Cat Tien N.P.: Eco trail, 14-20.v.2007. Leg. C. van Achterberg, R. de Vries & E. Gassó Miracle/ primary humid lowland forest; in malaise trap, 250 m, 11°26'54.9"N, 107°26'30.9"E” (RMNH), 2 ♂♂ “S Vietnam: Lam Dong Prov., Cat Tien Distr. Nam Cat Tien National Park, Headquarter area (120 m a.s.l.) 11-15.VI.2015 at light/ legit L. Bartolozzi, G. Chelazzi, S. Bambi, F. Fabiano, E. Orbach, V. Sbordoni (n. Magazzino 3023)” (MZUF).

**Figure 34.**
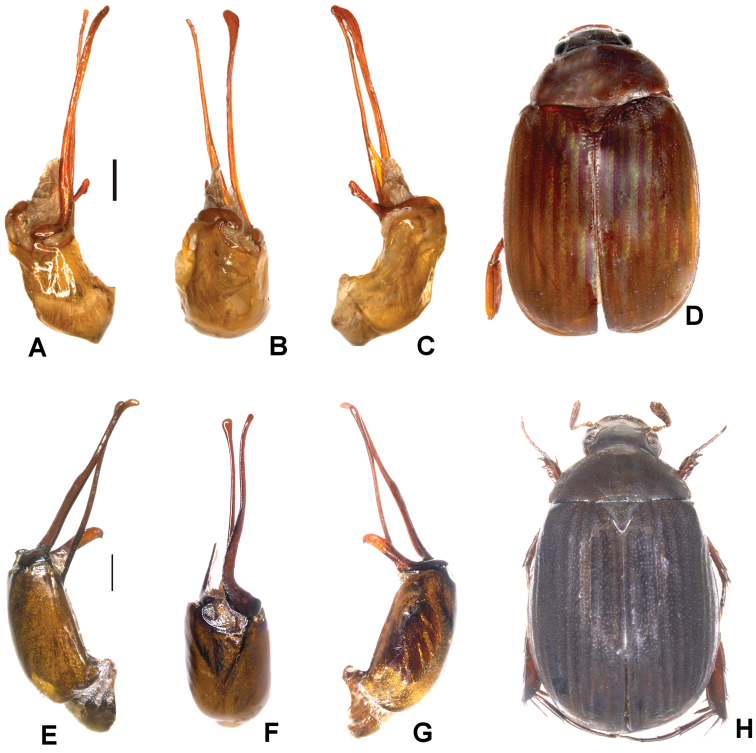
**A–D***Tetrasericaruiliensis* Liu et al., 2014 (holotype) **E–H***T.pseudoruiliensis* sp. n. (holotype) **A, E** aedeagus, left side lateral view **C, G** aedeagus, right side lateral view **B, F** parameres, dorsal view **D, H** habitus. Scale bars: 0.5 mm. Habitus not to scale.

Aedeagus: Fig. 34A–C. Habitus: Fig. 34D.

### Tetraserica pseudoruiliensis sp. n.

Taxon classificationAnimaliaColeopteraMelolonthidae

http://zoobank.org/2DBE2CE8-C461-4BEE-AF9E-CB1561AC7A80

Figures 34 , 54

#### Type material examined.

Holotype: ♂ 1 ♂ “BURMA (Myanmar) SW Shan state INLE lake - NYAUNGSHWE J. Rejsek 7.-16.6.1997/ coll. Dirk Ahrens/ 148 Sericini Asia spec.” (ZFMK). Paratypes: 8 ♂♂ “NW Thailand, 30.4.1992, Mae Hong Song 350 m, S. Bily leg./ NHM Basel/ 121 Sericini Asia spec.” (NHMB), 1 ♂ “X-DA4637/ X-DA4637 labcode: VD071, THAILAND, Chiang Dao Hill Resort 600 m, 19.55779N 99.0766E, 8.-12.vi.2011, M. Murzin, O. Shulga leg., Tetraserica spTH_V38/ sp LAV8” (ZFMK), 1 ♂ “NW Thailand: MAE HOG SON 29.-30.4.1992 J. Horak leg./ coll. P. Pacholátko” (CPPB), 1 ♂ “THAI, 9.-14.V.1991 CHIANG DAO 350m 19°22’ N 98°57'E, Vit Kubáň leg./ Thailand 91 “Thanon Thong Chai” D. Kral & V. Kubáň/ coll. P. Pacholátko” (CPPB), 4 ♂♂ “NE Thailand, 28.4.1991, Mae Hong Son S. Bily leg.” (NHMB), 16 ♂♂ “Thailand 9.-14.V.1991 Chiang Dao 350 m 19°22'N, 98°57'E, V. Kuban lg./ coll. Milan Nikodym, Praha” (ZFMK), 17 ♂♂, 3 ♀♀ “Thailand 9.-16.5.1991 Mae Hong Son Ban Busi Po 1600–2000 m J. Horak lgt./ coll. Milan Nikodym, Praha” (ZFMK), 25 ♂♂, 28 ♀♀ “Thai 9-14.5.1991 Chiang Dao 350 m 19°22'N, 98°57'E, D. Kral lgt.” (NMPC), 1 ♂ “X-DA4604/ X-DA4604 labcode: VD061, LAOS, Stupa GH, 5km W Muang Sing, 750 m, 21.1482N 101.1711E, 9.v-2.vi.2011, M. Murzin, O. Shulga leg. Tetraserica spLA_V34” (ZFMK), 1 ♂ “X-DA4605/ X-DA4605 labcode: VD062, LAOS, Stupa GH, 5km W Muang Sing, 750 m, 21.1482N 101.1711E, 9.v-2.vi.2011, M. Murzin, O. Shulga leg. Tetraserica spLA_V34” (ZFMK), 1 ♂ “X-DA4610/ X-DA4610 labcode: VD063, LAOS, Stupa GH, 5km W Muang Sing, 750 m, 21.1482N 101.1711E, 9.v-2.vi.2011, M. Murzin, O. Shulga leg. Tetraserica spLA_V34” (ZFMK), 1 ♂ “X-DA4612/ X-DA4612 labcode: VD064, LAOS, Stupa GH, 5km W Muang Sing, 750 m, 21.1482N 101.1711E, 9.v-2.vi.2011, M. Murzin, O. Shulga leg. Tetraserica spLA_V34” (ZFMK), 1 ♂ “X-DA4623/ X-DA4623 labcode: VD065, LAOS, Stupa GH, 5km W Muang Sing, 750 m, 21.1482N 101.1711E, 9.v-2.vi.2011, M. Murzin, O. Shulga leg. Tetraserica spLA_V34” (ZFMK), 1 ♂ “X-DA4636/ X-DA4636 labcode: VD070, LAOS, Stupa GH, 5 km W Muang Sing, 750 m, 21.1482N 101.1711E, 9.v-2.vi.2011, M. Murzin, O. Shulga leg. Tetraserica spLA_V38” (ZFMK), 1 ♂ “X-DA4519/ X-DA4519 labcode: VD035, LAOS NW, h=750 m 5 km W Muang Sing Chiang Tung (Stupa), GH 9-14.VI.2010, S. Murzin Tetraserica spLA_V8” (ZFMK), 1 ♂ “X-DA4520/ X-DA4520 labcode: VD036, LAOS NW, h=750 m 5 km W Muang Sing Chiang Tung (Stupa), GH 9-14.VI.2010, S. Murzin Tetraserica spLA_V8” (ZFMK), 1 ♂ “X-DA4529/ X-DA4529 labcode: VD038, LAOS NW, h=750 m 5 km W Muang Sing Chiang Tung (Stupa), GH 9-14.VI.2010, S. Murzin Tetraserica spLA_V8” (ZFMK), 1 ♂ “X-DA4536/ X-DA4536 labcode: VD035, LAOS NW, h=750 m 5 km W Muang Sing Chiang Tung (Stupa), GH 9-14.VI.2010, S. Murzin Tetraserica spLA_V8” (ZFMK), 1 ♂ “X-DA4732 LAOS Stupa GH, 5km W Muang Sing, 750 m, 21.1482N 101.1711E 9.v.-2.vi.2011 M. Murzin, O. Shulga leg.” (ZFMK), 1 ♂ “X-DA4735 LAOS Stupa GH, 5 km W Muang Sing, 750 m, 21.1482N 101.1711E 9.v.-2.vi.2011 M. Murzin, O. Shulga leg.” (ZFMK), 1 ♂ “X-DA4738 LAOS Stupa GH, 5km W Muang Sing, 750 m, 21.1482N 101.1711E 9.v.-2.vi.2011 M. Murzin, O. Shulga leg.” (ZFMK).

#### Description.

Length of body: 10.5 mm; length of elytra: 7.8 mm; maximum width: 6.4 mm. Surface of labroclypeus and disc of frons glabrous. Smooth area anterior to eye twice as wide as long. Eyes small, ratio of diameter/interocular width: 0.51. Ratio of length of metepisternum/metacoxa: 1/1.56. Metatibia short and wide, ratio width/length: 1/2.75; basal group of dorsal spines of metatibia at first third of metatibial length.

Aedeagus: Fig. 34E–G. Habitus: Fig. 34H.

Female unknown.

#### Variation.

Length of body: 10.0–10.5 mm; length of elytra: 7.2–7.8 mm; maximum width: 6.1–6.4 mm.

#### Diagnosis.

*Tetrasericapseudoruiliensis* sp. n. differs from the similar *T.gestroi* (Brenske, 1898) by the right paramere which is much less than one third as long as left paramere. The new species differs from *T.ruiliensis* Liu et al., 2014 by the left paramere being straight rather than bent at the base.

#### Etymology.

The species name (adjective in the nominative singular) is derived from the combined Greek prefix *pseudo*- (false) and the species name *ruiliensis*, with reference to the similarity to *T.ruiliensis* Liu et al.

### Tetraserica semiruiliensis sp. n.

Taxon classificationAnimaliaColeopteraMelolonthidae

http://zoobank.org/4B345E55-B2A8-4CC9-A049-DFC2F71D4108

Figures 35 , 51

#### Type material examined.

Holotype: ♂ “E Thailand, Chanta Buri, Pawa Kaeng Hang Naw, Khao Krok; 300 m; 25.-26.IV.1997; leg. Sadahiro Ohmomo” (ZFMK). Paratypes: 1 ♂ “E Thailand, Chanta Buri, Pawa Kaeng Hang Naw, Khao Krok; 300 m; 25.-26.IV.1997; leg. Sadahiro Ohmomo” (ZFMK), 4 ♂♂ “E Thailand, Chanta Buri, Houw Tabmon Rayong; Khao Chamao; 400 m; 28.IV.1997” (ZFMK), 2 ♂♂ “E Thailand, 5.-13.5- Chanthaburi Dist, Khao Soi Dao, 1998 J. Horák leg/ coll. P. Pacholátko” (CPPB), 2 ♂ “Coll. I. R. Sc. N. B., Cambodia, Pursat pr., Phnom Samkos Wildlife Sanctuary, Pramaoy, 16.XI.2005, forest edge, light trapping Leg. K. Smets & I. Var” (ISNB), 5 ♂♂ “Coll. I. R. Sc. N. B., Cambodia, Pursat Prov Phnom Samkos W.S. Pramaoy Forest Edge, 15-IV-2005, Light Trap Leg. K. Smets & I. Var” (ISNB), 4 ♂♂ “Coll. I. R. Sc. N. B., Cambodia, Pursat prov. Phnom Samkos W.S., forest edge, light trap 14.iv.2005 Leg. K. Smets & I. Var” (ISNB), 3 ♂♂ “Coll. I. R. Sc. N. B., Cambodia (Pursat Prov.) Phnom Samkos wildlife Sanctuary, light trap Forest Edge & Primary 14-IV-2005 Leg. K. Smets & I. Var” (ISNB).

#### Description.

Length of body: 10 mm; length of elytra: 7.1 mm; maximum width: 5.9 mm. Surface of labroclypeus and disc of frons glabrous. Smooth area anterior to eye twice as wide as long. Eyes moderately large, ratio of diameter/interocular width: 0.59. Ratio of length of metepisternum/metacoxa: 1/1.84. Metatibia short and wide, ratio width/length: 1/2.84; basal group of dorsal spines of metatibia at first third of metatibial length.

**Figure 35.**
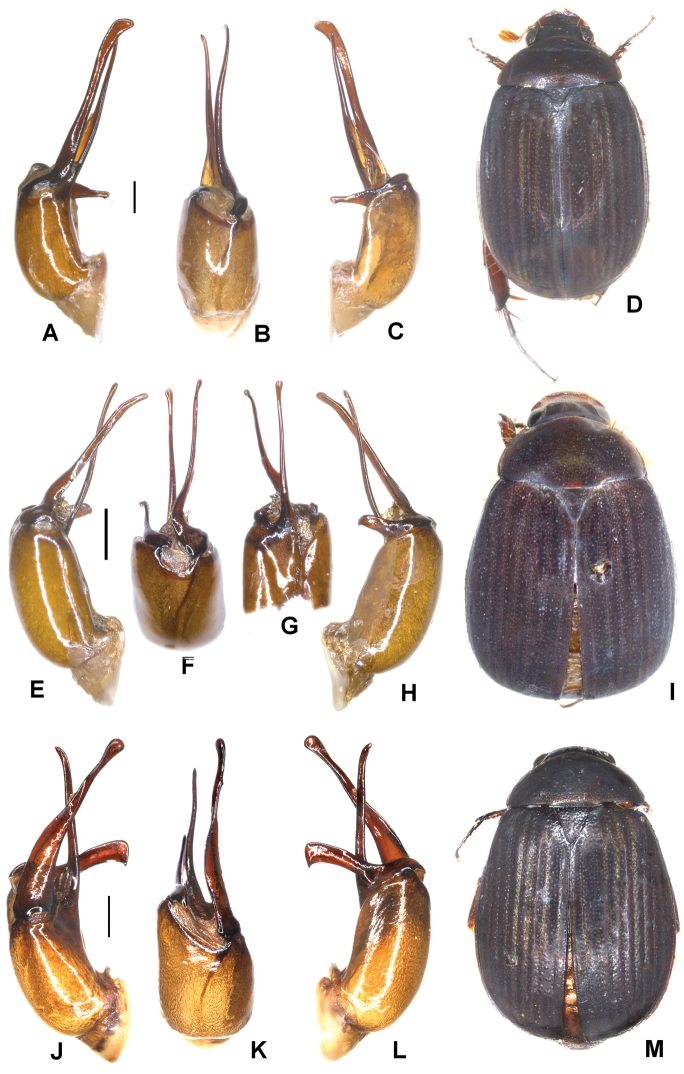
**A–D***Tetrasericasemiruiliensis* sp. n. (holotype) **E–I***T.gestroi* (Brenske, 1898) (lectotype) **J–M***T.desalvazzai* (holotype) **A, E, J** aedeagus, left side lateral view **C, H, L** aedeagus, right side lateral view **B, F, K** parameres, dorsal view **G** parameres, ventral view **D, I, M** habitus. Scale bars: 0.5 mm. Habitus not to scale.

Aedeagus: Fig. 35A–C. Habitus: Fig. 35D.

Female unknown.

#### Variation.

Length of body: 9.5–10.0 mm; length of elytra: 6.5–7.1 mm; maximum width: 5.5–5.9 mm.

#### Diagnosis.

*Tetrasericasemiruiliensis* sp. n. differs from the similar *T.ruiliensis* Liu et al., 2014 by the nearly straight right paramere (lateral view), which is moderately bent at middle (lateral view) in *T.ruiliensis*.

#### Etymology.

The species name (adjective in the nominative singular) is derived from the combined Latin prefix *semi*- (half) and the species name *ruiliensis*, with reference to the similarity to *T.ruiliensis* Liu et al.

### Tetraserica gestroi

Taxon classificationAnimaliaColeopteraMelolonthidae

(Brenske, 1898)

Figures 35 , 47

Neoserica gestroi Brenske, 1898: 347.
Tetraserica gestroi : Ahrens 2004: 168.

#### Type material examined.

Lectotype (here designated): ♂ “Teinzo Birmania Fea Maggio 1886/ ♂/ Sericagestroi Type Brsk./ Typus/ Gestroi Brsk./ S. Gestroi Brsk. typus!” (MSNG). Paralectotypes: 2 ♂♂ “Teinzo Birmania Fea Maggio 1886/ Gestroi Type Brsk./ ♂” (MSNG), 1 ♀ “Teinzo Birmania Fea Maggio 1886/ ♀/ Gestroi Type Brsk./ *gestroi* Brs.” (ZMHB).

#### Redescription.

Length of body: 9.5 mm; length of elytra: 7.1 mm; maximum width: 6.4 mm. Surface of labroclypeus and disc of frons glabrous. Smooth area anterior to eye twice as wide as long. Eyes small, ratio of diameter/interocular width: 0.51. Ratio of length of metepisternum/metacoxa: 1/1.68. Metatibia short and wide, ratio width/length: 1/2.8; basal group of dorsal spines of metatibia at first third of metatibial length.

Aedeagus: Fig. 35E–H. Habitus: Fig. 35I.

Female: Antennal club with three antennomeres, as long as remaining antennomeres combined. Exes smaller than in male, ratio of diameter/interocular width: 0.55. Pygidium flat.

### Tetraserica desalvazzai sp. n.

Taxon classificationAnimaliaColeopteraMelolonthidae

http://zoobank.org/75EAA1F9-F6D9-443D-BC58-638430A393F9

Figures 35 , 47

#### Type material examined.

Holotype: ♂ “Haut Mekong Nam Tha 9.IV.1918. R.V. de Salvazza/ Brit Mus. 1921-89./ 1019 Asia Sericini spec.” (NHMUK). Paratype 1 ♂ “Haut Mekong Weng Vai 23.V.1918, R.V. de Salvazza/ Brit Mus. 1921-89./ 1019 Asia Sericini spec.” (ZFMK).

#### Description.

Length of body: 7.6 mm; length of elytra: 6.0 mm; maximum width: 4.7 mm. Surface of labroclypeus and disc of frons glabrous. Smooth area anterior to eye twice as wide as long. Eyes large, ratio of diameter/interocular width: 0.72. Ratio of length of metepisternum/metacoxa: 1/1.62. Metatibia moderately long and wide, ratio width/length: 1/3.1; basal group of dorsal spines of metatibia at first third of metatibial length.

Aedeagus: Fig. 35J–L. Habitus: Fig. 35M.

Female unknown.

#### Variation.

Length of body: 7.6–9.1 mm; length of elytra: 6.0–6.5 mm; maximum width: 4.7–5.0 mm.

#### Diagnosis.

*Tetrasericadesalvazzai* sp. n. is rather similar to *T.gestroi*, from which the new species differs by the left paramere being straight at base, and both parameres being generally more robust.

#### Etymology.

The new species is named after the collector, RV de Salvazza (noun in genitive singular).

### Tetraserica phoupaneensis sp. n.

Taxon classificationAnimaliaColeopteraMelolonthidae

http://zoobank.org/53992DA5-8CA1-4B27-BF5F-329FD5065D14

Figures 36 , 51

#### Type material examined.

Holotype: ♂ “LAOS-NE, Houa Phan prov., 20°11–13'N, 103°59'–104°01'E, Ban Saluei -> Phou Pane Mt., 9.-17.vi.2009, 1300–1900 m, Michael Geiser leg./ NHMB Basel, NMPC Prague, Laos 2009, Expedition: M Brancucci, M Geiser, Z Kraus, D Hauck, V Kubáň/ 912 Sericini Asia spec.” (NHMB).

#### Description.

Length of body: 8.3 mm; length of elytra: 5.9 mm; maximum width: 4.5 mm. Surface of labroclypeus and disc of frons glabrous. Smooth area anterior to eye twice as wide as long. Eyes small, ratio of diameter/interocular width: 0.54. Ratio of length of metepisternum/metacoxa: 1/1.6. Posterior margin of metafemur with blunt tooth. Metatibia moderately long and wide, ratio width/length: 1/3.5; basal group of dorsal spines of metatibia at first third of metatibial length.

**Figure 36.**
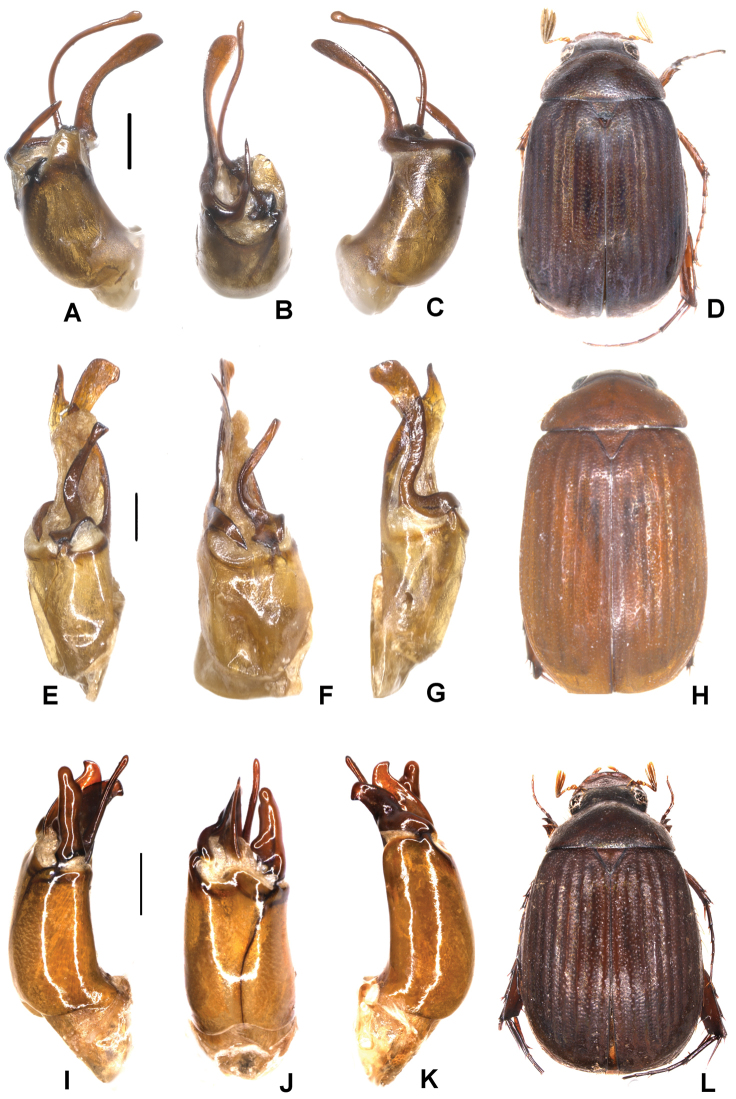
**A–D***Tetrasericaphoupaneensis* sp. n. (holotype) **E–H***T.vietnamensis* (Frey, 1972) (holotype) **I–L***T.nonglomensis* sp. n. (holotype) **A, E, I** aedeagus, left side lateral view **C, G, K** aedeagus, right side lateral view **B, F, J** parameres, dorsal view **D, H, L** habitus. Scale bars: 0.5 mm. Habitus not to scale.

Aedeagus: Fig. 36A–C. Habitus: Fig. 36D.

Female unknown.

#### Diagnosis.

*Tetrasericaphoupaneensis* sp. n. is similar to *T.vietnamensis* (Frey, 1972); the new species differs by the left paramere being as long as phallobase and by the right paramere having a longer basal lobe which is directed distally. In *T.vietnamensis* the left paramere is only half as long as phallobase, and the right paramere has no basal lobe.

#### Etymology.

The new species is named after the type locality, Mt Phou Pane (adjective in the nominative singular).

### Tetraserica vietnamensis

Taxon classificationAnimaliaColeopteraMelolonthidae

(Frey, 1969) comb. n.

Figures 36 , 51

Neoserica vietnamensis Frey, 1969: 109, fig. 2b.

#### Type material examined.

Holotype: ♂ “Dalat S. Vietnam/ Typus/ Typus Neosericavietnamensis n.sp. G. Frey 1967” (CF). Paratypes: 1 ♂ “Dalat 1966 S. Vietnam/ Paratype/ Neosericavietnamensis ♂ n. sp. G. Frey 1967” (CF).

#### Additional material examined.

1 ♂ “Dalat 1966 S. Vietnam/ Neosericavietnamensis Frey” (CF), 1 ♂ “Museum Leiden Viet Nam (Dak Lak Prov.) Chu Yang Sin N.P.: 6–8 km S of Dam construction-site. 1-10.vi.2007. Leg. C. van Achterberg, R. de Vries & E. Gassó Miracle/ primary evergreen forest near stream; in malaise traps; 750 m, 12°26'26.3"N, 108°19'5"E” (RMNH), 1 ♂ “S Vietnam, 12.03N 108.27E 12 km N of Dalat-Lang Bian, 1580–1750 m, 17-21.iv.1999, Pacholátko & Dembický leg./ coll. P. Pacholátko/ VS 72” (CPPB).

#### Redescription.

Length of body: 10.5 mm; length of elytra: 7.9 mm; maximum width: 6.1 mm. Surface of labroclypeus and disc of frons glabrous. Smooth area anterior to eye twice as wide as long. Eyes small, ratio of diameter/interocular width: 0.55. Ratio of length of metepisternum/metacoxa: 1/1.69. Posterior margin of metafemur with blunt tooth. Metatibia short and wide, ratio width/length: 1/3.06; basal group of dorsal spines of metatibia at first third of metatibial length.

Aedeagus: Fig. 36E–G. Habitus: Fig. 36H.

### Tetraserica nonglomensis sp. n.

Taxon classificationAnimaliaColeopteraMelolonthidae

http://zoobank.org/EB86CCFF-D0D4-4483-A1A8-DC5514A06F48

Figures 36 , 51

#### Type material examined.

Holotype: ♂ “S LAOS, Attapu, Nong Lom (lake), 18-30.iv.1999 800 m, 15°02N, 106°35'E, E Jendek & O Šauša leg./ Coll. P Pacholátko/ 196 Sericini Asia spec.” (CPPB).

#### Description.

Length of body: 8 mm; length of elytra: 6.3 mm; maximum width: 5 mm. Surface of labroclypeus and disc of frons glabrous. Smooth area anterior to eye twice as wide as long. Eyes moderately large, ratio of diameter/interocular width: 0.56. Ratio of length of metepisternum/metacoxa: 1/1.88. Metatibia short and wide, ratio width/length: 1/2.73; basal group of dorsal spines of metatibia at first third of metatibial length.

Aedeagus: Fig. 36I–K. Habitus: Fig. 36L.

Female unknown.

#### Diagnosis.

*Tetrasericanonglomensis* sp. n. is rather similar to *T.breviforceps* sp. n.; *T.nonglomensis* sp. n. differs by the much longer, robust dorsal lobe of left paramere.

#### Etymology.

The new species is named after the type locality, Nong Lom Lake (adjective in the nominative singular).

### Tetraserica satura

Taxon classificationAnimaliaColeopteraMelolonthidae

(Brenske, 1898) comb. n.

Figures 37 , 47

Neoserica satura Brenske, 1898: 345.
Tetraserica graciliforceps Liu, Fabrizi, Bai, Yang & Ahrens, 2014: 103, fig. 6E–H, syn. n.

#### Type material examined.

Syntypes: 3 ♂♂, 2 ♀♀ “Hte Birmanie Mines des Rubies 1200–2300 m Doherty 1890/ *satura* type Brsk.” (MNHN).

#### Additional material examined.

1 ♂ “N THAILAND, Mae Hong Son Pref, Soppong Pai Dist. Alt 1290 m 20-21.V.1998 leg. K Masumoto/ coll. Dirk Ahrens” (ZFMK).

**Figure 37.**
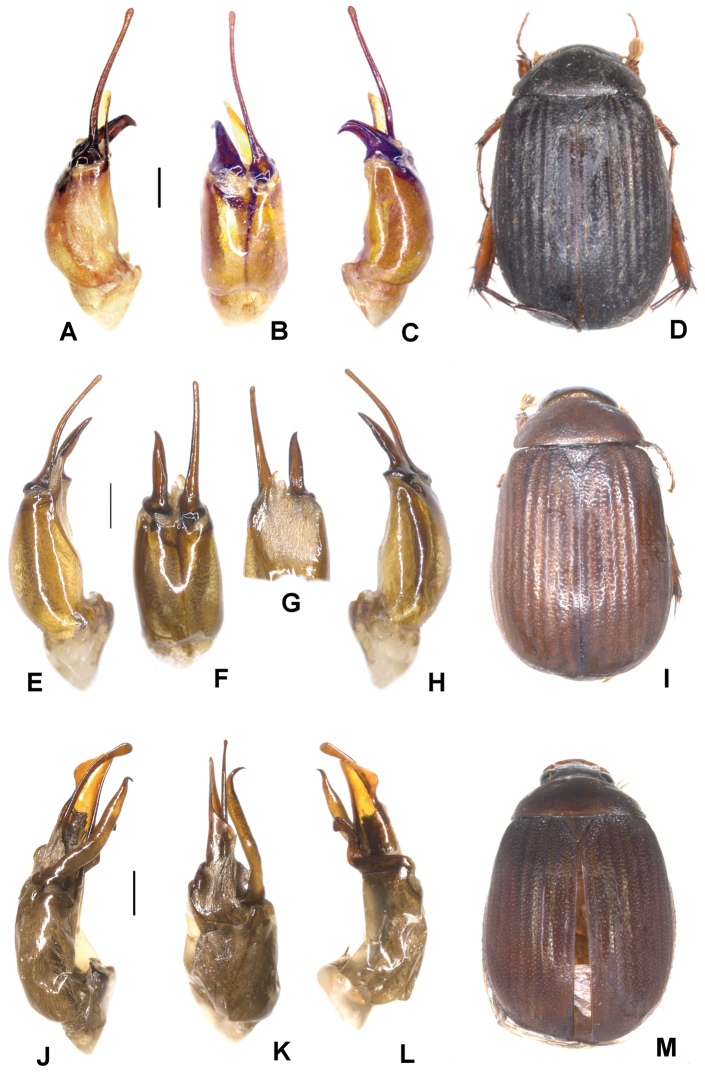
**A–D***Tetrasericasatura* (Brenske, 1898) (holotype of *T.graciliforceps*) **E–I***T.filiforceps* sp. n. (holotype) **J–M***T.spanglerorum* sp. n. (holotype) **A, E, J** aedeagus, left side lateral view **C, H, L** aedeagus, right side lateral view **B, F, K** parameres, dorsal view **G** parameres, ventral view **D, I, M** habitus. Scale bars: 0.5 mm. Habitus not to scale.

Aedeagus: Fig. 37A–C. Habitus: Fig. 37D.

#### Remarks.

The holotype of *Tetrasericagraciliforceps* Liu et al., 2014 is virtually identical with the male syntypes of *Tetrasericasatura* (Brenske, 1898) in shape of parameres; therefore the former is assigned a junior synonym of *T.satura*.

### Tetraserica filiforceps sp. n.

Taxon classificationAnimaliaColeopteraMelolonthidae

http://zoobank.org/22609FB7-9667-4538-A52D-6CBD8703A896

Figures 37 , 48

#### Type material examined.

Holotype: ♂ “NE-Laos: Houa Phan prov., 20°13'09–19"N, 103°59'54"–104°00'03"E, 1480–1510 m, Phou Pan (Mt.) 22.IV.-14.V.2008, Vit Kubáň leg.” (ZFMK). Paratypes: 3 ♂♂ “NE-Laos: Houa Phan prov., 20°13'09–19"N, 103°59'54"–104°00'03"E, 1480–1510 m, Phou Pan (Mt.) 22.IV.- 14.V. 2008, Vit Kubáň leg.” (ZFMK), 2 ♂♂ “NE-Laos: Houa Phan prov., 20°13'09–19"N, 103°59'54"–104°00'03"E, 1480–1510 m, Phou Pan (Mt.) 22.iv.- 14.v. 2008, Vit Kubáň leg.” (ZFMK), 1 ♂ “ Laos-NE: Houa Phan prov., 20°13'09–19"N, 103°59'54"–104°00'03"E, 1480–1510 m, Phou Pan (Mt.), 22.4.-14.5. 2008, Kubáň” (ZFMK), 3 ♂♂ “NE-Laos: Houa Phan prov., 20°13'09–19"N, 103°59'54"–104°00'03"E, Phu Pane Mt., 1480–1510 m, 22.4.- 14.5.2008, Vit Kubáň leg.” (ZFMK), 2 ♂♂ “NE-Laos: Houa Phan prov., 20°13'09–19"N, 103°59'54"–104°00'03"E, Phu Pane Mt., 1480–1510 m, 17.5.-3.6.2007, Vit Kubáň leg.” (ZFMK), 1 ♂ “N-Vietnam (Tonkin) pr. Vinh Phu 1990 Tam Dao 17.-21.V. leg. P. Pacholátko/ coll. P. Pacholátko/ VS81/ 236 Sericini Asia spec.” (CPPB), 1 ♂ “X-DA4581/ X-DA4581 labcode: VD 048 Laos: Houa Phan prov.; Ban Saleui, Phou Pan (Mt.) 20°12'N, 104°01'E, 15.-30.iv.2014 leg. C. Holzschuh - ZFMK Ankauf 2014/ Tetraserica spLA_V21/ sp-LA-V21” (ZFMK), 1 ♂ “ Laos: Houa Phan Prov.; Ban Saleui, Phou Pan (Mt.) - 20°12'N, 104°01'E, 11.iv.-15.v.2012 leg. C. Holzschuh ZFMK Ankauf 2012/13” (ZFMK), 1 ♂ “Sapa 11-18.6. N. Vietnam A. Olexa 1990” (NHMB), 1 ♂ “Vietnam N 1990 SaPa 11-19.VI. 1500 m Hoang Lien Son Prov. Strnad Jan lgt.” (NHMB), 1 ♂ “Vietnam N (Sa Pa) Lao Cai Prov., 250 km from Hanoi bearin 31°, Sa Pa vill. Env. Hoang Lien Son Nat. Res., 27.5.-3.6.1998 1250 m leg. A. Naplolov” (CNAR), 1 ♂ “LAOS-NE, Houa Phan prov., 20°11–13'N, 103°59–104°01'E, Ban Saluei- > Phou Pane Mt., 9.-17.vi.2009, 1300–1900 m, Michael Geiser leg./ NHMB Basel, NMPC Prague, Laos 2009, Expedition: M. Brancucci, M. Geiser, Z. Kraus, D. Hauck, V. Kubáň/ 914 Sericini Asia spec.” (NHMB), 1 ♂ “LAOS-NE, Houa Phan prov., 20°13'09–19"N, 103°59'54"–104°00'03"E, 1480–1550 m, Phou Pane Mt., 1.-16.vi.2009, Zdeněk Kraus leg./ NHMB Basel, NMPC Prague, Laos 2009, Expedition: M. Brancucci, M. Geiser, Z. Kraus, D. Hauck, V. Kubáň/ 914 Sericini Asia spec.” (NHMB), 1 ♂ “LAOS-NE, Xieng Khouang prov., 19°37–8'N, 103°20–1'E, 30 km NE Phonsavan Ban Na Lam -> Phou Sane Mt., 1300–1700 m, 10-30.v.2009, M. Geiser leg./ NHMBBasel, NMPC Prague, Laos 2009, Expedition: M. Brancucci, M. Geiser, Z. Kraus, D. Hauck, V. Kubáň/ 914 Sericini Asia spec.” (NHMB), 2 ♂♂ “Vietnam N (Sa Pa) Lao Cai Prov., 250 km from Hanoi bearin 31°, Sa Pa vill. Env. Hoang Lien Son Nat. Res., 25.VI.-5.VII.1998 1250 m leg. A. Naplolov” (CNAR).

#### Description.

Length of body: 9.1 mm; length of elytra: 6.6 mm; maximum width: 5 mm. Surface of labroclypeus and disc of frons glabrous. Smooth area anterior to eye twice as wide as long. Eyes small, ratio of diameter/interocular width: 0.53. Ratio of length of metepisternum/metacoxa: 1/1.43. Metatibia moderately long and wide, ratio width/length: 1/3.58; basal group of dorsal spines of metatibia at first third of metatibial length.

Aedeagus: Fig. 37E–H. Habitus: Fig. 37I.

Female unknown.

#### Variation.

Length of body: 9.1–9.2 mm; length of elytra: 6.6–7.1 mm; maximum width: 5.0–5.9 mm.

#### Diagnosis.

*Tetrasericafiliforceps* sp. n. differs from the very similar *T.satura* (Brenske, 1898) by the left paramere, which is slightly shorter than phallobase, and the right paramere which is nearly straight (lateral view).

#### Etymology.

The name of the new species (noun in apposition) is derived from the combined Latin words *filum* (string/ filament) and *forceps*, with reference to the filiform parameres.

### Tetraserica spanglerorum sp. n.

Taxon classificationAnimaliaColeopteraMelolonthidae

http://zoobank.org/FFA9116C-99A6-4F03-A859-FA88ADA634F3

Figures 37 , 51

#### Type material examined.

Holotype: ♂ “THAILAND: Nkn. Ratcha. Prov. Nakhon Ratchasima 60 km S. 2-4 Mar 1971, P & P Spangler/ Sakaerat Expt. Sta. 14°30'N, 101°55'E, 300–600 meters/ collected at white light trap/ 910 Sericini Asia spec.” (USNM). Paratypes: 11 ♂♂, 4 ♀♀ “THAILAND: Nkn. Ratcha. Prov. Nakhon Ratchasima 60 km S., 2-4 Mar 1971, P & P Spangler/ Sakaerat Expt. Sta. 14°30'N, 101°55'E, 300–600 meters/ collected at white light trap” (USNM, ZFMK).

#### Description.

Length of body: 7.8 mm; length of elytra: 6.3 mm; maximum width: 5.4 mm. Surface of labroclypeus and disc of frons glabrous. Smooth area anterior to eye twice as wide as long. Eyes small, ratio of diameter/interocular width: 0.54. Ratio of length of metepisternum/metacoxa: 1/1.88. Metatibia short and wide, ratio width/length: 1/3.14; basal group of dorsal spines of metatibia at first third of metatibial length.

Aedeagus: Fig. 37J–L. Habitus: Fig. 37M.

#### Variation.

Length of body: 7.7–7.8 mm; length of elytra: 5.7–6.3 mm; maximum width: 5.2–5.4 mm. Female: Antennal club with three antennomeres, as long as remaining antennomeres combined; eyes as large as in male; pygidium flat.

#### Diagnosis.

The shape of the aedeagus in *Tetrasericaspanglerorum* sp. n. is similar to that of *T.loeiensis* sp. n.; *T.spanglerorum* sp. n. differs by the dorsal lobe of left paramere being distinctly wider than the ventral one and slightly curved (dorsal view); also its right paramere is bent in a nearly sharp angle at the middle.

#### Etymology.

The new species is named after its collectors, P & P Spangler (noun in genitive case, plural).

### Tetraserica shanensis sp. n.

Taxon classificationAnimaliaColeopteraMelolonthidae

http://zoobank.org/A5C021A8-F6CE-4F75-B5BE-BB01403F55B8

Figures 38 , 50

#### Type material examined.

Holotype: ♂ “Burma (Myanmar) SW Shan state Taunggyi J. Rejsek 1.-18.6.1997/ coll. Dirk Ahrens/ 213 Sericini Asia spec.” (ZFMK). Paratypes: 3 ♂♂ “Burma (Myanmar) SW Shan state Taunggyi J. Rejsek 1.-18.6.1997/ coll. Dirk Ahrens” (ZFMK), 2 ♂♂, 6 ♀♀ “Burma (Myanmar) SW Shan state Taunggyi J. Reysek [sic!] 1.-18.6.1997” (ZFMK).

#### Description.

Length of body: 7.1 mm; length of elytra: 4.9 mm; maximum width: 4.1 mm. Body yellowish brown, head darker. Surface of labroclypeus and disc of frons glabrous. Smooth area anterior to eye twice as wide as long. Eyes moderately large, ratio of diameter/interocular width: 0.71. Ratio of length of metepisternum/metacoxa: 1/1.57. Metatibia short and wide, ratio width/length: 1/3.18; basal group of dorsal spines of metatibia at first third of metatibial length.

**Figure 38.**
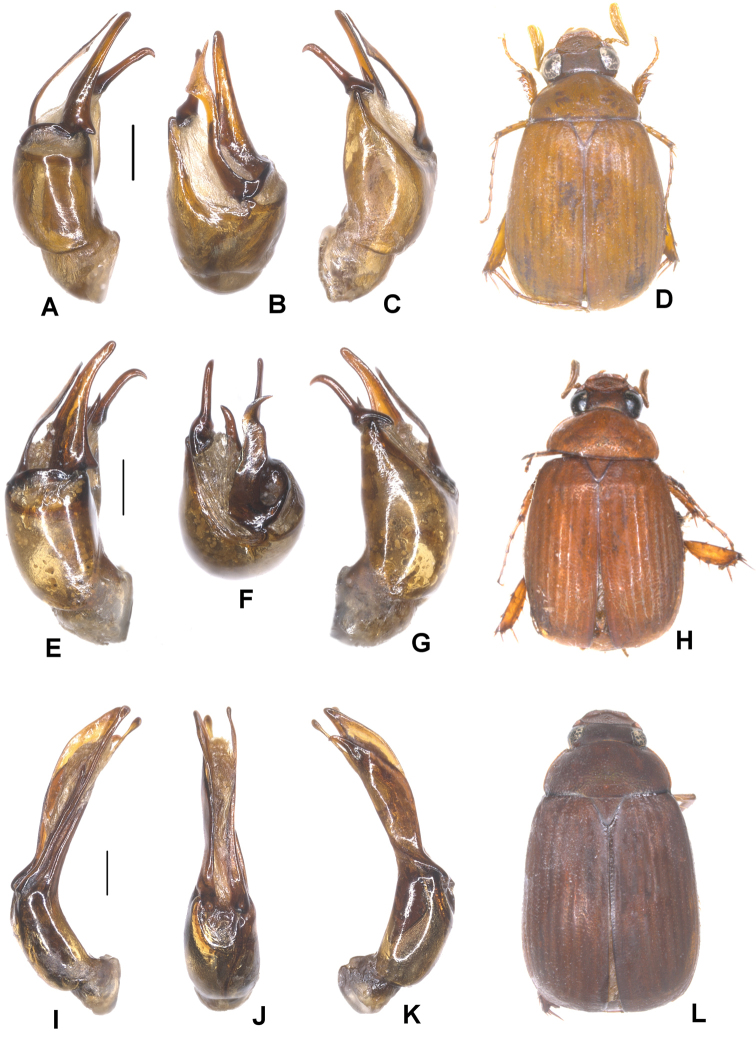
**A–D***Tetrasericashanensis* sp. n. (holotype) **E–H***T.semishanensis* sp. n. (holotype) **I–L***T.sejugata* (Brenske, 1898) (Myanmar, Ruby Mines) **A, E, I** aedeagus, left side lateral view **C, G, K** aedeagus, right side lateral view **B, F, J** parameres, dorsal view **D, H, L** habitus. Scale bars: 0.5 mm. Habitus not to scale.

Aedeagus: Fig. 38A–C. Habitus: Fig. 38D.

#### Variation.

Length of body: 7.1–8.3 mm; length of elytra: 4.9–5.6 mm; maximum width: 4.1–4.4 mm. Female: Antennal club with three antennomeres, as long as remaining antennomeres combined; eyes as large as in male; pygidium weakly convex at apex.

#### Diagnosis.

*Tetrasericashanensis* sp. n. differs from all other *Tetraserica* species in having evenly long spines on ventral margin of metatibia, and in the colour of the dorsal surface being yellowish brown.

#### Etymology.

The new species is named with reference to its occurrence in the Shan state (adjective in the nominative singular).

### Tetraserica semishanensis sp. n.

Taxon classificationAnimaliaColeopteraMelolonthidae

http://zoobank.org/EA1B7576-3D62-4630-BBEA-4C996654FC9F

Figures 38 , 55

#### Type material examined.

Holotype: ♂ “Birmania, S. S. S. Palaing, 1450 m. VI-1936 R. Perego/ MSN Milano” (MSNM). Paratypes: 1 ♂ “THAI 1-8.V.1993 SOPPONG PAI 1800 m Pacholátko & Dembický leg./ Coll. Pacholátko” (CPPB), 1 ♂ “THAI 1-8.V.1993 SOPPONG PAI 1800 m Pacholátko & Dembický leg./ Coll. Pacholátko/ 475 Sericini Asia spec.” (CPPB), 9 ♂♂ “NW Thailand, 19.19N, 97.59E, Mae Hong Son, 1991, Ban Huai Po, 1600–2000 m 17.-23.5. L. Dembický leg./ NHM Wien” (NHMW, ZFMK), 1 ♂, 1 ♀ “NW Thailand, 9.-16.51991 Mae Hong Son Ban Huai Po, 1600–2000 m J. Horak leg.” (ZFMK), 1 ♂ “Thailand bor. Hang Dong 6.1990. lgt. Wimmer” (ZFMK), 2 ♂♂, 3 ♀♀ “Thailand centr. Pa-Sak Fl. –IX.80 Rhetchabun- 600 m.” (ZFMK).

#### Description.

Length of body: 7.6 mm; length of elytra: 5.1 mm; maximum width: 4.4 mm. Body yellowish brown, head darker. Surface of labroclypeus and disc of frons glabrous. Smooth area anterior to eye twice as wide as long. Eyes moderately large, ratio of diameter/interocular width: 0.68. Ratio of length of metepisternum/metacoxa: 1/1.59. Metatibia short and wide, ratio width/length: 1/3.09; basal group of dorsal spines of metatibia at first third of metatibial length.

Aedeagus: Fig. 38E–G. Habitus: Fig. 38H.

#### Variation.

Length of body: 7.1–7.6 mm; length of elytra: 5.0–5.1 mm; maximum width: 4.1–4.4 mm. Female: Antennal club with three antennomeres, as long as remaining antennomeres combined; eyes as large as in male; pygidium weakly convex at apex.

#### Diagnosis.

*Tetrasericasemishanensis* sp. n. differs from the similar *T.shanensis* sp. n. by the narrower, nearly straight ventral lobe of left paramere (lateral view) and the dorsal lobe of left paramere being nearly evenly narrowed towards apex (rather than bluntly widened before apex, as in *T.shanensis*).

#### Etymology.

The species name (adjective in the nominative singular) is derived from the combined Latin words *semi*- (half) and the species name *shanensis*, with reference to the similarity to *T.shanensis* sp. n.

### Tetraserica sejugata

Taxon classificationAnimaliaColeopteraMelolonthidae

(Brenske, 1898) comb. n.

Figures 38 , 50

Serica sejugata Brenske, 1898: 323.

#### Type material examined.

Syntype: 1 ♂ “H.^te^ Birmanie Mines des Rubis 1200^_^2300 m Doherty 1890/ ♂/ E. brenske 1896/ *sejugata* Type Brsk./ Museum Paris ex Coll. R. Oberthur/ Type” (MNHN).

#### Additional material examined.

1 ♂ “H.^te^ Birmanie Mines des Rubis 1200^_^2300 m Doherty 1890/ Museum Paris ex Coll. R. Oberthur/ 120 Sericini Asia spec.” (MNHN), 1 ♂ “H.^te^ Birmanie Mines des Rubis 1200^_^2300 m Doherty 1890/ Museum Paris ex Coll. R. Oberthur” (MNHN), 1 ♂ “Birmah Rubym^se^ / Doherty/ Fry Coll. 1905-100” (NHMUK).

#### Redescription.

Length of body: 8.4 mm; length of elytra: 6.2 mm; maximum width: 4.9 mm. Surface of labroclypeus and disc of frons glabrous. Smooth area anterior to eye twice as wide as long. Eyes small, ratio of diameter/interocular width: 0.59. Ratio of length of metepisternum/metacoxa: 1/ 1.44. Metatibia long and moderately wide, ratio width/length: 1/ 3.54; basal group of dorsal spines of metatibia at first third of metatibial length.

Aedeagus: Fig. 38I–K. Habitus: Fig. 38L.

### Tetraserica allosejugata sp. n.

Taxon classificationAnimaliaColeopteraMelolonthidae

http://zoobank.org/56A2BFB2-B35E-4FDA-BD7F-1116ED912AE9

Figures 39 , 46

#### Type material examined.

Holotype: ♂ “China: S-Yunnan (Xishuangbanna) 27 km NW Jinghong vic. Beng Gang Ha Ni / N22 08.74; E 100 35.50, 1800–2000 m 29.V.2008 leg. A. Weigel KL/HF” (NME). Paratypes: 6 ♂♂ “China: S-Yunnan (Xishuangbanna) 27 km NW Jinghong vic. Beng Gang Ha Ni / N22 08.74; E 100 35.50, 1800–2000 m 29.V.2008 leg. A. Weigel KL/HF” (NME, ZFMK), 1 ♂ “Thai, Chiang Mai prov., 18°49'N, 98°54'E, 1600 m, DOI PUI mt., 2.-6.v., Vít Kubáň leg. 1996/ coll. P. Pacholátko/ 165 Sericini Asia spec.” (CPPB), 1 ♂ “Thai-N Chiang Mai prov., 18°49'N, 98°54'E, 1600 m, DOI PUI mt., 2.-6.v., Vít Kubáň leg. 1996/ coll. P. Pacholátko/ 136 Sericini Asia spec.” (CPPB), 1 ♂ “THAI 1-8.V.1993 SOPPONG PAI 1800 m Pacholátko & Dembický leg./ Coll. P. Pacholátko” (CPPB), 1 ♂ “N-Thailand V.1990 Doi Inthanon lg. Malicky” (ZSM), 1 ♂ “N-Thailand 25.-29.5.1990 Doi Inthanon leg. Malicky” (ZSM), 1 ♂ “839475/ 839475 Tetraserica sp THAI_DE09_2 Thailand L. Dembicky 23.-30.4.2009 Pha Pok Mt. Chiang Mai Prov. 20°02'36"N, 99°08'45"E, 1900–2000 m” (ZFMK), 1 ♂ “839484/ 839484 Tetraserica sp THAIL_DE09_2 Thailand L. Dembicky 2.-7.5.2009 Ang Khang region Chiang Mai Prov. 19°53'45’’ 99°02'45"E, 1600 m” (ZFMK), 1 ♂ “839475/ 839475 Tetraserica spTHAI_DE09_2 Thailand L. Dembicky 23.-30.4.2009 Pha Pok Mt. Chiang Mai Prov. 20°02'36"N, 99°08'45"E, 1900–2000 m” (ZFMK).

#### Description.

Length of body: 7.6 mm; length of elytra: 6.1 mm; maximum width: 4.4 mm. Surface of labroclypeus and disc of frons glabrous. Smooth area anterior to eye twice as wide as long. Eyes small, ratio of diameter/interocular width: 0.52. Ratio of length of metepisternum/metacoxa: 1/1, 81. Metatibia moderately long and wide, ratio width/length: 1/3.91; basal group of dorsal spines of metatibia at first third of metatibial length.

**Figure 39.**
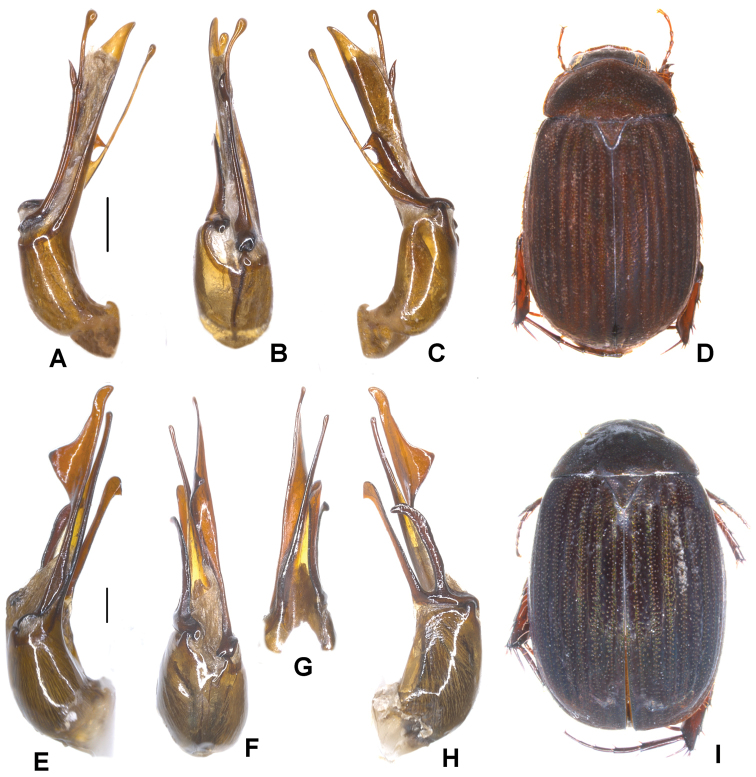
**A–D***Tetrasericaallosejugata* sp. n. (holotype) **E–I***T.veliformis* sp. n. (holotype) **A, E** aedeagus, left side lateral view **C, H** aedeagus, right side lateral view **B, F** parameres, dorsal view **G** parameres, ventral view **D, I** habitus. Scale bars: 0.5 mm. Habitus not to scale.

Aedeagus: Fig. 39A–C. Habitus: Fig. 39D.

Female unknown.

#### Variation.

Length of body: 7.6–8.1 mm; length of elytra: 6.0–6.1 mm; maximum width: 4.4–4.8 mm.

#### Diagnosis.

*Tetrasericaallosejugata* sp. n. differs from the very similar *T.sejugata* (Brenske, 1898) by the right paramere being split at the basal third into two narrow branches, while in *T.sejugata* this split is situated in the apical quarter.

#### Etymology.

The species name (adjective in the nominative singular) is derived from the combined Greek prefix *allo*- (nearly) and the species name *sejugata*, with reference to the similarity to *T.sejugata* (Brenske).

### Tetraserica veliformis sp. n.

Taxon classificationAnimaliaColeopteraMelolonthidae

http://zoobank.org/16DD8EDD-F0DD-4364-AEFC-790F65219E89

Figures 39 , 53

#### Type material examined.

Holotype: ♂ “W Thailand, Sai Yok, Kanchanaburi, Srinakarinda; 300m; 25.-27.IV.1996; leg. S. Ohmomo” (ZFMK). Paratypes: 11 ♂♂ “W Thailand, Sai Yok, Kanchanaburi, Srinakarinda; 300 m; 25.-27.IV.1996; leg. S. Ohmomo” (ZFMK), 9 ♂♂ “W-Thailand: Pu Nam Long, Hot Spring, 100 km NW of Kanchaburi; 06.-8.V.1993 leg. T. Itoh “ (ZFMK), 1 ♂ “Thailand 3.-7.IV.1991 Kanchanaburi 150 m 14°02'N, 99°31'E, Vit Kuban leg.” (ZFMK), 2 ♂♂ “Laos, Salavan prov., ca.16 Km NW Salavan (at light) boroken bridfge over SE DON river 15°47.4'N, 106°17.5'E, 150 m, Jiri Hájek leg. 15.V.2010” (NMPC, ZFMK), 1 ♂ “THAI 26.IV.-6.V.1991 UMPHANG 500m 16°04'N, 98°53'E, Vít Kubáň leg./ Thailand 91 “Thanon Tong Cha” D. Král & V. Kubáň/ coll. P. Pacholátko” (CPPB), 1 ♂ “THAI, 9.-13.IV.1991 THIMONGHTA 350 m 15 02'N, 98 35'E, P. Pacholátko leg./ coll. P. Pacholátko/ 149 Sericini Asia spec.” (ZFMK), 2 ♂♂ “Coll. I. R. Sc. N. B., Thailand, Kanchanaburi prov., Sai Yok N.P. 4-5.VI.2003 Leg. J. Constant & K. Smets” (ISNB, ZFMK), 2 ♂♂, 3 ♀♀ “W-Thailand: Khao Laem Dam, 15°10'N, 99°00'E; 130 km NNW of Kanchaburi; 7.V.1993 alt. Ca. 300 m; T. Itoh leg.” (ZFMK), 1 ♂ “Thailand occ. 08.-12.04.1991 Sangkhlaburi Jan Farkac leg.” (NHMB).

#### Description.

Length of body: 9.6 mm; length of elytra: 7.6 mm; maximum width: 6 mm. Surface of labroclypeus and disc of frons glabrous. Smooth area anterior to eye twice as wide as long. Eyes small, ratio of diameter/interocular width: 0.48. Ratio of length of metepisternum/metacoxa: 1/1.79. Metatibia short and wide, ratio width/length: 1/2.95; basal group of dorsal spines of metatibia at first third of metatibial length.

Aedeagus: Fig. 39E–H. Habitus: Fig. 39I.

#### Variation.

Length of body: 8.8–10.1 mm; length of elytra: 6.6–7.7 mm; maximum width: 5.9–6.1 mm. Female: Eyes as large as in male; antennal club composed of three antennomeres, club as long as remaining antennomeres combined; pygidium flat.

#### Diagnosis.

*Tetrasericaveliformis* sp. n. differs from all other *Tetraserica* species in having a straight or convex posterior margin of metafemur and by the left paramere having a large triangular dorsomedian extension.

#### Etymology.

The name of the new species (noun in apposition) is derived from the combined Latin words *velum* (sail) and *formis* (in shape), with reference to the large triangle-shaped tooth of the left paramere.

### Tetraserica latefemorata

Taxon classificationAnimaliaColeopteraMelolonthidae

Kobayashi, 2017

Figures 40 , 49

Tetraserica latefemorata Kobayashi, 2017: 35, figs 2, 11.

#### Material examined.

2 ♂♂ “THAI: SOPPONG 1-9.5.2000 MORAVEC PETER/ 923 Sericini Asia spec.” (ZFMK), 1 ♂ “Thailand Pai env. 1.5.-14.5.2001 J. Hromádka lgt.” (ZFMK).

#### Redescription.

Length of body: 9.6 mm; length of elytra: 7.1 mm; maximum width: 6.4 mm. Surface of labroclypeus and disc of frons glabrous. Smooth area anterior to eye twice as wide as long. Eyes small, ratio of diameter/interocular width: 0.47. Ratio of length of metepisternum/metacoxa: 1/1.77. Metatibia short and wide, ratio width/length: 1/2.45; basal group of dorsal spines of metatibia at first third of metatibial length.

**Figure 40.**
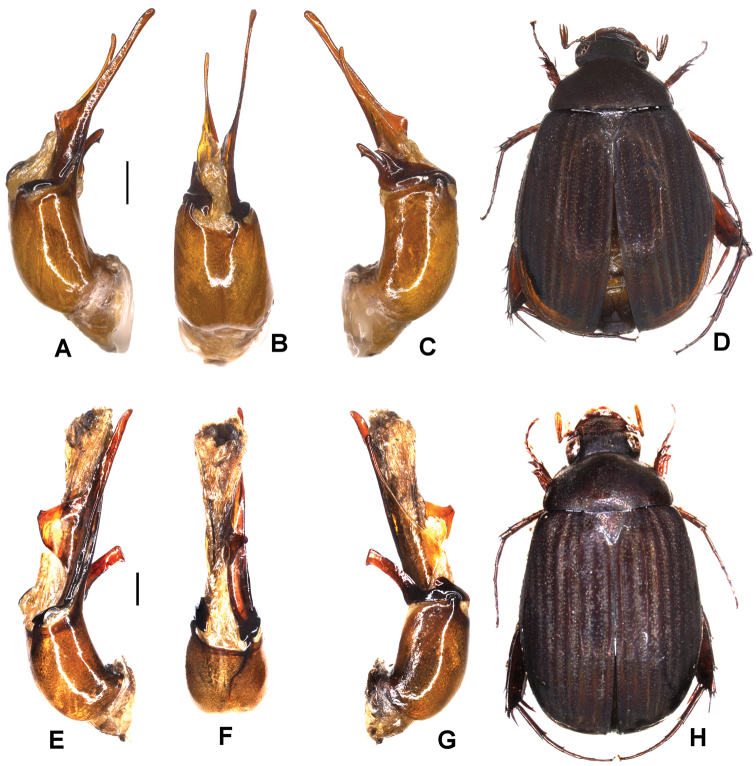
**A–D***Tetrasericalatefemorata* Kobayashi, 2017 (Thailand, Soppong) **E–H***T.umphangensis* sp. n. (holotype) **A, E** aedeagus, left side lateral view **C, G** aedeagus, right side lateral view **B, F** parameres, dorsal view **D, H** habitus. Scale bars: 0.5 mm. Habitus not to scale.

Aedeagus: Fig. 40A–C. Habitus: Fig. 40D.

### Tetraserica umphangensis sp. n.

Taxon classificationAnimaliaColeopteraMelolonthidae

http://zoobank.org/3DE9C1ED-0561-4DB9-9E09-25DB95D3FE85

Figures 40 , 51

#### Type material examined.

Holotype: ♂ “Thai 28/4 - 6/5.91 Umphang river 1000 m 16°07'N, 98°00'E, David Kral lgt./ 1015 Asia Sericini spec.” (NMPC). Paratype: 1 ♂ “Thai 26/4 - 6/5.91 Umphang 500 m 16°04'N, 98°53'E, David Kral lgt.” (ZFMK).

#### Description.

Length of body: 8.6 mm; length of elytra: 6.6 mm; maximum width: 5.3 mm. Surface of labroclypeus and disc of frons glabrous. Smooth area anterior to eye twice as wide as long. Eyes moderately large, ratio of diameter/interocular width: 0.57. Ratio of length of metepisternum/metacoxa: 1/1.78. Metatibia short and wide, ratio width/length: 1/3.2; basal group of dorsal spines of metatibia at first third of metatibial length.

Aedeagus: Fig. 40E–G. Habitus: Fig. 40H.

Female unknown.

#### Variation.

Length of body: 8.6–10.0 mm; length of elytra: 6.6–7.2 mm; maximum width: 5.3–5.8 mm.

#### Diagnosis.

*Tetrasericaumphangensis* sp. n. is rather similar to *T.veliformis* sp. n.; *T.umphangensis* sp. n. differs by the right paramere being only half as long as left paramere, and the dorsal lobe of the right paramere, which is distinctly shorter than the ventral lobe. In *T.veliformis* the right paramere is slightly more than half as long as left paramere, and the split between lobes of the right paramere is deep, the dorsal lobe is as long as the ventral lobe.

#### Etymology.

The new species is named after the type locality, Umphang River (adjective in the nominative singular).

### Tetraserica pingjiangensis

Taxon classificationAnimaliaColeopteraMelolonthidae

Liu, Fabrizi, Bai, Yang & Ahrens, 2014

Figures 41 , 53

Tetraserica pingjiangensis Liu, Fabrizi, Bai, Yang & Ahrens, 2014: 105, fig. 6I–L.

#### Material examined.

**China**: 1 ex. “China: S-Yunnan (Xishuangbanna) 20 km NW Jinghong vic. Man Dian (NNNR) / N22°07.80 E100°40.05 730 m 23.V.2008 MF leg. A. Weigel forest” (NME), 1 ex. “China: S-Yunnan (Xishuangbanna) 20 km NW Jinghong vic. Man Dian (NNNR) / N22°07.80 E100°40.05 730 m 13.V.2008 MF leg. A. Weigel forest” (NME), 2 ex. “China: S-Yunnan (Xishuangbanna) 37 km NW Jinghong Guo Men Shan (NNNR)/ N22°17.91 E100°38.85 1080 m 26.V.2008 leg. A. Weigel LF” (NME), 2 ex. “China: S-Yunnan (Xishuangbanna) 20 km NW Jinghong, Man Dian NNNR office / N22°07.80 E100°40.05 740 m LFF, 24.V.2008 leg. A. Weigel” (NME), 1 ex. “China: S-Yunnan (Xishuangbanna) 23 km NW Jinghong, vic. Na Ban (NNNR)/ N22°09.49 E100°39.92 730 m 12.V.2008 traps site leg. A. Weigel” (NME), 1 ex. “China: S-Yunnan (Xishuangbanna) 23 km NW Jinghong, (NNNR) Na Ban village 600 m/ N22°10.04 E100°39.52 17.V.2008 leg. A. Weigel” (NME). **Vietnam**: 2 ex. “Vietnam N. Tonkin Cuc-Phong Nat. Park 2.-12.V.1991 E. Jendek leg.” (ZFMK), 1 ex. “Vietnam, N. Ninh Binh Pr., 90 km SW Hanoi Cuc Phong NP, primate rescue centre, 25.iv./ 2012, 190 m, 20°14'24"N, 105°42'53’’, leg. A. Weigel, light trap” (NME), 1 ♂ “Coll. I.R.Sc.N.B. Vietnam, Cuc Phuong N.P. 20°19'00"N, 105°36'30"E, 19-23.vii.2011, light trap Leg. J. Constant & J. Bressel I.G.31.933” (ISNB), 62 ex. “N-Vietnam Cao Bang Prov., vic. Vin Den, Nui Pia Oac Nat. Res. 06.-10.V.2013, 22°33'53"N, 105°52'53"E, 900–1300 m A. Skale” (CASH), 3 ♂♂, 8 ♀♀ ex. “N-Vietnam Cao Bang Prov., vic. Vin Don, Nui Pia Oac Nat. Res. 22°23'52"N, 105°52'53"E, 15.V.2014, 900–1350 m leg. A. Skale” (NME), 1 ♂ “N-Vietnam Cao Bang Prov., vic Tinh Tuc, Son Dong, Nui Pia Oac Nature Reserve, 9.-15.V.2014 22°37'55"N, 105°52'98"E, 850–1300 m leg. A. Skale” (NME), 2 ex. “N-Vietnam Thai Nguyen Prov., vic. Ngoc Thanh, Me Linh (IEBR station), 12.V.2012, 21°23'3"N, 105°42'44"E, 60–80 m, leg. A. Skale” (CASH), 17 ex. “N-Vietnam Bac Kan Prov., Ba Bè NP. (entry), 16.-20.V.2014 22°25'07"N, 105°38'09"E, 180–220 m, leg. A. Skale” (CASH), 9 ex. “N. Vietnam Bac Kan Pr., Ba Be NP (entry) 16.-20.V.2014, N22°25'07’’, 105°38'09’’, 180–200 m, leg. A. Weigel, by light” (NME), 1 ex. “Coll.I.R.Sc.N.B. Vietnam-Cuc Phong 11-18-VIII-2010/ light trap I.G. 31668 Leg. J. Constant & P. Limbourg” (ISNB), 3 ex. “N. Vietnam 26-27.V.1989 Hoa Binh Brentlova lgt.” (NHMB), 1 ex. “Vietnam N 1990 Sa-Pa 11-19.VI. 1500 m Hoang Lien Son prov. Strnad Jan lgt.” (NHMB), 1 ex. “Hanoi Tonkin” (MNHN), 8 ex. “Tonkin occ. Rég. de Hoa-Binh 1919” (MNHN), 4 ex. “Tonkin occ. Rég. de Hoa-Binh R.P.A. de Cooman 1919” (MNHN), 1 ex. “N-Vietnam, Ninh Binh Prov., Cuc Phong NP, N20°17.572’ E105°40.052’, 270 m, 22.5.-24.5.2015, leg. A. Skale” (CASH), 1 ♂ “Coll. I.R.Sc.N.B. Vietnam-Cuc Phong 11-18-VIII-2010/ Light Trap I.G. 31.668 Leg. J. Contsant & P. Limbourg” (ISNB), 2 ♂♂ “N. Vietnam 26-27.V.1989 Hoa Binh Brantlová lgt.” (NHMB), 1 ♂ “Vietnam N (Sa Pa) Lao Cai Prov., 250 km from Hanoi bearin 31°, Sa Pa vill. Env. Hoang Lien Son Nat. Res., 27.5.-3.6.1998 1250 m leg. A. Napolov” (CNAR), 1 ♂ “Vietnam N (Sa Pa) Lao Cai Prov., 250 km from Hanoi bearin 31°, Sa Pa vill. Env. Hoang Lien Son Nat. Res., 21.-23.6.1998 1250 m leg. A. Naplolov” (CNAR), 1 ♂ “Vietnam-N (Na Hang) 160 km NW Ha Noi, NE env. of Na Hang 26.5-6.6.1996 150–200 m lg. A. Napolov & I. Roma/ CNa Riga” (CNAR), 5 ♂♂ “N-Vietnam Cao Bang Prov. Vic Vin Den, Nui Pia Qac Nature Reserve N22°33'53’’, E105°52'53’’ 09.V.2013, 900–1300 m, leg. A: Weigel” (NME), 4 ♂♂ “Vietnam – Ninh Binh Prov. Cuc Phuong Natl. Park (200 m) 20°15'01"N, 105°42'31"E, 3-5.V.2014, at light/ legit L. Bartolozzi, G. Chelazzi, A. Bandinelli, S. Bambi, F. Fabiano /n° Magazz. 2978)” (MZUF), 17 ♂♂ “N-Vietnam Bac Kan province, Ba Be National Park (350m) (at light) 3-8.VI.2011/ L. Bartolozzi, S. Bambi, F. Fabiano, E. Orbach leg. (Num. Magazzino 2909)” (MZUF), 2 ♂♂ “N Vietnam- Lao Cai province, van ban district: Van Ban Nature Reserve (at light) (1000 m) 23.-26.V.2011/ L. Bartolozzi, S. Bambi, F. Fabiano, E. Orbach leg. (Num. Magazzino 2909)” (MZUF), 1 ♂ “N Vietnam: Tuyen Quang Prov., Na Hang Nature Reserve (150 m) 5-7/VI/2013 at light/ legit L. Bartolozzi, S. Bambi, F. Ciariferoni, G. Mazza, E. Orbach (n° Mag. 2950)” (MZUF), 1 ♂ “N Vietnam Hoa Binh Prov., Pa Co Hang Kia Nature Reserve (700m), 9-12/VI/2013 at light/ legit L. Bartolozzi, S. Bambi, F. Ciariferoni, G. Mazza, E. Orbach (n° Mag. 2950)” (MZUF), 3 ♂♂ “N-Vietnam Cao Bang Prov. vic. Vin Den, Nui Pia Oac Nature Reserve, 6.-10.V.2013, 22°33'53"N, 105°52'53"E, 900–1300 m A. Skale” (ZFMK), 1 ♀ “X-DA3987 labcode: VD019 VIETNAM Cao Bang Prov., vic. Vin Den, Nui Pia Oac Nat. Res. 1300 m 22°33'53"N, 105°52'53"E, 6.-10.V.2013, A. Skale” (ZFMK), 1 ♀ “X-DA3988 labcode: VD020 VIETNAM Cao Bang Prov., vic. Vin Den, Nui Pia Oac Nat. Res. 1300m 22°33'53"N, 105°52'53"E, 6.-10.V.2013, A. Skale” (ZFMK), 4 ♂♂ “N-Vietnam Bac Kan Prov., Ba Be NP., (entry), 18.-20.V.2014 22°25'07"N, 105°38'09"E, 180–220 m, leg. A. Skale” (ZFMK), 11 ♂♂ “Vietnam-N Cao Bang prov., 12 km NE Cao Bang, 650±50 m 22°45'45"N, 106°19'E, L. Dembicky leg., 15.-16.V.2010” (ZFMK), 1 ♀ “X-DA3435 labcode VD014 Vietnam N.: Hanoi Prov., Ba Vi National Park (at light) 21-24.vi.2012 L. Bartolozzi, S. Bambi, F. Fabiano, E. Orbach Tetraserica spVi1” (MZUF), 1 ♀ “X-DA3439 labcode VD017 Vietnam N.: Hanoi Prov., Ba Vi National Park (at light) 21-24.vi.2012 L. Bartolozzi, S. Bambi, F. Fabiano, E. Orbach Tetraserica spVi1” (MZUF), 1 ♂ “X-DA4523/ X-DA4523 labcode: VD037 Laos NW h=750 m 5 km SE Muang Sing Chiang Tung (Stupa) GH 9-14.VI.2010 S. Murzin Tetraserica pinjiangensis” (ZFMK), 1 ♂ “X-DA4530/ X-DA4530 labcode: VD039 Laos NW h=750 m 5 km SE Muang Sing Chiang Tung (Stupa) GH 9-14.VI.2010 S. Murzin Tetraserica pinjiangensis” (ZFMK), 1 ♂ “X-DA2491a/ X- DA2491a labcode: VD011 Thailand Chiang Dao Hill resort (100 km N of Chiang Mai) 600 m 28.-31.5.2009 S. Murzin Tetraserica ThaiSpMU09_1” (ZFMK), 1 ♂ “X-DA2491b/ X- DA2491b labcode: VD011 Thailand Chiang Dao Hill resort (100 km N of Chiang Mai) 600 m 28.-31.5.2009 S. Murzin Tetraserica ThaiSpMU09_1” (ZFMK), 1 ♂ “X-DA3436/ X-DA3436 labcode VD015 Vietnam N.: Hanoi Prov., Ba Vi National Park (at light) 21-24.vi.2012 L. Bartolozzi, S. Bambi, F. Fabiano, E. Orbach Tetrasericapingjiangensis” (MZUF), 1 ♂ “X-DA3437/ X-DA3437 labcode VD016 Vietnam N.: Hanoi Prov., Ba Vi National Park (at light) 21-24.vi.2012 L. Bartolozzi, S. Bambi, F. Fabiano, E. Orbach Tetrasericapingjiangensis” (MZUF), 1 ♂ “X-DA3985/ X-DA3985 labcode VD018 Vietnam Cao Bang Prov., vic. Vin Den, Nui Pia Oac Nat. Res. 900–1300 m 22°33'53"N, 105°52'53"E, 6.-10.V.2013, A. Skale” (ZFMK). **Thailand**: 3 ♂♂ “Thailand, China Rai Doi Thung mountain 900 m 30-31.5.1984 Matti Hämälainen leg.” (RMNH), 1 ♂ “NW Thailand, 1991 Chom Thong, 24.-27.4. 19.26N, 98.41E L. Dembicky leg” (NHMW), 4 ♂♂ “Thailand occ. bor., 24.-28.04.1991 Chom Thong, J. Farkac leg.” (NHMB), 8 ♂♂ “NE Thailand, 23-27.4.1991 Chom Thong S. Bily leg.” (NHMB), 1 ♂ “THAI LAND: CHIANG DAO 10-15.5.2000 MORAVEC PETER “ (ZFMK), 1 ♂ “Coll. I. R. Sc. N. B., THAILAND (Loei), Na-Haeo (field res stat) 15-19.V.2003 Light trap Leg. J. Constant, K. Smets & P. Grootaert” (ISNB), 1 ♂ “Coll. I. R. Sc. N. B., THAILAND (Loei), Na-Haeo (Field Res. Stat.) 15-19.V.2003 Light trap Leg. J. Constant & K. Smets” (ISNB), 1 ♂ “Coll. I. R. Sc. N. B., THAILAND (Loei), Na Haeo Forest clearing 16.V.2003 Light Trap Leg. J. Constant & K. Smets “ (ISNB), 2 ♂♂ “Coll. I. R. Sc. N. B., THAILAND (Loei), Na-Haeo (edge pond) Light trap 17.V.2003 Leg. J. Constant & K. Smets” (ISNB), 7 ex. “NE Thailand, 23-27.4.1991 Chom Thong S. Bily leg.” (NHMB), 1 ex. “NW Thailand, 1991 Chom Thong, 24.-27.4. 18.26 N 98.41 E L. Dembicky leg.” (NHMW), 1 ex. “Thailand, Loei Prov., 15 km E Phu Ruea, h=735 m, 17°27'10, 2"N, 101°29'25, 7"E, 27.05.2010 light V.K. Zinchenko leg.” (ISEA), 6 ♂♂ “Thailand 9.-14.V.1991 Chiang Dao 350 m 19°22'N, 98°57'E, V. Kuban lg./ coll. Milan Nikodym, Praha” (ZFMK), 1 ♂ “Thailand 10.-16.V.1991 Chiang Dao 600 m 19°24'N, 98°55'E, V. Kuban lg./ coll. Milan Nikodym, Praha” (ZFMK), 5 ♂♂ “Thailand 26.-28.V.1991 Palong 750 m 19°55'N, 99°06'E, Vit Kuban lgt./ coll. Milan Nikodym, Praha” (ZFMK), 4 ex. “Thai 9-14.5.1991 Chiang Dao 350 m 19°22'N, 98°57'E, D. Kral lgt.” (NMPC), 1 ♂ “Thailand: Loei Province Phu Rua National Park 9 June 1998; L 173 Sites, Simpson, Vitheepradit black light at Park HQ/ University of Missouri” (UMRM). **Laos**: 1 ♂ “ LAOS, Vientiane Municipal, Nam Ngum river, Lao Pako 50 Km NW Vientiane, 1-5.5.2005, M. Pejcha/ 900 Sericini Asia spec.” (NME), 1 ♂ “THAILAND, CHAIYAPHUM Phu Khieo Wildlife Sanctuary 9-11.6.1984 M. Hämäiäinen leg./ MUSEUM LEIDEN Ex, Coll. M. Hämäiäinen acq. 2002” (RMNH), 1 ex. “N-Laos: Prov. Lg. Nam Tha ca. 10km E Lg. Nam Tha 19.6.1996 600 m leg. Schillhammer (32)” (NHMW), 1 ex. “N. Laos: Prov. Lg Nam Tha Muang Sing, at light 9.-13.6.1996, 600 m leg. Schillhammer (18)” (NHMW), 1 ♂ “N-Laos: Prov. Nam Tha ca. 10 km E Lg. Nam Tha 19.6.1996, 600 m leg. Schillhammer (32)” (NHMW), 1 ♂ “Burma: S. Shan States Keng Teng States 2000 feet 11.V.1931 Kingdon Ward B.M. 1938-274” (NHMUK), 4 ♂♂ “Hoa-Bin, Tonkin. A da Cooman. B.M.1934-95” (NHMUK), 1 ♂ “Tonkin Haobinh. A. de Cooman B.M. 1940-13.” (NHMUK), 1 ♂ “Haut Mekong Nam Tha 9.IV.1918 R.V. de Salvazza.” (NHMUK), 1 ♂ “Haut Mekong Houei Sai 6-10.VI.1918 R.V. de Salvazza.” (NHMUK), 1 ♂ “Louang Prabang Ban Sam Mo 3.IV.1918 R.V. de Salvazza.” (NHMUK), 1 ♂ “Tonkin Hoa Binh Aug. 1918 R.V. de Salvazza.” (NHMUK), 1 ♂ “Thailand: Loei Province Phu Rua Natinoal Park 10 June 1998: L-174 Sites, Simpson, Vitheepradit vegetation at Park HQ” (UMRM), 1 ♂ “Thailand Chiang Rai VI.2002 Machytka” (ZFMK), 3 ♂♂ “Tonkin Region de Hoa Binh/ Museum Paris 1934 A. de Cooman” (MNHN), 3 ♂♂ “Tonkin occ. Reg. de Hoa Binh 1919/ Muséum Paris 1952 Coll. R. Oberthur” (MNHN), 1 ♂ “Museum Paris Tonkin Langue 1985/ Omaloplia” (MNHN), 1 ♂ “Ban Angkhai alt. 750 m Samoeng Distr. Chiangmai pref. Thailand 15-20-V-1998 K. Masumoto leg.” (ZFMK), 1 ♂ “Chiang Mai N. Thailand 1985/ Ex coll. Takeshi Matsumoto (formerly Itoh) via Coll. D. Ahrens” (ZFMK).

**Figure 41.**
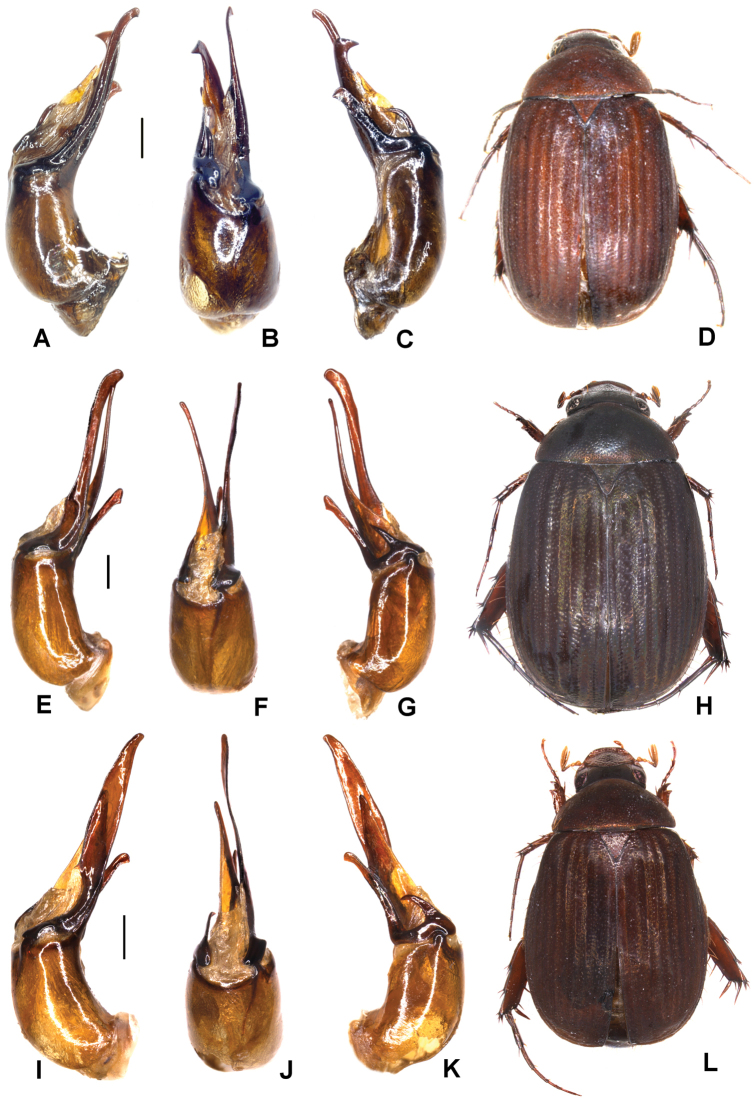
**A–D***Tetrasericapingjiangensis* Liu et al., 2014 (holotype) **E–H***T.bolavensensis* sp. n. (holotype) **I–L***T.nakaiensis* sp. n. (holotype) **A, E, I** aedeagus, left side lateral view **C, G, K** aedeagus, right side lateral view **B, F, J** parameres, dorsal view **D, H, L** habitus. Scale bars: 0.5 mm. Habitus not to scale.

Aedeagus: Fig. 41A–C. Habitus: Fig. 41D.

### Tetraserica bolavensensis sp. n.

Taxon classificationAnimaliaColeopteraMelolonthidae

http://zoobank.org/E47115B9-655E-48B1-A314-F61E717D9873

Figures 41 , 46

#### Type material examined.

Holotype: ♂ “Laos, Attapeu prov. Bolavens Plateau, bridge ca. 4 km E TAD KATAMTOK (at light) 15°07.8'N, 106°40.1'E, 260 m Jiři Hájek leg. 11-12.v.2010/ 929 Sericini Asia spec.” (NMPC). Paratypes: 8 ♂♂ “Laos, Attapeu prov. Bolavens Plateau, bridge ca. 4 km E TAD KATAMTOK (at light) 15°07.8'N, 106°40.1'E, 260 m Jiři Hájek leg. 11-12.v.2010” (NMPC, ZFMK).

#### Description.

Length of body: 8.9 mm; length of elytra: 6.5 mm; maximum width: 5.5 mm. Surface of labroclypeus and disc of frons glabrous. Smooth area anterior to eye twice as wide as long. Eyes small, ratio of diameter/interocular width: 0.48. Ratio of length of metepisternum/metacoxa: 1/1.93. Metatibia short and wide, ratio width/length: 1/2.82; basal group of dorsal spines of metatibia at first third of metatibial length.

Aedeagus: Fig. 41E–G. Habitus: Fig. 41H.

Female unknown.

#### Variation.

Length of body: 8.6–8.9 mm; length of elytra: 6.1–6.8 mm; maximum width: 5.3–5.9 mm.

#### Diagnosis.

*Tetrasericabolavensensis* sp. n. differs from the similar *T.latefemorata* Kobayashi, 2017 by the right paramere being half as long as left paramere and the deep split between lobes of right parameres; also the dorsal lobe is not reduced in size.

#### Etymology.

The new species is named after the type locality, Bolavens Plateau (adjective in the nominative singular).

### Tetraserica nakaiensis sp. n.

Taxon classificationAnimaliaColeopteraMelolonthidae

http://zoobank.org/6161C236-02E6-4921-B96D-A6BED9BEA2A6

Figures 41 , 49

#### Type material examined.

Holotype: ♂ “Laos centr., Khammouan prov. NAKAI env., 4-8.5.1998, Route No 8 alt. 560 ± 20 m, N17°42.8’, E 105°08.9 (GPS), E Jendek & O Šauša leg./ Coll. P Pacholátko/ 200 Sericini Asia spec.” (CPPB).

#### Description.

Length of body: 8.9 mm; length of elytra: 6.6 mm; maximum width: 5.5 mm. Surface of labroclypeus and disc of frons glabrous. Smooth area anterior to eye twice as wide as long. Eyes small, ratio of diameter/interocular width: 0.5. Ratio of length of metepisternum/metacoxa: 1/1.74. Metatibia short and wide, ratio width/length: 1/2.81; basal group of dorsal spines of metatibia at first third of metatibial length.

Aedeagus: Fig. 41I–K. Habitus: Fig. 41L.

Female unknown.

#### Diagnosis.

*Tetrasericanakaiensis* sp. n. differs from the similar *T.bolavensensis* sp. n. by the left paramere being at middle distinctly widened and distinctly longer than the median phallobasal lamina. In *T.bolavensensis* the left paramere is narrow in the middle (lateral view) and only a little longer than the median phallobasal lamina.

#### Etymology.

The new species is named with reference to its occurrence in Nakai province (adjective in the nominative singular).

### Tetraserica spinicrus

Taxon classificationAnimaliaColeopteraMelolonthidae

(Frey, 1972) comb. n.

Figures 42 , 54

Neoserica spinicrus Frey, 1972: 201.

#### Type material examined.

Paratype: 1 ♂ “Laos, V 1967 Ban-Van-Eue/ Paratype Neosericaspinicrus G. Frey 1971” (CF).

#### Additional material examined.

**Laos**: 1 ♂ “Laos 1963 Umgeb. Pak Lay/ CF” (CF), 1 ♂ “X-DA4793 labcode: VD099, LAOS, Stupa GH, 5 km W Muang Sing, 750 m, 21.1482N 101.1711E, 9.v-2.vi.2011, M. Murzin, O. Shulga leg. Tetraserica sp LA_V57/ sp-LA-V57/ X-DA4793” (ZFMK), 1 ♂ “X-DA4778 labcode: VD098, LAOS, Stupa GH, 5 km W Muang Sing, 750 m, 21.1482N 101.1711E, 9.v-2.vi.2011, M. Murzin, O. Shulga leg. Tetraserica spLA_V57/ X-DA4778” (ZFMK), 1 ♂ “LAOS north, 5-11.V.1997, 20 km NW Louang Namtha, N 21°09.2, E 101°18.7, alt. 900±100 m, E. Jendek & O. Šauša leg./ Coll. Pacholátko/ 183 Sericini Asia spec.”(CPPB), 1 ♂ “Laos, 21°09'N, 101°19'E, Louangnamtha pr. Namtha-Muang Sing, 5-31.v.1997, 900–1200 m Vit. Kubáň leg/ coll. P. Pacholátko/LS 55” (CPPB), 1 ♂ “Haut Mekong Vien Poukha 3.V.1918 R.V. de Salvazza” (NHMUK).

#### Redescription.

Length of body: 9.1 mm; length of elytra: 6.8 mm; maximum width: 5.6 mm. Surface of labroclypeus and disc of frons glabrous. Smooth area anterior to eye twice as wide as long. Eyes moderately large, ratio of diameter/interocular width: 0.61. Ratio of length of metepisternum/metacoxa: 1/1.66. Posterior margin of metafemur with sharp hook. Metatibia short and wide, ratio width/length: 1/2.47; basal group of dorsal spines of metatibia at first third of metatibial length.

**Figure 42.**
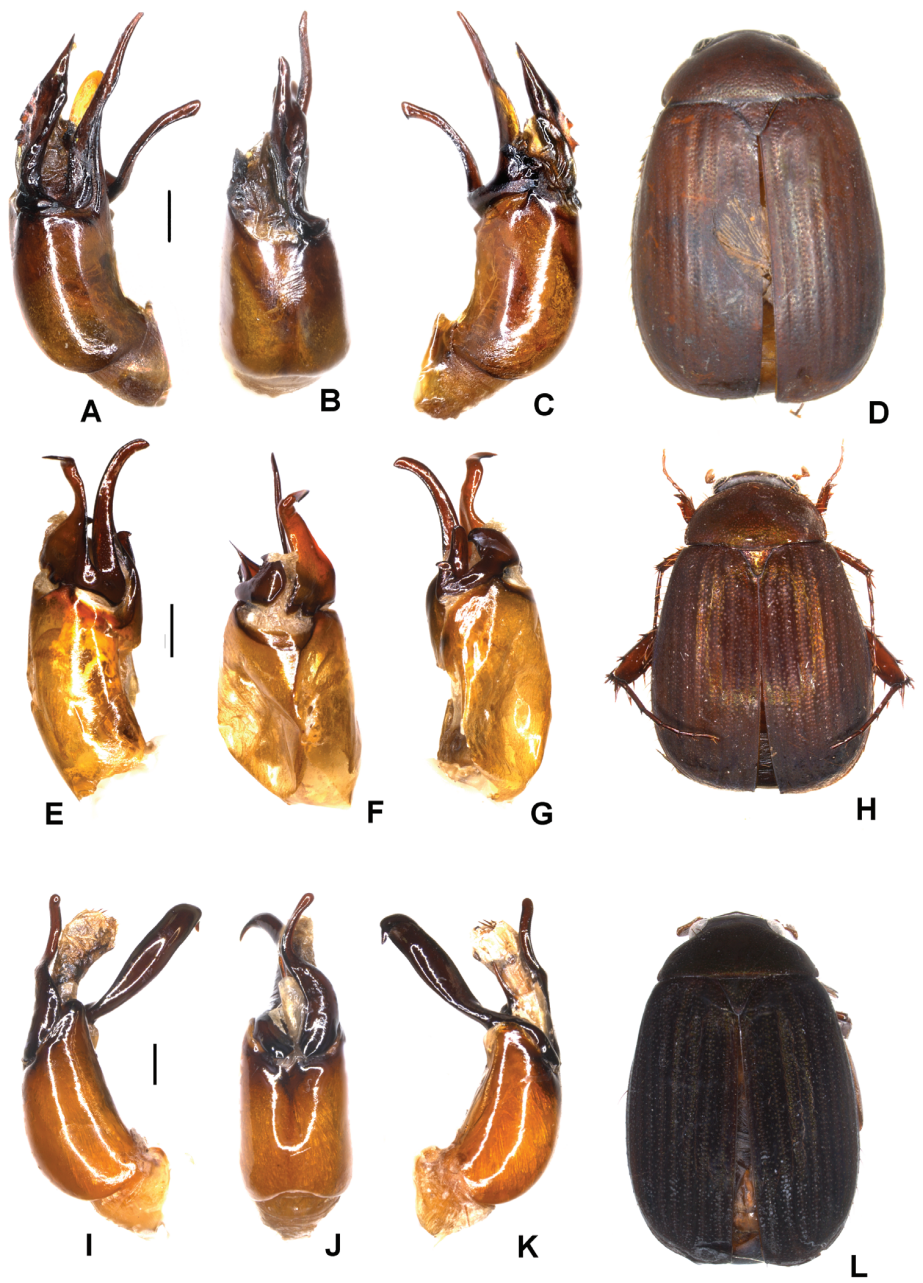
**A–D***Tetrasericaspinicrus* (Frey, 1972) (paratype) **E–H***T.lucai* sp. n. (holotype) **I–L***T.petrpacholatkoi* sp. n. (holotype) **A, E, I** aedeagus, left side lateral view **C, G, K** aedeagus, right side lateral view **B, F, J** parameres, dorsal view **D, H, L** habitus. Scale bars: 0.5 mm. Habitus not to scale.

Aedeagus: Fig. 42A–C. Habitus: Fig. 42D.

### Tetraserica lucai sp. n.

Taxon classificationAnimaliaColeopteraMelolonthidae

http://zoobank.org/2C5D0801-FA2A-4AE2-AA6D-1E033FAA364B

Figures 42 , 49

#### Type material examined.

Holotype: ♂ “S Vietnam, 14.10N 108.30E 40 km NW of An Khe Buon Luoi, 620–750 m 28.3-12.4.1995 Pacholátko & Dembický leg./ coll. P. Pacholátko/ 237 Sericini Asia spec.” (CPPB). Paratype: 1 ♂ “C Vietnam: Gia Lai Province, Kon Chu Rang Nature Reserve, surroundings HQ, about 900 m 14°28, 450'N, 108°32, 401'E/ leg. L. Bartolozzi, S. Bambi, A. Badinelli, V. Sbordoni- at light 8-12.V.2016 (n° Mag. 3078)” (MZUF).

#### Description.

Length of body: 9.4 mm; length of elytra: 7 mm; maximum width: 6.3 mm. Surface of labroclypeus and disc of frons glabrous. Smooth area anterior to eye twice as wide as long. Eyes moderately large, ratio of diameter/interocular width: 0.58. Ratio of length of metepisternum/metacoxa: 1/1.63. Posterior margin of metafemur with sharp hook. Metatibia short and wide, ratio width/length: 1/2.78; basal group of dorsal spines of metatibia at first third of metatibial length.

Aedeagus: Fig. 42E–G. Habitus: Fig. 42H.

Female unknown.

#### Variation.

Length of body: 9.4–10.1 mm; length of elytra: 7.0–7.8 mm; maximum width: 6.2–6.3 mm.

#### Diagnosis.

*Tetrasericalucai* sp. n. differs from the quite similar *T.spinicrus* (Frey, 1972) by having the metafemur not widened basally, and the sharp hook positioned behind the basal third of the metafemur.

#### Etymology.

The new species is named after one of its collectors, Dr Luca Bartolozzi (noun in genitive singular).

### Tetraserica petrpacholatkoi sp. n.

Taxon classificationAnimaliaColeopteraMelolonthidae

http://zoobank.org/D144DE87-458B-4930-9318-305BB69413B8

Figures 42 , 51

#### Type material examined.

Holotype: ♂ “X-DA4697 labcode: VD087 VIETNAM Vinh Phuc Prov., Tam Dao National Park (950 m) 1-4/VI/2013, Legit L. Bartolozzi, S. Bambi, F. Cianferoni, G. Mazza, E. Orbach (n° Mag. 2950), Leihgabe Florenz, Tetraserica sp VI_V48/ 241 Sericini Asia spec./ X-DA4697” (VNMN). Paratype: 1 ♂ “N-Vietnam, (TONKIN) TAMDAO 12-24.5.1989. Pacholátko/ coll. P. Pacholátko/ VS 47” (CPPB).

#### Description.

Length of body: 10 mm; length of elytra: 8.1 mm; maximum width: 6.5 mm. Surface of labroclypeus and disc of frons glabrous. Smooth area anterior to eye twice as wide as long. Eyes moderately large, ratio of diameter/interocular width: 0.59. Ratio of length of metepisternum/metacoxa: 1/1.6. Metatibia moderately long and wide, ratio width/length: 1/3.33; basal group of dorsal spines of metatibia at first third of metatibial length.

Aedeagus: Fig. 42I–K. Habitus: Fig. 42L.

Female unknown.

#### Variation.

Length of body: 9.8–10.0 mm; length of elytra: 7.1–8.1 mm; maximum width: 6.0–6.5 mm.

#### Diagnosis.

*Tetrasericapetrpacholatkoi* sp. n. differs from *T.constanti* sp. n. and *T.vientianeensis* sp. n. by the left paramere being half as long as the phallobase.

#### Etymology.

The new species is named after one of its collectors, Petr Pacholátko (noun in genitive singular).

### Tetraserica senohi

Taxon classificationAnimaliaColeopteraMelolonthidae

Kobayashi, 2018

Figures 43 , 55

Tetraserica senohi Kobayashi, 2018: 59, figs 2, 6–9.

#### Material examined.

**Thailand**: 1 ♂ “N THAILAND: Chiang Mai Pref., Ban Angkhai, Samoeng Dist., 750 m, 15-20.V.1998 K. Masumoto leg. /coll. Dirk Ahrens/ 134 Sericini Asia spec.” (ZFMK), 2 ♂♂ “Chiang Mai, N. Thailand, V.1983 Doi Suthep/ Museum Leiden coll. P.J.J.H. Kuijten” (RMNH), 1 ♂ “NW Thailand, 9.-16.5 Mae Hong Son, 1991 Ban Huai Po, 1600–2000 m Horák leg/ coll. P. Pacholátko” (CPPB), 1 ♂ “NW Thailand, 19.19N, 97.59E, Mae Hong Son, 1991 Ban Huai Po, 1600–2000 m 17.-23.5., L. Dembický leg.” (NHMW), 1 ♂, 1 ♀ “Doi Suthep Chieng Mai N-Thailand 21.V.1983 leg.” (ZMHB), 1 ♂, 1 ♀ “Doi Suthep Chieng Mai N-Thailand 22.V.1983 leg.” (ZMHB).

#### Redescription.

Length of body: 8.4 mm; length of elytra: 6.6 mm; maximum width: 5.4 mm. Surface of labroclypeus and disc of frons glabrous. Smooth area anterior to eye twice as wide as long. Eyes small, ratio of diameter/interocular width: 0.54. Ratio of length of metepisternum/metacoxa: 1/1.68. Metatibia short and wide, ratio width/length: 1/3.21; basal group of dorsal spines of metatibia at first third of metatibial length.

**Figure 43. A–E:**
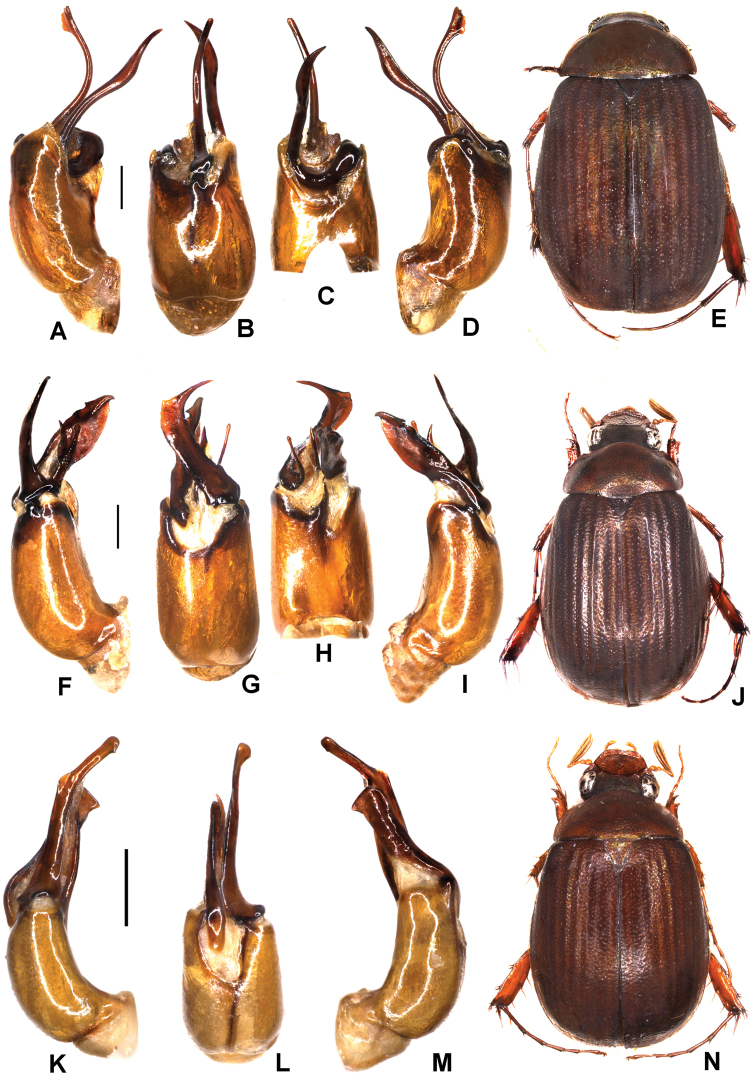
*Tetrasericasenohi* Kobayashi, 2018 (Thailand: Ban Angkhai) **F–J***T.rihai* sp. n. (holotype) **K–N***T.doipuiensis* sp. n. (holotype) **A, F, K** aedeagus, left side lateral view **D, I, M** aedeagus, right side lateral view **B, G, L** parameres, dorsal view **C, H** parameres, ventral view **E, J, N** habitus. Scale bars: 0.5 mm. Habitus not to scale.

Aedeagus: Fig. 43A–D. Habitus: Fig. 43E.

### Tetraserica rihai sp. n.

Taxon classificationAnimaliaColeopteraMelolonthidae

http://zoobank.org/B11102C4-9B53-48E5-B19B-C4163F4E4C67

Figures 43 , 50

#### Type material examined.

Holotype: ♂ “Thai, N, Nan prov., Doi Phu Kha N.P., Headq., 19°13'N, 101°07'E, 28.iv-1.v.1999, M. Riha leg./ coll. P. Pacholátko/ 124 Sericini Asia spec.” (CPPB).

#### Description.

Length of body: 9.5 mm; length of elytra: 6.5 mm; maximum width: 5.5 mm. Surface of labroclypeus and disc of frons glabrous. Smooth area anterior to eye twice as wide as long. Eyes moderately large, ratio of diameter/interocular width: 0.56. Ratio of length of metepisternum/metacoxa: 1/1.69. Metatibia short and wide, ratio width/length: 1/3.06; basal group of dorsal spines of metatibia at first third of metatibial length.

Aedeagus: Fig. 43F–I. Habitus: Fig. 43J.

Female unknown.

#### Diagnosis.

*Tetrasericarihai* sp. n. differs from *T.nonglomensis* sp. n. and *T.nussi* sp. n. by the dorsal lobe of left paramere being twice as long as the median ventral lamina of phallobase.

#### Etymology.

The new species is named after one of its collectors, M Riha (noun in genitive singular).

### Tetraserica doipuiensis sp. n.

Taxon classificationAnimaliaColeopteraMelolonthidae

http://zoobank.org/B2DEA154-A5AE-4134-8CB1-54E597057D84

Figures 43 , 49

#### Type material examined.

Holotype: ♂ “Thai, Chiang Mai prov., 18°49'N, 98°54'E, 1600 m, DOI PUI mt., 2-6.v., Vít Kubáň leg.1996/ coll. P Pacholátko/ 899 Sericini Asia spec.” (CPPB).

#### Description.

Length of body: 6.3 mm; length of elytra: 4.5 mm; maximum width: 3.8 mm. Surface of labroclypeus and disc of frons glabrous. Smooth area anterior to eye twice as wide as long. Eyes moderately large, ratio of diameter/interocular width: 0.62. Ratio of length of metepisternum/metacoxa: 1/1.55. Metatibia short and wide, ratio width/length: 1/3.1; basal group of dorsal spines of metatibia at first third of metatibial length.

Aedeagus: Fig. 43K–M. Habitus: Fig. 43N.

Female unknown.

#### Diagnosis.

*Tetrasericadoipuiensis* sp. n. differs from the similar *T.champassakana* sp. n. by the left paramere lacking the blunt lateral tooth before the apex and the right paramere being as long as medial phallobasal lamina.

#### Etymology.

The new species is named after the type locality, Mt Doi Pui (adjective in the nominative singular).

### Tetraserica doiphukhaensis sp. n.

Taxon classificationAnimaliaColeopteraMelolonthidae

http://zoobank.org/E1AC2466-836E-4B50-9375-18A16C741DB6

Figures 44 , 46

#### Type material examined.

Holotype: ♂ “Thai, N, Nan prov., Doi Phu Kha N.P, Headq., 19°13'N, 101°7'E, 28.iv-1.v.1999., M Riha leg. /coll. P Pacholátko/ 139 Sericini Asia spec.” (CPPB).

#### Description.

Length of body: 8.8 mm; length of elytra: 6.3 mm; maximum width: 5.3 mm. Surface of labroclypeus and disc of frons glabrous. Smooth area anterior to eye twice as wide as long. Eyes small, ratio of diameter/interocular width: 0.54. Ratio of length of metepisternum/metacoxa: 1/1.75. Metatibia moderately long and wide, ratio width/length: 1/3.5; basal group of dorsal spines of metatibia at first third of metatibial length.

**Figure 44.**
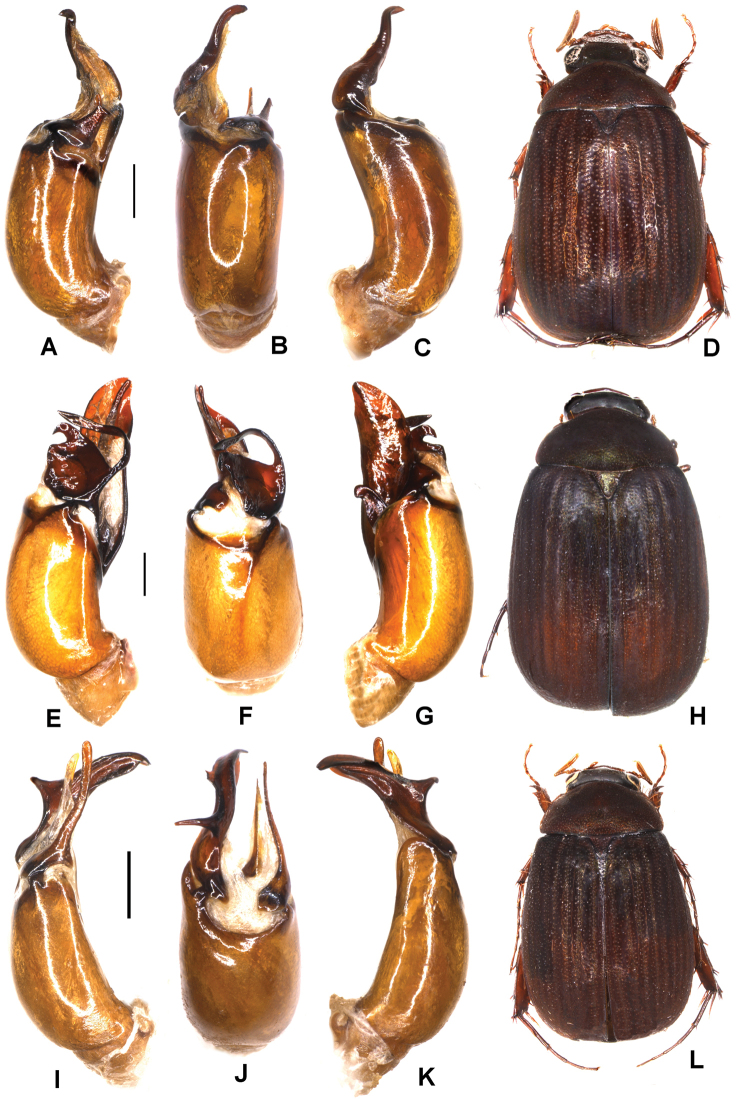
**A–D***Tetrasericadoiphukhaensis* sp. n. (holotype) **E–H***T.kontumensis* sp. n. (holotype) **I–L***T.semidamadiensis* sp. n. (holotype) **A, E, I** aedeagus, left side lateral view **C, G, K** aedeagus, right side lateral view **B, F, J** parameres, dorsal view **D, H, L** habitus. Scale bars: 0.5 mm. Habitus not to scale.

Aedeagus: Fig. 44A–C. Habitus: Fig. 44D.

Female unknown.

#### Diagnosis.

*Tetrasericadoiphukhaensis* sp. n. differs from all other *Tetraserica* in having a very short median phallobasal lamina, by having the left paramere short and stout, and a simple right paramere without a dorsal lobe.

#### Etymology.

The new species is named after the type locality, Doi Phu Kha NP (adjective in the nominative singular).

### Tetraserica kontumensis sp. n.

Taxon classificationAnimaliaColeopteraMelolonthidae

http://zoobank.org/2304423E-00A3-41DE-9C88-A6DFE74C6CFB

Figures 44 , 48

#### Type material examined.

Holotype: ♂ “X-DA4692 labcode VD085, VIETNAM, Kon Tum Prov. W border of Ngoc Linh Nat. Park, 20 km N Dak Glei (900 m) (at light), 5°14'26.04"N, 107°44'11.61"E, 30.V.2014, legit L Bartolozzi, G Chelazzi, A Bandinelli, S Bambi, F Fabiano (n° Magazz. 2978) Tetraserica spVI_V46” (VNMN).

#### Description.

Length of body: 10 mm; length of elytra: 7.4 mm; maximum width: 6.1 mm. Surface of labroclypeus and disc of frons glabrous. Smooth area anterior to eye twice as wide as long. Eyes small, ratio of diameter/interocular width: 0.52. Ratio of length of metepisternum/metacoxa: 1/1.7. Posterior margin of metafemur with sharp hook. Metatibia short and wide, ratio width/length: 1/3.17; basal group of dorsal spines of metatibia at first third of metatibial length.

Aedeagus: Fig. 44E–G. Habitus: Fig. 44H.

Female unknown.

#### Diagnosis.

*Tetrasericakontumensis* sp. n. differs from all other *Tetraserica* species by having a short and wide left paramere, split into a dorsal hook and a basal filiform branch which is also turned dorsally.

#### Etymology.

The new species is named with reference to its occurrence in the Kon Tum province (adjective in the nominative singular).

#### Remarks.

The correct GPS coordinates of the type locality are 15°14'26.04"N, 107°44'11.61"E.

### Tetraserica semidamadiensis sp. n.

Taxon classificationAnimaliaColeopteraMelolonthidae

http://zoobank.org/BAADD476-7EB1-494F-BE2B-CC5FC47B1F5F

Figures 44 , 51

#### Type material examined.

Holotype: ♂ “Laos centr., Bolikhamxai prov. BAN NAPE - Kaew Nua Pass, 18.4-1.5.1998, alt. 600 ± 100 m, N 18°22.3, E105°09.1 (GPS), E Jendek & O Šauša leg./ coll. P. Pacholátko/ 199 Sericini Asia spec.” (CPPB).

#### Description.

Length of body: 7.8 mm; length of elytra: 5.6 mm; maximum width: 5 mm. Surface of labroclypeus and disc of frons glabrous. Smooth area anterior to eye twice as wide as long. Eyes small, ratio of diameter/interocular width: 0.52. Ratio of length of metepisternum/metacoxa: 1/1.56. Metatibia moderately long and wide, ratio width/length: 1/3.75; basal group of dorsal spines of metatibia at first third of metatibial length.

Aedeagus: Fig. 44I–K. Habitus: Fig. 44L.

Female unknown.

#### Diagnosis.

*Tetrasericasemidamadiensis* sp. n. is very similar to *T.damadiensis* Liu et al., 2014 but the new species differs by the dorsolateral tooth being situated at the basal third of the right paramere, and by the wider right paramere (lateral view).

#### Etymology.

The species name (adjective in the nominative singular) is derived from the combined Latin words *semi*- (half) and the species name *damadiensis*, with reference to the similarity to *T.damadiensis* Liu et al., 2014.

### Tetraserica damaidiensis

Taxon classificationAnimaliaColeopteraMelolonthidae

Liu, Fabrizi, Bai, Yang, Ahrens, 2014

Figures 45 , 46

Tetraserica damaidiensis Liu, Fabrizi, Bai, Yang, Ahrens, 2014: 96, fig. 3E–H.

#### Material examined.

**Vietnam**: 3 ♂♂ “Vietnam N 1990 Sa-Pa, 11-19.VI, 1500 m, Hoang Lien Son prov. Strnad Jan lgt.” (NHMB), 1 ex. “N-Vietnam Cao Bang Prov., vic Tinh Tuc, Son Dong, Nui Pia Oac Nature Reserve, 9-15.V.2014, 22°37'55"N, 105°52'98"E, 850–1300 m, leg. A Skale” (CASH), 17 ♂♂ “Vietnam-N Cao Bang prov., 12 km NE Cao Bang, 650 ± 50 m, 22°45'45"N, 106°19'E, L Dembicky leg., 15-16.V.2010” (ZFMK).

**Figure 45.**
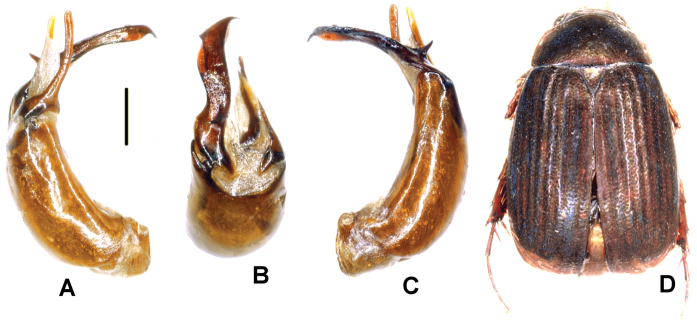
**A–D***Tetrasericadamaidiensis* Liu et al., 2014 (holotype) **A** aedeagus, left side lateral view **C** aedeagus, right side lateral view **B** parameres, dorsal view **D** habitus. Scale bar: 0.5 mm. Habitus not to scale.

Aedeagus: Fig. 45A–C. Habitus: Fig. 45D.

**Figure 46.**
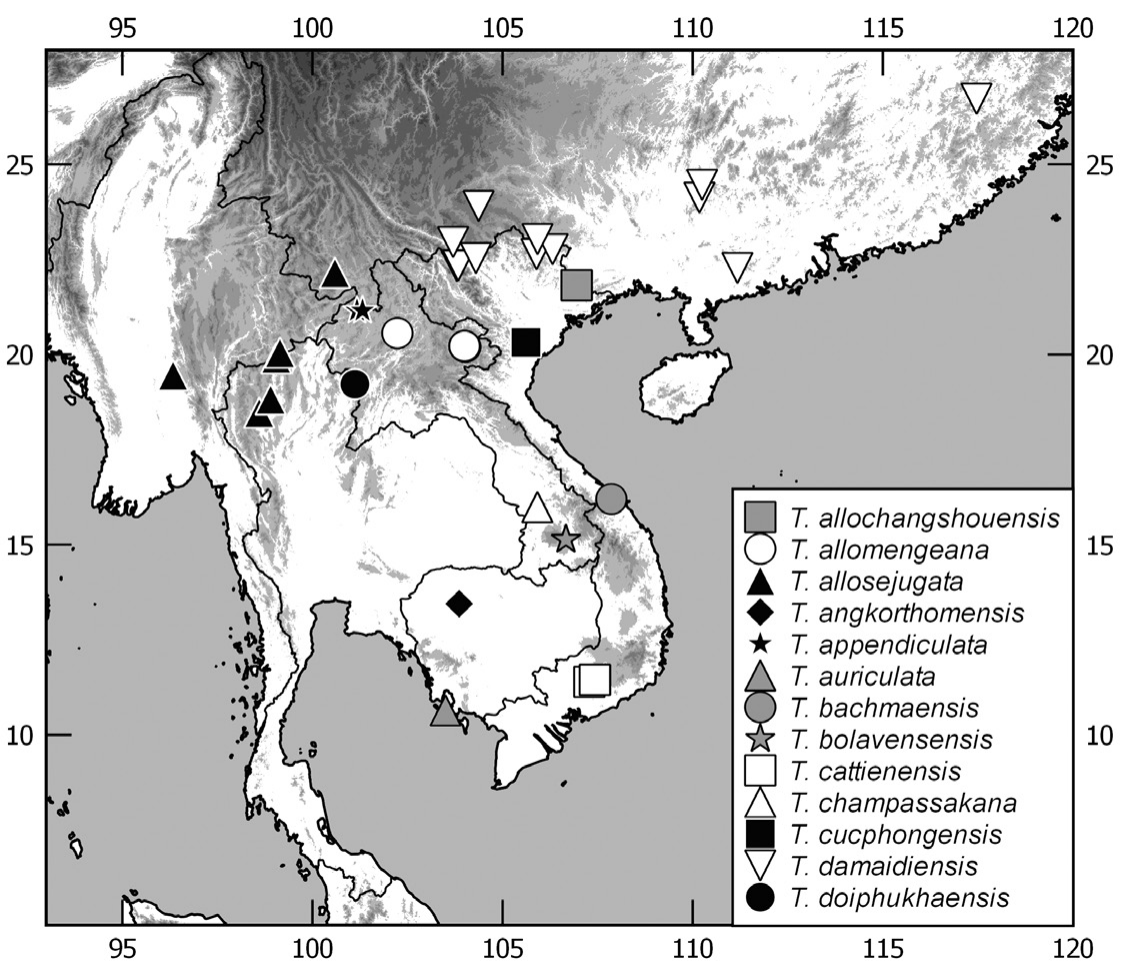
Distribution map of *Tetrasericaallochangshouensis* sp. n., *T.allomengeana* sp. n., *T.allosejugata* sp. n., *T.angkorthomensis* sp. n., *T.appendiculata* sp. n., *T.auriculata* sp. n., *T.bachmaensis* sp. n., *T.bolavensensis* sp. n., *T.cattienensis* sp. n., *T.champassakana* sp. n., *T.cucphongensis* sp. n., *T.damaidiensis* Liu et al., 2014, *T.doiphukhaensis* sp. n.

**Figure 47.**
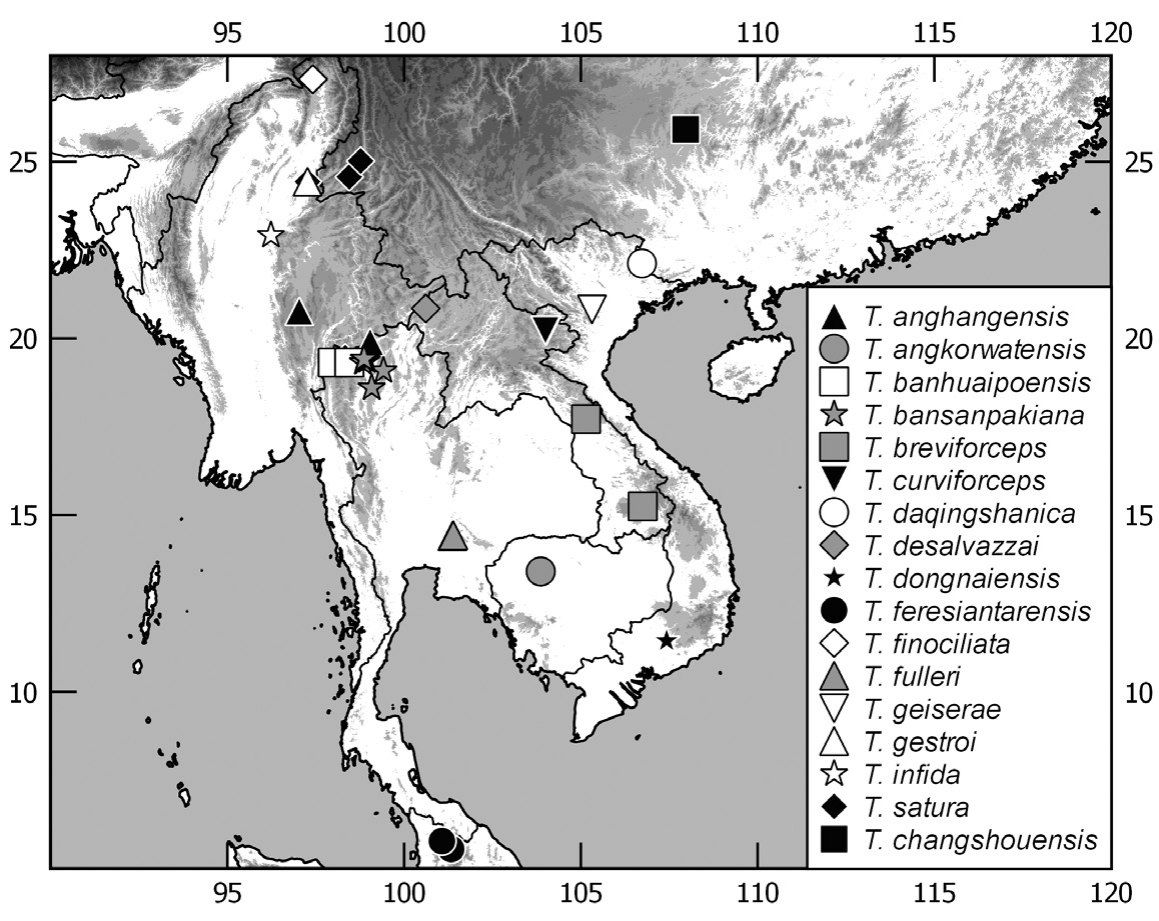
Distribution map of *Tetrasericaangkhangensis* Kobayashi, 2017, *T.angkorwatensis* sp. n., *T.banhuaipoensis* sp. n., *T.bansanpakiana* sp. n., *T.breviforceps* sp. n., *T.changshouensis* Liu et al., 2014, *T.curviforceps* sp. n., *T.daqingshanica* Liu et al., 2014, *T.desalvazzai sp. n.*, *T.dongnaiensis* sp. n., *T.feresiantarensis* sp. n., *T.finociliata* sp. n., *T.fulleri* sp. n., *T.geiserae* sp. n., *T.gestroi* (Brenske), *T.satura* (Brenske), *T.infida* sp. n.

**Figure 48.**
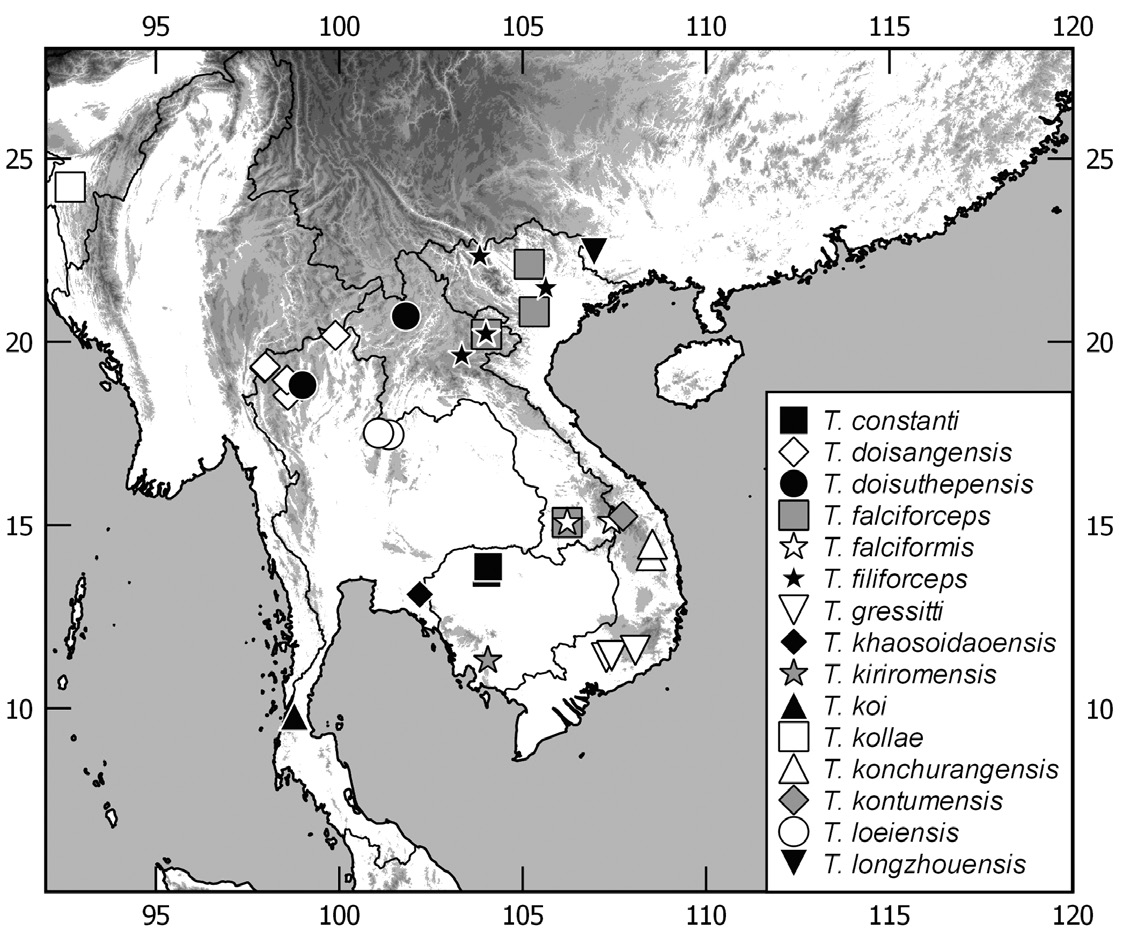
Distribution map of *Tetrasericaconstanti* sp. n., *T.doisangensis* Kobayashi, 2017, *T.doisuthepensis* sp. n., *T.falciforceps* sp. n., *T.falciformis* sp. n., *T.filiforceps* sp. n., *T.gressitti* (Frey, 1972), *T.khaosoidaoensis* sp. n., *T.kiriromensis* sp. n., *T.koi* sp. n., *T.kollae* sp. n., *T.konchurangensis* sp. n., *T.kontumensis* sp. n., *T.loeiensis* sp. n., *T.longzhouensis* Liu et al., 2014.

**Figure 49.**
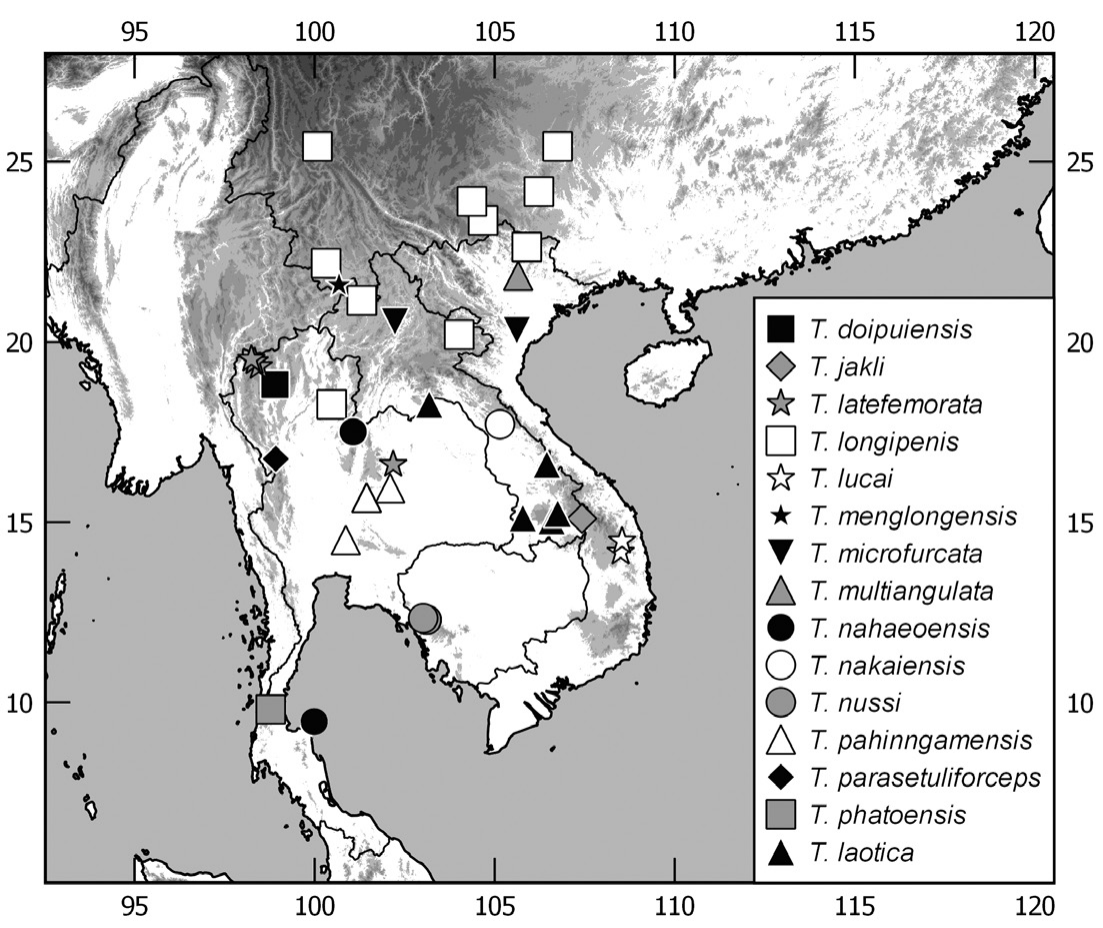
Distribution map of *Tetrasericadoipuiensis* sp. n., *T.jakli* sp. n., *T.laotica* (Frey, 1972), *T.latefemorata* Kobayashi, 2017, *T.longipenis* Liu et al., 2014, *T.lucai* sp. n., *T.menglongensis* Liu et al., 2014, *T.microfurcata* sp. n., *T.multiangulata* sp. n., *T.nahaeoensis* sp. n., *T.nakaiensis* sp. n., *T.nussi* sp. n., *T.pahinngamensis* sp. n., *T.parasetuliforceps* sp. n., *T.phatoensis* sp. n.

**Figure 50.**
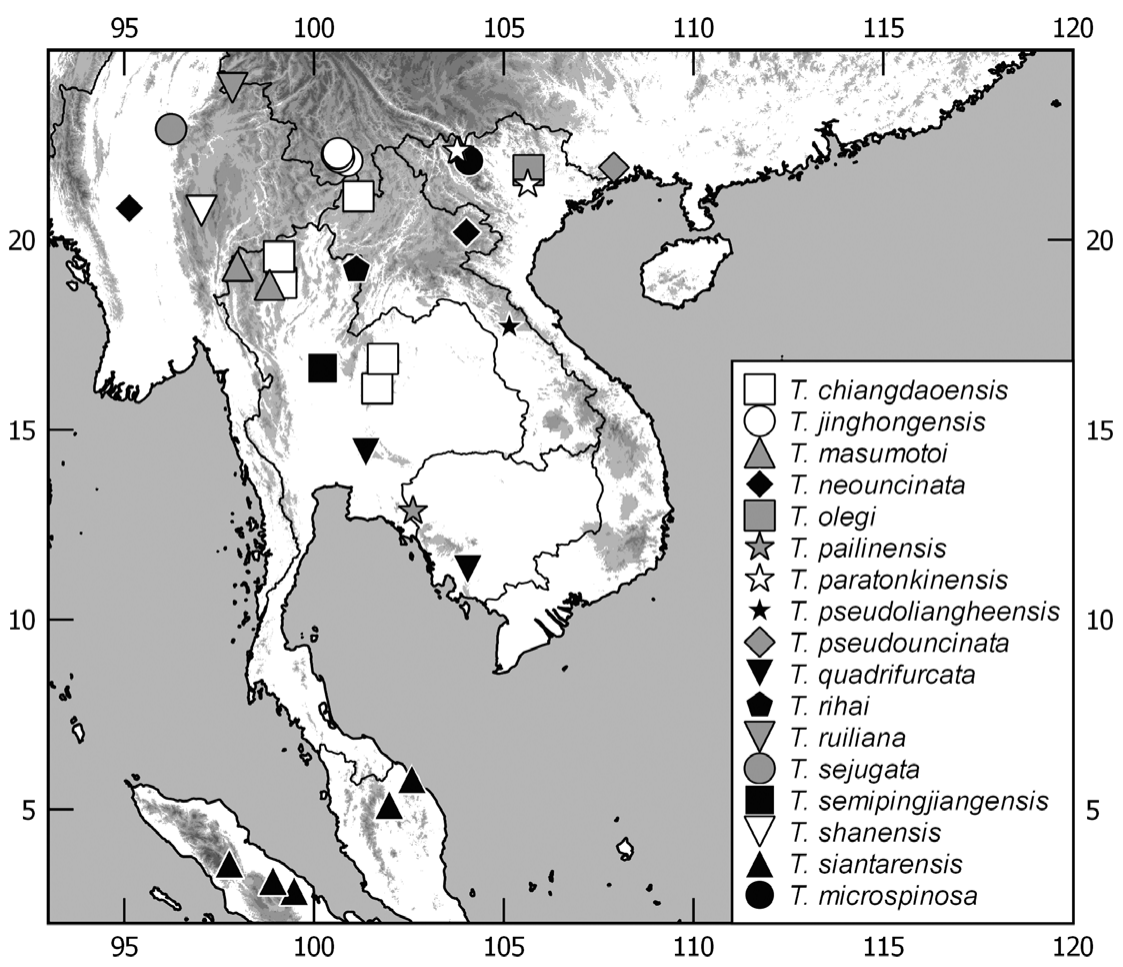
Distribution map of *Tetrasericachiangdaoensis* Kobayashi, 2017, *T.jinghongensis* Liu, Fabrizi, Bai, Yang & Ahrens, 2014, *T.masumotoi* Kobayashi, 2017, *T.microspinosa* sp. n., *T.neouncinata* sp. n., *T.olegi* sp. n., *T.pailinensis* sp. n., *T.paratonkinensis* sp. n., *T.pseudoliangheensis* sp. n., *T.pseudouncinata* sp. n., *T.quadrifurcata* sp. n., *T.rihai* sp. n., *T.ruiliana*, *T.sejugata* (Brenske, 1898), *T.semipingjiangensis* sp. n., *T.shanensis* sp. n., *T.siantarensis* (Moser, 1922).

**Figure 51.**
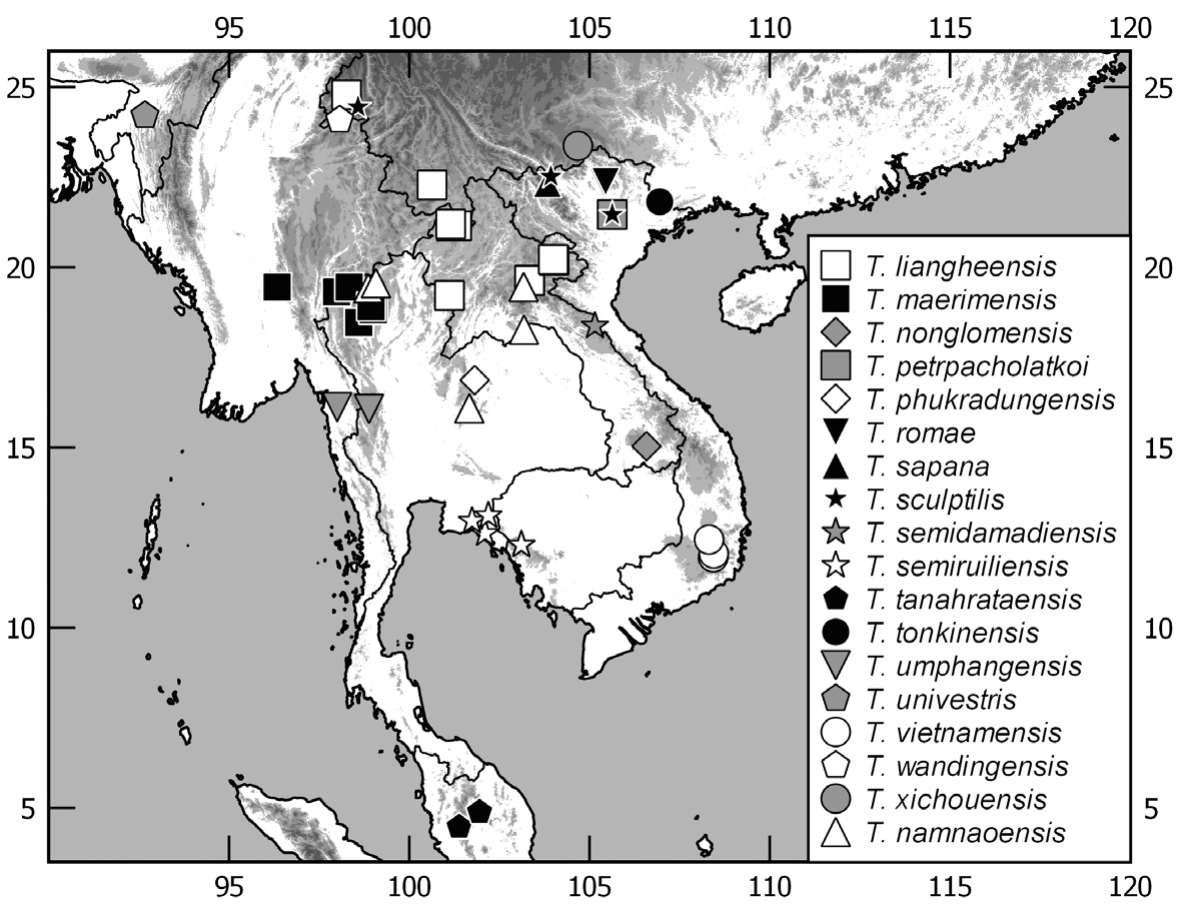
Distribution map of *Tetrasericaliangheensis* Liu et al., 2014, *T.maerimensis* Kobayashi, 2018, *T.namnaoensis* sp. n., *T.nonglomensis* sp. n., *T.petrpacholatkoi* sp. n., *T.phukradungensis* sp. n., *T.romae* sp. n., *T.rubrithorax* sp. n., *T.sapana* sp. n., *T.semidamadiensis* sp. n., *T.semiruiliensis* sp. n., *T.tanahrataensis* sp. n., *T.tonkinensis* (Moser, 1908), *T.umphangensis* sp. n., *T.univestris* Ahrens & Fabrizi, 2016, *T.vietnamensis* (Frey, 1969), *T.wandingensis* Liu et al., 2014, *T.xichouensis* Liu et al., 2014.

**Figure 52.**
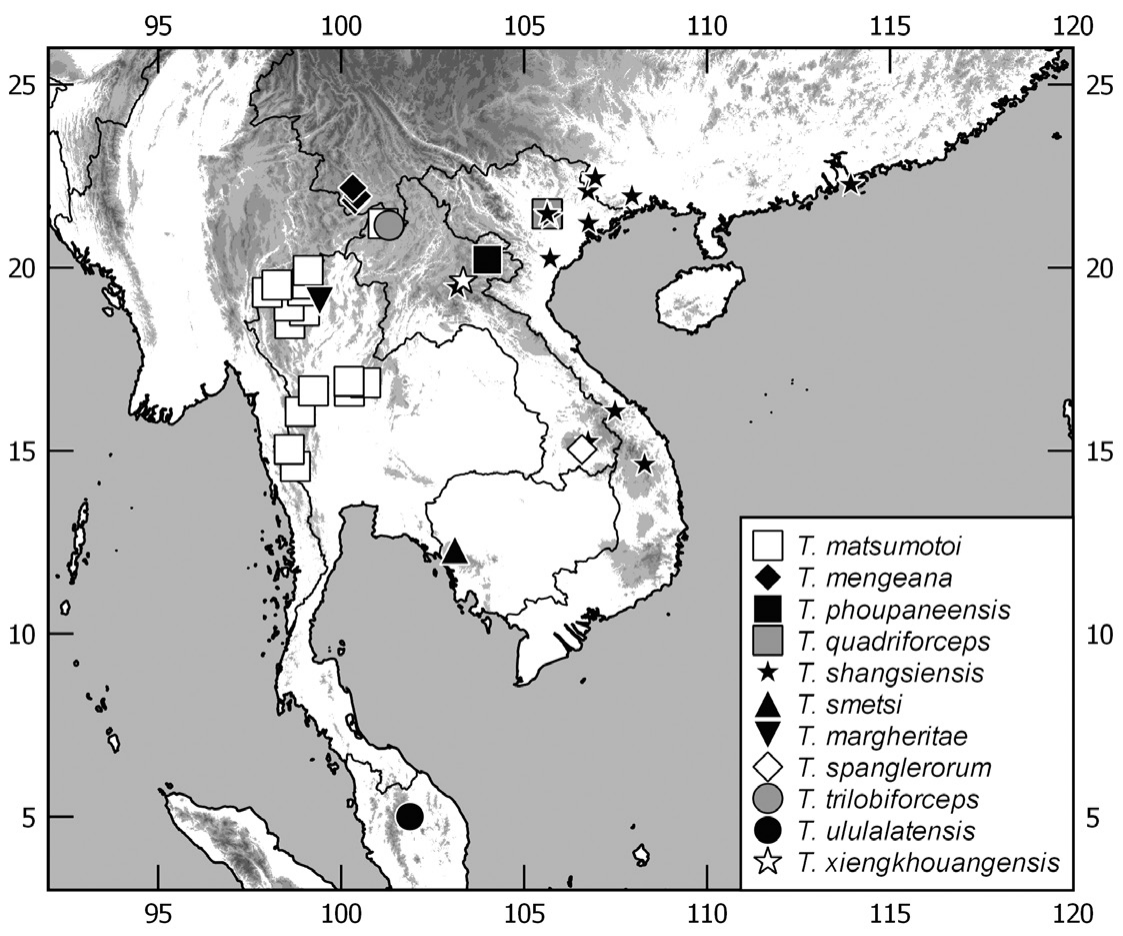
Distribution map of *Tetrasericamargheritae* sp. n., *T.matsumotoi* Kobayashi, 2017, *T.mengeana* Liu et al., 2014, *T.phoupaneensis* sp. n., *T.quadriforceps* sp. n., *T.shangsiensis* Liu et al., 2014, *T.smetsi* sp. n., *T.spanglerorum* sp. n., *T.trilobiforceps* sp. n., *T.ululalatensis* sp. n., *T.xiengkhouangensis* sp. n.

**Figure 53.**
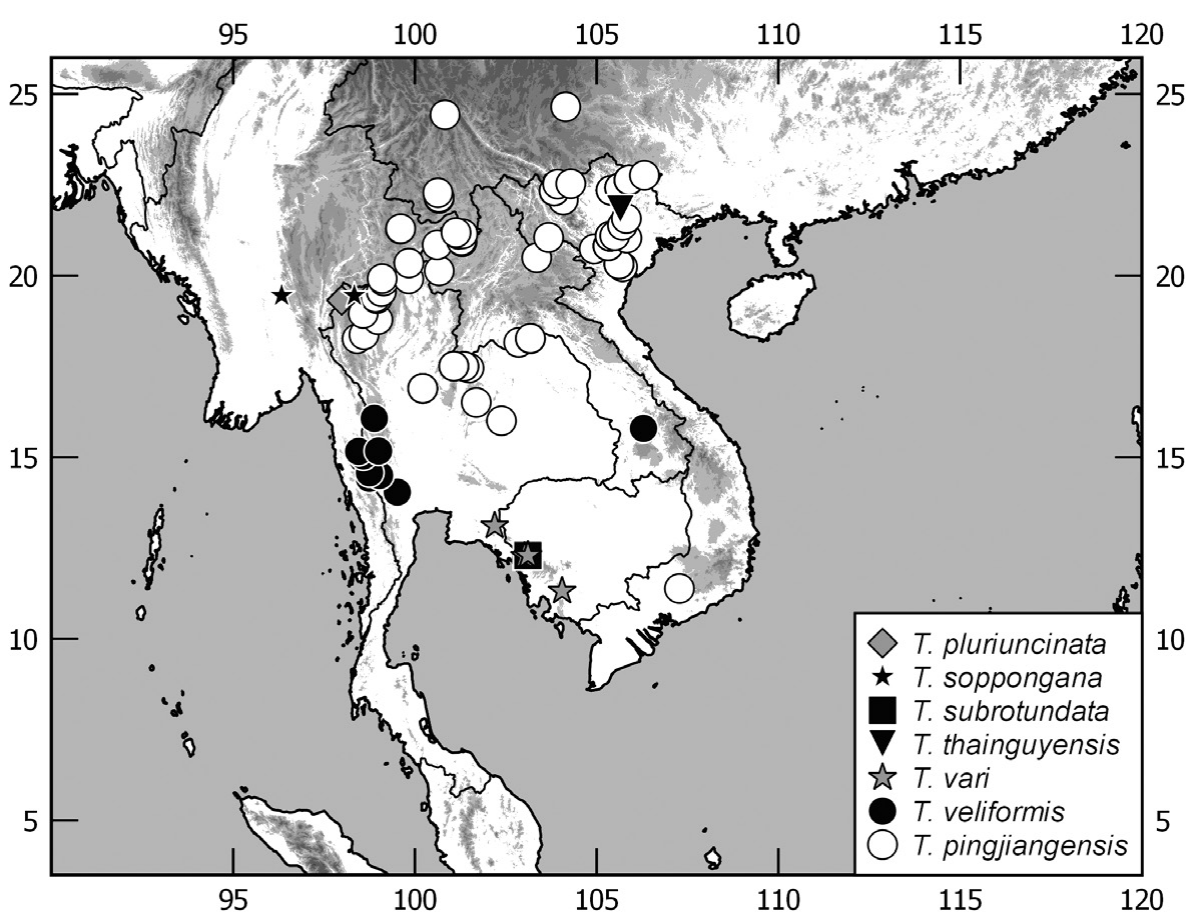
Distribution map of *Tetrasericapingjiangensis* Liu et al., 2014, *T.pluriuncinata* sp. n., *T.soppongana* sp. n., *T.subrotundata* sp. n., *T.thainguyensis* sp. n., *T.vari* sp. n., *T.veliformis* sp. n.

**Figure 54.**
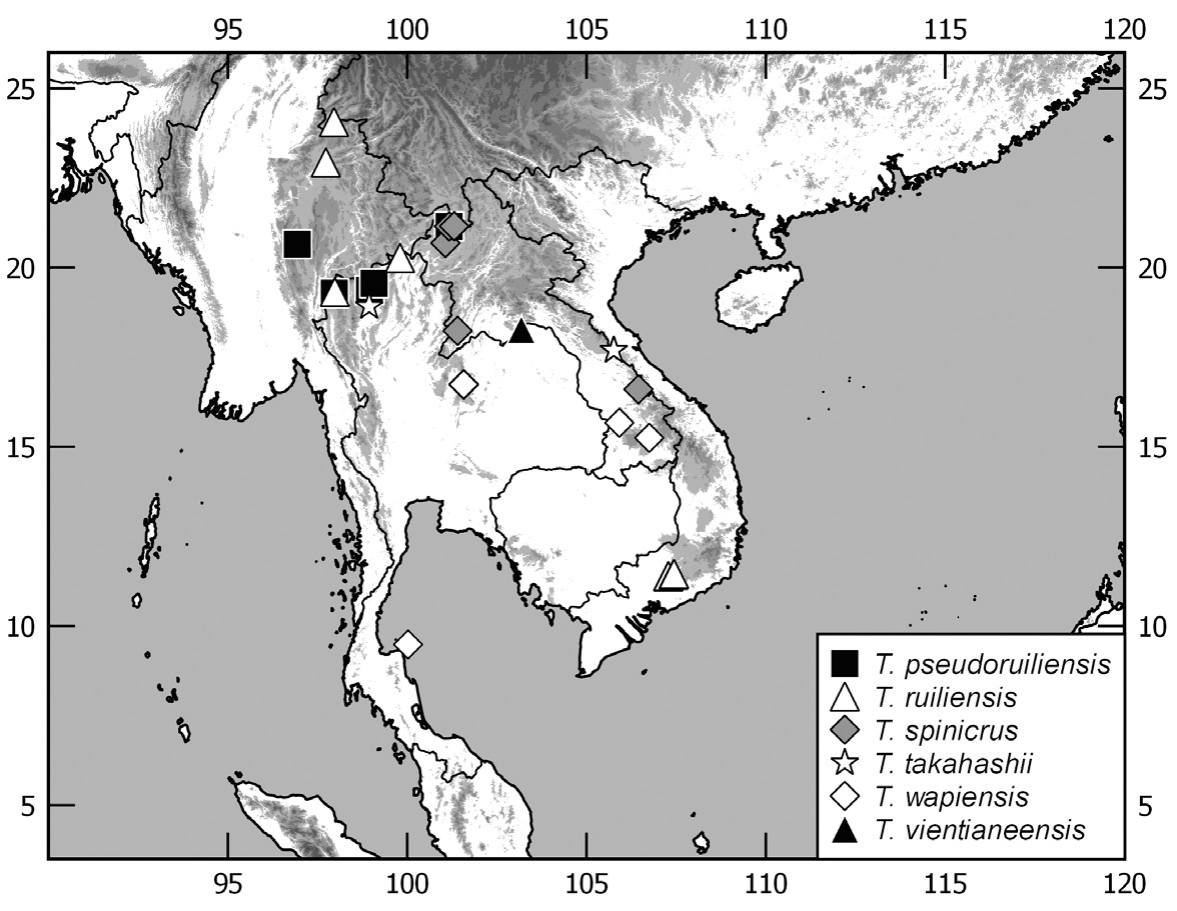
Distribution map of *Tetrasericapseudoruiliensis* sp. n., *T.ruiliensis* Liu et al., 2014, *T.spinicrus* (Frey, 1972), *T.takahashii* Kobayashi, 2017, *T.vientianeensis* sp. n., *T.wapiensis* (Frey, 1972).

**Figure 55.**
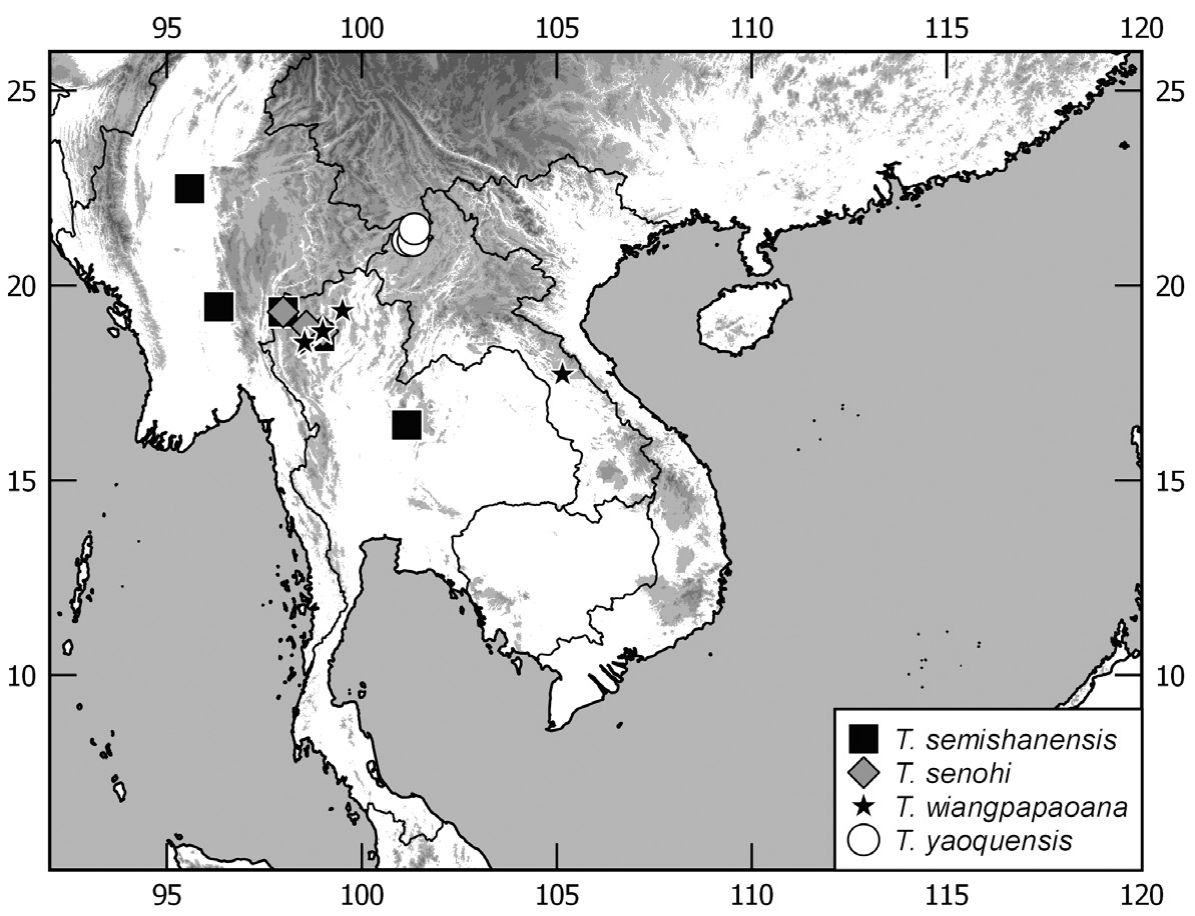
Distribution map of *Tetrasericasemishanensis* sp. n., *T.senohi* Kobayashi, 2018, *T.wiangpapaoana* Kobayashi, 2017, *T.yaoquensis* Liu et al., 2014.

**Figure 56.**
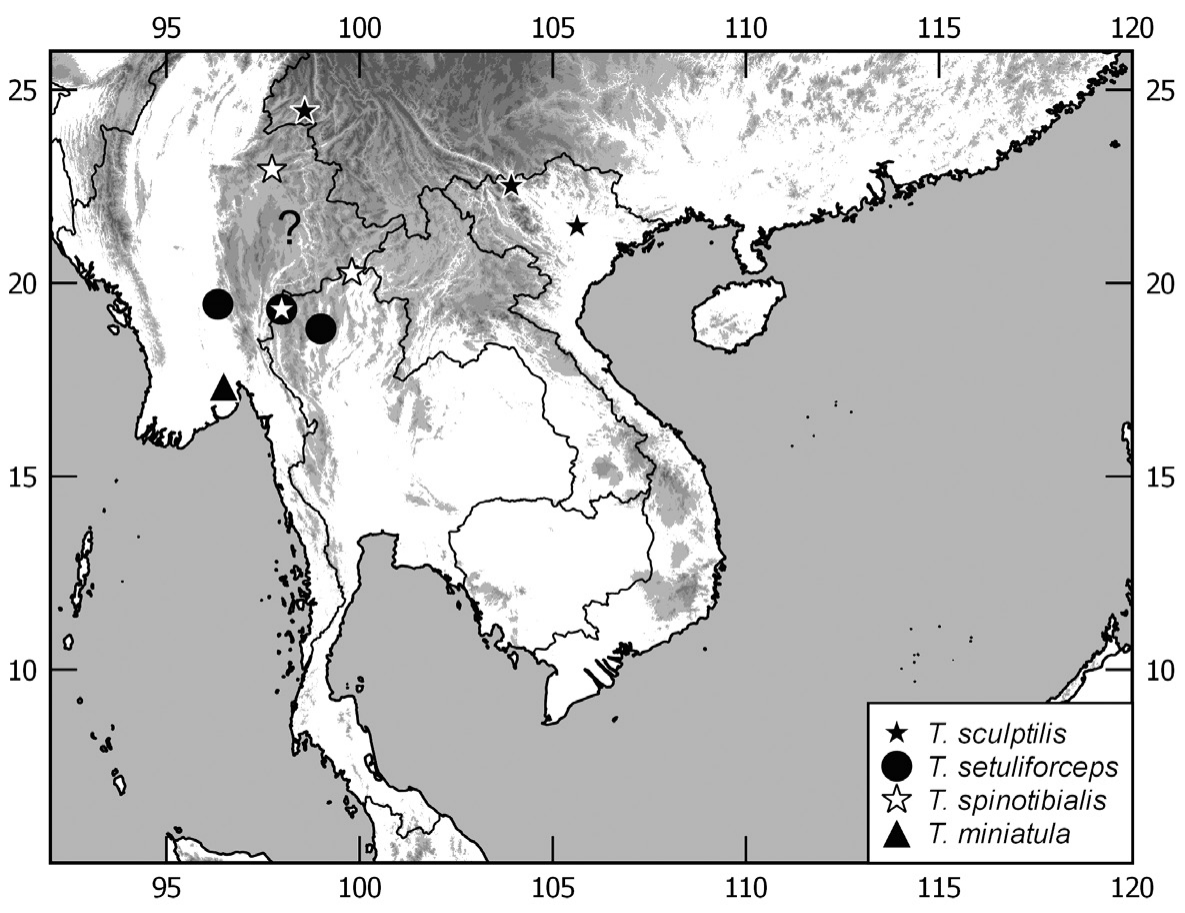
Distribution map of *Tetrasericagiulianae* sp. n. (? – not located exactly in Shan state), *T.miniatula* (Moser), *T.sculptilis* Liu et al., 2014, *T.setuliforceps* sp. n., *T.spinotibialis* sp. n.

## Appendix I

**Table 1.** Overview on the distribution of *Tetraserica* species in Indochina and the Asian mainland. * - records from Kobayashi (2017), not checked.

Species	Nepal	NW India	Darjeeling/ Sikkim	Bhutan	NE India* (Assam, Meghalaya, Mizoram, Nagaland, Manipur)	Thailand	Laos	Cambodia	Vietnam	Myanmar	CH-Yunnan	CH-Sichuan	CH-Hainan	CH-Hubei	CH-Hunan	CH-Fujian	CH-Guangdong	CH-Jianxi	CH-Guangxi	CH-Guizhou	CH-Shaanxi	CH-Chongqing	CH-Gansu	Malay peninsula	Sumatra
*T.allochangshouensis* sp. n.									x																
*T.allomengeana* sp. n.							x																		
*T.allosejugata* sp. n.						x					x														
*T.angkhangensis* Kobayashi, 2017						x				x															
*T.angkorthomensis* sp. n.								x																	
*T.angkorwatensis* sp. n.								x																	
*T.appendiculata* sp. n.							x																		
*T.auriculata* sp. n.								x																	
*T.bachmaensis* sp. n.									x																
*T.banhuaipoensis* sp. n.						x																			
*T.bansanpakiana* sp. n.						x																			
*T.bendai* Ahrens & Fabrizi, 2016					x																				
*T.bolavensensis* sp. n.							x																		
*T.brahmaputrae* Ahrens, 2004	x		x		x																				
*T.breviforceps* sp. n.							x																		
*T.cattienensis* sp. n.									x																
*T.champassakana* sp. n.							x																		
*T.changjiangensis* Liu et al., 2014													x												
*T.changshouensis* Liu et al., 2014												x		x						x					
*T.chiangdaoensis* Kobayashi, 2017						x	x																		
*T.constanti* sp. n.								x																	
*T.crenatula* Ahrens & Fabrizi, 2009					x																				
*T.cucphongensis* sp. n.									x																
*T.curviforceps* sp. n.							x																		
*T.damaidiensis* Liu et al., 2014									x		x								x						
*T.daqingshanica* Liu et al., 2014																			x						
*T.desalvazzai* sp. n.							x																		
*T.disoccupata* Ahrens, 2004			x																						
*T.doiphukhaensis* sp. n.						x																			
*T.doipuiensis* sp. n.						x																			
*T.doisangensis* Kobayashi, 2017						x																			
*T.doisuthepensis* sp. n.						x																			
*T.dongnaiensis* sp. n.									x																
*T.falciforceps* sp. n.							x		x																
*T.falciformis* sp. n.							x																		
*T.feresiantarensis* sp. n.						x																		x	
*T.ferrugata* (Blanchard), 1850	x	x																							
*T.fikaceki* Liu et al., 2014													x												
*T.filiforceps* sp. n.							x		x																
*T.finociliata* sp. n.										x															
*T.fulleri* sp. n.						x																			
*T.geiserae* sp. n.									x																
*T.gestroi* Brenske										x															
*T.giulianae* sp. n.										x															
*T.gressitti* (Frey, 1972)									x																
*T.hilaris* Ahrens & Fabrizi, 2009					x																				
*T.impar* Ahrens & Fabrizi, 2016					x																				
*T.infida* sp. n.										x															
*T.jakli* sp. n.							x																		
*T.jinghongensis* Liu et al., 2014						*					x														
*T.khaosoidaoensis* sp. n.						x																			
*T.kiriromensis* sp. n.								x																	
*T.koi* sp. n.						x																			
*T.kollae* sp. n.					x																				
*T.konchurangensis* sp. n.									x																
*T.kontumensis* sp. n.									x																
*T.laotica* (Frey, 1972)						x	x																		
*T.latefemorata* Kobayashi, 2017						x																			
*T.liangheensis* Liu et al., 2014						x	x				x														
*T.linaoshanica* Liu et al., 2014																			x						
*T.loeiensis* sp. n.						x																			
*T.longipenis* Liu et al., 2014						*	x		x		x								x						
*T.longzhouensis* Liu et al., 2014																			x						
*T.lucai* sp. n.									x																
*T.maerimensis* Kobayashi, 2018						x																			
*T.mininatula* (Moser, 1915)										x															
*T.maoershanensis* Liu et al., 2014																			x						
*T.margheritae* sp. n.						x																			
*T.masumotoi* Kobayashi, 2017						x																			
*T.matsumotoi* Kobayashi, 2017						x	x																		
*T.mengeana* Liu et al., 2014						*					x														
*T.menglongensis* Liu et al., 2014											x														
*T.microfurcata* sp. n.							x		x																
*T.microspinosa* sp. n.									x																
*T.multiangulata* sp. n.									x																
*T.nahaeoensis* sp. n.						x																			
*T.nakaiensis* sp. n.							x																		
*T.namnaoensis* sp. n.						x	x																		
*T.neouncinata* sp. n.							x			x															
*T.nonglomensis* sp. n.							x																		
*T.nussi* sp. n.								x																	
*T.olegi* sp. n.									x																
*T.pahinngamensis* sp. n.						x																			
*T.pailinensis* sp. n.								x																	
*T.parasetuliforceps* sp. n.						x																			
*T.paratonkinensis* sp. n.									x																
*T.petrpacholatkoi* sp. n.									x																
*T.phatoensis* sp. n.						x																			
*T.phoupaneensis* sp. n.							x																		
*T.phukradungensis* sp. n.						x																			
*T.pingjiangensis* Liu et al., 2014						x	x		x	x	x	x		x	x				x						
*T.pluriuncinata* sp. n.						x																			
*T.pseudoliangheensis* sp. n.							x																		
*T.pseudoruiliensis* sp. n.						x	x			x															
*T.pseudouncinata* sp. n.																			x						
*T.quadriforceps* sp. n.									x																
*T.quadrifurcata* sp. n.						x		x																	
*T.rihai* sp. n.						x																			
*T.romae* sp. n.									x																
*T.rubrithorax* sp. n.										x															
*T.rufimargo* Ahrens & Fabrizi, 2016					x																				
*T.ruiliana* Liu et al., 2014											x														
*T.ruiliensis* Liu et al., 2014						x			x	x	x														
*T.rungbongensis* Ahrens, 2004			x																						
*T.sapana* sp. n.									x																
*T.satura* (Brenske, 1898)						x				x	x														
*T.schneideri* Ahrens, 2004			x																						
*T.sculptilis* Liu et al., 2014									x		x			x											
*T.sejugata* (Brenske, 1898)										x															
*T.semidamadiensis* sp. n.							x																		
*T.semipingjiangensis* sp. n.						x																			
*T.semiruiliensis* sp. n.						x		x																	
*T.semishanensis* sp. n.						x				x															
*T.senohi* Kobayashi, 2018						x																			
*T.setuliforceps* sp. n.						x																			
*T.shanensis* sp. n.										x															
*T.shangsiensis* Liu et al., 2014									x										x	x					
*T.shunbiensis* Liu et al., 2014											x														
*T.siantarensis* (Moser, 1922)																								x	x
*T.sigulianshanica* Liu et al., 2014												x									x	x	x		
*T.smetsi* sp. n.								x																	
*T.soppongana* sp. n.						x																			
*T.spanglerorum* sp. n.						x																			
*T.spinicrus* (Frey, 1972)							x																		
*T.spinotibialis* sp. n.						x				x															
*T.subrotundata* sp. n.								x																	
*T.takahashii* Kobayashi, 2017						x			x																
*T.tanahrataensis* sp. n.																								x	
*T.thainguyensis* sp. n.									x																
*T.tianchiensis* Liu et al., 2014													x												
*T.tonkinensis* (Moser, 1908)									x																
*T.trilobiforceps* sp. n.							x																		
*T.ululalatensis* sp. n.																								x	
*T.umphangensis* sp. n.						x																			
*T.uncinata* Ahrens & Fabrizi, 2016					x																				
*T.univestris* Ahrens & Fabrizi, 2016					x																				
*T.vari* sp. n.						x		x																	
*T.veliformis* sp. n.						x	x																		
*T.vientianeensis* sp. n.							x																		
*T.vietnamensis* (Frey, 1969)									x																
*T.wandingensis* Liu et al., 2014											x														
*T.wangtongensis* Liu et al., 2014																	x	x							
*T.wapiensis* (Frey, 1972)						x	x																		
*T.wiangpapaoana* Kobayashi, 2017						x																			
*T.xichouensis* Liu et al., 2014											x														
*T.xiengkhouangensis* sp. n.							x																		
*T.yaoquensis* Liu et al., 2014							x				x														

**Table 2.** Genbank accessions of so far published Cox1 sequence data of *Tetraserica* species from Asia including voucher numbers and depository (see Dalstein et al. 2019).

Species	Voucher No.	Locality	Cox 1	Depository
*T.allosejugata* sp. n.	839475	Thailand: Pha Pok Mt. 1900-2000m	KT303284	ZFMK
*T.allosejugata* sp. n.	839484	Thailand: Ang Khang region, 1600m	KT303367	ZFMK
*T.appendiculata* sp. n.	X-DA4795	Laos: Stupa GH, 5km W Muang Sing, 750m,	MH171577	ZFMK
*T.auriculata* sp. n.	X-DA4700	Cambodia: Sihanouk-ville 20-40m	MH171565	ZFMK
*T.bachmaensis* sp. n.	X-DA4699	Vietnam: Tua Thien Hue Prov. Bach Ma Natl. Park, surr. Hotel Morin (1350–1400m)	MH171576	MZF
*T.chiangdaoensis* Kobayashi, 2017	X-DA4770	Laos: Stupa GH, 5km W Muang Sing, 750m,	MH171573	ZFMK
*T.chiangdaoensis* Kobayashi, 2017	X-DA4543	Thailand: Chiang Dao Hill Resort (100 km N of Chiang Mai) 600m	MH171599	ZFMK
*T.chiangdaoensis* Kobayashi, 2017	X-DA4714	Thailand: Chiang Dao Hill Resort 600m	MH171572	ZFMK
*T.chiangdaoensis* Kobayashi, 2017	X-DA4842	Thailand N: Chiang Dao Hill Resort (100 km N of Chiang Mai) 600m	MH171620	ZFMK
*T.chiangdaoensis* Kobayashi, 2017	X-DA4846	Thailand N: Chiang Dao Hill Resort (100 km N of Chiang Mai) 600m	MH171621	ZFMK
*T.chiangdaoensis* Kobayashi, 2017	X-DA4863	Thailand N: Chiang Dao Hill Resort (100 km N of Chiang Mai) 600m	MH171623	ZFMK
*T.chiangdaoensis* Kobayashi, 2017	X-DA4864	Thailand N: Chiang Dao Hill Resort (100 km N of Chiang Mai) 600m	MH171622	ZFMK
*T.chiangdaoensis* Kobayashi, 2017	835092	Thailand: Chiang Mai Pr., 114km N Chiang Mai Highway station, 700m	KT303338	ZFMK
*T.curviforceps* sp. n.	X-DA4511	Laos: Hua Phan prov.; Ban Saleui, Phou Pan (Mt.)	MH171534	ZFMK
*T.curviforceps* sp. n.	X-DA4566	Laos: Hua Phan prov.; Ban Saleui, Phou Pan (Mt.)	MH171603	ZFMK
*T.curviforceps* sp. n.	X-DA4570	Laos: Hua Phan prov.; Ban Saleui, Phou Pan (Mt.)	MH171607	ZFMK
*T.curviforceps* sp. n.	X-DA4577	Laos: Hua Phan prov.; Ban Saleui, Phou Pan (Mt.)	MH171608	ZFMK
*T.curviforceps* sp. n.	X-DA4583	Laos: Hua Phan prov.; Ban Saleui, Phou Pan (Mt.)	MH171604	ZFMK
*T.curviforceps* sp. n.	X-DA4588	Laos: Hua Phan prov.; Ban Saleui, Phou Pan (Mt.)	MH171606	ZFMK
*T.curviforceps* sp. n.	X-DA4593	Laos: Hua Phan prov.; Ban Saleui, Phou Pan (Mt.)	MH171605	ZFMK
*T.doisangensis* Kobayashi, 2017	X-DA4639	Thailand: Chiang Mai Doi Inthanon NP Vachirathan Fall 700m	MH171548	QSBG
*T.doisangensis* Kobayashi, 2017	X-DA4640	Thailand: Chiang Mai Doi Inthanon NP Vachirathan Fall 700m	MH171549	QSBG
*T.falciformis* sp. n.	X-DA4498	Laos: Attapeu prov. Annam Highlands Mts. Dong Amphan NBCA, ca. 1160 m Nong Fa	MH171520	ZFMK
*T.falciformis* sp. n.	X-DA4499	Laos: Attapeu prov. Annam Highlands Mts. Dong Amphan NBCA, ca. 1160 m Nong Fa	MH171521	ZFMK
*T.ferrugata* (Blanchard, 1850)	DA0001	Nepal: Darondi Khola, 650 m, 16.5.2006	KT303407	ZFMK
*T.filiforceps* sp. n.	X-DA4581	Laos: Hua Phan prov.; Ban Saleui, Phou Pan (Mt.)	MH171602	ZFMK
*T.kontumensis* sp. n.	X-DA4692	Vietnam: Kon Tum Prov. W border of Ngoc Linh Nat. Park, 20km N Dak Glei (900m)	MH171569	MZF
*T.liangheensis* Liu et al. 2014	LW1316	China: Yunnan, Lianghe	KJ959148	ZFMK
*T.liangheensis* Liu et al. 2014	X-DA2409	Laos: Xieng Khouang, Phonsavan (30 km NE), Phou Sane Mt., 1400-1600m	MH171581	ZFMK
*T.liangheensis* Liu et al. 2014	X-DA2439a	Laos: Xieng Khouang, Phonsavan (30 km NE), Phou Sane Mt., 1400-1600m	MH171583	ZFMK
*T.liangheensis* Liu et al. 2014	X-DA2439b	Laos: Xieng Khouang, Phonsavan (30 km NE), Phou Sane Mt., 1400-1600m	MH171582	ZFMK
*T.liangheensis* Liu et al. 2014	X-DA2443a	Laos: Xieng Khouang, Phonsavan (30 km NE), Phou Sane Mt., 1400-1600m	MH171584	ZFMK
*T.liangheensis* Liu et al. 2014	X-DA2443b	Laos: Xieng Khouang, Phonsavan (30 km NE), Phou Sane Mt., 1400-1600m	MH171585	ZFMK
*T.liangheensis* Liu et al. 2014	X-DA4504	Laos: Chiang Tung (Stupa) GH 5km SE Muang Sing, 750m	MH171522	ZFMK
*T.liangheensis* Liu et al. 2014	X-DA4505	Laos: Chiang Tung (Stupa) GH 5km SE Muang Sing, 750m	MH171523	ZFMK
*T.liangheensis* Liu et al. 2014	X-DA4508	Laos: Chiang Tung (Stupa) GH 5km SE Muang Sing, 750m	MH171524	ZFMK
*T.liangheensis* Liu et al. 2014	X-DA4509	Laos: Chiang Tung (Stupa) GH 5km SE Muang Sing, 750m	MH171525	ZFMK
*T.liangheensis* Liu et al. 2014	X-DA4513	Laos: Hua Phan prov.; Ban Saleui, Phou Pan (Mt.)	MH171526	ZFMK
*T.liangheensis* Liu et al. 2014	X-DA4562	Laos: Hua Phan prov.; Ban Saleui, Phou Pan (Mt.)	MH171600	ZFMK
*T.liangheensis* Liu et al. 2014	X-DA4812	Laos: Stupa GH, 5km W Muang Sing, 750m,	MH171619	ZFMK
*T.liangheensis* Liu et al., 2014	836953	Laos: Xieng Khouang, Phonsavan (30 km NE), Phou Sane Mt.	KT303169	ZFMK
*T.loeiensis* sp. n.	X-DA4647	Thailand: Loei Phu Ruea NP behind checkpoint 691m	MH171554	QSBG
*T.longipenis* Liu et al. 2014	X-DA4512	Laos: Hua Phan prov.; Ban Saleui, Phou Pan (Mt.)	MH171535	ZFMK
*T.maerimensis* Kobayashi, 2018	X-DA2309	N. Thailand: Doi Inthanon Nat. Res., 1250m	MH171586	ZFMK
*T.maerimensis* Kobayashi, 2018	X-DA2322	N. Thailand: Doi Inthanon Nat. Res., 1250m	MH171587	ZFMK
*T.maoershanensis* Liu et al. 2014	LW1066	China: Guangxi, Maoershan	KJ959149	ZFMK
*T.matsumotoi* Kobayashi, 2017	X-DA4660	Thailand: Chiang Dao Hill Resort 600m	MH171557	ZFMK
*T.matsumotoi* Kobayashi, 2017	X-DA4720	Thailand: Chiang Dao Hill Resort 600m	MH171566	ZFMK
*T.matsumotoi* Kobayashi, 2017	X-DA4722	Thailand: Chiang Dao Hill Resort 600m	MH171568	ZFMK
*T.matsumotoi* Kobayashi, 2017	X-DA4727	Thailand: Chiang Dao Hill Resort 600m	MH171567	ZFMK
*T.matsumotoi* Kobayashi, 2017	X-DA4869	Thailand N: Chiang Dao Hill Resort (100 km N of Chiang Mai) 600m	MH171625	ZFMK
*T.matsumotoi* Kobayashi, 2017	X-DA4956	Thailand N: Chiang Dao Hill Resort (100 km N of Chiang Mai) 600m	MH171626	ZFMK
*T.matsumotoi* Kobayashi, 2017	835112	Thailand: Chiang Dao Hill resort	KT303142	ZFMK
*T.namnaoensis* sp. n.	X-DA4881	Thailand N: Chiang Dao Hill Resort (100 km N of Chiang Mai) 600m	MH171624	ZFMK
*T.neouncinata* sp. n.	X-DA4582	Laos: Hua Phan prov.; Ban Saleui, Phou Pan (Mt.)	MH171601	ZFMK
*T.pahinngamensis* sp. n.	X-DA4630	Thailand: Chaiyaphum Pa Hin Ngam NP 461m	MH171550	QSBG
*T.pahinngamensis* sp. n.	X-DA4631	Thailand: Chaiyaphum Pa Hin Ngam NP 461m	MH171555	QSBG
*T.pahinngamensis* sp. n.	X-DA4648	Thailand: Chaiyaphum Pa Hin Ngam NP 698m	MH171556	QSBG
*T.pahinngamensis* sp. n.	X-DA4679	Thailand: Chaiyaphum Pa Hin Ngam NP 357m	MH171559	QSBG
*T.pahinngamensis* sp. n.	X-DA4680	Thailand: Chaiyaphum Pa Hin Ngam NP 357m	MH171560	QSBG
*T.pailinensis* sp. n.	X-DA4506	Cambodia: Pailin 200m	MH171538	ZFMK
*T.pailinensis* sp. n.	835104	Cambodia: Pailin, 270m	KT303124	ZFMK
*T.petrpacholatkoi* sp. n.	X-DA4697	Vietnam: Vinh Phuc Prov., Tam Dao Natinal Park (950m)	MH171571	MZF
*T.pingjiangensis* Liu et al. 2014	X-DA2491a	Thailand: Chiang Dao Hill resort (100km N of Chiang Mai), 600m	MH171588	ZFMK
*T.pingjiangensis* Liu et al. 2014	X-DA2491b	Thailand: Chiang Dao Hill resort (100km N of Chiang Mai), 600m	MH171593	ZFMK
*T.pingjiangensis* Liu et al. 2014	X-DA3435	N. Vietnam: Hanoi Prov., Ba Vi National Park	MH171589	MZF
*T.pingjiangensis* Liu et al. 2014	X-DA3436	N. Vietnam: Hanoi Prov., Ba Vi National Park	MH171590	MZF
*T.pingjiangensis* Liu et al. 2014	X-DA3437	N. Vietnam: Hanoi Prov., Ba Vi National Park	MH171594	MZF
*T.pingjiangensis* Liu et al. 2014	X-DA3439	N. Vietnam: Hanoi Prov., Ba Vi National Park	MH171592	MZF
*T.pingjiangensis* Liu et al. 2014	X-DA3985	Vietnam: Cao Bang Prov., vic. Vin Den, Nui Pia Oac Nat. res., 900-1300m	MH171591	ZFMK
*T.pingjiangensis* Liu et al. 2014	X-DA3987	Vietnam: Cao Bang Prov., vic. Vin Den, Nui Pia Oac Nat. res., 900-1300m	MH171595	ZFMK
*T.pingjiangensis* Liu et al. 2014	X-DA3988	Vietnam: Cao Bang Prov., vic. Vin Den, Nui Pia Oac Nat. res., 900-1300m	MH171596	ZFMK
*T.pingjiangensis* Liu et al. 2014	DA3434	N. Vietnam: Hanoi Prov., Ba Vi National Park	KT303293	MZF
*T.pingjiangensis* Liu et al. 2014	LW1076	China: Yunnan, Honghe, Hekou, Binlangzhai Shuiku	KJ959150	ZFMK
*T.pingjiangensis* Liu et al. 2014	X-DA4523	Laos NW: h=750m 5km SE Muang Sing Chiang Tung (Stupa) GH	MH171532	ZFMK
*T.pingjiangensis* Liu et al. 2014	X-DA4530	Laos NW: h=750m 5km SE Muang Sing Chiang Tung (Stupa) GH	MH171533	ZFMK
*T.pingjiangensis* Liu et al., 2014	839493	Thailand: Chiang Dao Hill resort	KT303319	ZFMK
*T.pseudoruiliensis* sp. n.	X-DA4519	Laos NW: h=750m 5km SE Muang Sing Chiang Tung (Stupa) GH	MH171528	ZFMK
*T.pseudoruiliensis* sp. n.	X-DA4520	Laos NW: h=750m 5km SE Muang Sing Chiang Tung (Stupa) GH	MH171529	ZFMK
*T.pseudoruiliensis* sp. n.	X-DA4529	Laos NW: h=750m 5km SE Muang Sing Chiang Tung (Stupa) GH	MH171530	ZFMK
*T.pseudoruiliensis* sp. n.	X-DA4536	Laos NW: h=750m 5km SE Muang Sing Chiang Tung (Stupa) GH	MH171531	ZFMK
*T.pseudoruiliensis* sp. n.	X-DA4604	Laos: Stupa GH, 5km W Muang Sing, 750m	MH171540	ZFMK
*T.pseudoruiliensis* sp. n.	X-DA4605	Laos: Stupa GH, 5km W Muang Sing, 750m	MH171541	ZFMK
*T.pseudoruiliensis* sp. n.	X-DA4610	Laos: Stupa GH, 5km W Muang Sing, 750m	MH171542	ZFMK
*T.pseudoruiliensis* sp. n.	X-DA4612	Laos: Stupa GH, 5km W Muang Sing, 750m	MH171543	ZFMK
*T.pseudoruiliensis* sp. n.	X-DA4623	Laos: Stupa GH, 5km W Muang Sing, 750m	MH171544	ZFMK
*T.pseudoruiliensis* sp. n.	X-DA4636	Thailand: Chiang Dao Hill Resort 600m	MH171546	ZFMK
*T.pseudoruiliensis* sp. n.	X-DA4637	Thailand: Chiang Dao Hill Resort 600m	MH171545	ZFMK
*T.pseudouncinata* sp. n.	X-DA4693	China: Guangxi A.R., Schiwandashan National Forest Park, 290-360m	MH171570	ZFMK
*T.romae* sp. n.	X-DA4501	Vietnam: Tuyen Quang pr. SE- E env. Of Na Hang, 200-700m	MH171527	ZFMK
*T.ruiliensis* Liu et al. 2014	LW1216	China: Yunnan, Ruili	KJ959151	ZFMK
*T.sigulianshanica* Liu et al. 2014	LW1129	China: Chongqing, Jinfoshan	KJ959152	ZFMK
*T.* sp.	DA1393	Philippines: Mindanao, Mt. Apo, L. Agco, 1400 m	KT303303	ZFMK
*T.* sp.	671344	Malaysia: Sabah, Croker Range NP. HQ Station, Road	EU084089	BYU
*T.spinicrus* (Frey, 1972)	X-DA4778	Laos: Stupa GH, 5km W Muang Sing, 750m,	MH171574	ZFMK
*T.spinicrus* (Frey, 1972)	X-DA4793	Laos: Stupa GH, 5km W Muang Sing, 750m,	MH171575	ZFMK
T. spKA3	DA3636	Kalimantan E: ca 55 km W of Balikpapan, PT Fajar Surya Swadaya [area] 82m	KT303085	BYU
*T.takahashii* Kobayashi, 2017	X-DA4502	Vietnam: Quang Binh prov. 1km N of Cha Lo, 400 m Vietnam- Laos border area	MH171536	ZFMK
*T.takahashii* Kobayashi, 2017	X-DA4503	Vietnam: Quang Binh prov. 1km N of Cha Lo, 400 m Vietnam- Laos border area	MH171537	ZFMK
*T.tanahrataensis* sp. n.	X-DA0347	W Malaysia, Kelantan, Road between Kampong Raja and Gua Musang, 1400-1700m	MH171579	ZFMK
*T.wandingensis* Liu et al., 2014	LW1247	China: Yunnan, Wanding	KJ959153	ZFMK
*T.wapiensis* (Frey, 1972)	X-DA2449	Thailand: Phetchabun Nam Nao NP Hill, 838m	MH171597	QSBG
*T.wapiensis* (Frey, 1972)	X-DA4646	Thailand: Petchabun Nam Nao NP Hill evergreen forest 834m	MH171551	QSBG
*T.wapiensis* (Frey, 1972)	X-DA4650	Thailand: Petchabun Nam Nao NP Hill evergreen forest 838m	MH171553	QSBG
*T.wapiensis* (Frey, 1972)	X-DA4651	Thailand: Petchabun Nam Nao NP Hill evergreen forest 838m	MH171552	QSBG
*T.wapiensis* (Frey, 1972)	X-DA4690	Thailand: Petchabun Nam Nao NP Hill evergreen forest 834m	MH171561	QSBG
*T.wapiensis* (Frey, 1972)	X-DA4708	Thailand: Petchabun Nam Nao NP Hill evergreen forest 883m	MH171563	QSBG
*T.wapiensis* (Frey, 1972)	X-DA4709	Thailand: Petchabun Nam Nao NP Hill evergreen forest 883m	MH171562	QSBG
*T.wapiensis* (Frey, 1972)	X-DA4710	Thailand: Petchabun Nam Nao NP Hill evergreen forest 883m	MH171564	QSBG
*T.wiangpapaoana* Kobayashi, 2017	X-DA2306	N. Thailand: Doi Inthanon Nat. Res., 1250m	MH171580	ZFMK
*T.wiangpapaoana* Kobayashi, 2017	836046	Thailand: N. Thailand: Doi Inthanon Nat. Res., 1250m	KT303404	ZFMK
*T.wiangpapaoana* Kobayashi, 2017	836048	Thailand: N. Thailand: Doi Inthanon Nat. Res., 1250m	KT303261	ZFMK
*T.yaoanica* Liu et al., 2014	LW1109	China: Guangxi, Shangsi Shiwandashan	KJ959154	ZFMK
*T.yaoanica* Liu et al., 2014	LW1257	China: Guangxi, Shangsi Shiwandashan	KJ959156	ZFMK
*T.yaoquensis* Liu et al., 2014	X-DA4627	Laos: Stupa GH, 5km W Muang Sing, 750m,	MH171547	ZFMK
